# Supplementary material for: Platform adaptive trial of novel antivirals for early treatment of COVID-19 In the community (PANORAMIC): protocol for a randomised, controlled, open-label, adaptive platform trial of community novel antiviral treatment of COVID-19 in people at increased risk of more severe disease
Source: BMJ Open. 2023 Aug 7;13(8):e069176. doi: 10.1136/bmjopen-2022-069176 (PMC10407406; doi:10.1136/bmjopen-2022-069176)
Supplement: Supplementary data [file bmjopen-2022-069176supp001.pdf]

Platform Adaptive trial of NOvel antiviRals for eArly treatMent of covid-19 In the Community: PANORAMIC

REC Number: 21/SC/0393 **IRAS Number: 1004274**

Chief Investigator: Professor Christopher Butler **Participant ID:** ______________________

CONSENT FORM

Thank you for completing the screening questionnaire, you have passed screening for the trial.

Please read the Participant Information Sheet (PIS) if you haven't already done so, and if you are willing to participate please select 'Yes', TYPE your FIRST and LAST names below and then click Submit

If you agree, please select 'Yes' to confirm that you have read and understood the following:

		YES	NO
1	I confirm I have read and understood the information sheet version number ____-________dated __ __ / __ __ __ / __ __ __ __ for the above study. I have had the opportunity to ask questions and had these answered satisfactorily.		
2	I understand my participation is voluntary and that I am free to withdraw at any time, without giving any reason, and without my medical care or legal rights being affected.		
3	I understand that if I chose to withdraw data already collected will continue to be used and I or my GP may be contacted if there are further questions regarding side-effects from trial treatments.		
4	I understand that I will be randomised to receive either: standard care plus an antiviral treatment or standard care, and that I will not be able to choose which I will receive.		
5	I understand that relevant sections of my GP and hospital medical notes and data collected during the study may be looked at by members of the research team and individuals from University of Oxford, both during and for up to 10 years after the scheduled follow-up period. It may also be reviewed by relevant people from regulatory authorities and from NHS Organisation(s). I give permission for these individuals to have access to my records which identify me by name.		
6	I understand that my personal information may be shared with University of Dundee's Health Informatics Centre and that my date of birth and/or my NHS number (or equivalent UK NHS Identifier) will be shared with NHS Digital**,** electronic Data Research and Innovation Service (eDRIS), The Secure Anonymised Information Linkage (SAIL) Databank or Health and Social Care Northern Ireland (HSC Business Services Organisation/HSC Trusts) (HSC NI) to enable them to supply the study team with additional healthcare data about me, which is relevant to the trial. The data supplied by NHS Digital, eDRIS, SAIL or HSC NI is linked by the research team to the data collected during my participation in the trial. I am free to withdraw my consent for data linkage with NHS Digital, eDRIS, SAIL or HSC NI at any time and it will not affect my ongoing care.		
7	I understand that members of the research team may view my general practice and hospital medical records, including the summaries of my medical records (e.g. Summary Care Record (SCR), Emergency Care Summary (ECS), The GP Summary, Northern Ireland		

NUFFIELD DEPARTMENT OF PRIMARY CARE HEALTH SCIENCES Primary Care Clinical Trials Unit

PANORAMIC Platform Adaptive trial of NOvel antiviRals for eArly treatMent of COVID-19 In the Community

	Electronic Care Record, and the Welsh Clinical Portal to check my medication, allergies, adverse reactions, and additional information to make sure that it is safe for me to take trial medication. I give permission for these individuals to access my medical records for this purpose.		
8	I consent to being contacted by the research team for the purposes of trial follow up (up to 6 months) and I understand that this will require me to provide my contact details to the research team.		
9	I consent to my GP and/or Care Home being informed of my participation within the study, and I understand that the trial team may contact my GP about my ongoing participation in the trial.		
10	I understand that the information collected about me may be shared in a form that cannot identify me with commercial companies to support the licensing of trial treatments, within the UK and abroad.		
11	I agree to take part in the trial.		
	For participants capable of being pregnant (regardless of current contraception methods) *(to show only for those who meet this criterion in the screening form)*		
12	I agree to taking a pregnancy test prior to taking the trial treatment.		
13	I understand that I must use reliable methods of contraception (as specified in the PIS appendices). I agree to provide information requested on any pregnancy, including pregnancy outcome, occurring within 28-days following first administration of the IMP, as requested by the MHRA. I understand that if I report a pregnancy the Sponsor will report this to The UK Teratology Information Service (UKTIS).		
	ADDITIONAL (optional, not required for study participation)	**YES**	**NO**
14	I agree to provide the research team with the contact details of my Trial Partner. I confirm my Trial partner is aware of their role and willing to answer questions.		
15	I agree to take part in the Virology Sampled Cohort.		
	For Participants Agreeing to take part in Virology Study		
16	I agree to donate blood and nasopharyngeal samples. I consider these samples a gift to the University of Oxford, and I understand I will not gain any direct personal or financial benefit from them. I understand that even if I withdraw from the above study, the samples collected from me may still be used in the study analysis.		

If you are the participant completing the consent form, please provide your signature below

Participant Signature: ______________________________

Gbinigie O, *et al. BMJ Open* 2023; 13:e069176. doi: 10.1136/bmjopen-2022-069176

NUFFIELD DEPARTMENT OF PRIMARY CARE HEALTH SCIENCES Primary Care Clinical Trials Unit

PANORAMIC Platform Adaptive trial of NOvel antiviRals for eArly treatMent of COVID-19 In the Community

First Name: ______________________ ______________________
Last Name:

Date: __ __ / __ __ / __ __ __ __

If the participant has provided verbal consent, but they are unable to complete the consent form due to lack of online access, too unwell, too frail or consent is completed via the telephone (the participant must have capacity), please provide:

1. **Name of the participant:**

First Name: ______________________ **Last Name:** ______________________

Date: __ __ / __ __ / __ __ __ __

2. **Signature of person completing the form:**

First Name: ______________________ **Last Name:** ______________________

Role: Trial partner/trial team member/Health Care Professional

Date: __ __ / __ __ / __ __ __ __

If participant lacks capacity to give consent:

I have read the information (or had it read to me), had an opportunity to ask questions and signed the *Legal Representative Letter*. I understand that the patient will be asked to confirm their consent as soon as they have the capacity to do so and that if they wish, they will be able to withdraw from the trial without it affecting their medical care.

Participant:

Name: ______________________ Date: __ __ / __ __ / __ __ __ __

I believe that if they were able to, the patient would wish to take part in this trial.

______________________ ______________________

PRINTED name of Legal Representative **Signature of Legal Representative**

Today's date__ __ / __ __ / __ __ __ __

Gbinigie O, *et al. BMJ Open* 2023; 13:e069176. doi: 10.1136/bmjopen-2022-069176

Relationship to participant (as confirmed in the signed *Legal Representative Letter*)

____

You will have the opportunity to print a copy of the consent form after submission. Please contact the study team if you would like a copy sent to you.

By submitting, I confirm that I am the person whose name is stated above.

If you have any questions about consent or the trial, please contact the study team:

Tel: 08081 560017 Email panoramic@phc.ox.ac.uk

Gbinigie O, *et al. BMJ Open* 2023; 13:e069176. doi: 10.1136/bmjopen-2022-069176
