# Supplementary material for: Platform adaptive trial of novel antivirals for early treatment of COVID-19 In the community (PANORAMIC): protocol for a randomised, controlled, open-label, adaptive platform trial of community novel antiviral treatment of COVID-19 in people at increased risk of more severe disease
Source: BMJ Open. 2023 Aug 7;13(8):e069176. doi: 10.1136/bmjopen-2022-069176 (PMC10407406; doi:10.1136/bmjopen-2022-069176)
Supplement: Author's manuscript [file bmjopen-2022-069176.draft_revisions.pdf]

BMJ Open is committed to open peer review. As part of this commitment we make the peer review history of every article we publish publicly available.

When an article is published we post the peer reviewers' comments and the authors' responses online. We also post the versions of the paper that were used during peer review. These are the versions that the peer review comments apply to.

The versions of the paper that follow are the versions that were submitted during the peer review process. They are not the versions of record or the final published versions. They should not be cited or distributed as the published version of this manuscript.

BMJ Open is an open access journal and the full, final, typeset and author-corrected version of record of the manuscript is available on our site with no access controls, subscription charges or pay-per-view fees (http://bmjopen.bmj.com).

If you have any questions on BMJ Open's open peer review process please email info.bmjopen@bmj.com

BMJ Open

Platform Adaptive trial of NOvel antiviRals for eArly treatMent of COVID-19 In the Community (PANORAMIC): protocol for a randomised, controlled, open-label, adaptive platform trial of community novel antiviral treatment of COVID-19 in people at increased risk of more severe disease.

Journal:	*BMJ Open*
Manuscript ID	bmjopen-2022-069176
Article Type:	Protocol
Date Submitted by the Author:	16-Oct-2022

Complete List of Authors:	Gbinigie, Oghenekome; University of Oxford, Nuffield Department of Primary Care Health Sciences<br>Ogburn, Emma; University of Oxford Nuffield Department of Primary Care Health Sciences<br>Allen, Julie; University of Oxford, Nuffield Department of Primary Care Health Sciences<br>Dorward, Jienchi; University of Oxford, Nuffield Department of Primary Care Health Sciences; Centre for the Aids Programme of Research in South Africa,<br>Dobson, Melissa; Oxford Respiratory Trials Unit, Nuffield Department of Medicine<br>Madden, Tracie-Ann; Centre for Trials Research, Cardiff University<br>Yu, Ly-Mee; University of Oxford Nuffield Department of Primary Care Health Sciences, Department of Primary Care Health Sciences<br>Lowe, David; Institute of Immunity and Transplantation, University College London<br>Rahman, Najib; Oxford Respiratory Trials Unit, Nuffield Department of Medicine; NIHR Oxford Biomedical Research Centre<br>Petrou, Stavros; Nuffield Department of Primary Care Health Sciences<br>Richards, Duncan; University of Oxford, NDORMS<br>Hood, Kerenza; Centre for Trials Research, Cardiff University<br>Patel, Mahendra; University of Oxford Nuffield Department of Primary Care Health Sciences<br>Saville, Benjamin; Berry Consultants; Vanderbilt University Medical Center Department of Biostatistics<br>Marion, Joe; Berry Consultants<br>Holmes, Jane; University of Oxford Nuffield Department of Primary Care Health Sciences<br>Png, May Ee; University of Oxford, Nuffield Department of Primary Care Health Sciences<br>Hayward, Gail; University of Oxford, Nuffield Department of Primary Care Health Sciences<br>`Lown, Mark; University of Southampton, Primary Care and Population Sciences<br>Harris, Victoria; University of Oxford Nuffield Department of Primary

Care Health Sciences
Jani, Bhautesh; University of Glasgow , 12. General Practice and Primary Care, School of Health and Wellbeing, MVLS
Hart, Nigel; Queen's University Belfast, School of Medicine, Dentistry and Biomedical Sciences - Centre for Public Health
Khoo, Saye; Department of Pharmacology, University of Liverpool
Rutter, Heather ; University of Oxford Nuffield Department of Primary Care Health Sciences
Chalk, Jem; University of Oxford Nuffield Department of Primary Care Health Sciences
Standing, Joseph; UCL Great Ormond Street Institute of Child Health Population Policy and Practice, Infection Inflammation and Immunology; Great Ormond Street Hospital for Children, Department of Pharmacy
Breuer, Judith; UCL Great Ormond Street Institute of Child Health Population Policy and Practice, Infection Inflammation and Immunology
Lavallee, Layla; University of Oxford Nuffield Department of Primary Care Health Sciences
Hadley, Elizabeth; University of Oxford Nuffield Department of Primary Care Health Sciences
Cureton, Lucy; University of Oxford Nuffield Department of Primary Care Health Sciences
Benysek, Magdalena; University of Oxford Nuffield Department of Primary Care Health Sciences
Andersson, Monique; Oxford University Hospitals NHS Foundation Trust, Department of Microbiology
Francis, Nick; University of Southampton, School of Primary Care Population Sciences and Medical Education
Thomas, Nicholas; Windrush Medical Practice; NIHR Clinical Research Network Thames Valley and South Midlands
Evans, Philip; University of Exeter, Faculty of Health and Life Sciences; National Institute for Health Research Clinical Research Network
van Hecke, Oliver; University of Oxford Nuffield Department of Primary Care Health Sciences
Koshkouei, Mona; University of Oxford, Nuffield Department of Primary Care Health Sciences
Coates, Maria; University of Oxford Nuffield Department of Primary Care Health Sciences
Barrett, Sarah; University of Oxford Nuffield Department of Primary Care Health Sciences
Bateman, Clare; University of Oxford Nuffield Department of Primary Care Health Sciences
Davies, Jennifer; University of Oxford Nuffield Department of Primary Care Health Sciences
Raymundo-Wood, Ivy; University of Oxford Nuffield Department of Primary Care Health Sciences
Ustianowski, Andrew; North Manchester General Hospital, Regional Infectious Diseases Unit
Nguyen-Van-Tam, Jonathan; University of Nottingham School of Medicine, Lifespan and Population Health Unit
Carson-Stevens, A; Cardiff University, Centre for Trials Research
Hobbs, Richard; University of Oxford Nuffield Department of Primary Care Health Sciences
Little, Paul; University of Southampton, Primary Care Research Centre
Butler, Christopher C.; University of Oxford, Nuffield Department of Primary Health Care Sciences

Keywords: COVID-19, Clinical trials < THERAPEUTICS, VIROLOGY, PRIMARY CARE, THERAPEUTICS

SCHOLARONE™
Manuscripts

Platform Adaptive trial of NOvel antiviRals for eArly treatMent of COVID-19 In the Community (PANORAMIC): protocol for a randomised, controlled, open-label, adaptive platform trial of community novel antiviral treatment of COVID-19 in people at increased risk of more severe disease.

For peer review only

Oghenekome A Gbinigie[1ǂ]

Emma Ogburn[1ǂ]

Julie Allen[1ǂ]

Jienchi Dorward[1,2]

Melissa Dobson[3]

Tracie-Ann Madden[4]

Ly-Mee Yu[1]

David M Lowe[5]

Najib M Rahman[3,6,7]

Stavros Petrou[1]

Duncan B Richards[8]

Kerenza Hood[4]

Mahendra G Patel[1]

Benjamin R Saville[9,10]

Joe Marion[9]

Jane Holmes[1]

May Ee Png[1]

Gail Hayward[1]

Mark Lown[11]

For peer review only - http://bmjopen.bmj.com/site/about/guidelines.xhtml

Victoria Harris[1]

Bhautesh Jani[12]

Nigel D Hart[13]

Saye Khoo[14]

Heather Rutter[1]

Jem Chalk[1]

Joseph F Standing[15,16]

Judith Breuer[15]

Layla Lavallee[1]

Elizabeth Hadley[1]

Lucy Cureton[1]

Magdalena Benysek[1]

Monique Andersson[17]

Nick Francis[11]

Nicholas P B Thomas[18,19,20]

Philip Evans[21,22]

Oliver van Hecke[1]

Mona Koshkouei[1]

Maria Coates[1]

Sarah Barrett[1]

Clare Bateman[1]

Jennifer C Davies[1]

Ivy Raymundo-Wood[1]

Andrew Ustianowski[23]

Jonathan S Nguyen-Van-Tam[24]

For peer review only

Andrew Carson-Stevens[4]

F.D. Richard Hobbs[1†]

Paul Little[11†]

Christopher C Butler[1†]

Corresponding author - Christopher C Butler christopher.butler@phc.ox.ac.uk

Postal address: Nuffield Department of Primary Care Health Sciences, University of Oxford, Woodstock Road, Oxford, OX2 6GG

[ǂ] Joint first authors

[†]Joint senior authors

For peer review only

Author affiliations

1. Nuffield Department of Primary Care Health Sciences, University of Oxford, Oxford, UK
2. Centre for the AIDS Programme of Research in South Africa (CAPRISA), University of KwaZulu–Natal, Durban, South Africa.
3. Oxford Respiratory Trials Unit, Nuffield Department of Medicine, University of Oxford, UK
4. Centre for Trials Research, Cardiff University, UK
5. Institute of Immunity and Transplantation, University College London, London, UK
6. Oxford NIHR Biomedical Research Centre, UK
7. Chinese Academy of Medicine Oxford Institute, University of Oxford, UK
8. Nuffield Department of Orthopaedics, Rheumatology and Musculoskeletal Sciences, University of Oxford, Oxford, UK
9. Berry Consultants, Austin, Texas, USA
10. Department of Biostatistics, Vanderbilt University School of Medicine, Tennessee, USA

11. Primary Care Research Centre, University of Southampton, Southampton, UK
12. General Practice and Primary Care, School of Health and Wellbeing, MVLS, University of Glasgow, UK
13. School of Medicine, Dentistry and Biomedical Sciences, Queen’s University Belfast, Northern Ireland, UK
14. Department of Pharmacology, University of Liverpool, Liverpool, UK
15. Infection Inflammation and Immunology, UCL Great Ormond Street Institute of Child Health, London, UK
16. Department of Pharmacy, Great Ormond Street Hospital for Children, London, UK
17. Department of Microbiology, Oxford University Hospitals NHS Foundation Trust, Oxford, UK
18. Windrush Medical Practice, Witney, UK
19. National Institute for Health and Care Research, Thames Valley and South Midlands Clinical Research Network, UK
20. Royal College of General Practitioners, London, UK
21. Faculty of Health and Life Sciences, University of Exeter, UK
22. National Institute for Health and Care Research Clinical Research Network, UK
23. Regional Infectious Diseases Unit, North Manchester General Hospital, Manchester, UK
24. Lifespan and Population Health Unit, University of Nottingham School of Medicine, Nottingham, UK

For peer review only

Word count: 5393/4000

For peer review only - http://bmjopen.bmj.com/site/about/guidelines.xhtml

ABSTRACT

Introduction: There is an urgent need to determine the safety, effectiveness and cost-effectiveness of novel antiviral treatments for COVID-19 in vaccinated patients in the community at increased risk of morbidity and mortality from COVID-19.

Methods and analysis: PANORAMIC is a UK-wide, open-label, prospective, adaptive, multi-arm platform, randomised clinical trial that evaluates antiviral treatments for COVID-19 in the community. A master protocol governs the addition of new antiviral treatments as they become available, and the introduction and cessation of existing interventions via interim analyses. The first two interventions to be evaluated are molnupiravir (Lagevrio) and nirmatrelvir/ritonavir (Paxlovid). Eligibility criteria: community-dwelling within five days of onset of symptomatic COVID-19 (confirmed by PCR or lateral flow test), and either (1) aged 50 years and over, or (2) aged 18-49 years with qualifying co-morbidities. Registration occurs via the trial website and by telephone. Recruitment occurs remotely through the central trial team, or in person through clinical sites. Participants are randomised to receive either usual care or a trial drug plus usual care. Outcomes are collected via a participant-completed daily electronic symptom diary for 28 days post randomisation. Participants and/or their Trial Partner are contacted by the research team after days 7, 14 and 28 if the diary is not completed, or if the participant is unable to access the diary. The primary efficacy endpoint is all-cause, non-elective hospitalisation and/or death within 28 days of randomisation. Multiple pre-specified interim analyses allow interventions to be stopped for futility or superiority based on pre-specified decision criteria. A prospective economic evaluation is embedded within the trial.

For peer review only

Ethics and dissemination: Ethical approval granted by South Central–Berkshire REC number: 21/SC/0393; IRAS project ID: 1004274. Results will be presented to policymakers and at conferences, and published in peer-reviewed journals.

Trial registration number: ISRCTN 30448031; EudraCT number: 2021-005748-31

296/300

For peer review only

For peer review only - http://bmjopen.bmj.com/site/about/guidelines.xhtml

STRENGTHS AND LIMITATIONS

- Efficacy studies with short-term follow-up and prior to the omicron strain becoming prevalent found that novel antiviral agents benefit unvaccinated people with COVID-19, however, PANORAMIC will add to the evidence base by determining: effectiveness in COVID multiply-vaccinated populations while current circulating SARS-CoV-2 variants are prevalent; the impact on viral load and antiviral resistance; the impact on longer term symptoms; and, cost-effectiveness.
- PANORAMIC is a platform trial: interventions can be added as the trial progresses, and interim analyses allow interventions to be dropped as soon as pre-specified criteria for superiority or futility are met, or for safety concerns, which may provide results to inform clinical care more rapidly than other approaches.
- In addition to recruitment by investigators at research sites, the research can be delivered "direct-to-patient" through recruitment by a centralised team, with remote consent, follow-up, and delivery of study medication to participants' homes, thereby not limiting participation to where people live or receive their health care, and increasing applicability of our findings to routine health care.
- A national inclusion and diversity strategy has been employed to actively promote the trial across the four UK nations to diverse communities and people from all backgrounds collaborating with the NIHR Clinical Research Network and equivalent networks in UK devolved administrations
- The open-label design means that it is not possible to quantify the contribution of any placebo-effect to treatment effects, but is more closely reflective of real-world practice.

For peer review only - http://bmjopen.bmj.com/site/about/guidelines.xhtml

INTRODUCTION

The development and roll-out of national Coronavirus disease 2019 (COVID-19) vaccination schemes has been transformative in reducing disease severity and to a lesser extent SARS-CoV-2 transmission. [1-3] Despite this, the emergence of new variants and waning immunity have led to intermittent surges in COVID-19 cases and hospitalisations. [4] The implementation of effective COVID-19 treatments therefore remains a critical management strategy and may be of great importance if future vaccine-escaping variants emerge. A number of drugs have been trialled as re-purposed COVID-19 community treatments with evidence that some should not be used for this indication [5 6] while others are likely to be beneficial. [7 8] Directly-acting antiviral drugs are an important therapeutic approach, but evidence is limited.

Two new antiviral options are molnupiravir (Lagevrio) and nirmatrelvir/ritonavir (Paxlovid), with others being developed. Molnupiravir is a prodrug; the ribonucleoside analogue β-d-N4 -hydroxycytidine (NHC) is metabolised to NHC-triphosphate in cells, which when integrated introduces catastrophic hypermutation. [9] Paxlovid is a combination of nirmatrelvir and ritonavir; nirmatrelvir inhibits the activity of the SARS-CoV-2 3-CL protease that is necessary for viral replication [10], and ritonavir significantly slows the clearance of nirmatrelvir.[11]

Initial trials of molnupiravir and nirmatrelvir/ritonavir for COVID-19 have demonstrated safety and efficacy. [12 13] However, these trials were in unvaccinated patients prior to the omicron SARS-CoV-2 variant becoming prevalent, and it is not clear if there are particular subgroups of patients who should be prioritised for treatment. Furthermore, the impact on viral load, antiviral resistance and emergence of new variants requires further evaluation, and cost-effectiveness of these agents at scale is as yet unknown. The impact on long COVID is

For peer review only - http://bmjopen.bmj.com/site/about/guidelines.xhtml

also yet to be assessed. Nevertheless, these encouraging efficacy trials, and the likelihood that further plausible treatments will become available and require evaluation, justifies a large-scale, ongoing, pragmatic evaluation of antiviral treatments for use in the community in a largely vaccinated population, while current variants are circulating, to rapidly generate robust evidence for guiding decisions about widespread deployment.

We therefore established an adaptive multi-arm platform trial with a master protocol to test whether novel antiviral agents are safe, effective and cost-effective treatments for people in the community with COVID-19 who are at increased risk of an adverse outcome.

Objective

To assess the effectiveness and cost effectiveness of novel antiviral treatments in reducing all-cause, non-elective hospitalisation and/or death within 28 days of randomisation among patients with test-positive COVID-19 in the community and who are at increased risk of requiring hospital treatment.

METHODS AND ANALYSIS

Trial Design

The Platform Adaptive trial of Novel antiviRals for eArly treatment of COVID-19 in the Community (PANORAMIC) is an open-label, prospective, adaptive platform, randomised clinical trial in community care.

A multi-arm 'platform trial' is a clinical trial that allows for multiple treatments for the same disease to be tested simultaneously under a single master protocol. Pre-specified adaptations

For peer review only - http://bmjopen.bmj.com/site/about/guidelines.xhtml

allow interventions to be added to the trial, or stopped for futility or superiority whilst the trial is in progress through pre-specified interim analyses. [14 15] Participants are randomly assigned to either usual care, or usual care plus a trial intervention. Usual care represents the standard care that participants would receive via the National Health Service (NHS).

The master protocol defines *a priori* decision rules to allow for dropping a treatment for futility or declaring a treatment superior to usual care. [16] If at an interim analysis, usual care plus an antiviral is deemed superior to usual care alone for the primary endpoint of all-cause, non-elective hospitalisation and/or death within 28 days of randomisation, the superior treatment may be incorporated into usual care as the new standard of care. Cost-effectiveness will also be assessed. A subset of participants is additionally enrolled into a virology sub-study, and are asked to provide nasopharyngeal swabs and fingerpick blood samples at intervals over the 14 days following recruitment.

The first and second antivirals to be evaluated in PANORAMIC are molnupiravir and nirmatrelvir/ritonavir, respectively.

Patient and Public Involvement (PPI)

PPI contributors contribute to refining the study question, design, implementation, interpretation and dissemination of findings. At trial conception, the aims and design of the study were discussed with members of the public who had experience of COVID-19, either personally or through household members, and who were at higher risk of complications from COVID-19. PPI groups supporting the trial include an ethnically diverse main study PPI group who have advised on patient facing documents and study processes, and have helped to draft easy read versions of study documents. In addition, bespoke PPI groups established in

For peer review only - http://bmjopen.bmj.com/site/about/guidelines.xhtml

Northern Ireland, Scotland and Wales have advised on data capture and recruitment processes specific to their local health systems, and will contribute to advise on dissemination. Two PPI contributors sit on the Trial Steering Committee to help guide trial progress. A co-investigator has a specific remit for community engagement, developing and implanting initiatives with the support of pharmacy networks to ensure uptake especially in areas of higher social deprivation and among minority ethnic groups: feedback about all aspects of the trial is received from this community engagement program.

Study Setting

The trial is implemented by the University of Oxford Primary Care and Vaccines Collaborative Clinical Trials Unit (PCV-CTU)[17] with further support from the Oxford Respiratory Trials Unit and the Centre for Trials Research, Cardiff University, supported by the National Institute of Health and Care Research Clinical Research Network, the National Institute of Health and Care Research, and the Department of Health and Social Care (and equivalents in devolved administrations).

The PCV-CTU is able to act as a central recruiting site, and PANORAMIC Hubs act as clinical recruitment sites. PANORAMIC Hubs are clinical sites that include GP sites as single practices or a federation of practices that are able to operate under a single site agreement with a Principal Investigator to undertake study procedures as detailed in the master protocol. Hubs can include GP practices, community trusts, and other healthcare providers. Potential participants can be referred to Hubs by other healthcare facilities for screening. As well as recruiting patients through routine consultations, Hubs perform database searches for COVID-19 positive test results in registered patients who are clinically vulnerable (see Table

For peer review only - http://bmjopen.bmj.com/site/about/guidelines.xhtml

1), and invite them to take part in the trial. All mandated study procedures can be conducted remotely, in keeping with the prevailing self-isolation advisory governmental guidance for patients with COVID-19 in the community. [18]

Table 1: Criteria considered to make a potential participant at higher risk of worse outcomes from COVID-19

- Chronic respiratory disease (including chronic obstructive pulmonary disease (COPD), cystic fibrosis and asthma requiring at least daily use of preventative and/or reliever medication)
- Chronic heart or vascular disease
- Chronic kidney disease
- Chronic liver disease
- Chronic neurological disease (including dementia, stroke, epilepsy)
- Severe and profound learning disability
- Down's syndrome
- Diabetes mellitus (Type 1 or Type 2)
- Immunosuppression: primary (e.g., inherited immune disorders resulting from genetic mutations, usually present at birth and diagnosed in childhood) or secondary due to disease or treatment (e.g., sickle cell, HIV, cancer, chemotherapy)
- Solid organ, bone marrow and stem cell transplant recipients
- Morbid obesity (BMI >35 kg/m²)
- Severe mental illness
- Care home resident
- Judged by recruiting medically qualified professional, research nurse, nurse prescriber, prescribing pharmacist, dependent on the Intervention Specific Appendix for the specific antiviral involved, to be clinically vulnerable

Eligibility criteria

The inclusion criteria are: patient or their legal representative is able and willing to provide informed consent; patient presenting with symptoms attributable to COVID-19 starting within the past five days and ongoing; patient has a positive SARS-CoV-2 test (lateral flow test and/or PCR) between two days of symptom onset and randomisation; and, patient is aged ≥50 years or aged 18-49 years with an underlying chronic health condition considered to make them clinically vulnerable (see Table 1). Exclusion criteria are: patient currently

For peer review only - http://bmjopen.bmj.com/site/about/guidelines.xhtml

admitted to hospital (inpatient); patient previously randomised in the PANORAMIC trial; and, patient currently participating in a clinical trial of a therapeutic agent for acute COVID-19. Additional exclusion criteria specific to each intervention arm, if any, are listed in the Intervention Specific Appendices (ISAs) of trial arms within the master protocol. Patients must be eligible for at least two arms (Usual Care and at least one novel antiviral intervention).

Study procedures

Recruitment

The entire recruitment process can be done remotely as well as in person. Potential participants can register via the trial website, through a free-phone telephone call to the central trial team, or via a PANORAMIC hub.

Informed consent, screening and enrolment

Eligibility is assessed at a PANORAMIC Hub, other NHS healthcare provider, or by the central clinical trial team, by a suitably trained and experienced medically qualified professional, research nurse, nurse prescriber or prescribing pharmacist, as determined by the regulator and specified in the ISA for the specific antiviral involved.

Prospective participants are provided with written, pictorial and/or verbal versions of the Patient Information Sheet (PIS), detailing the nature of the trial and the known side-effects/risks involved in taking part. Prospective participants with capacity and being recruited in-person provide written informed consent (see additional file 1). During a two-way discussion (apart from with those who lack capacity to do this) either face-to-face or by a telephone/video call from, participants provide their consent to participate and this is recorded.

For peer review only

Participants recruited remotely provide consent using an electronic consent form that is held securely on the trial database. Consent forms can be downloaded and sent to participants if requested, and are sent to their GPs if participants are recruited centrally. Consent forms for participants recruited in-person via PANORAMIC Hubs are filed in participants' medical notes, with a printed copy given to the participant.

Prospective participants lacking capacity to consent are only eligible if they live in a care home. If the recruiting healthcare professional considers that a patient in a care home lacks capacity to provide consent for themselves, then a personal or professional legal representative (England and Wales only) is asked to provide consent in-person or remotely.

Participants who are unable or too unwell to complete baseline information or respond to surveys for themselves can identify a Trial Partner to assist them in: completing the initial screening questionnaire and baseline information; completing the informed consent forms; and, completing the electronic symptom diary (see 'follow-up' section). A letter is issued to Trial Partners, informing them of the study and notifying them that they have been nominated for this role by the prospective participant.

Randomisation and blinding

Participants are randomised using a secure, fully validated, and compliant web-based randomisation system embedded within Spinnaker (a data entry system), with stratification by age and vaccination status. Participants are randomised to one trial arm using fixed equal allocation ratios corresponding to the number of eligible arms in the trial. For example, if there are two active interventions (A & B), the allocation ratio will be 1:1:1 for Usual Care,

For peer review only - http://bmjopen.bmj.com/site/about/guidelines.xhtml

active A, active B (respectively), such that 33% of participants are randomised to Usual Care. If there are three active interventions, the allocation ratio will be 1:1:1:1, such that 25% of participants are randomised to Usual Care. As this is a nationwide, individually randomised trial that aims to include large numbers of participants, individual participant characteristics and infecting strain types of SARS-CoV-2 are expected to be equally distributed between trial arms.

PANORAMIC is an open-label trial. The participant, legal representative (if applicable), and recruiting clinician know the participant's allocation. The trial team and recruiting clinicians are kept blind to emerging results of interim analyses. Only the unblinded statisticians and the independent members of the Data and Safety Monitoring Committee (DSMC) have access to unblinded interim results corresponding to a given intervention, until such time as a decision is made to close the intervention.

Baseline assessments

During screening and enrolment, participants and/or recruiting clinicians record baseline data including: date of birth; sex; ethnicity; vaccination status; qualifying co-morbidities; symptoms and severity; a measure of their health-related quality of life (EuroQoL EQ-5D-5L); [19] number of household contacts; and, whether they have received a monoclonal antibody treatment for COVID-19.

Interventions

PANORAMIC trial is testing novel antiviral agents that have the potential to be widely used to treat COVID-19 in the community. Each agent is fully specified in an ISA. The antiviral

For peer review only - http://bmjopen.bmj.com/site/about/guidelines.xhtml

drugs are couriered to participants, typically within 24 hours of randomisation. Pharmacies can supply antivirals to participants via community pharmacy services or online pharmacy services. The antivirals can also be collected from a pharmacy by the participant (or someone on their behalf, with appropriate infection control measures).

PANORAMIC is a randomised controlled, open-label, pragmatic trial. [20 21] The control arm is Usual Care. Usual Care can include antiviral treatment available to individual patients in routine care in the NHS. [22] In the UK, patients at highest risk are able to access antiviral treatments directly from the NHS via COVID Medicine Delivery Units (CMDUs) and analogous organisations; otherwise, in the absence of complicated infection (e.g. bacterial super-infection), Usual Care in the NHS is generally supportive. [22] Participants assigned to an intervention arm additionally receive the usual care through the NHS that they would ordinarily have received, had they not participated in the trial. The trial team are not involved in making clinical or clinical management decisions for participants.

Follow-up

Following randomisation, participants in the intervention arm receive a participant pack containing: the allocated antiviral agent; an information booklet; a participant card detailing how the medication should be administered, precautions and safety guidance; a medication appendix providing further information about the allocated intervention; an emergency card with a phone number with a 24 hour phone line to access an on-call clinician for safety concerns; and, a pregnancy test to be used by participants of child-bearing potential for certain interventions.

For peer review only - http://bmjopen.bmj.com/site/about/guidelines.xhtml

All participants are emailed a link each day to an online symptom diary and are asked to complete it daily for 28 days. Participants are asked: to rate a variety of symptoms (such as fever, cough, breathlessness and fatigue) on an ordinal scale (e.g. ‘no problem,’ ‘mild problem,’ ‘moderate problem’ or ‘major problem’); whether they have been hospitalised or required contact with health and social services; how they are feeling on a scale of zero to 10 (zero being the worst one can imagine, and 10 being the best one can imagine); whether they feel fully recovered; whether they are taking over-the-counter medication; whether the number of people in the household has changed; confirm whether they have taken the antiviral agent (if applicable); and, at fortnightly intervals the EQ-5D-5L to assess their health-related quality of life. The central trial team calls participants/Trial Partners with no internet access and those who have not completed their diary for at least two consecutive days before days 7, 14 and 28.

All participants receive a phone call from the trial team on Day 2 of the trial to confirm receipt of trial materials, confirm consent and understanding of follow-up procedures, and to answer any queries. Participants receiving an antiviral agent receive additional safety calls from members of the trial team, to determine whether participants are experiencing adverse effects, and, if applicable, to ensure that participants who are physiologically capable of becoming pregnant and who are not using highly effective contraception confirm a negative pregnancy test result prior to starting the intervention. The exact schedule of safety calls is intervention-dependent, and outlined in each ISA.

To investigate the impact of trial interventions on the longer-term effects of COVID-19, we contact participants at three and six months after randomisation to ascertain wellbeing, persistence of symptoms perceived to be related to the index COVID-19 illness, and longer-

For peer review only - http://bmjopen.bmj.com/site/about/guidelines.xhtml

term consequences. Participants' medical record data may additionally be accessed up to twelve months following enrolment to gather follow up data from enrolment to 6 months. Sources of routinely collected data (e.g., NHS Digital) may also be used to follow-up participants for up to 10 years.

Study Outcomes

The primary endpoint is all-cause, non-elective hospitalisation and/or death within 28 days of randomisation, ascertained through patient/Trial Partner report, and/or patient medical records. Secondary endpoints include: time to self-reported recovery defined as the first instance that a participant report feeling fully recovered from the illness; duration of symptoms; symptom recurrence; daily rating of feeling well reported by participants; healthcare service use; participant reported new COVID-19 infections in their household; safety and cost-effectiveness outcomes; symptoms; and, well-being at three and six months (with determination of proportion reporting symptoms perceived to be related to long COVID) from randomisation.

Data Collection and Management

Data are entered into electronic case report forms (CRFs) by the participant, their Trial Partner, or a Hub team member, using Spinnaker. Spinnaker is an online secure, FDA part 11B compliant, data entry system, which is designed to collect sensitive data, such as participant and Trial Partner contact details. All identifiable participant data are encrypted using the Advanced Encryption Standard. Data are stored on a secure cloud hosted server physically located in London, UK. Participant and Trial Partner data will be kept and stored securely for as long as required by the trial and reviewed on annual basis.

For peer review only - http://bmjopen.bmj.com/site/about/guidelines.xhtml

Statistical Methods

Primary endpoint Analysis

Details of the statistical design and methods are described in a Master Statistical Analysis Plan (M-SAP) and Adaptive Design Report (ADR). The primary endpoint analysis is a Bayesian logistic regression model of the primary endpoint comparing a given intervention versus Usual Care, adjusting for age, co-morbidity status, and vaccination status.

The trial design incorporates multiple pre-specified interim analyses that allow each intervention to stop early for futility or superiority. If the Bayesian posterior probability of beneficial treatment effect (alternative hypothesis) is greater than or equal to a pre-specified threshold at an interim or final analysis, the null hypothesis (no beneficial intervention effect) is rejected, and the intervention is deemed superior to Usual Care with respect to Hospitalisation/Death. The decision criteria are defined in the ADR and control the Type I error at the traditional 0.05 two-sided level for each intervention, accounting for multiple interim analyses. As described in the ADR, the pre-specified interim analyses may be bypassed for a given intervention at the discretion of the blinded Trial Management Group (TMG) in the event of a fast accrual rate. Such action requires appropriate modifications to the decision criteria as outlined in the ADR. The ADR also contains extensive simulations to explore the performance of the adaptive design, including power and Type I error. All statistical analyses of primary and some secondary outcome data analysis will be performed by Berry Consultants and the University of Oxford. Berry Consultants is based in the USA; as such they will not receive identifiable trial data.

Sample size

The master protocol specifies a maximum sample of approximately 5300 participants per arm, which provides approximately 90% power for detecting a 33% relative reduction in the

For peer review only - http://bmjopen.bmj.com/site/about/guidelines.xhtml

risk of hospitalisation/death in an experimental arm relative to Usual Care, based on the assumption of an underlying 3% hospitalisation/death rate in the Usual Care arm, and an intervention lowering the hospitalisation/death rate to 2%. However, an intervention-specific appendix may define an alternative maximum sample size for an intervention based on alternative assumptions for a given intervention or based on the relevant state of the pandemic. For example, if the severity of COVID-19 weakens and the aggregate (blinded) primary endpoint event rate is lower than expected, the maximum sample size may be increased to ensure sufficient statistical power.

Primary analysis population

For each intervention, the primary analysis population includes all concurrently randomised patients who were eligible to be randomised to an antiviral agent (concurrent and eligible), who fulfil the eligibility criteria, and who have had the opportunity to complete 28 days of follow-up. Eligible participants will be analysed according to the group they were randomised to regardless of deviation from protocol.

Safety Monitoring

Symptoms, potential medication side-effects and Serious Adverse Events (SAE) are collected from participant daily diaries, calls to participants/Trial Partners, face-to-face visits with Hub clinicians, medical records, notes reviews, and data extracts from hospital and primary care medical records from all UK devolved administrations.

A risk assessed and proportionate approach to safety monitoring is adopted for each antiviral included in the trial. In line with the Summary of Product Characteristics or Investigator Brochure, the risks and the safety profile for each antiviral agent are assessed, and the

For peer review only - http://bmjopen.bmj.com/site/about/guidelines.xhtml

For peer review only

mitigation and monitoring procedures are detailed in the ISA. All safety procedures will be according to University of Oxford Primary Care Clinical Trials Unit pharmacovigilance Standard Operating Procedures.

For each antiviral agent, we only collect Adverse Events (AEs), other than those pre-specified symptoms collected via the participant diaries, if and when specified in the relevant ISA. For certain interventions, pregnancy occurring within 28 days of first intervention administration is recorded as an AE of Special Interest. All-cause hospitalisation and/or death is the primary outcome, and these data are captured in CRFs. Serious adverse events (SAEs) other than hospitalisation or death due to COVID-19 are reported for all antiviral agents over the follow up period. Hospitalisations for pre-existing conditions, including elective procedures planned prior to trial entry, which has not worsened, do not contribute to our primary outcome, and do not constitute SAEs.

A risk assessment and monitoring plan is prepared before opening recruitment to each antiviral agent and is reviewed as necessary over the course of the trial to reflect significant changes to the protocol or outcomes of monitoring activities. Monitoring is performed by the Primary Care Clinical Trials Unit (PC-CTU). The level of monitoring required is informed by the risk assessment.

VIROLOGY SUB-STUDY

A subset of patients from the intervention and comparator arms of the trial are invited to participate in a virology sampled cohort for additional virological testing. The primary aim of

For peer review only - http://bmjopen.bmj.com/site/about/guidelines.xhtml

the virology sampled cohort is to determine whether the antiviral treatment under study reduces viral load to undetectable levels sooner, and to explore the effect of antiviral treatment on development of antiviral resistance. The sub-study primary outcome is SARS-CoV-2 viral load at Day 7. Secondary outcomes include SARS-CoV-2 viral load Days 0-7 and Day 14; SARS-CoV-2 viral genetic whole genome sequence at Day 1, Day 5 (+/- 1 day) and Day 14 (+/- 1 day) and SARS-CoV-2 antibodies at Day 1, Day 5 (+/- 1 day), and Day 14 (+/- 1 day); and to identify any common genetic mutations (occurring in greater than 1% of patients) in patients receiving novel antiviral(s).

The eligibility criteria are as for participants taking part in the main trial, but with an additional exclusion criterion: participants who are within 3 months of receiving a non-trial anti-SARS-CoV-2 antibody therapy are ineligible.

Up to approximately three hundred participants from each trial intervention arm and the Usual Care arm are recruited into the voluntary virology sampled cohort. The first 30 patients enrolling from each trial arm undergo intensive daily viral load monitoring, and are asked to provide daily nasopharyngeal swabs for seven days, and an additional nasopharyngeal swab on Day 14 (+/- 1 day). For participants in intervention arms, the first sample will be taken immediately prior to commencing anti-viral treatment (Day 1). The remaining 270 from each arm in the virology samples cohort have less intensive viral load monitoring, and are asked to provide three nasopharyngeal swabs: one prior to starting treatment, one on Day 5 (+/- 1 day) and one on Day 14 (+/- 1 day).

All participants are asked to take three finger prick dried blood spot samples: one pre-treatment, one on Day 5 (+/- 1 day) and one on Day 14 (+/- 1 day). Participants consenting to

For peer review only

For peer review only - http://bmjopen.bmj.com/site/about/guidelines.xhtml

take part in the virology sampled cohort are sent CE-IVD approved sampling kits for nasopharyngeal sampling, dried blood spot sampling, pre-paid postage and packaging, to post samples to the virology processing site. Samples taken at home should be posted to the trial team within 3 days of sampling, and ideally within 24 hours.

HEALTH ECONOMIC EVALUATION

A prospective economic evaluation is embedded within the trial design to assess the cost-effectiveness of each antiviral from an NHS and Personal Social Services (PSS) perspective. The resource inputs associated with embedding each trial antiviral treatment into routine clinical practice are estimated. Broader resource use is drawn from linked routine health data – encompassing primary care encounters, hospital inpatient/day case admissions, outpatient visits, and accident and emergency attendances. Unit costs are valued using national reference tariffs and attached to resource inputs to generate a compound total NHS and PSS cost per trial participant over the trial time horizon. EQ-5D-5L data are converted using standard algorithms into utility scores for quality-adjusted life year (QALY) estimation. Cost-effectiveness is expressed as incremental cost per QALY gained. [23] Secondary expressions of cost-effectiveness include incremental cost per hospitalisation and/or death prevented over 28 days. Bivariate regression of costs and measures of health consequence, with multiple imputation of missing data, will be conducted to generate within-trial estimates of incremental cost-effectiveness. Sensitivity analyses will assess the impact of areas of uncertainty surrounding components of the economic evaluation. If economic outcomes are non-convergent within the trial follow-up period, then extrapolation of cost-effectiveness through decision-analytic modelling will be considered, drawing upon the best available information from the literature to supplement the trial data. Specific plans for the economic evaluation are outlined in a pre-specified health economics analysis plan.

For peer review only - http://bmjopen.bmj.com/site/about/guidelines.xhtml

ETHICS, APPROVALS, MONITORING AND DISSEMINATION:

The trial has been approved by the University of Oxford Research Governance Ethics and Assurance Team as study sponsor, the South Central–Berkshire Research Ethics Committee (REC number: 21/SC/0393) of the Health Research Authority (HRA), and the UK Medicines and Healthcare products Regulatory Agency (MHRA). All participants provide informed consent, online or by telephone, before participation. All participants completing the 28 day follow up are provided with a £10 voucher in recognition of their contribution to the study. The University of Oxford as sponsor has a specialist insurance policy in place, which would operate in the event of any participant suffering harm as a result of their involvement in the research (Newline Underwriting Management Ltd, at Lloyd's of London). NHS indemnity operates in respect of the clinical treatment that is provided.

An independent Data Monitoring and Safety Committee (DMSC) reviews emerging data provided by the Statistical Analysis Committee (SAC) and communicates key decisions to the Trial Steering Committee (TSC), which in turn advises the Trial Management Group (TMG) and also provides trial oversight.

It is expected that trial results will be published in peer-reviewed journals and relevant findings presented at national and international conferences.

Trial Status

PANORAMIC was registered on the ISRCTN registry (ISRCTN 30448031) on 3rd November 2021. Enrolment started on 8th December 2021. By 17 September 2022, 26,285 participants have been recruited. Protocol v.5.0, 09 May 2022 (see additional file 2).

For peer review only - http://bmjopen.bmj.com/site/about/guidelines.xhtml

DISCUSSION

Summary

Despite high uptake of vaccination against COVID-19 in many countries, the disease remains prevalent, with many patients continuing to experience considerable morbidity and require treatment in hospital. We describe a platform randomised trial to evaluate antiviral therapeutic agents for use by people at higher risk from COVID-19 in the community with confirmed acute, symptomatic SARS-CoV-2 infection.

Comparison with other studies of novel antiviral agents for community treatment of COVID-19

A phase 3 placebo-controlled, randomised trial of molnupiravir recruited 1,433 COVID-19 outpatients in over 20 countries, with a primary efficacy endpoint of all-cause hospitalisation or death within 29 days of enrolment (MOVe-OUT trial). [24] The authors found that treatment with molnupiravir reduced the risk of hospitalisation or death compared with placebo (risk difference, −3.0 %; 95% CI: −5.9 % to −0.1%). [24] Adverse events occurred with similar frequency in molnupiravir and placebo groups (30.4 % and 33.0 %, respectively), as did adverse events deemed to be related to the trial regimen (8.0 % and 8.4%, respectively). No deaths were attributed to the trial treatment (one death in the molnupiravir group and nine deaths in the placebo group).

As in the PANORAMIC trial, participants were at higher risk of an adverse illness course, received a five-day course of molnupiravir at a dose of 800 mg twice daily, and received the intervention within five days of symptom onset. However, the trial recruited unvaccinated patients; the vast majority of the UK adult population are multiply-vaccinated (primary

For peer review only - http://bmjopen.bmj.com/site/about/guidelines.xhtml

course plus boosters).[25] Furthermore, Delta, Gamma and Mu variants accounted for the majority of SARS-CoV-2 variants in the MOVe-OUT trial[26], whereas the predominant variant in circulation in the UK has been Omicron since December 2021.[27] PANORAMIC additionally incorporates an assessment of the impact of antiviral drugs on viral load and markers of viral resistance.

In a phase 2-3 randomised, placebo-controlled trial of 2,246 outpatients with COVID-19 from the United States (41%), Europe (30%), South America (12.3%), Asia (14%) and Africa (0.6%), at higher risk of an adverse illness course, treatment with nirmatrelvir/ritonavir resulted in a 5.8% absolute risk reduction in the primary outcome of COVID-19 related hospitalisation and all-cause death within 28 days (0.72% and 6.53% respectively, risk difference -5.81%, 95% CI: -7.78 % to -3.84%, p<0.0001).[13] Viral load was significantly reduced by treatment with nirmatrelvir/ritonavir (adjusted mean difference of −0.868 log10 copies per millilitre, 95% CI: -1.074 to -0.6615, p<0.001). The incidence of adverse events was similar in both groups, and all thirteen deaths occurred in the placebo group. The trial population was again unvaccinated, and therefore distinct from the UK population taking part in the PANORAMIC trial.

Strengths and Limitations

The platform design, informed by the experience of the PRINCIPLE trial,[28] allows PANORAMIC to add new interventions to the trial as they become available; this increases the efficiency of the trial as multiple interventions can be assessed by a single trial platform without having to set up a new trial each time a new intervention for this condition requires evaluation. Pre-specified interim analyses allow randomisations to interventions to be stopped as soon as pre-specified criteria for superiority or futility are met, potentially

For peer review only - http://bmjopen.bmj.com/site/about/guidelines.xhtml

reducing time to trial conclusions. This ensures the trial's relevance in the face of rapidly evolving pandemic circumstances.

Deploying antimicrobials of any kind at scale raises the question of their possible impact on antimicrobial resistance. A virology sub-study has been incorporated in PANORAMIC, which allows us to estimate virological endpoints, as well as facilitating careful evaluation of potential harms associated with antiviral treatment, such as the development of antiviral resistance and emergence of new variants.

Cost effectiveness of novel antivirals is as yet unknown, but is critically important to considerations of widespread deployment of expensive: PANORAMIC aims to fill this gap in the evidence base for these agents.

Traditionally, primary care research implementation has followed a similar model to hospital-based studies, in which the "participant comes to the research." In this approach, potential participants are invited to participate if they receive their health care or live in the proximity to the research site. The capacity of PANORAMIC for recruitment of eligible people from almost anywhere in the UK, not limited by where people live or receive their health care, allows the "research to be taken to the patient." This is particularly important, given that participants are ill and probably highly infectious.

The trial has been designed to be minimally burdensome for participants; all trial procedures are possible remotely, from registration, to eligibility checks, to receiving trial medications and virology sub-study materials by courier. This has facilitated rapid recruitment to the trial, with over 26,000 participants recruited to date. PANORAMIC strives to be a truly

For peer review only - http://bmjopen.bmj.com/site/about/guidelines.xhtml

representative trial, with participants from various backgrounds recruited nationally from all four UK nations. A proactive outreach strategy has been employed, led by the trial's national pharmacy, and inclusion and diversity lead, with the support of UK-wide pharmacy networks, to help to promote the trial to diverse communities and to those disproportionately affected by COVID-19. This includes people from ethnic minority backgrounds and those living in areas of higher deprivation, traditionally known to be under-represented in clinical trials.

In addition to the primary outcome that is measured at 28 days, PANORAMIC evaluates longer-term outcomes at three and six months, which will help ascertain the effect of antiviral treatment on long COVID. Long COVID, defined as symptoms beyond four weeks after index illness [29] may affect between 10% [30] and 43.4% [31] of patients with COVID-19, and is characterised by a range of physical and psychological symptoms. [29] Thus far, we do not know whether novel antiviral treatments reduce symptoms associated with the acute illness over the longer term.

Some may consider the open-label design of the trial a weakness. The lack of blinding means that we cannot estimate the proportion of any positive effect from the treatment that results from a possible placebo effect. However, the objective primary outcome in PANORAMIC (non-elective hospitalisation and/or death) is unlikely to be affected by a placebo effect, as hospital admission is a clinical decision, and the virology sub-study will also provide a helpful pointer as to whether the treatments are effective. Furthermore, comparison with usual care is in keeping with pragmatic trial design and more closely reflective of real-world practice. [32] As placebos are not used in clinical care, the results of an open-label trial are more likely to reflect what would happen if the intervention were introduced into routine clinical practice, [32] additionally enabling a more realistic assessment of cost effectiveness.

For peer review only - http://bmjopen.bmj.com/site/about/guidelines.xhtml

For peer review only

Findings from the pragmatic, open label PRINCIPLE trial have found no difference in outcome measures that rely on participants' self-reported recovery between participants allocated to usual care and usual care plus a study drug. [5 28 33]

For peer review only

For peer review only - http://bmjopen.bmj.com/site/about/guidelines.xhtml

Acknowledgements

The authors would like to especially thank all participants in the study and acknowledge the work and support of all participating general practices, NHS COVID-19 treatment services and other health and social care organisations supporting the trial. We would also like to thank our PPI contributors, the Trial Steering Committee, the Data Monitoring and Safety Committee, primary care and other colleagues in the National Institute of Health and Care Research Clinical Research Network, and the National Institute of Health and Care Research. CCB acknowledges part support as Senior Investigator of the National Institute of Health and Care Research, the NIHR Community Healthcare Medtech and In-Vitro Diagnostics Co-operative (MIC), and the NIHR Health Protection Research Unit on Health Care Associated Infections and Antimicrobial Resistance. FDRH acknowledges his part-funding from the NIHR Applied Research Collaboration (ARC OxTV) and the NIHR Community Healthcare Medtech and In-Vitro Diagnostics Co-operative (MIC). GH is funded by and NIHR Advanced Fellowship and by the NIHR Community Healthcare Medtech and In-Vitro Diagnostics Co-operative (MIC). JD is funded by the Wellcome Trust PhD Programme for Primary Care Clinicians (216421/Z/19/Z). SP receives support as a NIHR Senior Investigator (NF-SI-0616-10103) and from the UK NIHR Applied Research Collaboration Oxford and Thames Valley. OVH is supported by an NIHR Development and Skills Enhancement Award. KH receives support as a HCRW Senior Research Leader and the Centre for Trials Research receives infrastructure funding from Health & Care Research Wales and Cancer Research UK. OAG is funded by ECRAID-Prime (grant number 101046109).

Data Monitoring and Safety Committee Independent members:

Prof Deborah Ashby (Chair)

For peer review only - http://bmjopen.bmj.com/site/about/guidelines.xhtml

Prof Benjamin Fisher

Prof Simon Gates

Prof Gordon Taylor

Prof Martin Underwood

Trial Steering Committee Independent members

Philip Hannaford (Chair)

Ms Corina Cheeks

Prof Ranjit Lall

Prof Alastair Hay

Prof William Hollingworth

Prof Matthew Sydes: Independent observer

Prof Mike Moore: Independent observer

Authors' contributions

CCB and JSN-V-T conceived the study. CCB is the Chief Investigator. PL, FDRH are co-Chief Investigators. CCB, PL, and FDRH decided to publish the paper. BRS, L-MY, JH, MD, CCB, FDRH, PL, GH, OAG, JD, NMR, DBR, SP, DML, JFS, KH, PE, OVH and ML provided input to the trial design. EO, JA, PE, LL, EH, LC, MB, MC, SB, CB, JCD, AC-S and IR-W are responsible for study implementation and acquisition of data. CCB, OAG, GH, FDRH, JH, L-MY, JD, JM, BRS, EO, JA, MGP, SP PL, KH, NMR, JFS and SP drafted the manuscript. HR leads the clinical team. L-MY, BRS, JH, VH and JM contribute to statistical analysis. SK, DBR, NMR and MD provide input to safety evaluations, monitoring, and drug interactions. MGP is the National Pharmacy, and Inclusion and Diversity Lead for the trial. SP and MEP run the economic evaluation. JFS, DML and JB lead the virology sub-study. JC

For peer review only - http://bmjopen.bmj.com/site/about/guidelines.xhtml

leads on the information systems. MB leads data management. CCB, PL, OAG, NMR, SP, DBR, KH, MGP, BRS, EO, JD, DML, SK, NF, NPBT, PE, JFS, JB, JA, MD, T-AM, MEP, GH, ML, BJ, NDH, JC, EH, LC, MB, MA, OvH, AU, MK, L-MY and FDRH are members of the Trial Management Group supporting site recruitment, activity and delivery. OAG and CCB produced the first draft of the manuscript. All authors critically revised the manuscript. All authors are contributing to the conduct of the trial.

Funding statement:

This work is supported by the UKRI/NIHR, Grant number NIHR135366. Virology team members are partly supported by an MRC COVID modelling grant, Grant number MR/W015560/1. JD is funded by the Wellcome Trust PhD Programme for Primary Care Clinicians (216421/Z/19/Z). For the purpose of Open Access, the author has applied a CC BY public copyright license to any Author Accepted Manuscript version arising from this submission. NDH was funded by the Northern Ireland Clinical Research Network, Public Health Agency. OAG is funded by ECRAID-Prime (grant number 101046109).

Competing interests:

JSN-V-T was seconded to the Department of Health and Social Care, England (DHSC) from October 2017 to March 2022. The views expressed in this paper are those of its authors and not necessarily those of DHSC. JSN-V-T reports a lecture fee from Gilead Sciences Ltd (manufacturer of remdesivir) and a paid Influenza Advisory Board for F. Hoffmann-La Roche (manufacturer of tocilizumab), both after March 2022. KH is a member of the following NIHR committees: HTA General Committee, HTA Funding Strategy Group, Research Professors Funding Committee. KH is co-investigator on the grant provided by UKRI/NIHR, Grant number NIHR135366 (subcontract from University of Oxford to Cardiff University). KH received a grant from AstraZeneca to support a trial of Evusheld for the

For peer review only - http://bmjopen.bmj.com/site/about/guidelines.xhtml

prevention of COVID in high risk individuals (to Cardiff University). KH is an independent member of the IDMC for the OCTAVE-DUO trial of vaccines for COVID in high risk individuals (unpaid). DML has received grants/contracts from LifeArc, Medical Research Council, Bristol Myers Squibb and Blood Cancer UK. DML received personal fees/honoraria for a lecture from Biotest UK, for an educational video from Gilead and for a 'round table' discussion with Merck. SP is co-investigator on the grant provided by UKRI/NIHR, Grant number NIHR135366. DBR has received consulting fees from OMASS therapeutics. DBR has a leadership/fiduciary role in the Heal-COVID trial TMG. BRS reports grant money paid to his employer (Berry Consultants, LLC) from The University of Oxford, from the Sponsor's grant from the UKRI/NIHR, per the statistical design and analyses for the PANORAMIC trial. GH reports that the NIHR funded this study. ML reports funding directly from the PANORAMIC trial (NIHR). ML reports being a RAPIS-TEST (NIHR EME) DMC member. JM reports that this is part of his consulting work for Berry Consultants. SK reports research funding from GSK, ViiV Healthcare, Ridgeback Biotherapeutics, Vir and Merck unrelated to this work. SK reports speaker's fees from ViiV Healthcare and participation on ViiV Healthcare, Pfizer advisory boards. SK reports receiving donation of drugs for clinical studies from ViiV Healthcare, Toyama and GSK. JFS reports receiving research grants from MRC (MR/X004724/1), Wellcome, NIHR, DNDi, Gates and MRC (MR/W015560/1) paid to his institution. JFS reports receiving Pharmacometric consultancy fees from Adrenomed Ltd paid to his institution. JFS reports participation on a Data Safety Monitoring board/Advisory Board for GSK Sotrovimab paediatric programme. MA reports receiving grants from BTRU – GEMS, Janssen – Cartography , Pfizer – Myst, Prenetics, Dunhill Medical Trust, BMA Trust – Kathleen Harper Fund and Antibiotic Research UK – all paid to the institution. MA reports receiving consultancy fees from Prenetics and OxDx. MA reports a planned patent for Ramanomics. MA reports participation on a Data Safety Monitoring board/Advisory Board

For peer review only - http://bmjopen.bmj.com/site/about/guidelines.xhtml

for Prenetics. MA has an unpaid leadership/fiduciary role in the E3 Initiative. NPBT reports his current affiliations with RCGP and NIHR TVSM. NPBT reports a payment for a single episode of participation on the MSD advisory board in July 2021, prior to any knowledge or planning of this trial. OVH reports receiving an NIHR Development and Skills Personal Award. OVH reports receiving consulting fees for MINDGAP BV, with the fees paid to Oxford University lnnovation Limited. OVH reports unpaid participation on a Data Safety Monitoring board/Advisory Board for The CHIldren with COugh Cluster Randomised Controlled Trial (CHICO). OVH has an unpaid leadership/fiduciary role in the British Society of Antimicrobial Chemotherapy. CB reports full employment with the Nuffield Department of Primary Care Health Sciences. AU reports receiving consulting fees and payment/honoraria from Merck/MSD and Gilead Sciences. FDRH reports receiving expenses reimbursed for talks on COVID monoclonal antibodies. PL reports support from the NIHR for the grant to do the PANORAMIC study. NF reports receiving consulting fees from Abbott Diagnostics and GSK, a presentation fee from Abbott Diagnostics, and has stocks in Synairgen PLC.

For peer review only

For peer review only - http://bmjopen.bmj.com/site/about/guidelines.xhtml

Word Count: 5393 words

References

1. Bergwerk M, Gonen T, Lustig Y, et al. Covid-19 breakthrough infections in vaccinated health care workers. *New England Journal of Medicine* 2021;385(16):1474-84.
2. Paris C, Perrin S, Hamonic S, et al. Effectiveness of mRNA-BNT162b2, mRNA-1273, and ChAdOx1 nCoV-19 vaccines against COVID-19 in healthcare workers: an observational study using surveillance data. *Clinical Microbiology and Infection* 2021;27(11):1699. e5-99. e8.
3. Pilishvili T, Gierke R, Fleming-Dutra KE, et al. Effectiveness of mRNA Covid-19 vaccine among US health care personnel. *New England Journal of Medicine* 2021;385(25):e90.
4. The Telegraph. Covid hospital admissions are surging again – is this the start of a new wave? 2022 [Available from: https://www.telegraph.co.uk/global-health/science-and-disease/covid-hospital-admissions-surging-start-new-wave/ accessed 10th May 2022.
5. Butler CC, Yu LM, Dorward J, et al. Doxycycline for community treatment of suspected COVID-19 in people at high risk of adverse outcomes in the UK (PRINCIPLE): a randomised, controlled, open-label, adaptive platform trial. *Lancet Respir Med* 2021;9(9):1010-20. doi: 10.1016/S2213-2600(21)00310-6 [published Online First: 20210727]
6. Principle Trial Collaborative Group. Azithromycin for community treatment of suspected COVID-19 in people at increased risk of an adverse clinical course in the UK (PRINCIPLE): a randomised, controlled, open-label, adaptive platform trial. *Lancet* 2021;397(10279):1063-74. doi: 10.1016/S0140-6736(21)00461-X [published Online First: 2021/03/08]
7. Yu L-M, Bafadhel M, Dorward J, et al. Inhaled budesonide for COVID-19 in people at higher risk of adverse outcomes in the community: interim analyses from the PRINCIPLE trial. *Medrxiv* 2021
8. RECOVERY Collaborative Group. Dexamethasone in hospitalized patients with Covid-19. *New England Journal of Medicine* 2021;384(8):693-704.
9. Malone B, Campbell EA. Molnupiravir: Coding for catastrophe. *Nature Structural & Molecular Biology* 2021;28(9):706-08.
10. Pfizer. Pfizer's Novel COVID-19 Oral Antiviral Treatment Candidate Reduced Risk of Hospitalization or Death by 89% in Interim Analysis of Phase 2/3 EPIC-HR Study 2021 [Available from: https://www.pfizer.com/news/press-release/press-release-detail/pfizers-novel-covid-19-oral-antiviral-treatment-candidate accessed 10th May 2022.
11. Extance A. Covid-19: What is the evidence for the antiviral Paxlovid? *bmj* 2022;377
12. Singh AK, Singh A, Singh R, et al. Molnupiravir in COVID-19: a systematic review of literature. *Diabetes & Metabolic Syndrome: Clinical Research & Reviews* 2021;15(6):102329.
13. Hammond J, Leister-Tebbe H, Gardner A, et al. Oral nirmatrelvir for high-risk, nonhospitalized adults with COVID-19. *New England Journal of Medicine* 2022;386(15):1397-408.
14. Berry SM, Connor JT, Lewis RJ. The platform trial: an efficient strategy for evaluating multiple treatments. *Jama* 2015;313(16):1619-20.
15. Butler CC, Connor JT, Lewis RJ, et al. Answering patient-centred questions efficiently: response-adaptive platform trials in primary care. *British Journal of General Practice* 2018;68(671):294-95.
16. Woodcock J, LaVange LM. Master protocols to study multiple therapies, multiple diseases, or both. *New England Journal of Medicine* 2017;377(1):62-70.
17. University of Oxford Primary Care Clinical Trials Unit. PANORAMIC trial 2021 [Available from: https://www.panoramictrial.org/ accessed 16th March 2022.
18. UK Health Security Agency. People with symptoms of a respiratory infection including COVID-19 2022 [Available from: https://www.gov.uk/guidance/people-with-symptoms-of-a-

For peer review only - http://bmjopen.bmj.com/site/about/guidelines.xhtml

respiratory-infection-including-covid-19#what-to-do-if-you-have-a-positive-covid-19-test-result accessed 10th May 2022.
19. Herdman M, Gudex C, Lloyd A, et al. Development and preliminary testing of the new five-level version of EQ-5D (EQ-5D-5L). *Quality of life research* 2011;20(10):1727-36.
20. Moustgaard H, Clayton GL, Jones HE, et al. Impact of blinding on estimated treatment effects in randomised clinical trials: meta-epidemiological study. *bmj* 2020;368
21. Anand R, Norrie J, Bradley JM, et al. Fool's gold? Why blinded trials are not always best. *Bmj* 2020;368
22. National Institute for Health and Care Excellence. COVID-19 rapid guideline: managing COVID-19 [NG191] 2021 [updated 13th April 2022. Available from: https://www.nice.org.uk/guidance/ng191 accessed 30th April 2022.
23. Van Hout B, Janssen M, Feng Y-S, et al. Interim scoring for the EQ-5D-5L: mapping the EQ-5D-5L to EQ-5D-3L value sets. *Value in health* 2012;15(5):708-15.
24. Jayk Bernal A, Gomes da Silva MM, Musungaie DB, et al. Molnupiravir for oral treatment of Covid-19 in nonhospitalized patients. *New England Journal of Medicine* 2022;386(6):509-20.
25. GOV.UK. Vaccinations in the United Kingdom 2022 [Available from: https://coronavirus.data.gov.uk/details/vaccinations accessed 10th May 2022.
26. Merck. Merck and Ridgeback's Investigational Oral Antiviral Molnupiravir Reduced the Risk of Hospitalization or Death by Approximately 50 Percent Compared to Placebo for Patients with Mild or Moderate COVID-19 in Positive Interim Analysis of Phase 3 Study 2021 [Available from: https://www.merck.com/news/merck-and-ridgebacks-investigational-oral-antiviral-molnupiravir-reduced-the-risk-of-hospitalization-or-death-by-approximately-50-percent-compared-to-placebo-for-patients-with-mild-or-moderat/ accessed 4th May 2022.
27. Burki TK. Omicron variant and booster COVID-19 vaccines. *The Lancet Respiratory Medicine* 2022;10(2):e17.
28. Hayward G, Butler CC, Yu LM, et al. Platform Randomised trial of INterventions against COVID-19 In older peoPLE (PRINCIPLE): protocol for a randomised, controlled, open-label, adaptive platform, trial of community treatment of COVID-19 syndromic illness in people at higher risk. *BMJ Open* 2021;11(6):e046799. doi: 10.1136/bmjopen-2020-046799 [published Online First: 20210618]
29. National Institute for Health and Care Excellence. COVID-19 rapid guideline: managing the long-term effects of COVID-19 2020 [updated 11 November 2021. Available from: https://www.nice.org.uk/guidance/NG188 accessed 10th October 2022.
30. Greenhalgh T, Knight M, Buxton M, et al. Management of post-acute covid-19 in primary care. *bmj* 2020;370
31. Cirulli E, Barrett KMS, Riffle S, et al. Long-term COVID-19 symptoms in a large unselected population. *medrxiv* 2020
32. MacPherson H. Pragmatic clinical trials. *Complementary therapies in medicine* 2004;12(2-3):136-40.
33. Dorward J, Yu LM, Hayward G, et al. Colchicine for COVID-19 in the community (PRINCIPLE): a randomised, controlled, adaptive platform trial. *Br J Gen Pract* 2022;72(720):e446-e55. doi: 10.3399/BJGP.2022.0083 [published Online First: 20220630]

For peer review only - http://bmjopen.bmj.com/site/about/guidelines.xhtml

NUFFIELD DEPARTMENT OF **PRIMARY CARE** HEALTH SCIENCES

Primary Care Clinical Trials Unit

PANORAMIC Platform Adaptive trial of NOvel antiviRals for eArly treatMent of COVID-19 In the Community

Platform Adaptive trial of NOvel antiviRals for eArly treatMent of covid-19 In the Community: PANORAMIC

REC Number: 21/SC/0393 **IRAS Number: 1004274**

Chief Investigator: Professor Christopher Butler **Participant ID:** ______________________

CONSENT FORM

Thank you for completing the screening questionnaire, you have passed screening for the trial.

Please read the Participant Information Sheet (PIS) if you haven't already done so, and if you are willing to participate please select 'Yes', TYPE your FIRST and LAST names below and then click Submit

If you agree, please select 'Yes' to confirm that you have read and understood the following:

		**YES**	**NO**
1	I confirm I have read and understood the information sheet version number ____-________dated __ __ / __ __ __/ __ __ __ __ for the above study. I have had the opportunity to ask questions and had these answered satisfactorily.		
2	I understand my participation is voluntary and that I am free to withdraw at any time, without giving any reason, and without my medical care or legal rights being affected.		
3	I understand that if I chose to withdraw data already collected will continue to be used and I or my GP may be contacted if there are further questions regarding side-effects from trial treatments.		
4	I understand that I will be randomised to receive either: standard care plus an antiviral treatment or standard care, and that I will not be able to choose which I will receive.		
5	I understand that relevant sections of my GP and hospital medical notes and data collected during the study may be looked at by members of the research team and individuals from University of Oxford, both during and for up to 10 years after the scheduled follow-up period. It may also be reviewed by relevant people from regulatory authorities and from NHS Organisation(s). I give permission for these individuals to have access to my records which identify me by name.		
6	I understand that my personal information may be shared with University of Dundee's Health Informatics Centre and that my date of birth and/or my NHS number (or equivalent UK NHS Identifier) will be shared with NHS Digital**,** electronic Data Research and Innovation Service (eDRIS), The Secure Anonymised Information Linkage (SAIL) Databank or Health and Social Care Northern Ireland (HSC Business Services Organisation/HSC Trusts) (HSC NI) to enable them to supply the study team with additional healthcare data about me, which is relevant to the trial. The data supplied by NHS Digital, eDRIS, SAIL or HSC NI is linked by the research team to the data collected during my participation in the trial. I am free to withdraw my consent for data linkage with NHS Digital, eDRIS, SAIL or HSC NI at any time and it will not affect my ongoing care.		
7	I understand that members of the research team may view my general practice and hospital medical records, including the summaries of my medical records (e.g. Summary Care Record (SCR), Emergency Care Summary (ECS), The GP Summary, Northern Ireland		

For peer review only

For peer review only - http://bmjopen.bmj.com/site/about/guidelines.xhtml

	Electronic Care Record, and the Welsh Clinical Portal to check my medication, allergies, adverse reactions, and additional information to make sure that it is safe for me to take trial medication. I give permission for these individuals to access my medical records for this purpose.		
8	I consent to being contacted by the research team for the purposes of trial follow up (up to 6 months) and I understand that this will require me to provide my contact details to the research team.		
9	I consent to my GP and/or Care Home being informed of my participation within the study, and I understand that the trial team may contact my GP about my ongoing participation in the trial.		
10	I understand that the information collected about me may be shared in a form that cannot identify me with commercial companies to support the licensing of trial treatments, within the UK and abroad.		
11	I agree to take part in the trial.		
	For participants capable of being pregnant (regardless of current contraception methods) *(to show only for those who meet this criterion in the screening form)*		
12	I agree to taking a pregnancy test prior to taking the trial treatment.		
13	I understand that I must use reliable methods of contraception (as specified in the PIS appendices). I agree to provide information requested on any pregnancy, including pregnancy outcome, occurring within 28-days following first administration of the IMP, as requested by the MHRA. I understand that if I report a pregnancy the Sponsor will report this to The UK Teratology Information Service (UKTIS).		
	ADDITIONAL (optional, not required for study participation)	**YES**	**NO**
14	I agree to provide the research team with the contact details of my Trial Partner. I confirm my Trial partner is aware of their role and willing to answer questions.		
15	I agree to take part in the Virology Sampled Cohort.		
	For Participants Agreeing to take part in Virology Study		
16	I agree to donate blood and nasopharyngeal samples. I consider these samples a gift to the University of Oxford, and I understand I will not gain any direct personal or financial benefit from them. I understand that even if I withdraw from the above study, the samples collected from me may still be used in the study analysis.		

If you are the participant completing the consent form, please provide your signature below

Participant Signature: ______________________________

For peer review only - http://bmjopen.bmj.com/site/about/guidelines.xhtml

NUFFIELD DEPARTMENT OF PRIMARY CARE HEALTH SCIENCES
Primary Care Clinical Trials Unit

PANORAMIC
Platform Adaptive trial of NOvel antiviRals for eArly treatMent of COVID-19 In the Community

First Name: ____________________ ____________________
Last Name:

Date: __ __ / __ __ / __ __ __ __

If the participant has provided verbal consent, but they are unable to complete the consent form due to lack of online access, too unwell, too frail or consent is completed via the telephone (the participant must have capacity), please provide:

1. **Name of the participant:**

First Name: ____________________ **Last Name:** ____________________

Date: __ __ / __ __ / __ __ __ __

2. **Signature of person completing the form:**

First Name: ____________________ **Last Name:** ____________________

Role: Trial partner/trial team member/Health Care Professional

Date: __ __ / __ __ / __ __ __ __

If participant lacks capacity to give consent:

I have read the information (or had it read to me), had an opportunity to ask questions and signed the *Legal Representative Letter*. I understand that the patient will be asked to confirm their consent as soon as they have the capacity to do so and that if they wish, they will be able to withdraw from the trial without it affecting their medical care.

Participant:

Name: ____________________ Date: __ __ / __ __ / __ __ __ __

I believe that if they were able to, the patient would wish to take part in this trial.

____________________ ____________________

PRINTED name of Legal Representative **Signature of Legal Representative**

Today's date__ __ / __ __ / __ __ __ __

For peer review only - http://bmjopen.bmj.com/site/about/guidelines.xhtml

NUFFIELD DEPARTMENT OF PRIMARY CARE HEALTH SCIENCES
Primary Care Clinical Trials Unit
PANORAMIC Platform Adaptive trial of NOvel antiviRals for eArly treatMent of COVID-19 In the Community

Relationship to participant (as confirmed in the signed *Legal Representative Letter*)

____

You will have the opportunity to print a copy of the consent form after submission. Please contact the study team if you would like a copy sent to you.

By submitting, I confirm that I am the person whose name is stated above.

If you have any questions about consent or the trial, please contact the study team:

Tel: 08081 560017 Email panoramic@phc.ox.ac.uk

For peer review only

For peer review only - http://bmjopen.bmj.com/site/about/guidelines.xhtml

NUFFIELD DEPARTMENT OF PRIMARY CARE HEALTH SCIENCES
Primary Care Clinical Trials Unit

PANORAMIC
Platform Adaptive trial of NOvel antiviRals for eArly treatMent of COVID-19 In the Community

Trial Title: **P**latform **A**daptive trial of **NO**vel antivi**R**als for e**A**rly treat**M**ent of covid-19 **I**n the **C**ommunity

Internal Reference Number / Short title: PANORAMIC
Ethics Ref: 21/SC/0393
IRAS Project ID: 1004274
EudraCT Number: 2021-005748-31
Date and Version No: 9 May 2022 Version 5.0

**Chief Investigator and trial leader:**	Professor Christopher Butler, Department of Primary Care Health Sciences University of Oxford
**Co-Principal Investigator and Co-trial lead:**	Professor Paul Little, School of Primary Care, Population Sciences and Medical Education, University of Southampton
**Co-Principal Investigator and Co-trial lead:**	Professor Richard Hobbs, Department of Primary Care Health Sciences University of Oxford
**Investigators:**	Dr Ly-Mee Yu, Primary Care Clinical Trials Unit, Department of Primary Care Health Sciences, University of Oxford
	Dr Emma Ogburn, Primary Care Clinical Trials Unit, Department of Primary Care Health Sciences, University of Oxford
	Dr Gail Hayward, Department of Primary Care Health Sciences, University of Oxford
	Dr Hannah Swayze, Primary Care Clinical Trials Unit, Department of Primary Care Health Sciences, University of Oxford
	Ms Julie Allen, Primary Care Clinical Trials Unit, Department of Primary Care Health Sciences, University of Oxford
	Dr Jienchi Dorward, Department of Primary Care Health Sciences, University of Oxford
	Dr Oliver van Hecke, Department of Primary Care Health Sciences, University of Oxford
	Dr Kome Gbinigie, Department of Primary Care Health Sciences, University of Oxford
	Professor Stavros Petrou, Department of Primary Care Health Sciences, University of Oxford

For peer review only

For peer review only - http://bmjopen.bmj.com/site/about/guidelines.xhtml
© Copyright: The University of Oxford and Oxford University Hospitals NHS Foundation Trust 2019

Professor Najib Rahman, Oxford Respiratory Trials Unit, Nuffield Department of Medicine of the University of Oxford

Ms Melissa Dobson, Oxford Respiratory Trials Unit, Nuffield Department of Medicine of the University of Oxford

Professor Duncan Richards, Oxford Clinical Trials Research Unit, Nuffield Department of Orthopaedics, Rheumatology and Musculoskeletal Sciences, University of Oxford

Dr Monique Anderson, Oxford University Hospitals NHS Foundation Trust, University of Oxford

Dr Ben Saville, Berry Consultants, Texas, USA, & Department of Biostatistics, Vanderbilt University School of Medicine, Tennessee, USA

Professor Saye Khoo, Department of Pharmacology, University of Liverpool

Professor Kerry Hood, Centre for Trials Research, Cardiff University

Dr Tracie-Ann Madden, Centre for Trials Research, Cardiff University

Professor Andrew Ustianowski, Infectious Disease and Tropical Medicine, Manchester University NHS Foundation Trust

Dr Bhautesh Jani, General Practice & Primary Care, University of Glasgow

Professor Joseph Standing, Infection, Immunity & Inflammation, University College London

Professor Judith Breuer, Infection, Immunity & Inflammation, University College London

Dr David Lowe, Infection, Immunity & Inflammation, University College London

Professor Tim McHugh, Infection, Immunity & Inflammation, University College London

Professor Mahendra G Patel, Primary Care Clinical Trials Unit, Department of Primary Care Health Sciences, University of Oxford
Dr Mark Lown, Department of Medicine, University of Southampton

Professor Nick Francis, School of Primary Care, Population Sciences and Medical Education, University of Southampton

Professor Nigel Hart, School of Medicine, Dentistry and Biomedical Sciences, Queens University Belfast

For peer review only

For peer review only - http://bmjopen.bmj.com/site/about/guidelines.xhtml
© Copyright: The University of Oxford and Oxford University Hospitals NHS Foundation Trust 2019

Dr Nicholas PB Thomas, National Institute for Health Research (NIHR) Clinical Research Network, National Institute for Health Research, London, UK and Royal College of General Practitioners, London, UK

Sponsor: University of Oxford
Joint Research Office
1st floor, Boundary Brook House
Churchill Drive, Headington, OX3 7GB

Funder: Department of Health and Social Care (DHSC), National Institute of Health Research (NIHR)

Chief Investigator Signature (Professor Christopher Butler):

Lead Trial Statistician Signature (Dr Ly-Mee Yu):

No potential conflict of interest

Confidentiality Statement
This document contains confidential information that must not be disclosed to anyone other than the Sponsor, the Investigator Team, HRA, host organisation, and members of the Research Ethics Committee and Regulatory Authorities unless authorised to do so.

The term '**central clinical team'** refers to a team of medically qualified professionals and research nurses located at the PC-CTU and ORTU.

The term '**central trial team'** refers to the team responsible for the day-to-day conduct of the trial, which includes the central clinical team, as well as other non-clinical trial staff.

PC-CTU SOPs will be used for all aspects of PANORAMIC.

See *supplementary material B* for **Key Trial Contacts**.

peer review only

For peer review only - http://bmjopen.bmj.com/site/about/guidelines.xhtml
© Copyright: The University of Oxford and Oxford University Hospitals NHS Foundation Trust 2019

NUFFIELD DEPARTMENT OF PRIMARY CARE HEALTH SCIENCES Primary Care Clinical Trials Unit

PANORAMIC Platform Adaptive trial of NOvel antiviRals for eArly treatMent of COVID-19 In the Community

Platform Adaptive trial of NOvel antiviRals for eArly treatMent of covid-19 In the Community (PANORAMIC): Overview

Background: Despite high uptake of vaccination against COVID-19, the disease remains prevalent in the UK and in many countries around the world, with many patients continuing to experience considerable morbidity and require treatment in hospital. There is therefore an urgent need to identify treatments for COVID-19 for use in the community early on in the illness that speeds recovery and prevents the need for hospital admission.

Aims and objectives:

Main trial: This protocol describes a platform randomised trial of antiviral therapeutic agents for use by clinically vulnerable people in the community with confirmed acute symptomatic SARS-CoV-2 infection.
Virology sampled cohort: The primary aim of the virology sampled cohort is to determine whether antiviral treatment in the community reduces viral load to undetectable levels more quickly than untreated patients and to explore antiviral treatment on potential development of antiviral resistance.

Platform trial: A "platform trial" is a trial in which multiple treatments for the same disease can be tested simultaneously, and in which new interventions can be added or replace existing ones during the course of the trial in accordance with pre-specified criteria.

Interventions: Participants will be randomised to receive either Usual Care (see Usual Care Intervention Specific Appendix (ISA), or an antiviral agent in addition to Usual Care (see ISA for each antiviral agent under trial). Potential participants can be included if they are eligible to be randomised to at least one novel antiviral agent, as well as the Usual Care arm.

Eligibility: Participants who meet the following inclusion criteria may be eligible to take part in the main trial:

- Participant or their legal representative is able and willing to provide informed consent
- Symptoms attributable to COVID-19 starting within the past 5 days and ongoing
- A positive PCR or lateral flow SARS-CoV-2 test
- Aged ≥50 years OR aged 18-49 years with any known underlying chronic health condition considered to make them clinically vulnerable

Adaptive randomisation: Participants in the main trial will be randomised to one trial arm using equal allocation ratios corresponding to the number of eligible arms in the trial. Pre-specified decision criteria allow for dropping an antiviral agent for futility, declaring an antiviral superior, or adding a new antiviral to be tested. If at any point an antiviral agent is deemed superior to the Usual Care, the superior antiviral may become part of Usual Care arm as the new standard of care according to recommended treatment guidelines and changing effects of Usual Care will be taken into account in the analysis.

Outcomes:
Main trial: The primary outcome will be all-cause, non-elective hospitalisation and/or death within 28 days of randomisation. Secondary outcomes will include time to self-reported recovery defined as

For peer review only - http://bmjopen.bmj.com/site/about/guidelines.xhtml
© Copyright: The University of Oxford and Oxford University Hospitals NHS Foundation Trust 2019

the first instance that a participant report feeling fully recovered from the illness; duration of symptoms; symptom recurrence; daily rating of feeling well reported by participants; healthcare service use; participant reported household infection rate; safety outcomes and cost-effectiveness outcomes; symptoms and well-being at three and six months (with determination of proportion with Long COVID) from randomisation.

Virology sampled cohort: The primary outcome will be SARS-CoV-2 viral load at Day 7. Secondary outcomes will include SARS-CoV-2 viral load Days 0-7 and Day 14; SARS-CoV-2 viral genetic whole genome sequence at Day 1, Day 5 and Day 14 and SARS-CoV-2 antibodies at Day 1, Day 5, and Day 14; and to identify any common genetic mutations in patient receiving novel antiviral(s).

See *supplementary material C* for details of objectives and outcome measures.

Efficient trial design: Depending on the drug licensing status and available safety data, all enrolment (screening, informed consent, eligibility review and baseline data) can be done either by PANORAMIC Hubs or by the central trial team, with follow-up procedures (daily diary, data capture of hospitalisations and deaths) conducted remotely with participants using the trial website or a telephone call with the trial team. Randomisation will be online and automatic, following eligibility confirmation.

PANORAMIC Hubs: These will include GP Sites, Community Trusts, and other health service providers, including government agencies e.g., UK Health Security Agency, who will actively identify potential participants and invite them to take part. Potential participants may be referred to Hubs by other NHS facilities for possible inclusion in the trial. A medically qualified professional , research nurse, nurse prescriber or prescribing pharmacist (as specified in the ISA for the specific antiviral involved) from the Hub will complete all recruitment procedures, screening, baseline, informed consent, and eligibility review. Participants will be provided with a participant pack (containing the antiviral agent, if randomised to this arm), either issued by the Hub or sent directly to participants homes. Hubs will be able to store and issue trial antiviral agents. The Hubs will also allow additional safety monitoring visits where required and as defined in the ISA. A Principal Investigator (PI) at each Hub will provide trial oversight for participants recruited via the Hub.

Central recruitment: A central trial team will also be able to recruit and randomise participants and a participant pack containing an antiviral agent (if randomised to this arm) will be sent directly to participants homes.

Data to be recorded: Demographic features including ethnicity will be captured at baseline. In the online daily diary (completed each day for 28 days) and during telephone calls, participants or their Trial Partners will rate the severity of symptoms including how well they are feeling, record contacts with the health services (including hospital admission), record trial medication use, resource use, and new infections in the household. Follow-up beyond 28 days after randomisation will be by accessing electronic medical records and by participant questionnaire for information relevant to the longer-term consequences of COVID-19 at three and six months from randomisation. To investigate the impact of trial interventions on the longer-term effects of COVID-19, we will also remotely follow-up participants, for up to 10 years.

For peer review only

For peer review only - http://bmjopen.bmj.com/site/about/guidelines.xhtml
© Copyright: The University of Oxford and Oxford University Hospitals NHS Foundation Trust 2019

Numbers to be randomised: An estimated maximum of approximately 5300 participants per arm will be required to provide approximately 90% power for detecting a 33% relative reduction in the hospitalisation/death in an experimental arm relative to Usual Care, based on the assumption of an underlying 3% combined hospitalisation/death rate in the Usual Care arm, and an intervention lowering the hospitalisation/death rate to 2%.

To enquire about the trial, contact the PANORAMIC Trial Team:

PANORAMIC Trial
Nuffield Department of Primary Care Health Sciences
Radcliffe Primary Care
Radcliffe Observatory Quarter, Woodstock Road
Oxford
OX2 6GG

Email Address: panoramic@phc.ox.ac.uk

For peer review only

For peer review only - http://bmjopen.bmj.com/site/about/guidelines.xhtml
© Copyright: The University of Oxford and Oxford University Hospitals NHS Foundation Trust 2019

NUFFIELD DEPARTMENT OF PRIMARY CARE HEALTH SCIENCES Primary Care Clinical Trials Unit

PANORAMIC Platform Adaptive trial of NOvel antiviRals for eArly treatMent of COVID-19 In the Community

TABLE OF CONTENTS

1. BACKGROUND and RATIONALE 11
1.1 Aims and objectives 11
2. TRIAL DESIGN AND PROCEDURES 11
2.1 Participant identification 12
2.1.1 Trial participants 12
2.1.2 Inclusion criteria 12
2.1.3 Exclusion criteria 12
2.1.3.1 Additional exclusion criteria for virology sampled cohort only: 12
2.2 Trial procedures 13
2.2.1 Informing potential participants about the trial 13
2.2.2.2 Central recruitment 14
2.2.2.3 Virology sampled cohort recruitment 14
2.3 Screening 14
2.4 Informed consent 15
2.5 Eligibility assessment 16
2.6 Randomisation 17
2.7 Blinding and codebreaking 17
2.8 Follow-up procedures 17
2.9 Virology sampled cohort additional sample processing and storage 20
2.10 Economic evaluation 20
2.11 Early discontinuation/withdrawal of participants 21
2.12 Definition of end of trial 21
3. TRIAL INTERVENTIONS 22
3.1 Medication distribution 22
3.2 Medication adherence 22
4. SAFETY REPORTING 22
4.1 Procedures for reporting Adverse Events (AEs) and SAEs 23
4.1.1. AE reporting 23
4.1.2 AE Severity assessment (for assessing clinician) 23
4.1.3 SAEs 24
4.1.4 Other events exempt from immediate reporting as SAEs 24
4.1.5 Procedure for immediate reporting of SAEs 24
4.1.6 Assessment of causality 24
4.1.7 Expectedness 25
4.2 SUSAR reporting 25

For peer review only

For peer review only - http://bmjopen.bmj.com/site/about/guidelines.xhtml
© Copyright: The University of Oxford and Oxford University Hospitals NHS Foundation Trust 2019

4.3 Development Safety Update Reports 25
5. STATISTICS 26
5.1 Master Statistical Analysis Plan (M-SAP) 26
5.2 Open platform trial 26
5.2.1 Primary efficacy endpoints and analyses 26
5.2.2 Primary efficacy hypothesis & analysis 26
5.2.3 Adaptive design 26
5.2.4 Interim analyses 27
5.2.5 Allocation & adaptive randomisation 27
5.2.6 Sample size justification 27
5.2.7 Virtual trial simulations 29
5.2.8 Procedure for accounting for missing, unused, and spurious data 29
5.3 Primary analysis population 29
5.4 Procedures for reporting unplanned deviation(s) from the M-SAP 29
6. DATA MANAGEMENT 29
6.1 Source data 29
6.2 Access to data 30
6.3 Data recording and record keeping 30
7. QUALITY ASSURANCE PROCEDURES 30
7.1 Risk assessment and monitoring 31
7.2 Trial committees 31
8. PROTOCOL DEVIATIONS 32
9. SERIOUS BREACHES 32
10. ETHICAL AND REGULATORY CONSIDERATIONS 32
10.1 Declaration of Helsinki 32
10.2 Guidelines for Good Clinical Practice 32
10.3 Approvals 32
10.4 Other ethical considerations 33
10.5 Reporting 33
10.6 Transparency in research 33
10.7 Participant confidentiality 33
10.8 Expenses and benefits 33
11. FINANCE AND INSURANCE 33
11.1 Funding 33
11.2 Insurance 33
11.3 Contractual arrangements 34
12. PUBLICATION POLICY 34

For peer review only

For peer review only - http://bmjopen.bmj.com/site/about/guidelines.xhtml
© Copyright: The University of Oxford and Oxford University Hospitals NHS Foundation Trust 2019

13. DEVELOPMENT OF A NEW PRODUCT/ PROCESS OR THE GENERATION OF INTELLECTUAL PROPERTY ... 34
14. ARCHIVING ... 34
15. APPENDIX A: SCHEDULE OF PROCEDURES ... 36
16. APPENDIX B: Participant Flow Diagram ... 39
17. APPENDIX C: AMENDMENT HISTORY ... 40
18. APPENDIX D: SUPPLEMENTARY MATERIAL ... 44
A. Abbreviations ... 44
B. Key trial contacts ... 45
C. Objectives and outcome measures ... 49
D. Adverse Events ... 52
19. APPENDIX E: INTERVENTION SPECIFIC APPENDICES ... 54
1. USUAL CARE ARM ... 54
1. Background and rationale ... 54
2. Detail of intervention ... 54
2. USUAL CARE PLUS MOLNUPIRAVIR ... 55
1. Background ... 55
2. Detail of intervention ... 56
3. Trial visits ... 58
4. Outcome measures ... 58
5. Eligibility criteria (in addition to master protocol) ... 58
6. Professional role of those checking eligibility ... 59
7. Antiviral agent: Molnupiravir ... 59
8. Safety reporting ... 61
3 USUAL CARE PLUS PAXLOVID ... 66
1. Background ... 66
2. Detail of intervention ... 67
3. Trial visits ... 69
4. Outcome measures ... 69
5. Eligibility criteria (in addition to master protocol) ... 69
6. Professional role of those checking eligibility ... 70
7. Antiviral agent: Paxlovid ... 70
8. Safety reporting ... 73
APPENDIX F: STANDARD SCRIPT FOR SAFETY MONITORING OF DRUGS THAT REQUIRE ADJUSTMENT WHEN CO-ADMINISTERED WITH PAXLOVID ... 81
APPENDIX G: PAXLOVID DRUG-DRUG INTERACTIONS ... 82
List A: Alphabetical summary of drugs that may interact with Paxlovid ... 82

For peer review only

For peer review only - http://bmjopen.bmj.com/site/about/guidelines.xhtml
© Copyright: The University of Oxford and Oxford University Hospitals NHS Foundation Trust 2019

List B: Details of Paxlovid drug interactions and implications for eligibility for drugs that are not recommended or require adjustment with Paxlovid in the PANORAMIC trial 85

20. REFERENCES 94

For peer review only

For peer review only - http://bmjopen.bmj.com/site/about/guidelines.xhtml

© Copyright: The University of Oxford and Oxford University Hospitals NHS Foundation Trust 2019

NUFFIELD DEPARTMENT OF PRIMARY CARE HEALTH SCIENCES Primary Care Clinical Trials Unit PANORAMIC Platform Adaptive trial of NOvel antiviRals for eArly treatMent of COVID-19 In the Community

1. BACKGROUND and RATIONALE

Despite high uptake of vaccination against COVID-19, the disease remains prevalent in the UK and in many countries around the world, with many patients continuing to require hospital admission. COVID-19 causes considerable suffering, including loss of ability to perform activities of daily living, loss of educational and work opportunities, and inability to perform caring duties, with far reaching personal and societal consequences. Many go on to experience persisting and/or relapsing symptoms. People with underlying health conditions, unvaccinated people, and those in whom the vaccine is not effective are at increased risk of more severe disease.(1) New 'vaccine escaping' variants may yet emerge, and the impact of early antiviral treatment on long COVID syndromes is as yet unknown. Early treatment with antiviral agents may prevent progression to the later phase of COVID-19. Therefore, there is an urgent need to identify treatments for COVID-19 for use in the community early on in the illness that prevent the need for hospital admission and improves time to recovery.(2, 3)

Antiviral agents may reduce viral shedding, and use of antiviral agents may lead to the emergence of resistance to novel antiviral agents, but the impact of novel antiviral agents on shedding and resistance is not yet known.(4)

1.1 Aims and objectives

Main trial: The primary aim is to determine the effectiveness of selected antiviral agents in preventing hospitalisation and/or death in higher-risk patients with a confirmed positive SARS-CoV-2 PCR or lateral flow test result (see Inclusion/Exclusion Criteria, below).

Virology sampled cohort: A subset of patients from the intervention and comparator arms of the trial will be invited to participate in a virology sampled cohort for virology which aims to determine if there are differences in viral load decay in patients who are/are not treated with antivirals and to identify any common genetic mutations (occurring in greater than 1% of patients) in patient receiving novel antiviral(s).

2. TRIAL DESIGN AND PROCEDURES

PANORAMIC is an open label, prospective, individually randomised, platform, adaptive, controlled clinical trial in community care. Trial arms will include:

Intervention arms: Novel antiviral agents (or combinations) targeting SARS-CoV-2, specified by the Antivirals Taskforce (AT) and with capacity for sequential introduction of each treatment regimen into the trial plus Usual Care.

Comparator arm: Usual Care, defined as the currently recommended treatment delivered by responsible clinicians. Usual Care will not be mandated by the trial, as recommended treatments may change and be tailored to individual characteristics, and self-care will vary. Use of over-the-counter medication as well as key medications such as inhaled steroids and monoclonal antibodies will be captured and changing outcomes and treatment modalities over time in the Usual Care arm will be accounted for in the analysis: see Usual Care ISA.

For peer review only

For peer review only - http://bmjopen.bmj.com/site/about/guidelines.xhtml
© Copyright: The University of Oxford and Oxford University Hospitals NHS Foundation Trust 2019

2.1 Participant identification

2.1.1 Trial participants

The trial includes participants who test positive for SARS-CoV-2 infection and with ongoing symptoms consistent with COVID-19, not hospitalised, and who are aged 50 years and over, or 18-49 years and considered clinically vulnerable (see Inclusion Criteria below).

2.1.2 Inclusion criteria

- Participant is able and willing to provide informed consent, or their legal representative is willing to provide informed consent
- Symptoms attributable to COVID-19 started within the past 5 days and ongoing
- A positive PCR or lateral flow SARS-CoV-2 test*
- Aged ≥50 years OR aged 18-49 years with one of the following known underlying chronic health conditions considered to make them clinically vulnerable:
    - chronic respiratory disease (including chronic obstructive pulmonary disease (COPD), cystic fibrosis and asthma requiring at least daily use of preventative and/or reliever medication)
    - chronic heart or vascular disease
    - chronic kidney disease
    - chronic liver disease
    - chronic neurological disease (including dementia, stroke, epilepsy)
    - severe and profound learning disability
    - Down's syndrome
    - Diabetes mellitus (Type or Type II)
    - immunosuppression: primary (e.g., inherited immune disorders resulting from genetic mutations, usually present at birth and diagnosed in childhood) or secondary due to disease or treatment (e.g., sickle cell, HIV, cancer, chemotherapy)
    - solid organ, bone marrow and stem cell transplant recipients
    - morbid obesity (BMI >35)
    - severe mental illness
    - care home resident
    - judged by recruiting medically qualified professional, research nurse, nurse prescriber, prescribing pharmacist, dependent on the ISA for the specific IMP involved, to be clinically vulnerable

* Any positive PCR or lateral flow test taken up to two days before symptom onset and randomisation qualifies.

2.1.3 Exclusion criteria

- Patient currently admitted to hospital (inpatient)
- Previous randomisation in the PANORAMIC trial
- Currently participating in a clinical trial of a therapeutic agent for acute COVID-19
- Additional exclusions specific to each intervention arm, if any, as listed in the ISA's of currently open trial arms

2.1.3.1 Additional exclusion criteria for virology sampled cohort only:

- Receipt of a non-trial anti-SARS-CoV-2 antibody therapy within the previous 3 months

For peer review only - http://bmjopen.bmj.com/site/about/guidelines.xhtml
© Copyright: The University of Oxford and Oxford University Hospitals NHS Foundation Trust 2019

2.2 Trial procedures

2.2.1 Informing potential participants about the trial

I. All Health, health related, and Social Care professionals (including NHS 111 and Test and Trace clinicians, care home staff, pharmacy staff, etc) will be able to provide information about participation and direct potential participants to the online trial information and the trial website

II. The ZOE COVID-19 Application, Health Wise Wales, Join Dementia Research (JDR) and other COVID-19 research studies e.g., REACT, VIRUS WATCH) will sign-post to the trial.

III. National media campaigns will use television, radio, and social media platforms to generate awareness of the trial and to signpost to the trial

IV. Targeted campaigns for vulnerable groups will be by media campaigns, via national charities, social media groups and relevant secondary care clinicians.

V. All NHS facilities including testing centres including NHS walk in/ drive through centres will be able to inform potentially eligible participants about the trial and refer them to the trial website and/or trial team

VI. Clinicians can reach out to potentially eligible participants identified by receiving SARS-CoV-2 test results from Test and Trace and laboratories, and by regular searches for patients with a positive SARS-CoV-2 test result in their clinical database. Contact can be made with potential participants verbally or by text, email, and telephone

VII. NHS Digital (and analogous services in devolved administrations) will provide a daily list of contact details from Pillar 2 testing data of people with a positive SARS-CoV-2 test. The trial team and the Hubs will be able to contact these people within 24-48hrs of test result to discuss participation. Patient details will be provided in accordance with section 251 under the General Notice under the Health Service Control of Patient Information Regulations 2002 (COPI). The COPI notice provides a temporary legal basis to allow access to participant data and protects participants whilst avoid confidentiality breaches for COVID-19 purposes. COPI is only applicable to Hubs in England and Wales. Following the expiration of the current COPI notice, PANORAMIC will gain access to and process participant identifiable information, in England and Wales only, without consent under Regulation 5 of the Health Service (Control of Patient Information) Regulations 2002 as amended by Section 117 of the Care Act 2014.

VIII. EMIS Anywhere, a data extraction service for primary care data, and analogous general practice clinical record facilities, will be able to reach out to potentially eligible participants and signpost them to the PANORAMIC website to explore their participation

2.2.2 Recruitment

Face-to-face as well as remote (trial website or telephone call) screening, eligibility and consent procedures will be used. All participants (apart from those who lack capacity to do this) will have a two-way discussion, either face-to-face or by a telephone/video call from a medically qualified professional , research nurse, nurse prescriber or prescribing pharmacist, dependent on the ISA for the specific IMP involved, prior to randomisation.

For participants who are too unwell or unable to respond to surveys for themselves, a Trial Partner they identify will be able to assist their participant in completing screening, baseline, consent and follow up online forms and/or calls and provide information to them on their behalf where necessary. A letter will be issued to Trial Partners, informing them of the trial, notifying them that they have been nominated for this role by the participant.

For peer review only

For peer review only - http://bmjopen.bmj.com/site/about/guidelines.xhtml

© Copyright: The University of Oxford and Oxford University Hospitals NHS Foundation Trust 2019

2.2.2.1 Recruitment at PANORAMIC Hubs

PANORAMIC Hubs will include GP sites (either single practices or a federation of practices that are able to operate under a single site agreement and PI to undertake trial procedures as detailed in the protocol), community trusts, and other health service providers, including government agencies e.g., UK Health Security Agency. Potential participants can be referred to Hubs by other health care facilities for possible inclusion. As well as recruiting patients through routine consultations, Hubs will search their databases and test results they receive for patients defined as clinically vulnerable (see inclusion criteria for definition) with a positive test for COVID-19, and telephone or text them to invite them to take part in the trial. Either face-to-face or by telephone, a medically qualified professional , research nurse, nurse presciber or prescribing pharmacist, as specified in the ISA for the specific IMP involved, at the Hub will explain the trial to the potentially eligible participant; collect screening, baseline, and contact information; take informed consent; and confirm eligibility (see details below for each trial procedure). If the participant is eligible, they will automatically be randomised to one of the trial arms and provided with a participant pack (see section 3.1 Medication Distribution).

A PI at each Hub will provide trial oversight, for participants recruited via the Hub and inform the central trial team of any Serious Adverse Events (SAE).

2.2.2.2 Central recruitment

Potential participants can present directly to the central trial team via the trial website or free-phone telephone number, in additional to via a PANORAMIC Hub. Screening, baseline, contact information and informed consent can be self-completed by the potential participant, or completed during a telephone call with a member of the central trial team. A medically qualified professional or appropriately trained research nurse will then confirm eligibility. If eligible, the participant will be randomised and provided with a participant pack (see section 3.1 Medication Distribution). All trial procedures are described below in detail.

2.2.2.3 Virology sampled cohort recruitment

The virology sampled cohort will consist of enhanced monitoring of a subset of participants who additionally volunteer for this aspect of the trial in each arm of the trial. Recruitment will be from PANORAMIC Hubs that are assigned virology sampled recruiting sites, or through the central trial team.

2.3 Screening

Screening can be completed face-to-face as well as remotely via the trial website, or a free-phone telephone service that enables participants to have a two-way discussion with the central trial team or Hub staff who are trained in trial procedures.

Participants of child-bearing potential are required to confirm a negative pregnancy test prior to starting any antiviral agent in the trial that may be teratogenic, and as specified in its ISA. Thus, they should indicate willingness to take such a pregnancy test at screening. For those recruited at face-to-face visits at PANORAMIC Hubs, undertaking a pregnancy test will be part of the initial screening visit. For participants recruited remotely, the pregnancy test will be supplied in the participant pack with the antiviral agent. The pregnancy test must be completed prior to starting an antiviral agent that requires confirmation of a negative pregnancy test before staring the agent. This will be clearly

For peer review only - http://bmjopen.bmj.com/site/about/guidelines.xhtml
© Copyright: The University of Oxford and Oxford University Hospitals NHS Foundation Trust 2019

explained prior to randomisation (see section 2.8 Follow-up Procedures for details regarding confirmation of a negative test result).
Those who are ineligible because they are asymptomatic will be alerted to possible trial participation should they develop symptoms.

2.4 Informed consent

There are separate procedures for recruiting eligible participants with capacity to give informed consent and residents of care homes who lack capacity to consent. All consent forms will be completed online and paperless.

Eligible participants capable of giving informed consent will be asked to provide informed consent after a two-way discussion between a medically qualified professional , research nurse, nurse prescriber or prescribing pharmacist, as specified in the ISA for the specific IMP involved, and the participant, either face-to-face or by telephone, prior to randomisation, where the risks and benefits of taking part and follow-up procedures will be explained.

In addition to taking consent face-to-face, consent may also be taken remotely, using online paperless consent forms and via telephone/video discussion, because of the pandemic circumstances and the need to maximise the pragmatic nature of the trial. Participants will be able to download their consent form after completion, and it can be printed by the central trial team and delivered to participants. Electronic consent forms will be held securely on the trial database. For those recruited in Hubs, a copy will be filed in patients' medical notes and a copy will be printed and given to patients.

Prior to consent, written and summary versions of the Patient Information Sheet (PIS), and Informed Consent Form (ICF) will be available to participants detailing no less than: the exact nature of the trial; and the known side-effects and risks involved in taking part. It will be clear that the participant is free to withdraw from the trial at any time. A pictorial and/or video and a summary PIS will be available which can be more easily read by those feeling very unwell, or those with low reading comprehension skills. Adequate time will be given to the participant to consider the information and to ask any questions about the trial before deciding whether to participate. After consent, participants will enter online baseline information, including their address, contact details and those of a Trial Partner. Identifying a Trial Partner is not a requirement of trial participation.

People who lack capacity to consent for themselves will only be recruited from care homes: adults who lack capacity to consent living elsewhere will not be recruited. If the recruiting health and social care professional deems that a patient in a care home lacks capacity to provide consent for themselves, then a personal or professional legal representative (England and Wales only) will be asked to provide consent. A personal legal representative is defined as a person not connected with the conduct of the trial who is suitable to act as the legal representative by virtue of their relationship with the adult. A professional legal representative may be a doctor responsible for the medical treatment of the adult if they are independent of the trial, or a person nominated by the healthcare provider. In all instances, a personal legal representative will be sought first, and a professional legal representative sought only if a personal legal representative cannot be identified. Legal representative and recruiting clinicians will not endeavour to obtain consent for or recruit people into the trial people who, in addition to their lack of capacity, have a quality of life which can reasonably be considered as not acceptable to the potential participant to avoid potentially life lengthening

For peer review only - http://bmjopen.bmj.com/site/about/guidelines.xhtml
© Copyright: The University of Oxford and Oxford University Hospitals NHS Foundation Trust 2019

intervention in those who would not wish to have such an intervention. Legal representative consent (relative/family member/independent treating physician) can be taken face to face or remotely.

The legal representative will be provided with information about the trial and made aware of the following: they are being asked to give consent on behalf of the incapacitated adult, they are free to decide whether they wish to make this decision or not, and they are being asked to consider what the adult would want, and to set aside their own personal views when making this decision.

2.5 Eligibility assessment

For participants who have provided consent, eligibility will be assessed by a medically qualified professional, research nurse, nurse prescriber or prescribing pharmacist, as specified in the ISA for the specific IMP involved, at a PANORAMIC Hub, other health service providers including government agencies e.g., UK Health Security Agency or by the central clinical team. For some antiviral agents, eligibility may only be assessed by a medically qualified professional, and the professional roles of each Health Care Professional (HCP) qualifying them to do this will be specified in the ISA for each agent.

PANORAMIC Hubs can contact the central clinical team for guidance regarding eligibility queries. Depending on the exclusion criteria outlined in ISAs, eligibility can be assessed by eliciting medical history and relevant information, including a drug history, directly from the participant, and the participant can be randomised if they are deemed eligible and there is no contraindication to the trial drugs currently in the trial. Where specified in the ISA, eligibility checking will be assessed additionally through direct access to the participant's Summary Care Record in England or a medical record summary in use for clinical care in any UK Devolved Administration, and by reference to relevant medical information obtained from the participant's primary care or secondary care records (where the person confirming eligibility deems this necessary)..

Potential participants will be informed that those at the highest risk of complications from COVID-19 are able to get antiviral treatment outside of the trial from the NHS.

If an additional IMP is introduced into the trial, which requires extensive clinical interpretation of the eligibility criteria, the eligibility assessment process will be reviewed and amended accordingly and outlined fully in the ISA with screening and eligibility CRFs and associated processes updated accordingly.

For peer review only

For peer review only - http://bmjopen.bmj.com/site/about/guidelines.xhtml
© Copyright: The University of Oxford and Oxford University Hospitals NHS Foundation Trust 2019

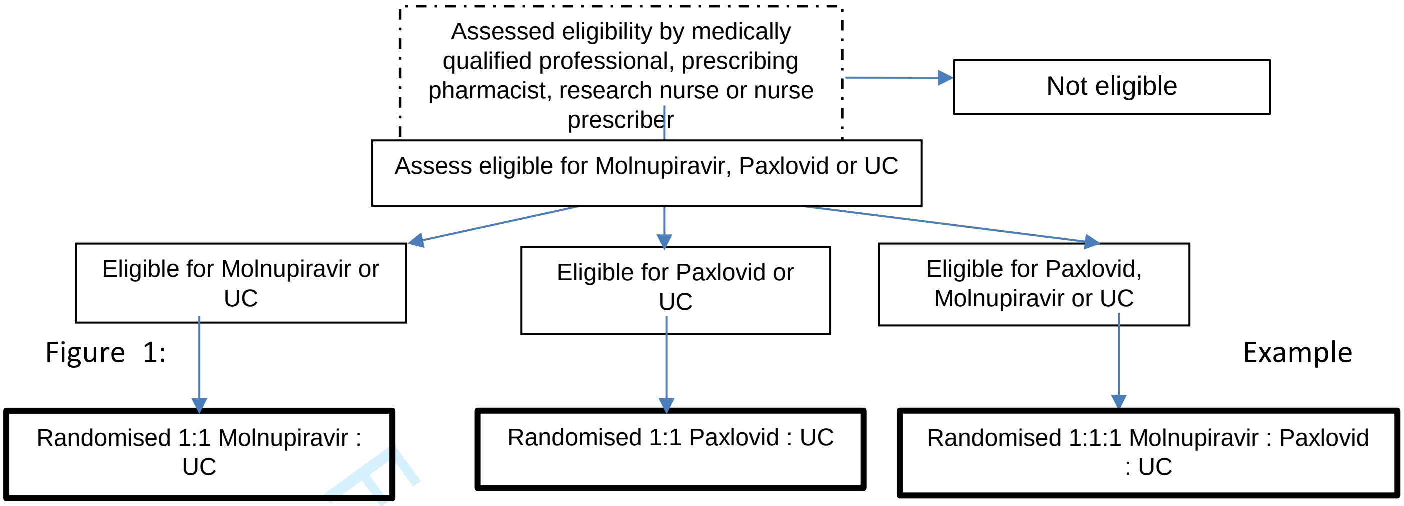

Figure 1: Example of eligibility flow for randomisation when adding Paxlovid as a new intervention.

2.6 Randomisation

Participants will be randomised using a secure, fully validated, and compliant web-based randomisation system. Once deemed eligible, a medically qualified professional , research nurse, nurse prescriber or prescribing pharmacist, as specified in the ISA for the specific IMP involved, from the central clinical team or Hub (as documented on the delegation log) will randomise the participant. Participants will be randomised to one trial arm using equal allocation ratios corresponding to the number of eligible arms for which the participant is eligible for in the trial. For instance, if there are two active interventions (A & B), the allocation ratio will be 1:1:1 for Usual Care, active A, active B (respectively), such that 33% of participants are randomised to Usual Care. If there are 3 active interventions, the allocation ratio will be 1:1:1:1, such that 25% of participants are randomised to Usual Care. Patients must be eligible for at least two arms (Usual Care and at least one novel antiviral intervention). Stratification will be by age and vaccination status.

The randomisation database will automatically alert the relevant IMP distributor and the participant, trial team and legal representative if applicable will be notified electronically of the treatment allocation. If the participant does not have an email address, they will be notified by telephone.

2.7 Blinding and codebreaking

PANORAMIC is an open-label trial. The participant, legal representative if applicable, and the recruiting clinician will know the participant's allocation. Therefore, no unblinding or code breaking is required. However, those managing the data will be blind to participant allocation.

The trial team and recruiting clinicians will be blinded to emerging results of interim analyses. During the course of the trial, only the unblinding statisticians and the independent members of the Data and Safety Monitoring Committee (DSMC) will have access to the unblinded interim results.

2.8 Follow-up procedures

Following randomisation, participants in the intervention arm will be sent a participant pack (see section 3.1 Medication Distribution). The participant pack will contain: the antiviral agent, an information booklet; participant card detailing how the medication should be administered, precautions and safety guidance; medication appendix providing further information about the treatment (available prior to randomisation as part of the PIS); wallet emergency card; pregnancy test

For peer review only - http://bmjopen.bmj.com/site/about/guidelines.xhtml
© Copyright: The University of Oxford and Oxford University Hospitals NHS Foundation Trust 2019

(only for participants of child-bearing potential). Those randomised to Usual Care, will receive an information booklet via email or post.

The participant pack for participants randomised to the intervention arm who have consented to take part in the virology sampled cohort will be supplemented with; an additional virology sampling kit containing approved instructions, and materials to post samples to the virology processing sites which will be posted separately to participants. Those randomised to Usual Care, will receive these additional materials in addition to an information booklet via email or post.

Patients might be asked to attend a face-to-face visit or to donate a microbiological or blood sample, depending on the requirements for the evaluation of each specific antiviral agent. This will depend on the antiviral agents' licensing status, available safety data and their approval status. Thus, for antiviral agents with an established safety profile, follow-up will be via self-completed questionnaires online or through telephone calls, and primary care and/or hospital record searches. For other antiviral agents, the trial will have capability for face-to-face assessment, sampling, and safety checks initially, after which a drug may progress to 'remote evaluation', which will only be implemented following approval of a substantial amendment.

A safety call will be made on Day 1 (day after randomisation) with participants of child-bearing potential who have been allocated to an antiviral agent with teratogenic potential (as specified in the relevant ISA) by a member of the central trial team or the recruiting PANORAMIC Hub, to confirm receipt of the participant pack (containing a urine pregnancy test). During this Day 1 call, a member of the trial team will confirm with participants of childbearing potential, that a pregnancy test has been done and that the result is negative before starting an antiviral agent with teratogenic potential. In the event of a positive test result, the participant will be asked not to take any of the antiviral agent, return it, and will be withdrawn from the trial. Results will be documented in the Day 1 Call CRF. The pregnancy test must be completed prior to taking the antiviral agent in question and this will be clearly explained prior to randomisation. Participants of child-bearing potential will also be asked to confirm a negative pregnancy test result in their day 1-3 of daily diaries.

All participants, irrespective of group allocation, will be contacted on Day 2 (2 days after randomisation) to confirm receipt of trial materials, confirm follow-up procedures and answer queries. This call will be made by a member of the central trial team or the PANORAMIC Hub. At this day 2 call, participants allocated to any antiviral agent arm of the trial, will be also asked if they have received their trial pack and if they are experiencing any potential side-effects from the IMP. This call will be made by clinicians, research nurses, nurse prescribers or prescribing pharmacists, dependent on the ISA for the specific IMP involved, from the central trial team (for those recruited centrally or from a Hub) or PANORAMIC Hub (for those recruited via their Hubs). For higher risk IMPs, additional safety calls may be made as detailed in the relevant ISA.

If the participant or their Trial Partner cannot be reached at this stage, the trial team will contact the patient's GP to request information on any healthcare contacts that the participant may have had since they were enrolled into the trial, to capture any potential safety events.

Participants on all arms of the trial will be asked to complete a daily diary each day for 28 days and be contacted at 3 and 6 months from randomisation, where they will rate the severity of symptoms, record contacts with the health services (including hospital admissions, hospital outpatient visits, accident and emergency attendances, use of specialist services and primary care encounters), impact

For peer review only - http://bmjopen.bmj.com/site/about/guidelines.xhtml
© Copyright: The University of Oxford and Oxford University Hospitals NHS Foundation Trust 2019

of symptoms on work/trial, record medication use and new infections in the household. We will collect the *EuroQoL EQ-5D-5L* (baseline, days 14 and 28, and 3 and 6 months). The central trial team will call participants/trial partners with no internet access or those who have not completed their diary for at least two consecutive days before days 7, 14 and 28. No more than six contact attempts will be made at each of these follow-up points. All participants will be telephoned within one day, and 24-hour access to the safety phone line and emergency procedures will be emphasised to those randomised to an antiviral agent. Participants will be contacted at three and six months to ascertain wellbeing and longer-term consequences of their illness, including proportion meeting criteria for 'long Covid'. Vaccination status, including number of vaccinations received will be recorded.

Adherence to trial medication will be assessed by self-report.

Participants' medical records will be accessed up to twelve months following enrolment to ascertain follow up data from enrolment to 6 months. Data will be collected as close to real time as possible; RCGP RSC, EMIS, NHS Digital, electronic Data Research and Innovation Service (eDRIS), The Secure Anonymised information Linkage (SAILS) Databank, Health and Social Care Northern Ireland (HSC Business Services Organisations/HSC Trusts) (HSC NI) and other sources of routinely collected data will be utilised if required. To investigate the impact of trial interventions on the longer-term effects of COVID-19, we will use these data collection methods to follow-up participants, for up to 10 years.

Virology samples cohort: 300 participants from each trial intervention arm and the Usual Care comparator arm will be recruited to enrol into the voluntary virology sampled cohort. Participants will fall into two categories; the first 30 patients volunteering to enrol from each trial arm will undergo intensive daily viral load monitoring, whereas the remaining 270 from each arm in the virology samples cohort will have less intensive viral load monitoring.

The first 30 participants in each arm will be asked to provide daily nasopharyngeal swabs for 7 days, and an additional nasopharyngeal swab on Day 14 (+/- 1 day). For patients in the intervention arms, the first sample will be taken immediately prior to the participant commencing anti-viral treatment (Day 1). For participants allocated to Usual Care Day 1 will be the day following randomisation.

The next 270 participants volunteering for this aspect of the trial in each arm will be asked to provide 3 nasopharyngeal swabs: once prior to starting treatment, once on Day 5 (+/- 1 day) and once on Day 14 (+/- 1 day).

All participants volunteering for this aspect of the trial will be asked to take 3 finger prick dried blood spot samples: once pre-treatment, once on Day 5 (+/- 1 day) and once on Day 14 (+/- 1 day).

Participants consenting to take part in the virology sampled cohort will be sent CE-IVD approved sampling kits for nasopharyngeal sampling, dried blood spot sampling, pre-paid postage, and packaging, to post samples to the virology processing site. The kits will include approved instructions and will be delivered to the participant by courier from a central stock or the PANORAMIC Hubs. Sampling may occur at home or at Hubs, with participants supported by the Hubs or the central trial team. Samples taken at home should be posted to the trial team within 3 days of sampling, and ideally within 24 hours.

For peer review only - http://bmjopen.bmj.com/site/about/guidelines.xhtml
© Copyright: The University of Oxford and Oxford University Hospitals NHS Foundation Trust 2019

Hubs and the central trial team will receive training in all virology sampling procedures from the Royal Free/University College London (UCL) team who will provide ongoing support to the Hubs and central trial team.

A telephone call and/or SMS text message/email reminder will be sent to participants who have enrolled into the intensive monitoring cohort (the first 30 in each trial arm) on Day 4 (+/- 1 day), Day 7 (+/-1 day) and Day 14 (+/- 1 day).

2.9 Virology sampled cohort additional sample processing and storage

Viral load in the upper respiratory tract rises to a peak at symptom onset, becoming undetectable in 1 or 2 weeks in most patients. The primary aim of this intensively sampled cohort is to assess the impact the antiviral agents have on viral load, with a focus on prediction of time to virus clearance. Important confounders of this are presence of antibodies and so these will be monitored.

The secondary aim is to evaluate the potential for antivirals to cause mutations. For those samples containing a sufficient viral load, whole genome sequencing of the pre- and post-treatment samples will be performed.

Viral load determination and viral genome sequencing will be performed using material extracted from nasopharyngeal swabs.

Since antibody status is likely most crucial to viral dynamics, it will be measured in dried blood spots collected via finger pricks as described above at Day 0, Day 5, and Day 14.

Samples will be labelled with the participant's trial ID number and the date of sample collection. Nasopharyngeal swabs will be sent to Great Ormond Street Hospital (GOSH) for Children who will process the samples for viral load and forward them to UCL for sequencing. Samples will be accessed by GOSH and UCL members of the trial team. Dried blood spots will be sent to Institute of Immunology and Immunotherapy Birmingham for processing to determine antibody status. After analyses samples will be returned to the research team and with participants consent may be stored for 12 months following the end of the trial. If consent is held for long-term storage, these samples may be used for future ethically approved research. However, where no consent is held samples will be destroyed on completion of the analyses in line with the Human Tissue Act 2004.

2.10 Economic evaluation

A prospective economic evaluation will be embedded within the trial design to assess the cost effectiveness of each antiviral from an NHS perspective. We will estimate the resource inputs associated with embedding each trial antiviral treatment into routine clinical practice and estimate societal costs. Broader resource use will be drawn from General Practice Data for Planning and Research (GPDPR) data and linked Hospital Episode Statistics – encompassing primary care encounters, hospital inpatient/day case admissions, outpatient visits, and accident and emergency attendances. Unit costs will be valued using national reference tariffs and attached to resource inputs to generate a compound total health care cost per trial participant over the trial time horizon. EQ-5D-5L data will be converted using standard algorithms into utility scores for quality-adjusted life year (QALY) estimation, and cost-effectiveness expressed as incremental cost per QALY gained (5). Secondary expressions of cost-effectiveness will include incremental cost per hospitalisation and/or death prevented over 28 days.

For peer review only - http://bmjopen.bmj.com/site/about/guidelines.xhtml
© Copyright: The University of Oxford and Oxford University Hospitals NHS Foundation Trust 2019

NUFFIELD DEPARTMENT OF PRIMARY CARE HEALTH SCIENCES Primary Care Clinical Trials Unit

PANORAMIC Platform Adaptive trial of NOvel antiviRals for eArly treatMent of COVID-19 In the Community

Bivariate regression of costs and measures of health consequence, with multiple imputation of missing data, will be conducted to generate within-trial estimates of incremental cost-effectiveness. Sensitivity analyses will assess the impact of areas of uncertainty surrounding components of the economic evaluation. Cost-effectiveness acceptability curves will show the probability of cost-effectiveness of each treatment evaluated at alternative cost-effectiveness thresholds. Cost-effectiveness threshold values will be informed by guidance from UK government departments on the value placed by decision-makers on an additional QALY (6) and on a statistical life (7).

A decision-analytic modelling-based economic evaluation will also be conducted. The baseline decision-analytic model will be developed during the early stages of the trial and aim to provide a framework for extrapolating the cost-effectiveness of each antiviral beyond the parameters of PANORAMIC trial. Accepted guidelines for good practice in decision-analytic modelling will be followed. The model will consider the progression of symptomatic COVID-19 status over time, and the model structure will capture disease progression using health states that represent the important natural history and clinical- and event-related activity for symptomatic COVID-19 symptomatic status, the appropriate model type (e.g., Markov or discrete-event simulation approach) and the appropriate analytical framework (e.g., cohort analysis versus individual-level simulation). Parameter inputs into the model will be informed by data extracted from PANORAMIC trial, supplemented by data identified from external sources following targeted literature searches. As with the within-trial economic evaluation, cost-effectiveness will be expressed in terms of incremental cost per QALY gained. Multi-parameter uncertainty in the model will be addressed using probabilistic sensitivity analysis. Cost-effectiveness acceptability curves will be used to show the probability of cost-effectiveness of each anti-viral strategy at alternative cost-effectiveness thresholds held by decision-makers. Long-term costs and health consequences will be discounted using nationally recommended discount rates. Specific plans for the economic evaluation will be outlined in a pre-specified health economics analysis plan.

2.11 Early discontinuation/withdrawal of participants

Each participant or their legal representative on the participant's behalf, has the right to withdraw from the trial at any time. For those that lack capacity, expression of dissent in any form will be taken as an indication they do not wish to be included and they will be withdrawn. In addition, the Investigator may discontinue a participant from the trial at any time if the Investigator considers it necessary for any reason including:

- Ineligibility (either arising during the trial or retrospectively)
- Withdrawal of consent

The reason for withdrawal will be recorded on the CRF. Data that has already been collected about the participant will be kept and used. Samples collected from participants and data arising from the processing of those samples for research purposes may be used in the trial analysis.

2.12 Definition of end of trial

The end of the trial will be the last data capture of last participant.

For peer review only - http://bmjopen.bmj.com/site/about/guidelines.xhtml
© Copyright: The University of Oxford and Oxford University Hospitals NHS Foundation Trust 2019

3. TRIAL INTERVENTIONS

Antiviral agent information can be found in the relevant ISAs.

3.1 Medication distribution

In general, the distribution of antivirals can be implemented by the PANORAMIC Hubs; an accredited licensed central facility; an online, community or hospital pharmacy, and the PC-CTU, if approved by MHRA. Distribution of trial packs to participants will be tracked via courier or call/text message. Clinicians may be able to prescribe trial antivirals that can be issued in the community, and pharmacies can issue antivirals to the patient by community pharmacy services or ‘on-line pharmacy’ services, or it can be collected from the pharmacy by the participant or someone on their behalf (with appropriate infection control measures).

The arrangements for the distribution of each antiviral agent are detailed in the ISA.

3.2 Medication adherence

Medication adherence will be captured in daily diaries and phone or video calls from the trial team.

Accountability logs will be kept by the distributor (as specified in the ISA) and central monitoring of the logs will allow oversight by the PC-CTU.

A member of the central clinical team or PANORAMIC Hub will telephone all participants to confirm receipt of the antiviral agent, and that the participant has read the instructions on the participant card. Receipt will be documented in the Day 1 or Day 2 telephone calls (see section 2.8 Follow-up procedures). If we are unable to contact participants or their trial partner, we will confirm and log receipt of antiviral agent by checking the patient’s daily diary, where they are asked daily whether they have taken their trial treatment and the number of tablets/capsules taken. We can also check via the courier portal, whether the medication has been received by the participant, for additional confirmation.

If a participant decides that they no longer wish to take their medication, we will provide a pre-paid envelope so that they can return the medication to the trial team via courier and the trial team will ensure all drug accountability logs are updated accordingly.

4. SAFETY REPORTING

Symptoms, potential medication side-effects and Serious Adverse Events (SAE) will be collected from participant daily diaries, calls to participants/Trial Partners, face-to-face visits with Hub clinicians, medical records, notes reviews, NHS Digital, eDRIS, SAIL, HSC NI, data extracts and RCGP data downloads.

We will adopt a risk assessed and proportionate approach to safety monitoring. In line with the SmPC or Investigator Brochure, we will assess the risks and the safety profile for each antiviral agent, and detail the mitigation and monitoring procedures in the ISA. All safety procedures will be according to PC-CTU pharmacovigilance SOP.

For peer review only - http://bmjopen.bmj.com/site/about/guidelines.xhtml
© Copyright: The University of Oxford and Oxford University Hospitals NHS Foundation Trust 2019

NUFFIELD DEPARTMENT OF PRIMARY CARE HEALTH SCIENCES Primary Care Clinical Trials Unit

PANORAMIC Platform Adaptive trial of NOvel antiviRals for eArly treatMent of COVID-19 In the Community

4.1 Procedures for reporting Adverse Events (AEs) and SAEs

The participant will be asked to rate the severity of a number key COVID-19 symptoms which are also possible common medication side effects in their daily diary. The severity of individual events and symptoms will be assessed over time by participants on the following scale: no problem/mild problem/moderate problem/major problem.

	Participant reported symptom rating
**No problem**	Individual symptom not currently experienced
**Mild problem**	Symptom is short-lived or mild; medication may be required. No limitation to usual activity
**Moderate problem**	Symptom causes moderate limitation in usual activity. Medication may be required.
**Major problem**	Symptom causes considerable limitation in activity. Medication or medical attention required.

Symptoms of COVID-19 and medication AE symptoms may overlap and can be difficult to disentangle. Trends in the prevalence in the severity of symptoms between Usual Care and antiviral agent arms will be compared, for evidence of increased severity of measured symptoms in those randomised to receive trial antiviral agents.

4.1.1. AE reporting

For each antiviral agent, we will only collect AEs (other than those pre-specified symptoms collected via the participant diaries) if and when specified in the relevant ISA. If there is a requirement to collect AEs or specific AEs for an antiviral agent these will be monitored from the start of treatment for the 28-day trial duration, unless otherwise specified in the ISA, and assessed by a clinician (independent from the Sponsor) for causality and severity (definitions below).

Participants will be free to withdraw from taking the antiviral if they perceive they have an intolerable AE. Participants will also be provided with a Participant Card detailing potential side-effects and a Wallet Emergency Card with 24-hour contact telephone line, answered by a clinical team, enabling them to report AEs they experience whilst taking the drug. This card will also alert hospital clinicians about trial participation, should a participant be admitted to hospital. In the event of a medical emergency, trial participants will be instructed to show this card to the clinician they see. Based on clinical judgement, the clinician may contact the participant directly within 24 hrs of becoming aware of an AE reported in their daily diary or on the Freephone number, to advise the participant on the appropriate clinical care.

4.1.2 AE Severity assessment (for assessing clinician)

	Clinical assessment of severity
**GRADE 1 (Mild)**	Short-lived or mild symptoms; medication may be required. No limitation to usual activity
**GRADE 2 (Moderate)**	Moderate limitation in usual activity. Medication may be required.
**GRADE 3 (Severe)**	Considerable limitation in activity. Medication or medical attention required.

For peer review only - http://bmjopen.bmj.com/site/about/guidelines.xhtml
© Copyright: The University of Oxford and Oxford University Hospitals NHS Foundation Trust 2019

4.1.3 SAEs

All-cause hospitalisation and/or death is the primary outcome, and this data will be captured in CRFs. SAEs other than hospitalisation or death due to COVID-19 must be reported for all antiviral agents.

SAEs must be reported to PC-CTU by the person who has discovered the SAE or nominated delegate within 24 hours of becoming aware of the event. The sponsor or delegate will ensure it is reviewed by the CI or other delegated personnel for relatedness and expectedness as soon as possible taking into account the reporting time for a potential SUSAR according to the relevant competent authority. If the event has not resolved, at the 28-day time point the SAE will be reviewed again by the central clinical team, to see if resolution has occurred. If the event is considered 'resolved' no further follow up is required. If not, the event must be followed up until such a time point.

All SAEs that have not resolved by the end of the trial or those that are identified retrospectively, or that have not resolved upon discontinuation of the participant's participation in the trial, must be followed until any of the following occurs:

- The event resolves
- The event stabilises
- The event returns to "baseline", if a "baseline" value/status is available
- The event can be attributed to agents other than the trial intervention or to factors unrelated to trial conduct
- It becomes unlikely that any additional information can be obtained (participant or health care practitioner refusal to provide additional information, lost to follow-up after demonstration of due diligence with follow-up efforts)

See Appendix D. Supplementary Material for definitions of AEs

4.1.4 Other events exempt from immediate reporting as SAEs

Hospitalisations will be defined as at least a one-night admission to hospital, or at least one night in a 'Hospital at Home' program after hospital assessment. Hospitalisation for a pre-existing condition, including elective procedures planned prior to trial entry, which has not worsened, does not contribute to our primary outcome, and does not constitute an SAE.

4.1.5 Procedure for immediate reporting of SAEs

- Trial team/responsible clinician/GP Practice/CI will complete an SAE report form, directly into the database, for all reportable SAEs
- GP practice/trial team/RCGP will provide additional, missing or follow up information in a timely fashion
- If necessary, the participant/trial partner may be contacted to provide additional, missing or follow up information as required

An investigator, who is independent to the Sponsor but part of the trial team, will review the SAE once reported, collect as much information and report to the Sponsor delegate within the timeframe according to the PC-CTU SOPs.

4.1.6 Assessment of causality

For peer review only - http://bmjopen.bmj.com/site/about/guidelines.xhtml
© Copyright: The University of Oxford and Oxford University Hospitals NHS Foundation Trust 2019

The relationship of each SAE to the antiviral agent must be determined by a medically qualified individual according to the following definitions:

- **Unrelated** – where an event is not considered to be related to the antiviral agent
- **Possibly** – although a relationship to the antiviral agent cannot be completely ruled out, the nature of the event, the underlying disease, concomitant medication, or temporal relationship make other explanations possible
- **Probably** – the temporal relationship and absence of a more likely explanation suggest the event could be related to the antiviral agent
- **Definitely** – the known effects of the antiviral agent, its therapeutic class or based on challenge testing suggest that the antiviral agent is the most likely cause

Reported SAEs will be assessed for relatedness by an individual who is independent of the Sponsor (i.e., either the Hub PI or an independent reviewer). An independent reviewer is an investigator independent of the Sponsor, but part of the trial team.

AEs/SAEs judged possibly, probably, or definitely related will be considered as related to the antiviral agent.

4.1.7 Expectedness

Expectedness of SAEs will be assessed and determined by delegated members of the central trial team or by an independent reviewer. Expectedness will be assessed in accordance with the relevant Reference Safety Information (RSI) section of the Summary of Product Characteristics (SmPC) Investigator's Brochure (IB). The RSI will be the current Sponsor and MHRA approved version at the time of the event occurrence.

4.2 SUSAR reporting

All SUSARs will be reported by the sponsor delegate to the relevant Competent Authority and to the REC and other parties as applicable. For fatal and life-threatening SUSARs, this will be done no later than seven calendar days after the Sponsor or delegate is first aware of the reaction. Any additional relevant information will be reported within eight calendar days of the initial report. All other SUSARs will be reported within 15 calendar days.

PIs will be informed of all SUSARs for the relevant antiviral agent for all studies with the same Sponsor, whether or not the event occurred in the current trial.

4.3 Development Safety Update Reports

The DSUR will be developed and submitted annually on the anniversary date that the trial receives Clinical Trial Authorisation +60 days. Due to the nature of this trial and the importance of sharing the science of COVID-19 and the drug, internationally, we expect to produce reports to the UK Government and regulatory agency more frequently upon request.

For peer review only - http://bmjopen.bmj.com/site/about/guidelines.xhtml
© Copyright: The University of Oxford and Oxford University Hospitals NHS Foundation Trust 2019

5. STATISTICS

5.1 Master Statistical Analysis Plan (M-SAP)

Details of the statistical design and methods for both the main trial and the virology substudy will be described in a Master Statistical Analysis Plan (M-SAP).

PANORAMIC will begin as a two arm, 1:1 randomised trial but will have the capability to add additional interventions over time. The evaluation of any new interventions will be governed by this master protocol and M-SAP (including adaptive algorithm and decision criteria), with any planned deviations from the master protocol and M-SAP to be specified in arm-specific appendices. The inclusion of any new interventions will require additional arm-specific appendices to the master protocol and M-SAP and will be implemented as a substantial amendment to regulatory bodies.

5.2 Open platform trial

5.2.1 Primary efficacy endpoints and analyses

The primary efficacy endpoint is all-cause, non-elective hospitalisation and/or death within 28 days of randomisation ascertained through patient/trial partner report, and/or patient medical records.

5.2.2 Primary efficacy hypothesis & analysis

Let p_j denote the probability of hospitalisation/death for persons in treatment group j, where j = 0 denotes the Usual Care arm. A Bayesian posterior distribution will be derived for the estimated difference in probability of hospitalisation/death between treatment groups. Let ϑ_j denote the log odds ratio of hospitalisation/death comparing intervention j to Usual Care. The primary analysis for intervention j will test the following hypothesis:

$$H_0 : \theta_j \geq 0$$
$$H_1 : \theta_j < 0$$

If the Bayesian posterior probability of beneficial treatment effect (alternative hypothesis) is greater than or equal to a pre-specified threshold (e.g., 0.98), the null hypothesis will be rejected, and the intervention will be deemed superior to Usual Care with respect to Hospitalisation/Death in the primary analysis population. The exact threshold will be pre-specified and calibrated via simulation in the Adaptive Design Report to demonstrate control of Type I error at the traditional 0.05 two-sided level for each intervention, accounting for multiple interim analyses.

The analysis of primary and some secondary outcome data analysis will be performed by Berry Consultancy with support from statisticians at the University of Oxford. The company is based in the USA; however, no identifiable data will be given to them during this process.

5.2.3 Adaptive design

The pre-specified design will allow adaptations to the trial based on the observed primary endpoint data. These adaptations include the declaration of success or futility of an intervention at an interim analysis and the removal of treatment arms based on pre-specified decision criteria. The adaptive algorithm will be documented in the Adaptive Design Report, including pre-specified criteria for decisions regarding futility or effectiveness of interventions and/or replacing interventions in the trial.

For peer review only - http://bmjopen.bmj.com/site/about/guidelines.xhtml
© Copyright: The University of Oxford and Oxford University Hospitals NHS Foundation Trust 2019

NUFFIELD DEPARTMENT OF PRIMARY CARE HEALTH SCIENCES | Primary Care Clinical Trials Unit | PANORAMIC Platform Adaptive trial of NOvel antiviRals for eArly treatMent of COVID-19 In the Community

The Adaptive Design Report (ADR) will contain extensive simulations to explore the performance of the adaptive design, including power and Type I error. Due to the urgent nature of the pandemic situation, this comprehensive ADR will be developed and finalised prior to the first scheduled interim analysis by a blinded statistician.

5.2.4 Interim analyses

Precise timing of the first interim analysis and frequency of subsequent interim analyses will be pre-specified in the Adaptive Design Report and DSMC Charter, based on both simulations and logistical considerations.

5.2.5 Allocation & adaptive randomisation

Participants will be randomised to one trial arm using fixed equal allocation ratios corresponding to the number of eligible arms in the trial. For instance, if there are two active interventions (A & B), the allocation ratio will be 1:1:1 for Usual Care, active A, active B (respectively), such that 33% of participants are randomised to Usual Care. If there are 3 active interventions, the allocation ratio will be 1:1:1:1, such that 25% of participants are randomised to Usual Care. As this is a nationwide, individually randomised trial that aims to include large numbers of participants, individual participant characteristics and infecting strain types of the infecting agent should be equally distributed between trial arms.

5.2.6 Sample size justification

Main Trial

The primary analysis will incorporate Bayesian logistic regression to estimate the odds ratio for hospitalisation/death for a treatment arm versus control, adjusting for age, vaccination status, and comorbidity status. An experimental treatment will be considered superior to the control if the Bayesian posterior probability of benefit is greater than a pre-specified threshold (e.g., 0.98) as detailed in the Adaptive Design Report. The trial design will incorporate multiple interim analyses that allow each intervention to stop early for futility, stop early for superiority, or continue to randomise participants. Additional interventions may be added as appendices to the master protocol throughout the duration of the trial. Extensive simulations will be conducted to evaluate and understand the operating characteristics and performance of the adaptive algorithm, such as control of Type I error and stopping guidance for efficacy and futility. Type I error will be controlled at the traditional 0.05 two-sided level for each intervention. A statistical analysis plan will be prepared and finalised before the first scheduled interim analysis.

The primary analysis will include those allocated to a particular antiviral agent and to the control condition (Usual Care) only during the period that that antiviral agent was in the trial (concurrently randomised population). A sensitivity analysis of the effect of subsequently introduced agents will include relevant control participants recruited prior to the introduction of that agent. To account for changes in the standard treatment in the Usual Care arm in this sensitivity analysis, and in changing patterns of recovery due to possible new variants, immunisations, behavioural interventions and other factors, this analytic model will include parameters to adjust for this temporal drift in the trial population, by estimating the primary endpoint in the usual care group across time via Bayesian hierarchical modelling.

For peer review only - http://bmjopen.bmj.com/site/about/guidelines.xhtml

© Copyright: The University of Oxford and Oxford University Hospitals NHS Foundation Trust 2019

For peer review only

Should an intervention demonstrate superiority versus Usual Care, the superior intervention may become included in Usual Care and so become part of the control arm for subsequent interventions. Additionally, the Bayesian secondary analysis model will provide “bridging” across overlapping treatment groups through the temporal parameters, which will enable comparisons of subsequent interventions to the original Usual Care, even if there are no concurrent randomisations to the original Usual Care.

If there are important changes in Usual Care due to the introduction of new and superior interventions, the Trial Management Group will assess whether any design feature (such as futility and superiority criteria) need to be re-considered.

We estimated that the hospitalisation/date rate will be reduced to 3% in the Usual Care arm.

Based on the unblinded data from the PRINCIPLE Trial that the overall estimated hospitalisation/death was 8.8% in the Usual Care arm for the period that Budesonide was open for recruitment. However, the percentage of fully vaccinated participants was lower than the current percentage. Subsequent blinded data from PRINCIPLE has observed the overall COVID-19 related hospitalisation/death was 3.8% between 27 May 2021 and 25 July 2021 (8, 9). So, we believe our estimated based rate is not overly overestimated for the primary outcome defined as all-cause hospitalisation/death. Although vaccine has been efficacious on preventing hospitalisation, there is still a sub-population of unvaccinated cohort that is at higher risk of hospital admission/death. The adaptive nature of the platform trial means that the recruitment will continue until a pre-specified probability of superiority or futility thresholds is met.

An estimated maximum of approximately 5300 participants per arm will be required to provide approximately 90% power for detecting a 33% relative reduction in the hospitalisation/death in an experimental arm relative to Usual Care, based on the assumption of an underlying 3% combined hospitalisation/death rate in the Usual Care arm, and an intervention lowering the hospitalisation/death rate to 2%. We expect fewer participants will be needed to detect the same relative reduction if the event rate is larger than 3% in the Usual Care arm (Table 1), or if there is a greater reduction in the relative risk of hospitalisation/death for a given intervention. However, should the event rate be lower than expected, then the target sample size will be increased to reflect this.

Table 1: Power and sample size estimates for PANORAMIC per treatment arm

**90% power**			**80% power**		
**Usual Care**	**Treatment**	**Sample size**	**Usual Care**	**Treatment**	**Sample size**
1.0%	0.67%	16578	1.0%	0.67%	12534
1.5%	1.0%	10771	1.5%	1.0%	8145
2.0%	1.3%	7241	2.0%	1.3%	5480
3.0%	2.0%	5319	3.0%	2.0%	4023
4.0%	2.7%	4177	4.0%	2.7%	3159
5.0%	3.4%	3425	5.0%	3.4%	2590

For peer review only - http://bmjopen.bmj.com/site/about/guidelines.xhtml
© Copyright: The University of Oxford and Oxford University Hospitals NHS Foundation Trust 2019

Simulations are used to further quantify the statistical power for each experimental arm in the context of an adaptive design, as well as general performance characteristics, as detailed in the Adaptive Design Report.

Virology Sampled Cohort

Simulations from a viral dynamic model from early 2020 [10] suggests that 30 patients per arm will detect a 2.5-fold increase in viral clearance (undetectable viral load at day 7) in patients who start therapy within 5 days of symptom onset (90% power; alpha 0.05). Clinical improvement may be associated with smaller decreases in viral load, and viral dynamic modelling leveraging time series viral load data can detect much smaller drug effect sizes [11]. 300 patients will provide a 95% probability of seeing at least one example of a mutation occurring in 1% or more of patients.

5.2.7 Virtual trial simulations

Virtual trial simulations are used to demonstrate good performance and adequate control of Type I error for the adaptive design. Simulations will be provided in the Adaptive Design Report.

5.2.8 Procedure for accounting for missing, unused, and spurious data

Full details of handling missing data will be specified in the M-SAP.

5.3 Primary analysis population

For each intervention, the primary analysis population will include all concurrently randomised patients that were eligible to be randomised to the intervention (concurrent and eligible) and Usual Care. The primary analysis will use trial participants who fulfil the eligibility criteria and have had the opportunity to complete 28 days of follow-up. Eligible participants will be analysed according to the group they were randomised to regardless of deviation from the protocol. All other analysis populations will be defined in the M-SAP.

Complier Average Causal Effect (CACE) modelling will be undertaken to account for adherence.

5.4 Procedures for reporting unplanned deviation(s) from the M-SAP

Analyses will be carried out in accordance with the M-SAP and corresponding appendices. Any additional analysis that is not specified in the M-SAP/appendices or any unplanned deviation(s) from the M-SAP/appendices will be specified in the Statistical Analysis Report. Reasons for these changes will be documented and authorised by the CI.

6. DATA MANAGEMENT

The data management aspects of the trial are summarised here with details fully described in the Data Management Plan.

6.1 Source data

Source documents are where data are first recorded. These include, but are not limited to, hospital/medical records (from which medical history and previous and concurrent medication may be summarised into the CRF), NHS Digital, eDRIS, SAIL and HSC NI data, clinical and office charts, laboratory and pharmacy records, diaries, microfiches, radiographs, and correspondence.

For peer review only

For peer review only - http://bmjopen.bmj.com/site/about/guidelines.xhtml
© Copyright: The University of Oxford and Oxford University Hospitals NHS Foundation Trust 2019

If a participant fails to complete data online and after six attempts at contacting the participant/Trial Partner, any sources of routinely collected data may be utilised to obtain missing data. Data collected will include participant identifiable information and will be accessed at the University of Oxford according to PC-CTU Information Governance policies and UK GDPR. Data will only be held for the duration it is required; this will be reviewed annually.

CRF entries will be considered source data if the CRF is the site of the original recording (e.g., there is no other written or electronic record of data). All documents will be stored safely in confidential conditions. On all trial-specific documents, other than the signed consent, the participant will be referred to by the trial participant number/code, not by name.

6.2 Access to data

Direct access will be granted to authorised representatives from the Sponsor, host institution, centres in other UK Devolved Administrations and the regulatory authorities to permit trial-related monitoring, audits, and inspections.

6.3 Data recording and record keeping

The Investigators will maintain appropriate medical and research records for this trial, in compliance with the requirements of the Medicines for Human Use (Clinical Trial) Regulations 2004, ICH E6 GCP and regulatory and institutional requirements for the protection of confidentiality of volunteers. The CI, PI, Co-Investigators, clinical team, including Clinical Research Nurses, and other authorised members of the trial team will have access to records. The Investigators will permit authorised representatives of the sponsor, and regulatory agencies to examine (and when required by applicable law, to copy) clinical records for the purposes of quality assurance reviews, audits and evaluation of the trial safety and progress.

The data will be entered into CRFs in an electronic format by the participant, trial Partner, Hub team member or trial team using an FDA part 11B compliant database. Data is stored on a secure cloud hosted server physically located in London, UK. Data will be entered in a web browser and then transferred to the database by encrypted (Https) transfer. This includes safety data, laboratory data and outcome data. Safety data will be collected through electronic diaries. Risks are mitigated using the ISO97001 framework.

An online secure data entry system designed to collect sensitive data, such as participant and Trial Partner contact details, will be used. All identifiable participant data is encrypted using the Advanced Encryption Standard. The participant portal will also manage online eligibility, eConsent and ePRO. Participant and Trial Partner data will be kept and stored securely for as long as it's required by the trial and reviewed on annual basis.

7. QUALITY ASSURANCE PROCEDURES

The trial will be conducted in accordance with the current approved protocol, GCP, relevant regulations and PC-CTU Standard Operating Procedures. All PIs, coordinating centre staff and site staff will receive training in trial procedures according to GCP where required. Regular monitoring will be performed according to GCP using a risk-based approach. Data will be evaluated for compliance with the protocol and accuracy in relation to source documents where possible.

For peer review only - http://bmjopen.bmj.com/site/about/guidelines.xhtml
© Copyright: The University of Oxford and Oxford University Hospitals NHS Foundation Trust 2019

The PC-CTU Trial Management Group will be responsible for the monitoring of all aspects of the trial's conduct and progress and will ensure that the protocol is adhered to, and that appropriate action is taken to safeguard participants and the quality of the trial itself. The TMG will be comprised of individuals responsible for the trial's day to day management and will meet regularly throughout the course of the trial.

7.1 Risk assessment and monitoring

A risk assessment and monitoring plan will be prepared before the trial opens for each antiviral agent and will be reviewed as necessary over the course of the trial to reflect significant changes to the protocol or outcomes of monitoring activities. Monitoring will be performed by the PC-CTU Quality Assurance Manager or delegate. The level of monitoring required will be informed by the risk assessment.

7.2 Trial committees

The composition, roles and responsibilities of committee are detailed in their respective charters except for the core project team and AT however their basic functions are as follows:

- Data and Safety Monitoring Committee (DSMC) will review the data received from the SAC at each interim analysis as described in the Statistical Analysis section, in order to ensure that the process is working correctly and to review and monitor the accruing data to ensure the rights, safety and wellbeing of the trial participants. Composition, and roles and responsibilities of the DSMC are detailed in the DSMC charter. The DSMC reviews data from interim analyses and makes recommendations to the TSC about antiviral agent s that have reached pre-specified thresholds for futility, success, or for which safety concerns have emerged
- Trial Steering Committee (TSC) will ensure the rights, safety, and wellbeing of the trial participants. They will make recommendations about how the trial is operating, any ethical or safety issues and any data being produced from other relevant studies that might impact the trial. Composition, and roles and responsibilities of the TSC are detailed in the TSC charter. The TSC advises the TMG about the conduct of the trial and stopping randomisation to trial arms (based on recommendations received from the DSMC and/or relevant information external to the trial), and the addition of new trial arms
- The Statistical Analysis Committee (SAC) will perform interim analysis and report these to the DSMC. The TMG will remain blind to these interim analyses until a recommendation is received form the TSC about stopping randomisation or safety concerns.
- Enhanced Safety Group (ESG) will review accumulating safety data in accordance with the ISA for each antiviral. The ESG will also provide advice and guidance to the relevant trial committees regarding the safety monitoring requirements for antiviral agents depending on their known safety profile
- Trial Management Group (TMG) – will be responsible for the day-to-day running of the trial, including monitoring all aspects of the trial and ensuring that the protocol is being adhered to. It will include Co-Investigators and will meet weekly in the first instance
- A project team from within the TMG will meet weekly or as required for operational decision making (meet daily at the start of the trial)
- The AT will advise on the antiviral agents to be included in the PANORAMIC trial

For peer review only - http://bmjopen.bmj.com/site/about/guidelines.xhtml
© Copyright: The University of Oxford and Oxford University Hospitals NHS Foundation Trust 2019

8. PROTOCOL DEVIATIONS

A trial related deviation is a departure from the ethically approved trial protocol or other trial document or process (e.g., consent process or administration of trial intervention) or from Good Clinical Practice (GCP) or any applicable regulatory requirements. Any deviations from the protocol will be documented in a protocol deviation form and filed in the trial master file.

A PC-CTU SOP is in place describing the procedure for identifying non-compliances, escalation to the central team and assessment of whether a non-compliance /deviation may be a potential Serious Breach.

9. SERIOUS BREACHES

A "serious breach" is a breach of the protocol or of the conditions or principles of Good Clinical Practice which is likely to affect to a significant degree:

(a) the safety or physical or mental integrity of the trial subjects; or
(b) the scientific value of the research.

In the event that a serious breach is suspected the Sponsor must be contacted within one working day. In collaboration with the CI, the serious breach will be reviewed by the Sponsor and, if appropriate, the Sponsor will report it to the approving REC committee and the relevant NHS host organisation within seven calendar days.

10. ETHICAL AND REGULATORY CONSIDERATIONS

10.1 Declaration of Helsinki

The Investigators will ensure that this trial is conducted in accordance with the principles of the Declaration of Helsinki.

10.2 Guidelines for Good Clinical Practice

The Investigators will ensure that this trial is conducted in accordance with relevant regulations and with Good Clinical Practice.

10.3 Approvals

Following Sponsor approval, the protocol, informed consent form, participant information sheets and any proposed informing material will be submitted to an appropriate Research Ethics Committee (REC), regulatory authorities, and host institution(s) for written approval. The PI and coordinating centres for each country will ensure and confirm correct regulatory approvals are gained prior to recruitment.

The Investigator will submit and, where necessary, obtain approval from the above parties for all substantial amendments to the original approved documents.

For peer review only

For peer review only - http://bmjopen.bmj.com/site/about/guidelines.xhtml
© Copyright: The University of Oxford and Oxford University Hospitals NHS Foundation Trust 2019

NUFFIELD DEPARTMENT OF PRIMARY CARE HEALTH SCIENCES Primary Care Clinical Trials Unit

PANORAMIC Platform Adaptive trial of NOvel antiviRals for eArly treatMent of COVID-19 In the Community

10.4 Other ethical considerations

If a particular arm is deemed futile and dropped, no further participants will be randomised to this arm and anyone who is currently on this arm will be informed it has been dropped.
Once a particular intervention has been declared superior and effective, that may become the comparator arm (i.e., standard care).

Participants who lack capacity to consent for themselves will only be recruited after consultation with their legal representative. Any sign of dissent in any form from the participant who lacks capacity to consent for themselves will be taken as an indication they do not wish to be involved and they will be withdrawn. Only residents of care homes who lack capacity to consent will be recruited, adults who lack capacity to consent will not be recruited from the wider community.

10.5 Reporting

The CI shall submit once a year throughout the clinical trial, or on request, an Annual Progress Report to the REC, HRA (where required), host organisation, funder (where required) and Sponsor. In addition, an End of Trial notification and final report will be submitted to the MHRA, the REC, host organisation and Sponsor.

10.6 Transparency in research

Prior to the recruitment of the first participant, the trial will have been registered on the ISRCTN Database. Results will be uploaded to this register within 12 months of the end of trial date as given on the end of trial declaration by the CI or their delegate. Where the trial has been registered on multiple public platforms, the trial information will be kept up to date during the trial, and the CI or their delegate will upload results to all those public registries within 12 months of the end of the trial declaration.

10.7 Participant confidentiality

The trial will comply with the UK General Data Protection Regulation (GDPR) and Data Protection Act 2018, which require data to be anonymised as soon as it is practical to do so. The processing of the personal data of participants will be minimised by making use of a unique participant trial number only on all trial documents and any electronic database(s). All documents will be stored securely and only accessible by trial staff and authorised personnel. The trial staff will safeguard the privacy of participants' personal data.

10.8 Expenses and benefits

All participants will be reimbursed with a £10 voucher as a token of recognition of giving their time and contribution to the trial. There will be no prescription charges for trial antiviral agents incurred by trial participants.

11. FINANCE AND INSURANCE

11.1 Funding

The trial is funded by the Department of Health and Social Care and the NIHR.
The Department of Health and Social Care will provide the antiviral agents to be evaluated in the trial without cost to the trial budget for trial use.

11.2 Insurance

For peer review only - http://bmjopen.bmj.com/site/about/guidelines.xhtml
© Copyright: The University of Oxford and Oxford University Hospitals NHS Foundation Trust 2019

NUFFIELD DEPARTMENT OF PRIMARY CARE HEALTH SCIENCES Primary Care Clinical Trials Unit PANORAMIC Platform Adaptive trial of NOvel antiviRals for eArly treatMent of COVID-19 In the Community

The University has a specialist insurance policy in place, which would operate in the event of any participant suffering harm as a result of their involvement in the research (Newline Underwriting Management Ltd, at Lloyd's of London). NHS indemnity operates in respect of the clinical treatment that is provided.

11.3 Contractual arrangements

Appropriate contractual arrangements will be put in place with all third parties.

12. PUBLICATION POLICY

The Investigators (those listed on the protocol and others to be decided at publication) will be involved in reviewing drafts of the manuscripts, abstracts, press releases and any other publications arising from the trial. Authors will acknowledge the trial funders. Authorship will be determined in accordance with the ICMJE guidelines and other contributors will be acknowledged.

13. DEVELOPMENT OF A NEW PRODUCT/ PROCESS OR THE GENERATION OF INTELLECTUAL PROPERTY

Ownership of IP generated by employees of the University vests in the University. The University will ensure appropriate arrangements are in place as regards any new IP arising from the trial.

14. ARCHIVING

Archiving will be done according to PC-CTU SOP and trial specific working instructions. Research documents with personal information, such as consent forms, will be held securely at the University of Oxford's archiving facility according to the PC-CTU Archiving SOP.

For peer review only - http://bmjopen.bmj.com/site/about/guidelines.xhtml
© Copyright: The University of Oxford and Oxford University Hospitals NHS Foundation Trust 2019

NUFFIELD DEPARTMENT OF PRIMARY CARE HEALTH SCIENCES
Primary Care Clinical Trials Unit
PANORAMIC Platform Adaptive trial of NOvel antiviRals for eArly treatMent of COVID-19 In the Community

For peer review only

For peer review only - http://bmjopen.bmj.com/site/about/guidelines.xhtml
© Copyright: The University of Oxford and Oxford University Hospitals NHS Foundation Trust 2019

NUFFIELD DEPARTMENT OF PRIMARY CARE HEALTH SCIENCES
Primary Care Clinical Trials Unit

PANORAMIC
Platform Adaptive trial of NOvel antiviRals for eArly treatMent of COVID-19 In the Community

15. APPENDIX A: SCHEDULE OF PROCEDURES

Main Trial

Procedures	Day 0	Day 0	Day 0	Day 1	Day 2	Day 0	Day 5	Daily Diary 1-28 and 3 and 6 months	Day 0 -12 months	Up to 10 years
	Screening completed by participant online/ phone	Baseline completed by participant online/ phone	Re-affirm consent and Eligibility completed by Clinician online/ phone	Telephone call: confirm receipt of participant pack	Telephone call to all participants	Antivirals requiring face-to-face recruitment (As defined in its ISA)		Symptom Diaries completed by participant online/ phone	Retrospective data collection by trial team	Data extraction from routine clinical records
						Screening/Baseline by Clinician face to face	Safety Review by Clinician face to face			
Informed consent	X	X	X			X	X	X		
Questionnaire	X	X						X		
Pregnancy test confirmation				X	X			X*		
Demographics	X	X				X			X	
Medical history	X	X	X			X			X	

For peer review only

For peer review only - http://bmjopen.bmj.com/site/about/guidelines.xhtml
© Copyright: The University of Oxford and Oxford University Hospitals NHS Foundation Trust 2019

Physical examination						X	X			
Concomitant medications		X	X			X		X**	X	
Vital signs measurements (if specified in ISA)						X				
Eligibility assessment	X		X			X				
Randomisation			X			X				
Dispensing of trial drugs			X			X				
Administer drug in clinic						X				
Post drug observation (for high-risk antivirals)						X				
Compliance								X		
Primary endpoint and secondary outcomes								X	X	X
AE assessments					X	X	X	X		
Safety bloods						X	X			
Evidence of sequalae and health care utilisation										X

* Days 1-3 only ** Daily symptom diaries will collect information on concomitant medications as specified in the antiviral ISA

For peer review only

For peer review only - http://bmjopen.bmj.com/site/about/guidelines.xhtml
© Copyright: The University of Oxford and Oxford University Hospitals NHS Foundation Trust 2019

Virology Sampled Cohort (as well as procedures described for the main trial and only for the first 300 patients who consent for this cohort in **each** arm of the trial)

	Baseline (Day 1, before first dose)	Day 2	Day 3	Day 4	Day 5	Day 6	Day 7	Day 14
**First 30 intensive sampled cohort participants****								
Virology sampling (nasopharyngeal swabs) at Hub or home	X	X	X	X	X	X	X	X*
Finger prick antibody test	X				X			X*
**Next viral 270 less intensive sampled cohort participants*****								
Virology sampling (nasopharyngeal swab sample (self-swab)	X				X*			X*
Finger prick antibody test	X				X*			X*

* +/- 1 day from randomisation ** To be evaluable for the intensive sampled cohort participants must return: i) a minimum of three nasopharyngeal swabs on Day 1, Day 4 and Day 7 and two finger prick blood tests on Day 1 and either Day 5 or Day 15. ***To be evaluable for the less intensive sampled cohort participants must return a minimum of two nasopharyngeal swabs on Day 1 and either the Day 5 or Day 14 and two finger prick blood tests on Day 1 and either Day 5 or Day 15.

For peer review only

For peer review only - http://bmjopen.bmj.com/site/about/guidelines.xhtml
© Copyright: The University of Oxford and Oxford University Hospitals NHS Foundation Trust 2019

16. APPENDIX B: Participant Flow Diagram

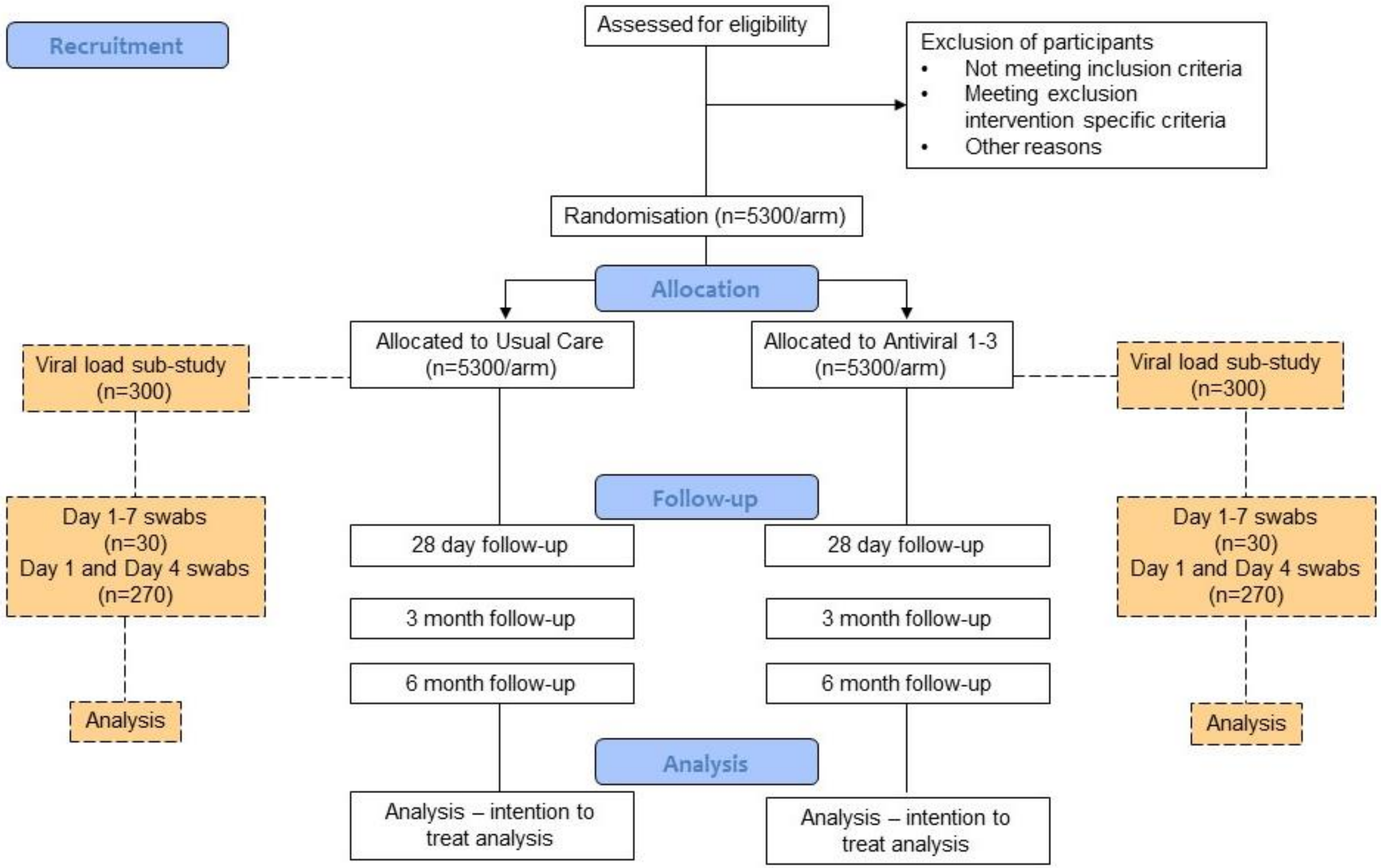

For peer review only - http://bmjopen.bmj.com/site/about/guidelines.xhtml

© Copyright: The University of Oxford and Oxford University Hospitals NHS Foundation Trust 2019

NUFFIELD DEPARTMENT OF PRIMARY CARE HEALTH SCIENCES
Primary Care Clinical Trials Unit
PANORAMIC
Platform Adaptive trial of NOvel antiviRals for eArly treatMent of COVID-19 In the Community

17. APPENDIX C: AMENDMENT HISTORY

Amendment No.	Protocol Version No.	Date issued	Author(s) of changes	Details of Changes made
Initial REC/MHRA submission	1.1	11/11/2021	Mina Davoudianfar	Replaced the word 'tablets' with 'capsules' in Molnupiravir ISA.
Initial REC/MHRA submission	1.2	18/11/2021	Mina Davoudianfar	Changes made in response to comments from REC review:<br>Removal of wording which allows recruitment of patients who lack capacity to consent, in a care home only.<br>Clarification of Day 1 and Day 2 phone calls.
Non-Substantial Amendment 1	1.3	24/11/2022	Mina Davoudianfar	Reinstated wording to include participants lacking capacity, to only be recruited from care homes, following request of Sponsor.
Substantial Amendment 1	1.4	17/01/2022	Tracie Madden	Changed health care providers to health service providers including government agencies e.g., UK Health Security Agency.
Substantial Amendment 2	2.0	02/03/2022	Tracie Madden	Added the Virology Sampled Cohort including sample processing and labelling requirements.
				Added Paxlovid as a new intervention.
				Updated information on contraception, following discussions with MHRA.
				Added that informed consent can be taken by a prescribing pharmacist, if specified in the relevant ISA.
				Provided clarification around change to the professional roles that each

For peer review only - http://bmjopen.bmj.com/site/about/guidelines.xhtml
© Copyright: The University of Oxford and Oxford University Hospitals NHS Foundation Trust 2019

NUFFIELD DEPARTMENT OF PRIMARY CARE HEALTH SCIENCES | Primary Care Clinical Trials Unit | PANORAMIC Platform Adaptive trial of NOvel antiviRals for eArly treatMent of COVID-19 In the Community

				HCP (medically qualified clinicians, research nurses and prescribing pharmacists) can have with respect to assessing participant eligibility for randomisation to antiviral agents.
				Amended follow-up and medication adherence sections to reflect the fact that the Hubs are now recruiting.
				Added that informed consent will be sought from participants partner to collect pregnancy follow-up data.
				Added details for the members of trial oversight committees and referenced the committee charters where appropriate.
				Updated the sample size justification in case of a lower than anticipated event rate.
				Revised the definition of the primary analysis population and secondary outcome measures for clarity.
				Updated the participant flow diagram to reflect inclusion of the Virology Sampled Cohort.
				Added in Lateral Flow Test as an alternative to PCR for trial entry and removed the requirement for a confirmatory PCR test for participants to be included in the main analysis.
				Added two new co-investigators.
				Added in a statement to reflect that the main PIS has been edited to highlight to

For peer review only

For peer review only - http://bmjopen.bmj.com/site/about/guidelines.xhtml
© Copyright: The University of Oxford and Oxford University Hospitals NHS Foundation Trust 2019

NUFFIELD DEPARTMENT OF PRIMARY CARE HEALTH SCIENCES
Primary Care Clinical Trials Unit
PANORAMIC
Platform Adaptive trial of NOvel antiviRals for eArly treatMent of COVID-19 In the Community

				potential participants, eligible for direct access to antivirals, that they can receive antiviral treatments out with the trial. DHSC approved table of potentially eligible cohorts added.
				Performed minor text corrections throughout.
				Added in details and function of ESG.
Substantial Amendment 3	3.0	25/03/22	Tracie Madden	Updated RSI and Paxlovid ISA, at the request of the MHRA, to reflect new information in the Paxlovid SmPC updated on 02/03/22.
				Inserted a statement, at the request of the MHRA, to state that a protocol substantial amendment will be required to be submitted for regulatory approval when List B in the Paxlovid ISA is modified.
				Replaced reference to access to a participants Summary Care Record including medication list as being sufficient to assess eligibility for entry into the Paxlovid trial arm with access primary care record.
				Updated references 21 and 22.
				Updated date of Molnupiravir RSI.
				Updated the risk mitigation strategies for drug interactions and side effect monitoring in the Paxlovid ISA at the request of the MHRA.
				Inserted Appendix F: standard script for safety monitoring of drugs that require adjustment when

For peer review only

For peer review only - http://bmjopen.bmj.com/site/about/guidelines.xhtml
© Copyright: The University of Oxford and Oxford University Hospitals NHS Foundation Trust 2019

NUFFIELD DEPARTMENT OF PRIMARY CARE HEALTH SCIENCES Primary Care Clinical Trials Unit PANORAMIC Platform Adaptive trial of NOvel antiviRals for eArly treatMent of COVID-19 In the Community

				co-administered with Paxlovid at the request of the MHRA.
				Updated safety monitoring procedure for overdose in the Molnupiravir and Paxlovid ISAs at the request of the ESG.
				Updated AE reporting sections in the Molnupiravir and Paxlovid ISAs at the request of the ESG.
				Performed minor text corrections throughout.
				Appendix A: Schedule of Procedures updated to state that the daily symptom diary will collect information on concomitant medications as specified in the antiviral ISA.
				Removed website links to all RSI.
				Definitions of evaluable participants for the intensive and less intensive Virology sampled cohorts added to schedule of procedures for Virology sampled cohorts at request of TMG.
Substantial Amendment 4	4.0	20/04/2022	**Elizabeth Hadley**	Replacing a COPI notice used to recruit participants which expires in June 2022 with a CAG Approval.
Substantial Amendment 5	5.0	09/05/2022	**Julie Allen**	Updating of eligibility assessment for Paxlovid. To include information relating to standard prescribing practices across the UK, who can perform eligibility assessments and which medical records can be used. Update PPI Members. Update the use of national data collection agencies in all devolved nations.

For peer review only - http://bmjopen.bmj.com/site/about/guidelines.xhtml
© Copyright: The University of Oxford and Oxford University Hospitals NHS Foundation Trust 2019

NUFFIELD DEPARTMENT OF PRIMARY CARE HEALTH SCIENCES Primary Care Clinical Trials Unit
PANORAMIC Platform Adaptive trial of NOvel antiviRals for eArly treatMent of COVID-19 In the Community

Lists details of all protocol amendments whenever a new version of the protocol is produced.

Protocol amendments must be submitted to the funder and Sponsor for approval prior to submission to the REC committee, HRA (where required) and/or MHRA.

18. APPENDIX D: SUPPLEMENTARY MATERIAL

A. Abbreviations

AE	Adverse event
AR	Adverse reaction
AT	Antiviral Taskforce
CI	Chief Investigator
CRF	Case Report Form
CT	Clinical Trials
CTA	Clinical Trials Authorisation
DHSC	Department of Health and Social Care
DSMC	Data Monitoring Committee / Data and Safety Monitoring Committee
DSUR	Development Safety Update Report
eDRIS	Electronic Data Research and Innovation Service
ESG	Enhanced Safety Group
GCP	Good Clinical Practice
GPDPR	General Practice Data for planning and research
HSC NI	Health and Social Care Northern Ireland (HSC Business Services Organisation/HSC Trusts)
GP	General Practitioner
HRA	Health Research Authority
HCP	Healthcare Professional
IB	Investigators Brochure
ICF	Informed Consent Form
ICH	International Conference on Harmonisation
IMP	Investigational Medicinal Product
ISA	Intervention Specific Appendix
MHRA	Medicines and Healthcare products Regulatory Agency
NHS	National Health Service
NIHR	National Institute of Health Research
RES	Research Ethics Service
PI	Principal Investigator
PIS	Participant/ Patient Information Sheet
R&D	NHS Trust Research and Development Department
RCGP RSC	Royal College of General Practitioners Research Surveillance Centre

For peer review only - http://bmjopen.bmj.com/site/about/guidelines.xhtml
© Copyright: The University of Oxford and Oxford University Hospitals NHS Foundation Trust 2019

NUFFIELD DEPARTMENT OF PRIMARY CARE HEALTH SCIENCES Primary Care Clinical Trials Unit

PANORAMIC Platform Adaptive trial of NOvel antiviRals for eArly treatMent of COVID-19 In the Community

REC	Research Ethics Committee
RGEA	Research Governance, Ethics and Assurance
RSI	Reference Safety Information
SAE	Serious Adverse Event
SAIL	The Secure Anonymised Information Linkage Databank
SAR	Serious Adverse Reaction
SDV	Source Data Verification
SmPC	Summary of Medicinal Product Characteristics
SOP	Standard Operating Procedure
TSC	Trial Steering Committee
SUSAR	Suspected Unexpected Serious Adverse Reactions
TMF	Trial Master File
UCL	University College London
UKTIS	UK Teratology Service

B. Key trial contacts

**Chief Investigator**	Professor Chris Butler<br>Nuffield Department of Primary Care Health Sciences<br>Gibson Building<br>Radcliffe Observatory Quarter<br>Woodstock Road<br>Oxford<br>OX2 6GG<br>christopher.butler@phc.ox.ac.uk
**Sponsor**	Research Governance, Ethics and Assurance (RGEA)<br>Joint Research Office<br>1st floor, Boundary Brook House<br>Churchill Drive,<br>Headington<br>Oxford OX3 7GB<br>ctrg@admin.ox.ac.uk<br>Tel: +44 (0)1865616480
**Funder(s)**	UKRI/NIHR
**Clinical Trials Unit**	Primary Care Clinical Trials Unit,<br>Nuffield Department of Primary Care Health Sciences<br>Radcliffe Observatory Quarter<br>Woodstock Road<br>Oxford<br>OX2 6GG<br>panoramic@phc.ox.ac.uk<br>Tel: TBC
**Statistician**	Dr Ben Saville,<br>Berry Consultants,<br>Austin, Texas, USA,

For peer review only

For peer review only - http://bmjopen.bmj.com/site/about/guidelines.xhtml
© Copyright: The University of Oxford and Oxford University Hospitals NHS Foundation Trust 2019

	And<br>Department of Biostatistics,<br>Vanderbilt University School of Medicine,<br>Nashville, Tennessee, USA<br><br>Dr Ly-Mee Yu<br>Primary Care Clinical Trials Unit,<br>Nuffield Department of Primary Care Health Sciences<br>Radcliffe Observatory Quarter<br>Woodstock Road<br>Oxford<br>OX2 6GG
**Committees**	**DSMC Chair:**<br>Prof Deborah Ashby<br>Faculty of Medicine, School of Public Health<br>Imperial College London<br>deborah.ashby@imperial.ac.uk<br><br>**DSMC Members:**<br>Prof Simon Gates<br>Cancer Research Clinical Trials Unit (CRCTU)<br>Institute of Cancer and Genomic Sciences<br>University of Birmingham<br>S.Gates@bham.ac.uk<br><br>Prof Gordon Taylor<br>University of Exeter<br>g.j.taylor@exeter.ac.uk<br><br>Dr Benjamin Fisher<br>Institute of Inflammation and Ageing<br>University of Birmingham Research Labs<br>Queen Elizabeth Hospital<br>B.Fisher@bham.ac.uk<br><br>Prof Martin Underwood<br>Warwick Clinical Trials Unit<br>Warwick Medical School<br>The University of Warwick<br>M.Underwood@warwick.ac.uk
	**TSC Chair:**<br>Prof Philip Hannaford<br>University of Aberdeen<br>p.hannaford@abdn.ac.uk

For peer review only - http://bmjopen.bmj.com/site/about/guidelines.xhtml
© Copyright: The University of Oxford and Oxford University Hospitals NHS Foundation Trust 2019

NUFFIELD DEPARTMENT OF PRIMARY CARE HEALTH SCIENCES | Primary Care Clinical Trials Unit | PANORAMIC Platform Adaptive trial of NOvel antiviRals for eArly treatMent of COVID-19 In the Community

	**TSC Members:**<br>Prof Ranjit Lall<br>Warwick Clinical Trials Unit<br>Warwick Medical School<br>The University of Warwick<br>R.Lall@warwick.ac.uk<br><br>Prof Alastair Hay<br>Bristol Population Health Science Institute<br>Health Protection Research Unit (HPRU)<br>University of Bristol<br>alastair.hay@bristol.co.uk<br><br>Prof William Hollingworth<br>Bristol Population Health Science Institute<br>University of Bristol<br>William.Hollingworth@bristol.ac.uk<br><br>*PPI representatives*<br>Ms Corina Cheeks<br><br>**TSC Observers:**<br>Prof Matt Sydes<br>Institute of Clinical Trials and Methodology<br>90 High Holborn<br>2nd Floor<br>London<br><br>Prof Mike Moore<br>Primary Care Research Centre<br>Primary Care, Population Sciences and Medical Education (PPM)<br>Faculty of Medicine<br>University of Southampton
	**ESG Chair:**<br>Prof Najib Rahman<br>Oxford Respiratory Trials Unit<br>University of Oxford<br>najib.rahman@ndm.ox.ac.uk<br><br>**ESG Members:**<br>Professor Duncan Richards<br>Oxford Clinical Trial Research Unit<br>University of Oxford<br>duncan.richards@ndorms.ox.ac.uk<br><br>Professor Paramjit Gill<br>Department of Health Sciences<br>Warwick Medical School

For peer review only - http://bmjopen.bmj.com/site/about/guidelines.xhtml
© Copyright: The University of Oxford and Oxford University Hospitals NHS Foundation Trust 2019

NUFFIELD DEPARTMENT OF PRIMARY CARE HEALTH SCIENCES | Primary Care Clinical Trials Unit | PANORAMIC Platform Adaptive trial of NOvel antiviRals for eArly treatMent of COVID-19 In the Community

	P.Gill1@warwick.ac.uk<br><br>Professor Gail Hayward<br>Department of Primary Care Health Sciences<br>University of Oxford<br>gail.hayward@phc.ox.ac.uk<br><br>Professor Andrew Ustianowski<br>Infectious Disease and Tropical Medicine, Manchester University NHS Foundation Trust<br>North Manchester General Hospital<br>North Manchester Care Organisation (Part of the Northern Care Alliance NHS Group)<br>Delaunays Road<br>Manchester M8 5RB. UK.<br>Andrew.Ustianowski@mft.nhs.uk

For peer review only

For peer review only - http://bmjopen.bmj.com/site/about/guidelines.xhtml
© Copyright: The University of Oxford and Oxford University Hospitals NHS Foundation Trust 2019

NUFFIELD DEPARTMENT OF PRIMARY CARE HEALTH SCIENCES
Primary Care Clinical Trials Unit
PANORAMIC Platform Adaptive trial of NOvel antiviRals for eArly treatMent of COVID-19 In the Community

C. Objectives and outcome measures

	Objectives	Outcome Measures	Timepoint (s)
**Main Trial**			
**Primary**	To determine whether antiviral treatment in the community safely reduces non-elective hospitalisations/ deaths in higher risk, symptomatic patients with confirmed COVID-19	All cause, non-elective hospitalisation and/or death, within 28 days of randomisation	Within 28 days of randomisation Patient report, Trial Partner report, HES/ONS/medical record data linkage
**Secondary**	To explore whether antiviral treatment affects:<br>1) Time to recovery (defined as the first instance that a participant report of feeling recovered from the illness).<br>2) Participant reported illness severity, reported by daily rating of how well participant feels, enabling identification of sustained recovery.<br>3) Duration of severe symptoms and symptom recurrence including time to alleviation of symptoms, time to initial reduction of severity of symptoms, time to sustained recovery, time to sustained alleviation of symptoms, number of days of severe symptoms and	1-3 Participant reports symptoms daily for 28 days and at 3 and 6 months.	1-3 Daily online symptom scores. Telephone call or text on days 7, 14 and 28 if data is not obtained through the online diary. Also, at 3 and 6 months.

For peer review only - http://bmjopen.bmj.com/site/about/guidelines.xhtml
© Copyright: The University of Oxford and Oxford University Hospitals NHS Foundation Trust 2019

	worsening of symptoms.<br><br>4) Contacts with the health services.<br><br>5) New infections in household.<br><br>6) To investigate the safety of antiviral agents.<br><br>7) Longer term effects including proportion with long covid, long covid symptoms, health care use and wellness.<br><br>8) Cost effectiveness.	4) Contacts with health services reported by patients and/or captured by reports of patients' medical records.<br><br>5) Reports of new infections in the household from daily diary.<br><br>6) Evaluation of overall safety of drugs by the monitoring of AEs as defined in the ISAs).<br><br>7) Well-being, symptoms, and heath care utilisation.<br><br>8) Resource use and cost data and EQ-5D-5L.	4) GP notes review if available through Oxford RCGP RSC network; otherwise, other sources of routinely collected data after 28 days. Medical notes review for up to 10 years.<br><br>5) Daily online symptom scores or telephone call or text on days 7, 14 and 28.<br><br>6) For the duration of the antiviral course and a defined period after the antiviral finishes (see ISAs).<br><br>7) Patient contact at three and six months, electronic medical record search for up to one year.<br><br>8) Baseline and Day 28.
	**Objectives**	**Outcome Measures**	**Timepoint (s)**
**Virology Sampled Cohort**			

For peer review only

© Copyright: The University of Oxford and Oxford University Hospitals NHS Foundation Trust 2019

**Primary**	To determine whether antiviral treatment in the community reduces viral load to undetectable levels more quickly than untreated patients.	SARS-CoV-2 viral load.	Day 7.
**Secondary**	1) To determine whether antiviral treatment in the community leads to faster viral elimination rates than untreated patients.<br><br>2) To determine whether genetic mutations in the virus are more frequent in patients taking antiviral treatment compared with untreated patients.<br><br>3) To assess the impact of antibodies on viral load decline in patients taking antiviral treatment compared to with untreated patients.<br><br>4) To assess the antibody response on viral load decline in patients taking antiviral treatment compared with untreated patients.<br><br>5) (Exploratory endpoint) To compare viral load rate of decline in patients receiving	1) SARS-CoV-2 viral load.<br><br>2) SARS-CoV-2 viral genetic whole genome sequence.<br><br>3) SARS-CoV-2 viral load.<br><br>4) SARS-CoV-2 antibodies.<br><br>5) SARS-CoV-2 viral load.	1) Days 1-7, Day 14.<br><br>2) Day 1, Day 5, Day 14.<br><br>3) Day 1, Day 5, Day 14.<br><br>4) Day 1, Day 5, Day 14.<br><br>5) Days 1-7, Day 14.

For peer review only - http://bmjopen.bmj.com/site/about/guidelines.xhtml
© Copyright: The University of Oxford and Oxford University Hospitals NHS Foundation Trust 2019

	different antiviral therapies.		

D. Adverse Events

Definitions:	
**Adverse Event (AE)**	Any untoward medical occurrence in a participant to whom a medicinal product has been administered, including occurrences which are not necessarily caused by or related to that product.
**Adverse Reaction (AR)**	An untoward and unintended response in a participant to an investigational medicinal product which is related to any dose administered to that participant.<br>The phrase "response to an investigational medicinal product" means that a causal relationship between a trial medication and an AE is at least a reasonable possibility, i.e., the relationship cannot be ruled out.<br>All cases judged by either the reporting medically qualified professional or the Sponsor as having a reasonable suspected causal relationship to the trial medication qualify as adverse reactions.
**Serious Adverse Event (SAE)**	A SAE is any untoward medical occurrence that:<br>• results in death<br>• is life-threatening<br>• requires inpatient hospitalisation or prolongation of existing hospitalisation<br>• results in persistent or significant disability/incapacity<br>• consists of a congenital anomaly or birth defect*.<br>Other 'important medical events' may also be considered a SAE when, based upon appropriate medical judgement, the event may jeopardise the participant and may require medical or surgical intervention to prevent one of the outcomes listed above.<br>NOTE: The term "life-threatening" in the definition of "serious" refers to an event in which the participant was at risk of death at the time of the event; it does not refer to an event which hypothetically might have caused death if it were more severe.<br>*NOTE: Pregnancy is not, in itself an SAE. In the event that a participant or their partner becomes pregnant whilst taking part in a clinical trial or

For peer review only

For peer review only - http://bmjopen.bmj.com/site/about/guidelines.xhtml
© Copyright: The University of Oxford and Oxford University Hospitals NHS Foundation Trust 2019

NUFFIELD DEPARTMENT OF PRIMARY CARE HEALTH SCIENCES Primary Care Clinical Trials Unit

PANORAMIC Platform Adaptive trial of NOvel antiviRals for eArly treatMent of COVID-19 In the Community

	during a stage where the foetus could have been exposed to the medicinal product (in the case of the active substance or one of its metabolites having a long half-life) the pregnancy should be followed up by the investigator until delivery for congenital abnormality or birth defect, at which point it would fall within the definition of "serious".
**Serious Adverse Reaction (SAR)**	An AE that is both serious and, in the opinion of the reporting Investigator, believed with reasonable probability to be due to one of the trial antiviral agents, based on the information provided.
**Suspected Unexpected Serious Adverse Reaction (SUSAR)**	A SAR, the nature and severity of which is not consistent with the Reference Safety Information (RSI) for the medicinal product in question set out:<br>• in the case of a product with a marketing authorisation, in the approved summary of product characteristics (SmPC) for that product<br>• in the case of any other investigational medicinal product, in the approved investigator's brochure (IB) relating to the trial in question.

NB: To avoid confusion or misunderstanding the difference between the terms "serious" and "severe", the following note of clarification is provided: "Severe" is often used to describe intensity of a specific event, which may be of relatively minor medical significance. "Seriousness"

For peer review only

For peer review only - http://bmjopen.bmj.com/site/about/guidelines.xhtml
© Copyright: The University of Oxford and Oxford University Hospitals NHS Foundation Trust 2019

19. APPENDIX E: INTERVENTION SPECIFIC APPENDICES

1. USUAL CARE ARM

1. Background and rationale

This Usual Care arm will follow current NHS care provision and provides a control against which the effect of new interventions that are added to usual care can be assessed. If a new trial intervention plus Usual Care is found to be superior to Usual Care alone, then the Usual Care will evolve to include interventions that are recommended as part of standard care in the NHS. Usual Care in the trial will not be specified or mandated, and it will vary over time according to emerging evidence and evolving national recommendations and will be tailored by responsible clinicians to patient characteristics, clinical picture, and individual need. In addition, individual patients will vary in the self-care they choose to use, including use of over-the-counter medication. Use of key treatments such as monoclonal antibodies will be captured and considered in analyses.

2. Detail of intervention

Participants randomised to the usual care arm will receive usual clinical care as per NHS care delivery practice.

a. Investigational Medicinal Product (IMP) description

Not applicable

b. Storage of IMP

Not applicable

3. Safety reporting

Mechanisms for safety reporting are outlined in the trial protocol

For peer review only

For peer review only - http://bmjopen.bmj.com/site/about/guidelines.xhtml
© Copyright: The University of Oxford and Oxford University Hospitals NHS Foundation Trust 2019

2. USUAL CARE PLUS MOLNUPIRAVIR

1. Background

a. Potential mechanism of efficacy

Molnupiravir is an oral antiviral that was initially developed for treatment of influenza, but has now been developed for treatment and prevention of COVID-19.(12-14) It is a prodrug of the ribonucleoside analogue NHC that is incorporated into viral RNA by RNA-dependent RNA polymerase and inhibits viral replication by inducing *viral error catastrophe* (i.e. causing the build-up of viral mutations with each replication cycle that impair viral fitness).(14, 15)

b. Evidence for potential benefits of Molnupiravir in COVID-19 illness

Pre-clinical data

Molnupiravir has been shown *in vitro* to have a high barrier to resistance and to inhibit pathogenic coronaviruses (e.g., MERS-CoV, SARS-CoV-1, and SARS-CoV-2) (8). Data from mouse, (9) ferret (10) and Syrian hamster models (11) shows that Molnupiravir inhibits SARS-CoV-2 replication in vivo.

Phase 1 studies

A phase 1 trial among 130 healthy adults found that Molnupiravir was well tolerated with no signals of clinical concern. (12)

Phase 2/3 studies

As of 17-JUL-2020, 122 participants have received placebo or MK-4482 in single doses of 50 to 1600 mg or in multiple doses of 50 to 600 mg Q12H for 5.5 days. Molnupiravir was generally well tolerated in hospitalised and non-hospitalised participants. The proportion of participants with AEs, drug related AEs (per investigator), SAEs, and AEs leading to trial intervention discontinuation during the protocol-specified AE safety follow-up period were comparable across the intervention groups, with no apparent dose effect observed. One participant was discontinued from trial treatment because of a rash of moderate intensity, appearing following 3 days of dosing (6 doses) with 800 mg Q12H MK-4482 or placebo (blinded trial). No clinically meaningful trends were observed for changes in clinical laboratory values as a function of dose or treatment. In trial MK-4482-001 among hospitalised patients, there was a numerical imbalance in AEs resulting in death in participants treated with Molnupiravir (14/218, 6.4%) compared with placebo (2/75, 2.7%). However, Molnupiravir was well tolerated in both hospitalised (MK-4482-001) and non-hospitalised (MK-4482-002) participants with COVID-19, and there were no clinically meaningful differences in the incidence of AEs, SAEs, drug-related AEs, discontinuations due to AEs, and deaths observed when comparing Molnupiravir to placebo, and no evidence of a dose response relationship with Molnupiravir (see below). There was no apparent dose effect based on the incidence of death in each of the Molnupiravir groups. None of the deaths were considered related to trial intervention by the investigator, and most were associated with complications of COVID-19 or to secondary bacterial infections.

Virology data from the completed Phase 2 trial (MK-4482-006) in 204 non-hospitalised participants with COVID-19 have shown that treatment with Molnupiravir results in an antiviral effect, including reduction in viral load and in infectious virus as well as a higher percentage of

For peer review only - http://bmjopen.bmj.com/site/about/guidelines.xhtml
© Copyright: The University of Oxford and Oxford University Hospitals NHS Foundation Trust 2019

random mutations in viral RNA post treatment consistent with the mechanism of action (i.e., viral error catastrophe. (13)

Regarding disease progression, in the ongoing Phase 2/3 randomised, placebo-controlled, double-blind MK-4482-002 trial in non-hospitalised patients with COVID-19 (n=302), there was a consistent trend toward potential benefit from treatment with Molnupiravir early in the course of disease as well as in individuals with risk factors for severe illness from COVID-19. Interim analyses showed the following:

- Fewer participants in the combined Molnupiravir treatment groups (7/225, 3.1%) were hospitalised or died through Day 29 compared with participants in the placebo group (4/74, 5.4%) **
- While none of the comparisons reached statistical significance, the difference in the rate of death or hospitalisation favours Molnupiravir in all comparisons
- Most participants achieved sustained symptom improvement/resolution by Day 29, regardless of treatment received. However, confidence intervals were wide and did not provide clear evidence of treatment effect for time to progression or sustained improvement/resolution of COVID-19 signs and symptoms

*** A post-hoc analysis of the primary endpoint in the subgroup of participants who were randomised within 5 days of initial COVID-19 symptom onset and who had at least 1 risk factor for severe illness was also performed: 4/107 (3.7%) participants were hospitalised in the combined Molnupiravir groups compared with 4/34 (11.8%) participants in the placebo group representing an observed reduction in the relative risk for hospitalisation of 68%.*

A systematic review of early studies suggest benefit in terms of reduced hospital admissions.(16)

2. Detail of intervention

Participants randomised to the Usual Care plus Molnupiravir arm will receive Usual Care as per NHS guidelines, plus Molnupiravir for five days.

a. Precautions

No adverse drug reactions have been defined for Molnupiravir based on current data safety data from a Phase 1 trial (MK-4482-004) in 130 healthy participants who received single doses up to 1600 mg (including the food effect panel) and multiple doses up to 800 mg Q12H for 5.5 days indicate that Molnupiravir was generally well tolerated.(12) One participant discontinued from trial treatment because of a rash, appearing following 3 days of dosing with 800 mg Q12H Molnupiravir. This AE was rated as mild in intensity and considered by the investigator to be related to trial drug.

Safety data from Phase 2 studies show that all evaluated Molnupiravir doses were generally well tolerated in both hospitalised (MK-4482-001) and non-hospitalised (MK-4482-002) participants with COVID-19. No clinically meaningful differences in the incidence of AEs, SAEs, drug-related AEs, discontinuations due to AEs, and deaths were observed when comparing Molnupiravir to placebo, and no evidence of a dose response relationship with Molnupiravir.

For peer review only - http://bmjopen.bmj.com/site/about/guidelines.xhtml
© Copyright: The University of Oxford and Oxford University Hospitals NHS Foundation Trust 2019

There were no clinically meaningful trends for changes in liver enzymes or amylase and lipase as a function of either dose or treatment. Additionally, there were no meaningful abnormalities in haematological parameters as a function of either dose or treatment, and no evidence of changes relative to baseline in any haematological parameters over time in those treated with Molnupiravir compared with placebo through Day 29. Preliminary unblinded safety data from MK-4482-006 in non-hospitalised participants and blinded safety data from hospitalised participants in MK-4482-007 support the above safety conclusions. In MK-4482-001, there was a numerical imbalance in AEs resulting in death in hospitalised participants treated with Molnupiravir (14/218, 6.4%) compared with placebo (2/75, 2.7%). There was no apparent dose effect based on the incidence of death in each of the Molnupiravir groups. None of the deaths were considered related to trial intervention by the investigator, and most were associated with complications of COVID-19 or to secondary bacterial infections.

A dose-escalating, open-label, randomised-controlled (standard-of-care) Bayesian adaptive Phase I trial of adult outpatients with PCR-confirmed SARS-CoV-2 infection within 5 days of symptom onset randomised participants in 2:1 in groups of 6 participants to 300, 600 and 800mg doses of Molnupiravir orally, twice daily for 5 days or control. A dose was judged unsafe if the probability of 30% or greater dose-limiting toxicity (the primary outcome) over controls was 25% or greater. Secondary outcomes included safety, clinical progression, pharmacokinetics, and virological responses. Of 103 participants screened, 18 participants were enrolled between 17 July and 30 October 2020. Molnupiravir was well tolerated at 300, 600 and 800mg doses with no serious or severe AEs. Overall, 4 of 4 (100%), 4 of 4 (100%) and 1 of 4 (25%) of the participants receiving 300, 600 and 800mg Molnupiravir, respectively, and 5 of 6 (83%) controls, had at least one AE, all of which were mild (grade 2). The probability of 30% excess toxicity over controls at 800mg was estimated at 0.9%. They concluded that Molnupiravir was safe and well tolerated at a dose of 800mg twice daily for 5 days.(17)

b. Pregnancy and lactation

In the reproductive and developmental toxicity studies, there were no Molnupiravir-related effects on female or male fertility or early embryonic development up to the highest dose tested, 500 mg/kg/day (2.1/6.1-fold (female/male) the clinical NHC exposure at 800 mg Q12H). In pregnant rats dosed with Molnupiravir during the organogenesis period, developmental toxicity including embryo lethality (post implantation losses) and teratogenicity was observed at 1000 mg/kg/day (7.5-fold the clinical NHC exposure at 800 mg Q12H), and reduced fetal growth was noted at ≥500 mg/kg/day (2.9-fold the clinical NHC exposure at 800 mg Q12H).
There was no developmental toxicity at doses up to 250 mg/kg/day (0.8-fold the clinical NHC exposure at 800 mg Q12H). In pregnant rabbits, developmental toxicity was limited to reduced mean fetal body weights at 750 mg/kg/day (18-fold the clinical NHC exposure at 800 mg Q12H). There was no developmental toxicity in rabbits at up to 400 mg/kg/day (6.5-fold the clinical NHC exposure at 800 mg Q12H).

There are no human studies of its use among pregnant or lactating women.

Pregnancy (known or suspected) and breast-feeding are exclusions for the Molnupiravir arm of the trial based on the currently available data:

- Limited information on animal reproductive toxicity studies is provided in the SmPC
- There is evidence for the potential teratogenicity of Molnupiravir

For peer review only - http://bmjopen.bmj.com/site/about/guidelines.xhtml
© Copyright: The University of Oxford and Oxford University Hospitals NHS Foundation Trust 2019

- The effects of Molnupiravir on pregnant people are unknown

To mitigate the risk of pregnancy in the trial, all participants of child-bearing potential will be required to take a urine pregnancy test prior to commencing trial treatment. We will confirm a negative test result during the Day 1 or Day 2 telephone call with a member of the trial team (see section 2.8 of the master protocol for further information). Before starting the trial treatment, the clinician/research nurse will explain to the participant that pregnancy is an exclusion criterion and explain the contraception requirements during the trial. If the participant confirms that there is a possibility that they may be pregnant during this call, they will be excluded from taking part.

As per 'PC-CTU SOP TM119 Pharmacovigilance', any pregnancy that occurs during Molnupiravir (antiviral agent) administration requires monitoring and follow-up until the outcome of the pregnancy and any postnatal sequelae are known is known. The CI, PI or delegated individual will report any pregnancy occurring whilst in the trial to the PC-CTU. The Sponsor will report any pregnancy occurring whilst in the trial to the UK Teratology Information Service (UKTIS).

Participants themselves will be asked in their daily diaries or during the day 7, 14 and 28 phone calls, whether they have become pregnant since enrolling into the trial. These responses will be monitored daily and if a participant does become pregnant during the trial, the clinical team will inform them to immediately stop the medication. Consent to collect follow-up information regarding the outcome of the pregnancy and any postnatal sequelae in the infant will be sought from potential participants prior to trial entry. The CI or delegated individual will liaise with the relevant Obstetrician or equivalent HCP throughout the pregnancy until delivery to monitor for congenital abnormality or birth defect, at which the pregnancy would fall under the definition of serious and require reporting as an SAE.

The DSMC will be informed of any pregnancies in this treatment group, in weekly safety reports. Pregnancies and outcomes will be included in annual safety reports.

3. Trial visits

As per Master Protocol

4. Outcome measures

As per Master Protocol

5. Eligibility criteria (in addition to master protocol)

Inclusion criteria:

- Willingness to take a pregnancy test prior to starting Molnupiravir treatment (Participants of childbearing potential)

Exclusion criteria:

- Known or suspected pregnancy*
- Breastfeeding
    - Participants of childbearing potential (participants who are anatomically and physiologically capable of becoming pregnant), not willing to use effective

For peer review only - http://bmjopen.bmj.com/site/about/guidelines.xhtml
© Copyright: The University of Oxford and Oxford University Hospitals NHS Foundation Trust 2019

contraceptive** for 28-day duration of the trial, and who do not confirm a negative pregnancy test prior to starting the drug.

- Known allergy to Molnupiravir
- Currently taking Molnupiravir

* As recorded by the participant on the screening form and confirmed by interaction between clinician and participant, and the pregnancy test supplied by the trial.

** Effective methods include sterilisation, long-acting reversible contraceptive (LARC) methods (intrauterine devices and implants), combined hormonal methods (oral, transdermal, or intravaginal), or the progestogen only pill or injection. Participants will also be eligible if they have been abstinent for the 28 days before enrolling in the trial and will continue to be abstinent for the 28-day duration of follow-up where this is in line with the preferred and usual lifestyle of the subject.

Note: a barrier method on its own is not sufficient.

6. Professional role of those checking eligibility

To confirm that the participant meets the criteria defined above, information will be elicited through a direct discussion between a medically qualified professional , research nurse, nurse prescriber or a prescribing pharmacist, dependent on the ISA for the specific IMP involved and the participant. The participant can be randomised to Molnupiravir if any of these three categories of HCPs considers the potential participant is eligible.

7. Antiviral agent: Molnupiravir

a. Name

Lagevrio contains the active substance Molnupiravir. The drug will be referred to by the active substance only.

b. Dose

Molnupiravir 200 milligram (mg) capsules. The capsules are for oral administration. Four 200mg capsules (800mg) Molnupiravir to be taken every 12 hours (twice a day), for five days. This regimen was identified and found to be safe in a dose finding trial,(17) and has been used in a clinical trial in which early reports indicate was safe and efficacious.(18)

c. Common side effects

Common side effects, according to the SmPC, include dizziness, headache, diarrhoea, and nausea. These symptoms will be collected in daily diaries and calls on 7, 14 and 28 and will be monitored weekly by DSMC committee.

For peer review only - http://bmjopen.bmj.com/site/about/guidelines.xhtml
© Copyright: The University of Oxford and Oxford University Hospitals NHS Foundation Trust 2019

d. Concomitant medications

Molnupiravir has been found to lack inhibitory or inductive activity towards xenobiotic metabolic enzymes and transporters tested in vitro, suggesting that the potential for DDIs between Molnupiravir/NHC and co-medications is low.

e. Licensing Status

At the time of writing, the MHRA has approved the IMP for a Conditional Marketing Authorisation.

f. Manufacturer

Merck Sharp & Dohme (UK) Limited, Marketing Authorisation Number: PLGB 53095/0089.

g. Labelling and QP release

Vertical Pharma Resources Ltd (trading as IPS Pharma), 41 Central Avenue, West Molesey, KT8 2QZ, UK Authorisation number: WDA (H) 32879, will label and QP release the medication for trial purposes in accordance with Annex 13.

h. Storage

All trial medication is to be kept in a dry area, stored at 1° to 30°C (59° to 86°F). We will ask participants to store the medication at room temperature.

The medication will be stored at Vertical Pharma Resources Ltd in locked cupboards in restricted access rooms. It may also be stored securely with restricted access in the Nuffield Department of Primary Care Health Sciences; in GP Practices; in Pharmacies.

i. Distribution

Molnupiravir will be labelled and QP released by an accredited licensed central facility: Vertical Pharma Resources Ltd. Vertical Pharma Resources Ltd will prepare and dispatch the participant pack containing IMP, directly to the participant at home, in accordance with their SOPs. The labelled and QP released Molnupiravir can also be held by the PC-CTU and trial Hubs, from where it may also be issued to participants.

j. Drug accountability

No additional mechanisms for drug accountability are required beyond those outlined in the master protocol.

k. Drug destruction/returns

Participants will be asked to return unused Molnupiravir to the PC-CTU via pre-paid courier, which will be documented in accountability logs. After a final reconciliation of drug accountability records and authorisation by the sponsor or delegate, unused trial medication at the PC-CTU and Vertical Pharma Resources Ltd will be disposed of in line with local SOPs. Unused trial medication may be destroyed by an authorised third party.

l. Overdose

There is no human experience of overdosage with Molnupiravir. Treatment of overdose with Molnupiravir should consist of general supportive measures including the monitoring of the clinical status of the patient. Haemodialysis is not expected to result in effective elimination of NHC. (SmPC, section 4.9). In line with the SmPC we will monitor potential overdoses by asking in the daily diary and Day 7,14 and 28 call CRF whether the participant has taken more than the

For peer review only - http://bmjopen.bmj.com/site/about/guidelines.xhtml
© Copyright: The University of Oxford and Oxford University Hospitals NHS Foundation Trust 2019

specified dose. A safety alert will be triggered if the participant records that they have exceeded the dose

A doctor from the central clinical team will contact the participant immediately and then follow-up accordingly (at clinical discretion) to monitor any potential AEs caused by the overdose. This may include no further action or repeated contact depending on the nature and severity of symptoms.

8. Safety reporting

a. Adverse effects

Pregnancy will be recorded as an AE of Special Interest.

Reporting period: Occurring within 28-day following first administration of the IMP as requested by the MHRA. Such events discovered after 28-day time point, will also be reported.

b. Reference Safety Information (RSI)

See section 4.8 of the SmPC, Merck Sharp and Dohme (UK) Limited, 05 Nov 2021.

c. Risk/benefit assessment

The UK Antivirals Taskforce (AT) established by the Department of Health and Social Care recommends including Molnupiravir into the PANORAMIC platform with an 800mg twice a day, for five days**,** based on a review of efficacy and safety data.

i. Risks

In the available six clinical studies in participants with COVID-19 (n=922 with COVID-19 receiving placebo or Molnupiravir as multiple doses up to 800 mg for 5 days), Molnupiravir was well-tolerated, with no clinically meaningful trends were observed for changes in clinical laboratory values as a function of dose or treatment.

In one phase 1 randomised, double-blind, placebo-controlled SAD/MAD trial (single ascending dose/multiple ascending dose) in 130 healthy adult male and female participants, receiving placebo or Molnupiravir in single doses of 50 to 1600 mg or in multiple doses of 50 to 800 mg twice daily for 5.5 days, overall, found no clinically meaningful trends for changes in clinical laboratory values, vital signs, or ECGs as a function of dose or treatment.(12) No clinically meaningful haematological laboratory test result abnormalities were observed. Transient elevations in serum lipase of ≥3-times the ULN were observed ≥3 days after the last dose of trial drug in a low and similar proportion of Molnupiravir and placebo recipients and were not associated with clinical symptoms of pancreatitis.

In a Phase 2 trial randomised, placebo-controlled, double-blind trial in hospitalised patients with COVID-19, there was an imbalance in mortality rates in patients treated with Molnupiravir (14/218, 6.4%) compared with placebo (2/75, 2.7%). None of the deaths were considered related to trial intervention by the investigators, and most were associated with complications of COVID-19 or to secondary bacterial infections.

Taking this evidence into account, participation requires participants to agree to use adequate contraception for the duration of the treatment and 28 days of follow-up.

For peer review only - http://bmjopen.bmj.com/site/about/guidelines.xhtml

© Copyright: The University of Oxford and Oxford University Hospitals NHS Foundation Trust 2019

ii. Benefits

Molnupiravir may reduce SARS-CoV-2 viral loads, COVID-19 symptoms, risk of onward transmission, and severity of disease.

Virology data from clinical studies (Part 1 of MK-4482-001 in hospitalised patients and MK-4482-002 in non-hospitalised patients) show that treatment with Molnupiravir reduces the SARS-CoV-2 VL compared with placebo (based on change from baseline, slope of decline, and greater proportion of participants with a VL below the LOQ at early time points) in non-hospitalised participants enrolled in MK-4482-002 and participants who had COVID-19 symptom onset ≤5 days prior to randomisation in both MK-4482-001 and MK-4482-002. In addition, consistent with the proposed mechanism of action of Molnupiravir of viral error catastrophe, the highest percentage of mutations in viral RNA post-treatment at Day 5 were observed in the 800 mg Q12H intervention group in MK-4482-001 and MK-4482-002.

In hospitalised participants (MK-4482-001), the observed rate of sustained recovery through 29 days was low for all studied doses of Molnupiravir as compared with placebo. While no clear dose effect was observed across Molnupiravir doses studied, there were a higher number of deaths through Day 29 in participants who received Molnupiravir compared with placebo. None of the deaths were assessed as related to trial intervention.

In non-hospitalised participants (MK-4482-002) evaluation of the primary clinical efficacy endpoint showed that 11 of 299 participants were hospitalised through Day 29 (; ~3% of participants in the Molnupiravir intervention groups were hospitalised or died through Day 29 (compared with ~5% in the placebo group). All hospitalised participants had at least 1 risk factor for severe illness from COVID. Protocol-specified subgroup analyses for the primary endpoint indicated potential clinical benefit from treatment with Molnupiravir early in the course of disease (i.e., symptom onset ≤5 days prior to the day of randomisation) as well as in individuals with risk factors for progression to severe illness from COVID-19, including age >60 years.

For peer review only

For peer review only - http://bmjopen.bmj.com/site/about/guidelines.xhtml
© Copyright: The University of Oxford and Oxford University Hospitals NHS Foundation Trust 2019

NUFFIELD DEPARTMENT OF PRIMARY CARE HEALTH SCIENCES — Primary Care Clinical Trials Unit — PANORAMIC Platform Adaptive trial of NOvel antiviRals for eArly treatMent of COVID-19 In the Community

d. Risk Assessment: Oral Molnupiravir Four 200mg capsules (800mg) Molnupiravir, twice a day, for five days.

Hazard	Likelihood (L, M, H)	Impact (L, M, H)	Mitigation	Monitoring
1. Pregnancy:<br>i. Potential teratogenicity.<br><br>ii. There are no human studies of use among pregnant or lactating people.	H	H	Requirement for negative pregnancy test in participants of child-bearing potential, prior to starting medication.<br><br>We will exclude known pregnancy, breastfeeding, and require participants to use adequate contraception for the duration of the treatment and 28 days of follow-up.<br><br>During the pre-randomisation call, the clinician/research nurse will confirm this exclusion criteria with the participant.	Confirmation of negative pregnancy test documented in the Day 1 and/or Day 2 Call CRFs and Daily Diary.<br><br>We will monitor daily responses to the question 'have you become pregnant since starting the trial?' and follow-up as required to immediately stop treatment, if applicable.<br><br>Pregnancy occurring during the 28-day trial follow-up period will be reported as an AE of Special Interest.<br><br>As per 'PC-CTU SOP TM119 Pharmacovigilance', any pregnancy that occurs during IMP administration requires monitoring and follow-up until the outcome of the pregnancy is known. The CI or delegated individual will liaise with the relevant Obstetrician throughout the pregnancy.<br><br>The DSMC will be informed of any pregnancies in this treatment group, in weekly safety reports.

For peer review only - http://bmjopen.bmj.com/site/about/guidelines.xhtml
© Copyright: The University of Oxford and Oxford University Hospitals NHS Foundation Trust 2019

For peer review only

NUFFIELD DEPARTMENT OF PRIMARY CARE HEALTH SCIENCES
Primary Care Clinical Trials Unit
PANORAMIC Platform Adaptive trial of NOvel antiviRals for eArly treatMent of COVID-19 In the Community

2. Unknown/other potential side-effects.	M	M	All participants will receive a call on day 1 to make sure that they understand the possible risks associated with Molnupiravir and how to report potential side-effects and seek medical care if required.<br><br>Participants will be provided with a 24-hour contact telephone line to report any AEs that they experience and are concerned about, directly to a clinician.<br><br>We will collect symptoms and side effects from symptom diaries and participant telephone calls.	The DSMC will review weekly reports of unblinded symptom data to identify potential side-effects of Molnupiravir.<br><br>Any safety signals will be communicated to the TSC and TMG as defined in the DSMC Charter.<br><br>The ESG will review accumulating safety data in the Molnupiravir arm including AEs, SAEs and laboratory results as defined in the ISA and ESG Charter.<br><br>TMG will review the total number of SAEs as per TMG Charter.

For peer review only - http://bmjopen.bmj.com/site/about/guidelines.xhtml
© Copyright: The University of Oxford and Oxford University Hospitals NHS Foundation Trust 2019

3. Compliance			Participants will be asked in their daily diaries about trial medication use.	The trial team will monitor daily diary responses where the participant indicates that they have taken too much IMP and escalate to the clinical team to follow-up with the participant.

For peer review only

For peer review only - http://bmjopen.bmj.com/site/about/guidelines.xhtml
© Copyright: The University of Oxford and Oxford University Hospitals NHS Foundation Trust 2019

3 USUAL CARE PLUS PAXLOVID

1. Background

a. Potential mechanism of efficacy

Paxlovid consists of nirmatrelvir [PF-07321332] tablets and ritonavir tablets. Nirmatrelvir is an oral antiviral that has been developed specifically for treatment of COVID-19. (19) It is a protease inhibitor and inhibits the SARS-CoV-2 3CL protease, thereby preventing viral replication. (19) Ritonavir inhibits CYP3A-mediated metabolism of nirmatrelvir, and therefore increases plasma concentrations of nirmatrelvir to therapeutic levels.

b. Evidence for potential benefits of Paxlovid in COVID-19 illness

In vitro antiviral activity

In vitro studies have demonstrated that PF-07321332 is a potent inhibitor of SARS-CoV-2 3CL protease in a biochemical enzymatic assay (Ki = 3.11 nM) and in epithelial Vero E6 cells (EC50 = 74.5 nM). (19) PF-07321332 also exhibited antiviral activity against SARS-CoV-2 infection of differentiated normal human bronchial epithelial (dNHBE) cells (EC_{90} value of 181 nM) and human adenocarcinoma–derived alveolar basal epithelial cells expressing ACE2 (A549-ACE2 cells, EC_{90} value 215 nM). (19)

In vivo antiviral activity

PF-07321332 showed antiviral activity in mouse models with mouse-adapted SARS-CoV-2 infection in BALB/c and 129 mouse strains. Oral administration of PF-07321332 at 300 mg/kg or 1,000 mg/kg twice daily initiated 4 hours post-inoculation or 1,000 mg/kg twice daily initiated 12 hours post inoculation with SARS-CoV-2 MA10 resulted in reduction of lung viral titres and ameliorated indicators of disease (weight loss and lung pathology) compared to placebo-treated animals. (19)

Phase 1 studies

In a phase 1, randomised placebo-controlled trial of 70 healthy adult participants, (20) PF-07321332 was administered alone or with ritonavir in ascending doses. PF-07321332 was well tolerated and safe, and plasma concentrations were boosted when co-administered with ritonavir. (19) With a PF-07321332 dose of 250 mg, and 100mg of RTV at -12, 0 and 12 hours, plasma PF-07321332 concentrations after 12 hours were considerably above the SARS-CoV-2 antiviral EC_{90} value (total EC_{90} = 292 ng/ml, unbound EC_{90} = 90.5 ng/ml, 181 nM).

Phase 2/3 studies

The efficacy of Paxlovid to treat COVID-19 has been assessed in the Phase 2/3 Evaluation of Protease Inhibition for COVID-19 in High-Risk patients (EPIC-HR) trial. 2,246 non-hospitalized, high-risk adult patients with COVID-19 and symptom onset ≤5 days were randomised 1:1 to receive Paxlovid 300mg/100mg or placebo every 12 hours for 5 days. Eligible participants had at least one risk factor for severe COVID-19 and must not have been vaccinated or previously had COVID-19. Among those who received treatment within 3 days, 5/697 (0.7%) in the Paxlovid group met the primary endpoint of 28-day all-cause hospitalisation or death, compared to 44/682 (6.5%)

For peer review only - http://bmjopen.bmj.com/site/about/guidelines.xhtml
© Copyright: The University of Oxford and Oxford University Hospitals NHS Foundation Trust 2019

in the placebo group (relative risk reduction 89%). (21) (22) There were no deaths in the Paxlovid group and 9 deaths in the placebo group. In a secondary analysis among those treated within 5 days of symptom onset, 8/1039 (0.8%) in the Paxlovid group were hospitalised or died, versus 66/1046 (6.3%) in the placebo group (relative risk reduction 88%). Among 1574 participants who had SARS-CoV-2 viral load measured at Days 0 and 5, Day 5 viral loads were approximately 10-fold lower in the Paxlovid group versus placebo, after adjusting for baseline viral load, geographic region, and serology status. (21) Regarding safety, 23% of participants in the Paxlovid group experienced AEs, versus 24% in the placebo group. SAEs occurred in 1.6% of Paxlovid group versus 6.6% of placebo group participants. Dysgeusia (6% and <1%, respectively), diarrhoea (3% and 2%), and vomiting (1% and <1%) were the AEs (all grades regardless of causality) that occurred more frequently in the Paxlovid group (≥ 1%) than the placebo group respectively. (21)

The Evaluation of Protease Inhibition for COVID-19 in Standard-Risk Patients (EPIC-SR) Phase 2/3 trial, is assessing efficacy of Paxlovid among unvaccinated adults who were at standard risk (i.e., low risk of hospitalization or death) as well as vaccinated adults who had one or more risk factors for progressing to severe illness.(23) In an interim analysis, there was no evidence of superiority in the primary outcome of self-reported sustained recovery for 4 consecutive days.(21) The secondary outcome of hospitalisations and deaths was 70% lower in the Paxlovid group (3/428, 0.7%) versus placebo (10/426, 2.4%, p 0.051), and viral loads were approximately 10 times lower in the Paxlovid group. There were 22% versus 21% AEs, 1.4% versus 1.9% SAEs, and 2.1% versus 1.2% discontinuations of trial drug due to AEs in the Paxlovid versus placebo arms respectively. (21)

2. Detail of intervention

Participants randomised to the usual care plus Paxlovid arm will receive usual clinical care as per NHS guidelines, plus Paxlovid for five days. Nirmatrelvir must be given with ritonavir to achieve therapeutic concentrations. The usual recommended dosage is 300 mg PF 07321332 (two 150 mg tablets) with 100 mg ritonavir (one 100 mg tablet) all taken together orally twice daily for 5 days.

a. Precautions

Potential SARs due to drug-drug interactions

Paxlovid contains ritonavir. Ritonavir is an inhibitor, inducer, and substrate of various drug-metabolizing enzymes and/or drug transporters. Most notably, as a strong inhibitor of CYP3A, it may increase concentrations of certain concomitant medications, thereby increasing the potential for significant drug toxicities. CYP3A inhibition by ritonavir typically resolves 3 to 5 days after the drug is discontinued. When ritonavir is used for a treatment duration of 5 days, its induction properties are less likely to be clinically relevant than when the drug is used chronically for HIV. See section Appendix F for full lists of contraindicated concomitant medications and concomitant medications that may be taken with caution.

Medications that induce or inhibit CYP3A may also reduce or increase Paxlovid levels. Induction of 3A4 may result in sub-therapeutic Paxlovid levels, increasing the risk of development of viral

For peer review only

For peer review only - http://bmjopen.bmj.com/site/about/guidelines.xhtml
© Copyright: The University of Oxford and Oxford University Hospitals NHS Foundation Trust 2019

resistance. Increased inhibition of 3A4 may increase the risk of significant adverse reactions from increased levels of Paxlovid.

Hepatotoxicity

Increased hepatic transaminases, hepatitis and jaundice have occurred in patients receiving ritonavir. Patients with known severe liver disease will not be eligible to be randomised to Paxlovid.

Excipients

PF-07321332 tablets contain lactose. Patients with rare hereditary problems of galactose intolerance, total lactase deficiency or glucose-galactose malabsorption should not take Paxlovid.

b. Fertility, pregnancy, and lactation

Fertility

There are no human data on the effects of Paxlovid on fertility. In rats there was no evidence of effects of PF-07321332 on fertility or early embryonic development at doses up to 1000mg/kg/day, representing 12x/4.3x based on the predicted human Cmax/AUC24 at a twice-daily dose of 300 mg/100 mg PF-07321332/ritonavir.

Pregnancy

There is no human data on the effects of Paxlovid on pregnancy.

In studies of the effects of PF-07321332 on embryo-foetal development in rats and rabbits at doses of up to 1000mg/day, there was no evidence of PF-07321332 related effects in the rat model at any of the doses studied. In the rabbit model, foetal morphology and viability were not affected at any dose, however lower foetal body weights were noted with the highest dose of PF-07321332 1000mg/kg/day, along with slight decreases in maternal body weight and food consumption.

In rat and rabbit studies, ritonavir was associated with early resorptions, decreased foetal weight, ossification delays, decreased litter sizes and developmental variations, but only at dose levels that caused maternal toxicity. In humans, over 6100 live births have been reported to be exposed to ritonavir during pregnancy, of which 2800 were during the first trimester, with no increase in birth defects compared to rates seen in the population base birth defect surveillance system.

As the effect of Paxlovid on pregnancy in humans is unknown, pregnant women will be excluded and pregnancy will be reported as an AE of special interest.

As per 'PC-CTU SOP TM119 Pharmacovigilance', any pregnancy that occurs during Paxlovid (antiviral agent) administration requires monitoring and follow-up until the outcome of the pregnancy and any postnatal sequelae are known. The CI, PI or delegated individual will report any pregnancy occurring whilst in the trial to the PC-CTU. The Sponsor will report any pregnancy occurring whilst in the trial to UKTIS.

Breast-feeding

For peer review only - http://bmjopen.bmj.com/site/about/guidelines.xhtml
© Copyright: The University of Oxford and Oxford University Hospitals NHS Foundation Trust 2019

There are no human data on the effects of Paxlovid in breast-feeding, and it is not known whether PF-07321332 is excreted in human breast milk. Ritonavir is excreted in breast milk but the effect on breast milk production and on the new-born, infant is not known.

3. Trial visits

As per Master Protocol with the addition of extra safety calls on day 4 and day 10 to participants randomised to the Paxlovid arm only. The purpose of the day 4 safety call is to detect any early side-effects of Paxlovid and to enable the investigator to suggest changes to participants medication including stopping where required. The day 10 safety call is to allow the side-effect profile of Paxlovid to be compared against the SmPC for Paxlovid rather than the Usual Care arm.

4. Outcome measures

As per Master Protocol

5. Eligibility criteria (in addition to master protocol)

Inclusion criteria:

- Willing to take a pregnancy test after randomisation and prior to starting Paxlovid treatment (Participants of childbearing potential)
- Patients with known mild kidney disease (CKD) stage 2, must have an eGFR measurement taken in the past 6 months

Exclusion criteria:

- Known or suspected pregnancy*
- Breastfeeding*
- Participants of childbearing potential (participants who are anatomically and physiologically capable of becoming pregnant) who do not confirm a negative pregnancy test prior to starting the drug, and who are not willing to use one of the contraceptive methods for the durations defined below:
    - sterilisation, long-acting reversible contraceptive (LARC) methods (intrauterine devices, intrauterine systems, and implants), or the progestogen only pill or injection, for the 28-day duration of follow-up in the trial
    - combined hormonal contraception (oral, transdermal, or intravaginal) alongside **an additional barrier method** (e.g., male condom) for the duration of Paxlovid treatment, and until after one complete menstrual cycle after stopping Paxlovid
    - abstinence: being abstinent for the 28 days before enrolling in the trial and will continue to be abstinent for the 28-day duration of follow-up where this is in line with the preferred and usual lifestyle of the subject
    - Note: a barrier method on its own is **not** sufficient
- History of clinically significant hypersensitivity to the active substances in Paxlovid (PF-07321332/ritonavir) or to any of its excipients
- Patients with known rare hereditary problems of galactose intolerance, total lactase deficiency or glucose-galactose malabsorption

For peer review only - http://bmjopen.bmj.com/site/about/guidelines.xhtml
© Copyright: The University of Oxford and Oxford University Hospitals NHS Foundation Trust 2019

NUFFIELD DEPARTMENT OF PRIMARY CARE HEALTH SCIENCES Primary Care Clinical Trials Unit
PANORAMIC Platform Adaptive trial of NOvel antiviRals for eArly treatMent of COVID-19 In the Community

- Patients with known current severe liver impairment (characterised by severe ascites, encephalopathy, jaundice, or prolonged INR. People with liver disease **without** any of these features are eligible)
- Patients with known moderate or severe renal disease (defined as CKD stage 3, 4 or 5 or current acute kidney injury or most recent eGFR in the past 6 months <60 ml/min)
- Currently taking Paxlovid
- Clinical requirement to continue taking a drug which is contraindicated or not recommended for administration with Paxlovid in in the context of PANORAMIC (Appendix G) or is taking a drug which in the opinion of the investigator would put the subject at unacceptable risk

* As recorded by the participant on the screening form and confirmed by interaction between clinician and participant, and the pregnancy test supplied by the trial.

6. Professional role of those checking eligibility

To confirm that the participant meets the criteria defined above, information will be elicited through a direct discussion between the participant and a medically qualified professional , a prescribing pharmacist or a nurse prescriber (as required by standard prescribing practices at Covid Medicines Delivery Units across all four Administrations within the UK). Those assessing eligibility must take a relevant drug history and have access to a version of a summary care record in use in any Devolved Administration, and may, if necessary according to their clinical judgement, access and review further information contained within secondary care records or full primary care records.
If after reviewing relevant medical records and discussion with the patient, the recruiting health care professional considers the potential participant is eligible, they may then be randomised to Paxlovid.

7. Antiviral agent: Paxlovid

a. Name

Paxlovid is the brand name for two active substances nirmatrelvir (PF07321332) plus ritonavir. The drug will be referred to by brand name only.

b. Dose

Nirmatrelvir [PF-07321332] 150 mg tablets and ritonavir 100mg tablets. The tablets are for oral administration. Two 150 mg tablets (300mg) nirmatrelvir and one 100mg tablet (100 mg) ritonavir all taken together orally twice daily for 5 days.

If the patient misses a dose of Paxlovid within 8 hours of the time it is usually taken, the patient should take it as soon as possible and resume the normal dosing schedule. If the patient misses a dose by more than 8 hours, the patient should not take the missed dose and instead take the next

For peer review only - http://bmjopen.bmj.com/site/about/guidelines.xhtml
© Copyright: The University of Oxford and Oxford University Hospitals NHS Foundation Trust 2019

dose at the regularly scheduled time. The patient should not double the dose to make up for a missed dose.

If a patient requires hospitalization due to severe or critical COVID-19 after starting treatment with Paxlovid, the patient should complete the full 5-day treatment course at the discretion of his/her healthcare provider.

Paxlovid can be taken with or without food. The tablets should be swallowed whole and not chewed, broken, or crushed.

Renal failure

No dose adjustment is necessary for patients with mild renal impairment (eGFR ≥60 ml/min, CKD stage 1-2). Patients with moderate renal impairment (eGFR ≥30 to <60 mL/min, CKD stage 3) will not be eligible for randomisation to Paxlovid, as the dose of Paxlovid should be reduced to PF-07321332/ritonavir 150 mg/100 mg (1 tablet of each) twice daily for 5 days, and this is not feasible in this large scale, pragmatic trial. Patients with severe renal impairment (eGFR <30 ml/min, CKD stage 4-5) are not recommended to have Paxlovid and are also not eligible for randomisation to the Paxlovid arm.

Hepatic impairment

No dose adjustment is required for patients with mild to moderate hepatic impairment. Patients with severe hepatic impairment are not recommended for Paxlovid and are not eligible for randomisation to the Paxlovid arm.

Concomitant therapy with ritonavir- or cobicistat-containing regimen

No dose adjustment is needed; the dose of Paxlovid is 300 mg/100 mg twice daily for 5 days. Patients diagnosed with human immunodeficiency virus (HIV) or hepatitis C virus (HCV) infection who are receiving ritonavir- or cobicistat-containing regimen should continue their treatment as indicated.

c. Common side effects

Common side effects include dysgeusia (disturbances of taste), diarrhoea and vomiting. (22)

d. Concomitant medications

Medications that may interact with Paxlovid, and the implications for eligibility for PANORAMIC, are listed in Appendix G. This list is based on the summary of product characteristics and will be updated as new information becomes available. Clinical judgement is required to evaluate potential drug interactions. Detailed advice is also available from the Liverpool COVID-19 Drug Interactions Checker website: https://www.covid19- druginteractions.org/. Patients who are taking Paxlovid as part of the trial will be advised that they must check with a clinician before initiating any new medications while taking Paxlovid to ensure that the potential for drug-drug interaction has been considered. Such participants with also be provided with a drug interaction

For peer review only - http://bmjopen.bmj.com/site/about/guidelines.xhtml
© Copyright: The University of Oxford and Oxford University Hospitals NHS Foundation Trust 2019

warning card with advice for their clinician, and their clinician will be able to seek advice from the trials clinical team.

e. Licensing status

At the time of writing, the MHRA has issued a Conditional Marketing Authorisation for Paxlovid in Great Britain and a temporary Regulation 174 authorisation for Northern Ireland.

f. Manufacturer

Pfizer Limited, Ramsgate Rd, Sandwich, Kent, CT13 9NJ, UK.
Marketing Authorisation Number: PLGB 00057/1710

g. Labelling and QP release

Vertical Pharma Resources Ltd (trading as IPS Pharma), 41 Central Avenue, West Molesey, KT8 2QZ, UK Authorisation number: WDA (H) 32879, will label and QP release the medication for trial purposes in accordance with Annex 13.

h. Storage

All trial medication is to be kept in a dry area, stored at 1° to 30°C (59° to 86°F). We will ask participants to store the medication at room temperature and not to refrigerate or freeze.

The medication will be stored at Vertical Pharma Resources Ltd in locked cupboards in restricted access rooms. It may also be stored securely with restricted access in the Nuffield Department of Primary Care Health Sciences; in GP Practices; in Pharmacies.

i. Distribution

Paxlovid will be labelled and QP released by an accredited licensed central facility: Vertical Pharma Resources Ltd. Vertical Pharma Resources Ltd will prepare and dispatch the participant pack containing IMP, directly to the participant at home, in accordance with their SOPs. The labelled and QP released Paxlovid can also be held by the PC-CTU and trial Hubs, from where it may also be issued to participants.

j. Drug accountability

No additional mechanisms for drug accountability are required beyond those outlined in the master protocol.

k. Drug destruction/returns

Participants will be asked to return unused Paxlovid to the PC-CTU via pre-paid courier, which will be documented in accountability logs. After a final reconciliation of drug accountability records and authorisation by the sponsor or delegate, unused trial medication at the PC-CTU and Vertical Pharma Resources Ltd will be disposed of in line with local SOPs. Unused trial medication may be destroyed by an authorised third party.

For peer review only - http://bmjopen.bmj.com/site/about/guidelines.xhtml
For peer review only
© Copyright: The University of Oxford and Oxford University Hospitals NHS Foundation Trust 2019

l. Overdose

There is no human experience of overdosage with nirmatrelvir and limited human experience of acute overdose with ritonavir. One patient in clinical trials took ritonavir 1500 mg/day for two days and reported paraesthesia, which resolved after the dose was decreased, and a case of renal failure with eosinophilia after ritonavir overdose has been reported. (24)

The signs of ritonavir toxicity observed in animals (mice and rats) included decreased activity, ataxia, dyspnoea, and tremors.

Treatment of overdose with Paxlovid should consist of general supportive measures including the monitoring of the clinical status of the patient. In line with the SmPC we will monitor potential overdoses by asking in the daily diary and in the D2 and D4 call CRF whether the participant has taken more than the specified dose. A safety alert will be triggered if the participant records that they have exceeded the dose. A doctor from the central clinical team will contact the participant immediately and then follow-up accordingly (at clinical discretion) to monitor any potential AEs caused by the overdose. This may include no further action or repeated contact depending on the nature and severity of symptoms.

8. Safety reporting

a. Adverse effects

All non-COVID-19 events (at the discretion of the reporting nurse/clinician) reported during the safety and follow up calls and recorded in the daily symptom diaries will be recorded as AEs in the first instance.
Pregnancy will be recorded as an AE of Special Interest.
Reporting period: Occurring within 28-day following first administration of the IMP as requested by the MHRA. Such events discovered after 28-day time point, will also be reported.

b. Reference Safety Information

See section 4.8 of the SmPC, Pfizer (UK) Limited, 02-Mar-2022.

c. Risk/benefit assessment

The UK AT established by the Department of Health and Social Care recommends including Paxlovid into the PANORAMIC platform with a dose of 300/100mg twice a day, for five days, based on a review of efficacy and safety data.

i. Risks

For peer review only - http://bmjopen.bmj.com/site/about/guidelines.xhtml
© Copyright: The University of Oxford and Oxford University Hospitals NHS Foundation Trust 2019

Adverse events

In the EPIC-HR trial, among 2,224 symptomatic unvaccinated adults age ≥18 years of age and at high risk of developing severe COVID-19 illness, n=1,109 received at least one dose of Paxlovid and n=1,115 received placebo. 23% versus 24% experienced AEs, and 1.6% versus 6.6% experienced SAEs (including COVID-19 related AEs), in the Paxlovid group versus placebo group respectively.(3) AEs (all grades regardless of causality) in the Paxlovid group (≥1%) that occurred at a greater frequency (≥5 subject difference) than in the placebo group were dysgeusia (6% and <1%, respectively), diarrhoea (3% and 2%), hypertension (1% and <1%), and myalgia (1% and <1%). 2% of participants in the Paxlovid group and 4% in the placebo group discontinued treatment due to an AE. (22)

In an interim analysis of the EPIC-SR trial among standard risk patients (i.e., unvaccinated with no risk factors for severe disease or vaccinated with a risk factor for severe disease), AEs (22% versus 21%), SAEs(1.4% vs 1.9%) and discontinuation of trial drug due to AEs (2.1% vs. 1.2%) were comparable between Paxlovid (22%) and placebo (21%). (21)

Hepatic transaminase elevations, clinical hepatitis and jaundice have occurred in patients receiving ritonavir. Therefore, caution should be exercised when administering Paxlovid to patients with pre-existing liver diseases, liver enzyme abnormalities or hepatitis.

Risk of drug interactions

CYP3A related drug interactions listed in Appendix G could lead to clinically significant adverse reactions, including severe, life threatening or fatal events, due to increased levels of concomitant medications, or increased levels of Paxlovid. Medications that induce CYP3A may also reduce Paxlovid levels, leading to sub-therapeutic Paxlovid levels and the risk of development of viral resistance. This may occur if Paxlovid is initiated in patients receiving CYP3A metabolised medications, or if CYP3A metabolised medications are initiated among patients receiving Paxlovid.

Risk of pregnancy in participants receiving combined oral contraceptives

Ritonavir may reduce ethinyl estradiol concentrations and reduce the efficacy of combined oral contraceptive methods. This is unlikely to impair contraceptive efficacy, particularly considering the short duration of nirmatrelvir/ritonavir treatment, though it may increase the risk of irregular bleeding. (25) We will advise participants of childbearing potential who are using combined hormonal contraception (oral, transdermal, or intravaginal) to use an additional barrier method of contraception during treatment with Paxlovid, and until one menstrual cycle is completed after the last dose of Paxlovid.

Risks in pregnancy and during breastfeeding

There is no human data on the effect of Paxlovid on pregnancy or in breastfeeding. The summary of product characteristics states that breast-feeding should be discontinued during treatment with Paxlovid and for 7 days after the last dose of Paxlovid. Therefore, to be eligible for

For peer review only

For peer review only - http://bmjopen.bmj.com/site/about/guidelines.xhtml
© Copyright: The University of Oxford and Oxford University Hospitals NHS Foundation Trust 2019

NUFFIELD DEPARTMENT OF PRIMARY CARE HEALTH SCIENCES Primary Care Clinical Trials Unit

PANORAMIC Platform Adaptive trial of NOvel antiviRals for eArly treatMent of COVID-19 In the Community

randomisation to Paxlovid, participants are required to use a highly effective method of contraception for the duration of the treatment and 28 days of follow-up. Pregnant and breastfeeding participants will not be eligible.

Antiretroviral resistance

In individuals with HIV-1 viraemia (either undiagnosed or diagnosed but not controlled), the low dose ritonavir in Paxlovid may lead to a risk of HIV-1 developing resistance to HIV protease inhibitors. However, due to the short duration of ritonavir exposure, and the high genetic barrier to HIV-1 drug resistance with HIV protease inhibitors, this risk is thought to be low.

ii. Benefits

Paxlovid may reduce SARS-CoV-2 viral loads and severity of disease.

In the Phase 2/3 EPIC-HR trial among 2246 non-hospitalized high-risk adults with laboratory confirmed SARS-CoV-2 infection and with symptom onset ≤5 days, hospital admissions and deaths were 88% lower in the Paxlovid group compared to placebo. Day 5 nasopharyngeal viral load levels were approximately 9-fold lower in the Paxlovid group versus placebo. (21) (23)

In an interim analysis of the Phase 2/3 EPIC-SR trial among non-hospitalized **standard-risk** adults with laboratory confirmed SARS-CoV-2 infection and with symptom onset ≤5 days EPIC-SR, there was no difference in self-reported alleviation of all symptoms, but hospitalisations were 70% lower in the Paxlovid group versus placebo. Viral loads were also 10-fold lower in the Paxlovid group. (21)

d. Risk Assessment: Oral Paxlovid: two 150 mg tablets (300mg) nirmatrelvir and one 100mg tablet (100 mg) ritonavir all taken together orally twice daily for 5 days.

Hazard	Likelihood (L, M, H)	Impact (L, M, H)	Mitigation	Monitoring
1. Risk of drug interactions	H	H	We will exclude patients currently taking contra-indicated concomitant medication. Patients will be asked to confirm they are not taking contraindicated medication as part of the screening process.<br><br>Participants who report taking concomitant medication will be assessed for eligibility by a medically trained professional with	The DSMC will review weekly reports of unblinded symptom data to identify potential AEs caused by drug interactions with Paxlovid. Any safety signals will be communicated to the TSC and TMG as defined in the DSMC Charter. The ESG will review accumulating safety data in the

For peer review only - http://bmjopen.bmj.com/site/about/guidelines.xhtml
© Copyright: The University of Oxford and Oxford University Hospitals NHS Foundation Trust 2019

NUFFIELD DEPARTMENT OF PRIMARY CARE HEALTH SCIENCES Primary Care Clinical Trials Unit

PANORAMIC Platform Adaptive trial of NOvel antiviRals for eArly treatMent of COVID-19 In the Community

			access to a summary care medical record in use in any Devolved Administration in the UK and additional medical records if considered necessary. Participants who are on drugs that do not lead to exclusion (per Appendix G) but have specific recommendations for monitoring will be flagged on the Spinnaker data collection system.<br><br>Participants in the Paxlovid arm have enhanced safety follow up calls on days 2, 4 and 10. Participants who are flagged in the system will be asked about clinically significant drug interactions using the standard scripts (per Appendix F) on days 2 and 4. These include specific actions in the event of elicitation of AEs.<br><br>The importance of the participant informing their recruiting clinician or the safety line clinician, and completing the new medication CRF to alert the central safety team will be emphasised during the day 0, day 2 and day 4 calls.<br><br>Participants for whom we have no diary data will be asked additional questions	Paxlovid arm including AEs, SAEs and laboratory results as defined in the ISA and ESG Charter.

For peer review only - http://bmjopen.bmj.com/site/about/guidelines.xhtml
© Copyright: The University of Oxford and Oxford University Hospitals NHS Foundation Trust 2019

			regarding whether any new medications have been started during their day 7 diary follow-up call.<br><br>In addition, all participants will be asked in their daily diary (up to day 8) if they have started any new medications.<br><br>Completion of the new medication CRF will trigger a safety alert to the central safety team to follow-up participants.<br><br>Participants randomised to Paxlovid will be provided with an emergency wallet card stating that they are participating in the PANORAMIC trial and have been assigned Paxlovid. Their clinician will also be able to discuss any medication related queries with the trial clinical team.	
2. Pregnancy: There are no human studies of use among pregnant or lactating people.	H	H	Requirement for negative pregnancy test in participants of child-bearing potential, prior to starting medication. We will exclude known pregnancy, breastfeeding, and require participants to use effective contraception for the duration of the treatment and 28 days of follow-up.<br>During the pre-randomisation call, the	Confirmation of negative pregnancy test documented in the Day 1 and/or Day 2 Call CRFs and Daily Diary<br>We will monitor daily responses to the question 'have you become pregnant since starting the trial?' and follow-up as required to immediately stop

For peer review only - http://bmjopen.bmj.com/site/about/guidelines.xhtml
© Copyright: The University of Oxford and Oxford University Hospitals NHS Foundation Trust 2019

NUFFIELD DEPARTMENT OF PRIMARY CARE HEALTH SCIENCES Primary Care Clinical Trials Unit

PANORAMIC Platform Adaptive trial of NOvel antiviRals for eArly treatMent of COVID-19 In the Community

			clinician/research nurse will confirm this exclusion criteria with the participant.<br><br>Participants using combined hormonal contraceptive methods will not be eligible unless willing to use additional barrier methods during treatment with Paxlovid, and until after one complete menstrual cycle after stopping Paxlovid.	treatment, if applicable. Pregnancy occurring during the 28-day trial follow-up period will be reported as an AE of Special Interest. As per 'PC-CTU SOP TM119 Pharmacovigilance', any pregnancy that occurs during IMP administration requires monitoring and follow-up until the outcome of the pregnancy is known. The CI or delegated individual will liaise with the relevant Obstetrician throughout the pregnancy. The DSMC will be informed of any pregnancies in this treatment group.
3. Risk of antiretroviral resistance	L	M	The risk of HIV drug resistance with the short duration and dose of ritonavir is very low.	The risk of HIV drug resistance with the short duration and dose of ritonavir is very low.
4. Unknown/other potential side-effects	M	M	During the eligibility assessment, a medically qualified professional will fully explain the possible	The DSMC will review weekly reports of unblinded symptom data to

For peer review only - http://bmjopen.bmj.com/site/about/guidelines.xhtml
© Copyright: The University of Oxford and Oxford University Hospitals NHS Foundation Trust 2019

NUFFIELD DEPARTMENT OF PRIMARY CARE HEALTH SCIENCES Primary Care Clinical Trials Unit

PANORAMIC Platform Adaptive trial of NOvel antiviRals for eArly treatMent of COVID-19 In the Community

			risks associated with Paxlovid treatment to potential participants and advise them on how to report potential side-effects and seek medical care if required.<br><br>All participants will receive a call on day 2, 24h after starting treatment to discuss any side-effects experienced and how to seek medical care if required.<br><br>All participants will receive a call on day 4 to discuss any side-effects experienced and how to seek medical care. This will allow the investigator to detect any early side effects of Paxlovid and to suggest any required changes to the participants medication including stopping medications where required.<br><br>All participants will receive a call on day 10 to discuss any side-effects, this call will allow the investigator to compare the side-effect profile of Paxlovid against the SmPC rather than the Usual Care arm.<br><br>Participants will be provided with a 24-hour contact telephone line to report any AEs that they experience and	identify potential side-effects of Paxlovid. Any safety signals will be communicated to the TSC and TMG as per DSMC Charter.<br><br>The ESG will review accumulating safety data in the Paxlovid arm including AEs, SAEs and laboratory results as defined in the ISA and ESG Charter.<br><br>TMG will review the total number of SAEs as per TMG Charter.

For peer review only

For peer review only - http://bmjopen.bmj.com/site/about/guidelines.xhtml
© Copyright: The University of Oxford and Oxford University Hospitals NHS Foundation Trust 2019

			are concerned about, directly to a clinician. We will collect symptoms and side effects from symptom diaries and participant telephone calls.	
5. Compliance			Participants will be asked in their daily diaries about trial medication use	The trial team will monitor daily diary responses where the participant indicates that they have taken too much IMP and escalate to the clinical team to follow-up with the participant.

For peer review only

For peer review only - http://bmjopen.bmj.com/site/about/guidelines.xhtml
© Copyright: The University of Oxford and Oxford University Hospitals NHS Foundation Trust 2019

APPENDIX F: STANDARD SCRIPT FOR SAFETY MONITORING OF DRUGS THAT REQUIRE ADJUSTMENT WHEN CO-ADMINISTERED WITH PAXLOVID

Question 1	Question 2	Action
Are you taking:<br>Buprenorphine<br>*[Brand names: Bunov; Bupeaze; BuTec; BuTrans; BuVidal; Carlosafine; Hapoctasin; Panitas; Prefibin; Rebrikel; Reletrans; Relevtec]*<br><br>Norbuprenorphine	If yes:<br>Have you experienced increased drowsiness	If yes:<br>**Mild/moderate**- counsel about driving / operating machinery<br>Severe- withdraw Paxlovid
Are you taking:<br>Methadone<br>*[Brand name Physeptone]*	If yes:<br>Have you experienced increased withdrawal symptoms [N.B. subjects will know these as given for opioid dependency]	If yes:<br>**Mild**- follow up<br>**Moderate / severe**- withdraw Paxlovid
Are you taking:<br>Morphine<br>*[Brand names: Morphgesic; MST Continus; MXL capsules; Sevredol; Zomorph]*	If yes:<br>Have you experienced breakthrough pain/ increased pain	If yes:<br>**Mild**- recommend simple analgesia<br>**Moderate**- contact GP to request short term dose adjustment<br>**Severe**- withdraw Paxlovid
Are you taking:<br>Afatinib *[Giotrif]*,<br>Ceritinib *[Zykadia]*,<br>Dasatinib *[Sprycel]*,<br>Nilotinib *[Tasigna]*,<br>Vincristine,<br>Vinblastine,<br>Fostamatinib *[Tavlesse]*	If yes:<br>Have you experienced new symptoms of:<br>Bleeding or bruising<br>Nausea, vomiting or diarrhoea<br>Muscle pain or weakness<br>Pins and needles or shooting pains	If yes:<br>Withdraw Paxlovid
Are you taking:<br>Warfarin *[Brand name Marevan]*	If yes:<br>Ask to contact GP (unless unable to do so, in which case the safety monitor can do on their behalf) to organise an INR check on or around day 5 subject to self isolation advice	If the INR is out of range:<br>Withdraw Paxlovid

For peer review only

For peer review only - http://bmjopen.bmj.com/site/about/guidelines.xhtml
© Copyright: The University of Oxford and Oxford University Hospitals NHS Foundation Trust 2019

APPENDIX G: PAXLOVID DRUG-DRUG INTERACTIONS

How to use this Appendix

List A is a summary list in alphabetical order summarizing medication which is an absolute exclusion criterion for PANORAMIC Paxlovid arm, drugs that should not be included in PANORAMIC because temporary interruption or the monitoring requirements are considered impractical, and those drugs that may be included.

List B contains more detail with the rationale.

These lists are based on the SmPC of 02 March 2022 and UK Interim Clinical Commissioning Policy: Therapies for symptomatic non-hospitalised patients with COVID-19 and a protocol substantial amendment will be submitted to update List B when there are updates to the SmPC.

Clinical judgement is required to evaluate potential drug interactions. Detailed advice is also available from the Liverpool COVID-19 Drug Interactions Checker website. https://www.covid19-druginteractions.org/

List A: Alphabetical summary of drugs that may interact with Paxlovid

Note: You MUST check BOTH columns for each drug

Drugs NOT to be included in PANORAMIC Paxlovid arm	Drugs which may be included in PANORAMIC Paxlovid arm
**Drugs that are contraindicated with Paxlovid and /or because interruption or monitoring requirements considered impractical in the setting of the clinical trial**	**Drugs which may be used with Paxlovid with caution**<br><br>**Those marked with an asterisk have a specific recommendation- see list B for details**<br><br>**The investigator should consider whether inclusion is appropriate**
acalabrutinib<br>abemaciclib<br>aliskiren<br>alfuzosin<br>amiodarone<br>Amlodipine (≥10 mg daily)<br>apalutamide<br>apixaban<br>astemizole<br>atovaquone<br>avanafil<br>bedaquiline<br>bepridil<br>bosentan<br>carbamazepine	afatinib<br>alprazolam*<br>amitriptyline<br>amlodipine (2.5 or 5 mg) *<br>amprenavir<br>atazanavir<br>atorvastatin<br>budesonide<br>buprenorphine*<br>bupropion<br>buspirone*<br>ceritinib<br>clarithromycin*<br>clopidogrel*<br>dabigatran*

For peer review only - http://bmjopen.bmj.com/site/about/guidelines.xhtml
© Copyright: The University of Oxford and Oxford University Hospitals NHS Foundation Trust 2019

cisapride	darunavir
clonazepam	dasatinib
clozapine	dexamethasone
colchicine	diltiazem
cyclosporine	divalproex
dasabuvir	efavirenz
delaminid	ethinyl estradiol*
desipramine	fexofenadine
dexamfetamine	fluoxetine
diazepam	fluticasone propionate
digoxin	fluvastatin
dihydroergotamine	fosamprenavir
disopyramide	fostamatinib
dronedarone	haloperidol*
elbasvir	itraconazole (topical)
eletriptan	ketoconazole (topical)
encainide	lamotrigine
encorafenib	levothyroxine
(enzalutamide- discontinued)	loratadine
eplerenone	maraviroc
ergonovine	methadone*
ergotamine	morphine*
erythromycin	nifedipine
estazolam	nilotinib
everolimus	norbuprenorphine
fentanyl	nortriptyline
flecainide	paroxetine
flurazepam	pravastatin
fusidic acid	prednisolone
glecaprevir	raltegravir
grazoprevir	risperidone*
ibrutinib	rosuvastatin
imipramine	sertraline
isavuconazole	sulfamethoxazole/trimethoprim
itraconazole (systemic)	theophylline
ivabradine	thioridazine*
ketoconazole (systemic)	triamcinolone
lercanidipine	trimethoprim
letermovir	valproic acid
lomitapide	vinblastine
lovastatin	vincristine
lurasidone	voriconazole (topical)
methylergonovine	warfarin*
methylphenidate	zidovudine
midazolam (oral or parenteral)	zolpidem*
neratinib	
pethidine	
phenobarbital	

For peer review only

For peer review only - http://bmjopen.bmj.com/site/about/guidelines.xhtml
© Copyright: The University of Oxford and Oxford University Hospitals NHS Foundation Trust 2019

phenytoin<br>pibrentasvir<br>pimozide<br>piroxicam (systemic)<br>propafenone<br>propoxyphene<br>quetiapine<br>quinidine<br>ranolazine<br>rifabutin<br>rifampicin<br>riociguat<br>rivaroxaban<br>salmeterol<br>sildenafil (Revatio®, for pulmonary arterial hypertension or for erectile dysfunction)<br>simvastatin<br>sirolimus<br>sodium fusidate<br>St. John's Wort (Hypericum perforatum)<br>tadalafil<br>tacrolimus<br>terfenadine<br>ticagrelor<br>triazolam<br>vardenafil<br>venetoclax<br>vorapaxar<br>voriconazole (systemic)	

Details of Paxlovid drug interactions and implications for eligibility for the Paxlovid arm of the PANORAMIC trial

Paxlovid is subject to a large number of drug interactions. At this stage the full range of interactions and their clinical significance is incompletely understood as clinical experience is limited. The PANORAMIC trial participants are home-based and are advised to isolate. This imposes some constraints on drugs which can be safely co-administered with Paxlovid in this context.

The following list is based on the summary of product characteristics (SmPC) list of drugs that are contraindicated for use with Paxlovid or should be used with caution with Paxlovid. For each a recommendation is provided based on a risk assessment. List B will be updated as new information becomes available and a protocol substantial amendment will be submitted for regulatory approval when the list is modified. The list is not exhaustive and if the investigator considers the potential participant is taking a drug which could put the subject at unacceptable risk, they should be excluded. There are some drugs which can be interrupted or adjusted during the trial- a specific recommendation is made for these.

For peer review only - http://bmjopen.bmj.com/site/about/guidelines.xhtml
© Copyright: The University of Oxford and Oxford University Hospitals NHS Foundation Trust 2019

List B: Details of Paxlovid drug interactions and implications for eligibility for drugs that are not recommended or require adjustment with Paxlovid in the PANORAMIC trial

Medicinal product class	Drugs in class with indicative effect of Paxlovid on Concentration of Medicinal product	Clinical comments	Implications for eligibility in PANORAMIC Paxlovid arm
α1-adrenoreceptor antagonist	↑alfuzosin	Increased alfuzosin plasma concentrations may lead to severe hypotension. **Contraindicated.**	**NOT ELIGIBLE**
Aldosterone antagonist	↑Eplerenone	Not recommended with strong 3A4 inhibitor as risk of hyperkalaemia. **Contraindicated.**	**NOT ELIGIBLE**
Amphetamine derivatives	↑methylphenidate, ↑dexamfetamine	Potential for increased concentrations of amphetamine and its derivatives. Careful monitoring of adverse effects is recommended. **Contraindicated.**	**NOT ELIGIBLE**
Analgesics	↑buprenorphine, ↑norbuprenorphine	The increases of plasma levels of buprenorphine and its active metabolite did not lead to clinically significant pharmacodynamic changes in a population of opioid tolerant patients.	**ELIGIBLE** – advise to monitor for opioid toxicity.
	↑pethidine, ↑piroxicam (systemic), ↑propoxyphene	Increased plasma concentrations may result in serious respiratory depression or haematologic abnormalities. **Contraindicated.**	**NOT ELIGIBLE**
	↑fentanyl	Ritonavir expected to increase the plasma concentrations of fentanyl. **Contraindicated.**	**NOT ELIGIBLE**
	↓methadone	Increased methadone dose may be necessary. Dose adjustment should be considered based on the patient's clinical response to methadone therapy.	**ELIGIBLE** – advise to monitor for potential under dosing of methadone.

For peer review only

For peer review only - http://bmjopen.bmj.com/site/about/guidelines.xhtml
© Copyright: The University of Oxford and Oxford University Hospitals NHS Foundation Trust 2019

	↓morphine	Morphine levels may be decreased due to induction of glucuronidation by co-administered ritonavir dosed as a pharmacokinetic enhancer.	**ELIGIBLE** – advise to monitor for morphine underdosing and adjust dose if required.
Antianginal	↑ranolazine	Potentially increased ranolazine plasma concentrations may result in serious and/or life-threatening reactions.<br><br>**Contraindicated.**	**NOT ELIGIBLE**
Antiarrhythmics	↑amiodarone<br>↑bepridil<br>↑disopyramide,<br>↑dronedarone,<br>↑encainide,<br>↑flecainide,<br>↑ivabradine<br>↑propafenone,<br>↑quinidine	Potentially increased plasma concentrations may result in arrhythmias or other serious adverse effects.<br><br>**Contraindicated.**	**NOT ELIGIBLE**
	↑digoxin	Potentially increased concentrations. Inhibition of pgp may decrease renal digoxin clearance. Magnitude of effect not known.<br><br>**Contraindicated.**	**NOT ELIGIBLE**
Antiasthmatic	↓theophylline	Ritonavir could potentially decrease theophylline concentrations, but effects unlikely with short course of Paxlovid.	**ELIGIBLE** – no theophylline dose adjustment required.
Anticancer agents	↑afatinib,<br>↑ceritinib, ↑dasatinib,<br>↑nilotinib,<br>↑vincristine,<br>↑vinblastine,<br>↑fostamatinib	Serum concentrations may be increased when co-administered with ritonavir resulting in the potential for increased incidence of AEs.	**ELIGIBLE** with caution. Advise to monitor symptoms of increased anticancer agent concentrations.
	↑acalabrutinib<br>↑apalutamide,<br>↑abemaciclib,<br>↑encorafenib,<br>↑ibrutinib	Co-administration not recommended due to potentially increased concentration of anticancer agents and SAEs. Apalutamide may also decrease exposure of Paxlovid and cause potential loss of virologic response.	**NOT ELIGIBLE**

For peer review only - http://bmjopen.bmj.com/site/about/guidelines.xhtml
© Copyright: The University of Oxford and Oxford University Hospitals NHS Foundation Trust 2019

		**Contraindicated.**	
	↑neratinib, ↑venetoclax	Increased plasma concentrations which may increase the potential for serious and/or life-threatening reactions.<br><br>**Contraindicated.**	**NOT ELIGIBLE**
Anticoagulants	warfarin, ↑↓S-warfarin, ↓↔R-warfarin	Potentially decreased R-warfarin concentrations which may lead to reduced anticoagulation.	**ELIGIBLE** – recommend check INR on or around day 5 (as self-isolation allows)
	↑apixaban,	Potentially increased concentrations which may lead to an increased bleeding risk.<br><br>**Contraindicated.**	**NOT ELIGIBLE** as contraindicated.
	↑dabigatran	Potentially increased concentrations which may lead to an increased bleeding risk. No effect when co-administered with ritonavir (small effect when given at different time).	**ELIGIBLE –** recommend taking Paxlovid simultaneously with dabigatran
	↑rivaroxaban	Potentially increased concentrations which may lead to an increased bleeding risk.<br><br>**Contraindicated.**	**NOT ELIGIBLE**
	↑vorapaxar	Serum concentrations may be increased. Coadministration not recommended.<br><br>**Contraindicated.**	**NOT ELIGIBLE** as contraindicated.
Anticonvulsants	carbamazepine, phenobarbital These drugs are expected to reduce the concentrations of Paxlovid	Decreased plasma concentrations of Paxlovid may lead to loss of virologic response and possible resistance.<br><br>**Contraindicated.**	**NOT ELIGIBLE** as contraindicated.

For peer review only - http://bmjopen.bmj.com/site/about/guidelines.xhtml
© Copyright: The University of Oxford and Oxford University Hospitals NHS Foundation Trust 2019

	↓phenytoin	Ritonavir is expected to decrease the plasma concentrations of phenytoin. Phenytoin may decrease serum levels of ritonavir.<br><br>**Contraindicated.**	**NOT ELIGIBLE**
	↓divalproex, valproic acid ↓lamotrigine	Ritonavir may decrease the plasma concentrations of anticonvulsants over time but given the short course of Paxlovid treatment, no a priori dosage adjustment is recommended.	**ELIGIBLE**
Antidepressants	↑amitriptyline,<br><br>↑fluoxetine,<br>↑nortriptyline,<br>↑paroxetine,<br>↑sertraline	Ritonavir used at higher doses than present in Paxlovid may increase concentrations of these antidepressants. With Paxlovid no a priori dosage adjustment is recommended.	**ELIGIBLE**
	↑desipramine,	Dosage reduction is recommended when co-administered.	**NOT ELIGIBLE**
	↑imipramine	Nirmatrelvir/ritonavir could potentially increase imipramine concentrations and increase the risk of QT prolongation.<br><br>**Contraindicated.**	**NOT ELIGIBLE**
Anti-gout	↑colchicine	Increased colchicine plasma concentrations may result in serious and/or life-threatening reactions.<br><br>**Contraindicated.**	**NOT ELIGIBLE**
Antihistamines	↑astemizole,<br>↑terfenadine	Increased plasma concentrations of astemizole and terfenadine may result in serious arrhythmias from these agents. Note both withdrawn from market globally.<br><br>**Contraindicated.**	**NOT ELIGIBLE**
	↑fexofenadine<br>↑loratadine	Ritonavir may increase fexofenadine and loratadine concentrations.	**ELIGIBLE**

For peer review only

For peer review only - http://bmjopen.bmj.com/site/about/guidelines.xhtml
© Copyright: The University of Oxford and Oxford University Hospitals NHS Foundation Trust 2019

Antifungals	↑ketoconazole, ↓voriconazole, ↑itraconazole, ↑isavuconazole	Potentially increased concentrations of ketoconazole, isavuconazole and itraconazole, and decreased plasma concentrations of voriconazole.<br><br>**Systemic use contraindicated.**	**NOT ELIGIBLE** when administered systemically. Topically used agents are not an exclusion criterion.
Antimycobacterial	rifampicin	Potentially decreased concentrations of Paxlovid may lead to loss of virologic response and possible resistance.<br><br>**Contraindicated.**	**NOT ELIGIBLE**
	↑bedaquiline, ↑delaminid ↑rifabutin	Potentially increased plasma concentrations of bedaquiline, delaminid and rifabutin.<br><br>**Contraindicated.**	**NOT ELIGIBLE**
Anti-infective	↑clarithromycin ↓14-OH clarithromycin metabolite	Potentially increased plasma concentrations of clarithromycin. Clarithromycin doses greater than 1 gr per day should not be co-administered with Paxlovid. For patients with renal impairment, a clarithromycin dose reduction should be considered: for patients with creatinine clearance of 30 to 60 ml/min the dose should be reduced by 50%, for patients with creatinine clearance less than 30 ml/min the dose should be reduced by 75%.	**Potentially ELIGIBLE** if no known renal impairment. If known renal impairment, **NOT ELIGIBLE.**
	↑erythromycin	Ritonavir is expected to increase plasma concentrations of erythromycin which may increase risk of QT prolongation.<br><br>**Contraindicated.**	**NOT ELIGIBLE**
	sulfamethoxazole/trimethoprim	Dose alteration of sulfamethoxazole/trimethoprim should not be necessary.	**ELIGIBLE**
	↑fusidic acid / sodium fusidate	Increased plasma concentrations of fusidic acid and ritonavir.<br><br>**Contraindicated.**	**NOT ELIGIBLE**

For peer review only - http://bmjopen.bmj.com/site/about/guidelines.xhtml
© Copyright: The University of Oxford and Oxford University Hospitals NHS Foundation Trust 2019

Anti-malarial	↓atovaquone	Ritonavir is expected to decrease the plasma concentrations of atovaquone.<br><br>**Contraindicated.**	**NOT ELIGIBLE**
Anti-platelet	↓Clopidogrel	Paxlovid may reduce conversion to active drug. Avoid in in patients at high risk of thrombosis and those within 6 weeks of stenting.	**POTENTIALLY ELIGIBLE. If within 6 weeks of coronary stent or at high risk of thrombosis NOT ELIGIBLE**
	↑ticagrelor	Expected to substantially increase exposure to ticagrelor.<br><br>**Contraindicated.**	**NOT ELIGIBLE**
Anti-HIV protease inhibitors	↑amprenavir, ↑atazanavir, ↑darunavir, ↑fosamprenavir	Potentially increased concentrations of protease inhibitors, but consensus is that no dose adjustment needed.	**ELIGIBLE**
Anti-HIV	↑efavirenz, ↑maraviroc	Potentially increased plasma concentrations of efavirenz and maraviroc.	**ELIGIBLE** – advise monitor for potential side effects.
	↓raltegravir, ↓zidovudine	Potentially minor decreased plasma concentrations of raltegravir and zidovudine.	**ELIGIBLE** – no dose adjustments required.
Antiviral	Letermovir. This drug is expected to reduce concentrations of Paxlovid.	Letermovir is an enzyme inducer so may render Paxlovid ineffective.<br><br>**Contraindicated.**	**NOT ELIGIBLE**
Antipsychotics	↑clozapine, ↑pimozide, ↑lurasidone ↑quetiapine	Increased concentrations may result in serious and/or life-threatening reactions.<br><br>**Contraindicated.**	**NOT ELIGIBLE**
	↑Haloperidol, ↑Risperidone, ↑Thioridazine	Ritonavir is likely to increase concentrations of haloperidol, risperidone and thioridazine.	**ELIGIBLE** – with caution and advise to monitor for increased adverse effects.
Long-acting beta-adrenoceptor agonist	↑salmeterol	Ritonavir is expected to increase the plasma concentrations of salmeterol, and may increase risk of QT prolongation, palpitations, and sinus tachycardia. Therefore,	**NOT ELIGIBLE**

For peer review only - http://bmjopen.bmj.com/site/about/guidelines.xhtml
© Copyright: The University of Oxford and Oxford University Hospitals NHS Foundation Trust 2019

		concomitant use is not recommended.<br><br>**Contraindicated.**	
Calcium channel antagonist	↑amlodipine,	Ritonavir is expected to increase the plasma concentrations of calcium channel antagonists.	**Potentially ELIGIBLE** – if taking 2.5 or 5 mg. If taking 10 mg or able **NOT ELIGIBLE**
	↑diltiazem,<br>↑nifedipine	Ritonavir may increase the plasma concentrations of calcium channel antagonists.	**ELIGIBLE** – advise to monitor for side effects.
	↑lercanidipine	Expected to substantially increase exposure to lercanidipine.<br><br>**Contraindicated.**	**NOT ELIGIBLE**
Endothelin receptor antagonists	↑bosentan<br>↑riociguat	Potentially increased concentrations.<br><br>**Contraindicated.**	**NOT ELIGIBLE**
Ergot Derivatives	↑dihydroergotamine,<br>↑ergonovine,<br>↑ergotamine,<br>↑methylergonovine	Increased concentrations of ergot derivatives potentially leading to acute ergot toxicity, including vasospasm and ischaemia.<br><br>**Contraindicated.**	**NOT ELIGIBLE**
GI motility agent	↑cisapride	Increased plasma concentrations of cisapride, thereby increasing the risk of serious arrhythmias from this agent.<br><br>**Contraindicated.**	**NOT ELIGIBLE**
Hepatitis C direct acting antivirals	↑elbasvir/grazoprevir,<br>↑glecaprevir/pibrentasvir<br>↑dasabuvir	Serum concentrations may be increased by ritonavir, leading to an increased risk of ALT elevations associated with increased glecaprevir and grazoprevir exposure.<br><br>**Contraindicated.**	**NOT ELIGIBLE**
Herbal products	St. John's Wort (*Hypericum perforatum*)<br>This drug is expected to reduce concentrations of Paxlovid	Potentially decreased concentrations of Paxlovid may lead to loss of virologic response and possible resistance.<br><br>**Contraindicated.**	**NOT ELIGIBLE**

For peer review only - http://bmjopen.bmj.com/site/about/guidelines.xhtml
© Copyright: The University of Oxford and Oxford University Hospitals NHS Foundation Trust 2019

NUFFIELD DEPARTMENT OF PRIMARY CARE HEALTH SCIENCES
Primary Care Clinical Trials Unit

PANORAMIC
Platform Adaptive trial of NOvel antiviRals for eArly treatMent of COVID-19 In the Community

HMG-CoA reductase inhibitors	↑lovastatin, ↑simvastatin	Increased concentrations resulting in increased risk of myopathy, including rhabdomyolysis. **Contraindicated.**	**NOT ELIGIBLE**
	↑atorvastatin, ↑fluvastatin, ↑pravastatin, ↑rosuvastatin,	Potentially increased concentrations.	**ELIGIBLE** – advise to monitor for side effects.
Microsomal triglyceride transfer protein (MTTP) inhibitor	↑lomitapide	Increased plasma concentrations of lomitapide.<br><br>**Contraindicated.**	**NOT ELIGIBLE**
Migraine treatments	↑Eletriptan	Not recommended with strong 3A4 inhibitors.<br><br>**Contraindicated.**	**NOT ELIGIBLE**
Hormonal contraceptive	↓ethinyl estradiol	Ritonavir may reduce ethinyl estradiol concentrations and change the uterine bleeding profile and reduce the effectiveness of estradiol-containing contraceptives.	**Potentially ELIGIBLE** if willing to use an additional barrier method during treatment with Paxlovid, and until one full menstrual cycle after stopping Paxlovid.
Immunosuppressants	↑cyclosporine, ↑tacrolimus, ↑everolimus ↑sirolimus	Ritonavir is expected to increase the plasma concentrations of cyclosporine, tacrolimus, sirolimus or everolimus.<br><br>**Contraindicated.**	**NOT ELIGIBLE**
Phosphodiesterase (PDE5) Inhibitors	↑ avanafil ↑ vardenafil	Increased plasma concentrations of avanafil and vardenafil.<br><br>**Contraindicated.**	**NOT ELIGIBLE**
	↑sildenafil (Revatio®) used for pulmonary arterial hypertension (PAH)	Increased sildenafil concentrations can potentially result in visual abnormalities, hypotension, prolonged erection, and syncope.<br><br>**Contraindicated.**	**NOT ELIGIBLE**
	↑sildenafil for erectile dysfunction	**Contraindicated.**	**NOT ELIGIBLE**
	↑tadalafil	**Contraindicated.**	**NOT ELIGIBLE**
Renin inhibitor	↑Aliskiren	Not recommended with a 3A4 and pgp inhibitor.	**NOT ELIGIBLE**

For peer review only - http://bmjopen.bmj.com/site/about/guidelines.xhtml
© Copyright: The University of Oxford and Oxford University Hospitals NHS Foundation Trust 2019

		**Contraindicated.**	
Sedative/hypnotics	↑clonazepam, ↑diazepam, ↑estazolam, ↑flurazepam, ↑oral and parenteral midazolam, ↑triazolam	Increased concentrations of can increase risk of extreme sedation and respiratory depression.<br><br>**Contraindicated.**	**NOT ELIGIBLE**
	↑alprazolam<br>↑buspirone	Potentially increased concentrations of alprazolam and buspirone.	**ELIGIBLE** – but advise to monitor for side effects and drowsiness.
Sleeping agent	↑zolpidem	Zolpidem & ritonavir may be co-administered with careful monitoring for excessive sedative effects.	**ELIGIBLE** – but advise to monitor for side effects and drowsiness.
Smoke cessation	↓bupropion	Concurrent administration of bupropion with repeated doses of ritonavir is expected to decrease bupropion levels. Effects may not be seen with the short course of Paxlovid.	**ELIGIBLE**
Steroids	Inhaled, injectable or intranasal fluticasone propionate, budesonide, triamcinolone	Systemic corticosteroid effects have been reported in patients receiving long-term ritonavir and inhaled or intranasal fluticasone propionate<br>Given the short course of Paxlovid this risk is considered to be low.	**ELIGIBLE**
	↑dexamethasone, ↑prednisolone	Ritonavir is expected to increase concentrations of dexamethasone and prednisolone. However, given the short duration of Paxlovid treatment, this risk is considered to be low.	**ELIGIBLE**
Thyroid hormone replacement therapy	levothyroxine (no interaction expected)	Post-marketing cases have been reported indicating a potential interaction between ritonavir containing products and levothyroxine. Given the short duration of Paxlovid treatment, this risk is considered to be low.	**ELIGIBLE**

For peer review only - http://bmjopen.bmj.com/site/about/guidelines.xhtml
© Copyright: The University of Oxford and Oxford University Hospitals NHS Foundation Trust 2019

20. REFERENCES

1. Hippisley-Cox J, Coupland CA, Mehta N, Keogh RH, Diaz-Ordaz K, Khunti K, et al. Risk prediction of covid-19 related death and hospital admission in adults after covid-19 vaccination: national prospective cohort study. BMJ. 2021;374:n2244.

2. Stokel-Walker C. The search for antivirals for covid-19. BMJ. 2021;374:n2165.

3. Kim PS, Read SW, Fauci AS. Therapy for Early COVID-19: A Critical Need. JAMA. 2020;324(21):2149-50.

4. Cevik M, Tate M, Lloyd O, Maraolo AE, Schafers J, Ho A. SARS-CoV-2, SARS-CoV, and MERS-CoV viral load dynamics, duration of viral shedding, and infectiousness: a systematic review and meta-analysis. Lancet Microbe. 2021;2(1):e13-e22.

5. van Hout B, Janssen MF, Feng YS, Kohlmann T, Busschbach J, Golicki D, et al. Interim scoring for the EQ-5D-5L: mapping the EQ-5D-5L to EQ-5D-3L value sets. Value Health. 2012;15(5):708-15.

6. National Institute for Health and Care Excellence (NICE). NICE Guide to the methods of technology appraisal London, UK: NICE; 2013 [Available from: http://www.nice.org.uk/article/pmg9/resources/non-guidance-guide-to-the-methods-oftechnology-appraisal-2013-pdf.

7. HM Treasury. The Green Book. Appraisal and Evaluation in Central Government. 2020 [Available from: https://www.gov.uk/government/publications/the-green-book-appraisal-and-evaluationin-central-governent.

8. Yu LM, Bafadhel M, Dorward J, Hayward G, Saville BR, Gbinigie O, et al. Inhaled budesonide for COVID-19 in people at high risk of complications in the community in the UK (PRINCIPLE): a randomised, controlled, open-label, adaptive platform trial. Lancet. 2021;398(10303):843-55.

9. Dorward J, Yu LM, Hayward G. Colchicine for COVID-19 in adults in the community (PRINCIPLE): a randomised, controlled, adaptive platform trial. medRxiv 2021.

10. Gastine S, Pang J, Boshier FAT, Carter SJ, Lonsdale DO, Cortina-Borja M, et al. Systematic Review and Patient-Level Meta-Analysis of SARS-CoV-2 Viral Dynamics to Model Response to Antiviral Therapies. Clin Pharmacol Ther. 2021;110(2):321-33.

11. Laouenan C, Guedj J, Mentre F. Clinical trial simulation to evaluate power to compare the antiviral effectiveness of two hepatitis C protease inhibitors using nonlinear mixed effect models: a viral kinetic approach. BMC Med Res Methodol. 2013;13:60.

12. Amara A, Penchala SD, Else L, Hale C, FitzGerald R, Walker L, et al. The development and validation of a novel LC-MS/MS method for the simultaneous quantification of Molnupiravir and its metabolite ss-d-N4-hydroxycytidine in human plasma and saliva. J Pharm Biomed Anal. 2021;206:114356.

13. Fischer W, Eron JJ, Holman W, Cohen MS, Fang L, Szewczyk LJ, et al. Molnupiravir, an Oral Antiviral Treatment for COVID-19. medRxiv. 2021.

14. Imran M, Kumar Arora M, Asdaq SMB, Khan SA, Alaqel SI, Alshammari MK, et al. Discovery, Development, and Patent Trends on Molnupiravir: A Prospective Oral Treatment for COVID-19. Molecules. 2021;26(19).

15. Abdelnabi R, Foo CS, Kaptein SJF, Zhang X, Do TND, Langendries L, et al. The combined treatment of Molnupiravir and Favipiravir results in a potentiation of antiviral efficacy in a SARS-CoV-2 hamster infection model. EBioMedicine. 2021;72:103595.

16. Singh AK, Singh A, Singh R, Misra A. Molnupiravir in COVID-19: A systematic review of literaturef. Diabetes Metab Syndr. 2021;15(6):102329.

For peer review only - http://bmjopen.bmj.com/site/about/guidelines.xhtml
© Copyright: The University of Oxford and Oxford University Hospitals NHS Foundation Trust 2019

17. Khoo SH, Fitzgerald R, Fletcher T, Ewings S, Jaki T, Lyon R, et al. Optimal dose and safety of Molnupiravir in patients with early SARS-CoV-2: a Phase I, open-label, dose-escalating, randomized controlled study. J Antimicrob Chemother. 2021.

18. Mahase E. Covid-19: Molnupiravir reduces risk of hospital admission or death by 50% in patients at risk, MSD reports. BMJ. 2021;375:n2422.

19. Owen DR, Allerton CMN, Anderson AS, et al. An oral SARS-CoV-2 M(pro) inhibitor clinical candidate for the treatment of COVID-19. *Science* 2021; **374**(6575): 1586-93.

20. ClinicalTrials.gov. Study of PF-07321332 in health participants. 2021. https://clinicaltrials.gov/ct2/show/NCT04756531.

21. Hammond J, Leister-Tebbe H, Gardner A, et al. Oral Nirmatrelvir for High-Risk, Nonhospitalized Adults with Covid-19. N Engl J Med 2022.

22.Pfizer Limited. Paxlovid 150 mg/100 mg film-coated tablets. Summary of Product Characteristics. Updated 02-Mar-2022.

23. Pfizer. Evaluation of Protease Inhibition for COVID-19 in Standard-Risk Patients (EPIC-SR) (NCT05011513). 2021. https://clinicaltrials.gov/ct2/show/NCT05011513 (accessed 06 Jan 2022.

24. The Electronic Medicines Compendium (EMC). Norvir 100mg film-coated tablets. 2022. https://www.medicines.org.uk/emc/product/510/smpc.

25. University of Liverpool. COVID-19 Drug interaction checker. 2022. https://www.covid19-druginteractions.org/ (accessed 06 Jan 2022.

For peer review only

For peer review only - http://bmjopen.bmj.com/site/about/guidelines.xhtml
© Copyright: The University of Oxford and Oxford University Hospitals NHS Foundation Trust 2019

STANDARD PROTOCOL ITEMS: RECOMMENDATIONS FOR INTERVENTIONAL TRIALS

SPIRIT 2013 Checklist: Recommended items to address in a clinical trial protocol and related documents*

Section/item	Item No	Description	Page Number on which item is reported
**Administrative information**			
Title	1	Descriptive title identifying the study design, population, interventions, and, if applicable, trial acronym	1
Trial registration	2a	Trial identifier and registry name. If not yet registered, name of intended registry	6
	2b	All items from the World Health Organization Trial Registration Data Set	6
Protocol version	3	Date and version identifier	24
Funding	4	Sources and types of financial, material, and other support	30
Roles and responsibilitie s	5a	Names, affiliations, and roles of protocol contributors	1-3 & 31-32
	5b	Name and contact information for the trial sponsor	24
	5c	Role of study sponsor and funders, if any, in study design; collection, management, analysis, and interpretation of data; writing of the report; and the decision to submit the report for publication, including whether they will have ultimate authority over any of these activities	30
	5d	Composition, roles, and responsibilities of the coordinating centre, steering committee, endpoint adjudication committee, data management team, and other individuals or groups overseeing the trial, if applicable (see Item 21a for data monitoring committee)	30-31
**Introduction**			

For peer review only

Background and rationale	6a	Description of research question and justification for undertaking the trial, including summary of relevant studies (published and unpublished) examining benefits and harms for each intervention	8-9
	6b	Explanation for choice of comparators	8-9
Objectives	7	Specific objectives or hypotheses	9
Trial design	8	Description of trial design including type of trial (e.g. parallel group, crossover, factorial, single group), allocation ratio, and framework (e.g. superiority, equivalence, non-inferiority, exploratory)	9-10
**Methods: Participants, interventions, and outcomes**			
Study setting	9	Description of study settings (e.g. community clinic, academic hospital) and list of countries where data will be collected. Reference to where list of study sites can be obtained	11-12
Eligibility criteria	10	Inclusion and exclusion criteria for participants. If applicable, eligibility criteria for study centres and individuals who will perform the interventions (e.g. surgeons, psychotherapists)	12-13
Interventions	11a	Interventions for each group with sufficient detail to allow replication, including how and when they will be administered	15-16
	11b	Criteria for discontinuing or modifying allocated interventions for a given trial participant (e.g. drug dose change in response to harms, participant request, or improving/worsening disease)	N/A
	11c	Strategies to improve adherence to intervention protocols, and any procedures for monitoring adherence (e.g. drug tablet return, laboratory tests)	11-12
	11d	Relevant concomitant care and interventions that are permitted or prohibited during the trial	15-16
Outcomes	12	Primary, secondary, and other outcomes, including the specific measurement variable (e.g. systolic blood pressure), analysis metric (e.g. change from baseline, final value, time to event), method of aggregation (e.g. median, proportion), and time point for each outcome. Explanation of the clinical relevance of chosen efficacy and harm outcomes is strongly recommended	18 and 21-22

For peer review only - http://bmjopen.bmj.com/site/about/guidelines.xhtml

Participant timeline	13	Time schedule of enrolment, interventions (including any run-ins and washouts), assessments, and visits for participants. A schematic diagram is highly recommended (see Figure)	16-18
Sample size	14	Estimated number of participants needed to achieve study objectives and how it was determined, including clinical and statistical assumptions supporting any sample size calculations	19-20
Recruitment	15	Strategies for achieving adequate participant enrolment to reach target sample size	13-14 & 27-28
**Methods: Assignment of interventions (for controlled trials)**			
Allocation:			
Sequence generation	16a	Method of generating the allocation sequence (eg, computer-generated random numbers), and list of any factors for stratification. To reduce predictability of a random sequence, details of any planned restriction (e.g. blocking) should be provided in a separate document that is unavailable to those who enrol participants or assign interventions	14-15
Allocation concealment mechanism	16b	Mechanism of implementing the allocation sequence (e.g. central telephone; sequentially numbered, opaque, sealed envelopes), describing any steps to conceal the sequence until interventions are assigned	14-15
Implementation	16c	Who will generate the allocation sequence, who will enrol participants, and who will assign participants to interventions	14-15
Blinding (masking)	17a	Who will be blinded after assignment to interventions (e.g. trial participants, care providers, outcome assessors, data analysts), and how	15
	17b	If blinded, circumstances under which unblinding is permissible, and procedure for revealing a participant's allocated intervention during the trial	N/A
**Methods: Data collection, management, and analysis**			

Data collection methods	18a	Plans for assessment and collection of outcome, baseline, and other trial data, including any related processes to promote data quality (e.g. duplicate measurements, training of assessors) and a description of study instruments (e.g. questionnaires, laboratory tests) along with their reliability and validity, if known. Reference to where data collection forms can be found, if not in the protocol	15-17 and 22-23
	18b	Plans to promote participant retention and complete follow-up, including list of any outcome data to be collected for participants who discontinue or deviate from intervention protocols	13-14 & 27-28
Data management	19	Plans for data entry, coding, security, and storage, including any related processes to promote data quality (e.g. double data entry; range checks for data values). Reference to where details of data management procedures can be found, if not in the protocol	14
Statistical methods	20a	Statistical methods for analysing primary and secondary outcomes. Reference to where other details of the statistical analysis plan can be found, if not in the protocol	18-19
	20b	Methods for any additional analyses (e.g. subgroup and adjusted analyses)	21-23
	20c	Definition of analysis population relating to protocol non-adherence (e.g. as randomised analysis), and any statistical methods to handle missing data (e.g. multiple imputation)	20
**Methods: Monitoring**			
Data monitoring	21a	Composition of data monitoring committee (DMC); summary of its role and reporting structure; statement of whether it is independent from the sponsor and competing interests; and reference to where further details about its charter can be found, if not in the protocol. Alternatively, an explanation of why a DMC is not needed	24 & 30-31
	21b	Description of any interim analyses and stopping guidelines, including who will have access to these interim results and make the final decision to terminate the trial	18-19

Harms	22	Plans for collecting, assessing, reporting, and managing solicited and spontaneously reported adverse events and other unintended effects of trial interventions or trial conduct	20-21
Auditing	23	Frequency and procedures for auditing trial conduct, if any, and whether the process will be independent from investigators and the sponsor	20-21
**Ethics and dissemination**			
Research ethics approval	24	Plans for seeking research ethics committee/institutional review board (REC/IRB) approval	24
Protocol amendments	25	Plans for communicating important protocol modifications (e.g. changes to eligibility criteria, outcomes, analyses) to relevant parties (e.g. investigators, REC/IRBs, trial participants, trial registries, journals, regulators)	24
Consent or assent	26a	Who will obtain informed consent or assent from potential trial participants or authorised surrogates, and how (see Item 32)	18-19
	26b	Additional consent provisions for collection and use of participant data and biological specimens in ancillary studies, if applicable	18-19 and 22
Confidentialit y	27	How personal information about potential and enrolled participants will be collected, shared, and maintained in order to protect confidentiality before, during, and after the trial	18
Declaration of interests	28	Financial and other competing interests for principal investigators for the overall trial and each study site	30-31
Access to data	29	Statement of who will have access to the final trial dataset, and disclosure of contractual agreements that limit such access for investigators	18
Ancillary and post-trial care	30	Provisions, if any, for ancillary and post-trial care, and for compensation to those who suffer harm from trial participation	24
Disseminatio n policy	31a	Plans for investigators and sponsor to communicate trial results to participants, healthcare professionals, the public, and other relevant groups (e.g. via publication, reporting in results databases, or other data sharing arrangements), including any publication restrictions	24

For peer review only - http://bmjopen.bmj.com/site/about/guidelines.xhtml

	31b	Authorship eligibility guidelines and any intended use of professional writers	N/A
	31c	Plans, if any, for granting public access to the full protocol, participant-level dataset, and statistical code	N/A
**Appendices**			
Informed consent materials	32	Model consent form and other related documentation given to participants and authorised surrogates	Additional file
Biological specimens	33	Plans for collection, laboratory evaluation, and storage of biological specimens for genetic or molecular analysis in the current trial and for future use in ancillary studies, if applicable	21-23

*It is strongly recommended that this checklist be read in conjunction with the SPIRIT 2013 Explanation & Elaboration for important clarification on the items. Amendments to the protocol should be tracked and dated. The SPIRIT checklist is copyrighted by the SPIRIT Group under the Creative Commons "Attribution-NonCommercial-NoDerivs 3.0 Unported" license.

For peer review only

BMJ Open

Platform Adaptive trial of NOvel antiviRals for eArly treatMent of COVID-19 In the Community (PANORAMIC): protocol for a randomised, controlled, open-label, adaptive platform trial of community novel antiviral treatment of COVID-19 in people at increased risk of more severe disease.

Journal:	*BMJ Open*
Manuscript ID	bmjopen-2022-069176.R1
Article Type:	Protocol
Date Submitted by the Author:	17-May-2023
Complete List of Authors:	Gbinigie, Oghenekome; University of Oxford, Nuffield Department of Primary Care Health Sciences<br>Ogburn, Emma; University of Oxford Nuffield Department of Primary Care Health Sciences<br>Allen, Julie; University of Oxford, Nuffield Department of Primary Care Health Sciences<br>Dorward, Jienchi; University of Oxford, Nuffield Department of Primary Care Health Sciences; Centre for the Aids Programme of Research in South Africa,<br>Dobson, Melissa; Oxford Respiratory Trials Unit, Nuffield Department of Medicine<br>Madden, Tracie-Ann; Centre for Trials Research, Cardiff University<br>Yu, Ly-Mee; University of Oxford Nuffield Department of Primary Care Health Sciences, Department of Primary Care Health Sciences<br>Lowe, David; Institute of Immunity and Transplantation, University College London<br>Rahman, Najib; Oxford Respiratory Trials Unit, Nuffield Department of Medicine; NIHR Oxford Biomedical Research Centre<br>Petrou, Stavros; Nuffield Department of Primary Care Health Sciences<br>Richards, Duncan; University of Oxford, NDORMS<br>Hood, Kerenza; Centre for Trials Research, Cardiff University<br>Patel, Mahendra; University of Oxford Nuffield Department of Primary Care Health Sciences<br>Saville, Benjamin; Berry Consultants; Vanderbilt University Medical Center Department of Biostatistics<br>Marion, Joe; Berry Consultants<br>Holmes, Jane; University of Oxford Nuffield Department of Primary Care Health Sciences<br>Png, May Ee; University of Oxford, Nuffield Department of Primary Care Health Sciences<br>Hayward, Gail; University of Oxford, Nuffield Department of Primary Care Health Sciences<br>`Lown, Mark; University of Southampton, Primary Care and Population Sciences<br>Harris, Victoria; University of Oxford Nuffield Department of Primary

	Care Health Sciences<br>Jani, Bhautesh; University of Glasgow , 12. General Practice and Primary Care, School of Health and Wellbeing, MVLS<br>Hart, Nigel; Queen's University Belfast, School of Medicine, Dentistry and Biomedical Sciences - Centre for Public Health<br>Khoo, Saye; Department of Pharmacology, University of Liverpool<br>Rutter, Heather ; University of Oxford Nuffield Department of Primary Care Health Sciences<br>Chalk, Jem; University of Oxford Nuffield Department of Primary Care Health Sciences<br>Standing, Joseph; UCL Great Ormond Street Institute of Child Health Population Policy and Practice, Infection Inflammation and Immunology; Great Ormond Street Hospital for Children, Department of Pharmacy<br>Breuer, Judith; UCL Great Ormond Street Institute of Child Health Population Policy and Practice, Infection Inflammation and Immunology<br>Lavallee, Layla; University of Oxford Nuffield Department of Primary Care Health Sciences<br>Hadley, Elizabeth; University of Oxford Nuffield Department of Primary Care Health Sciences<br>Cureton, Lucy; University of Oxford Nuffield Department of Primary Care Health Sciences<br>Benysek, Magdalena; University of Oxford Nuffield Department of Primary Care Health Sciences<br>Andersson, Monique; Oxford University Hospitals NHS Foundation Trust, Department of Microbiology<br>Francis, Nick; University of Southampton, School of Primary Care Population Sciences and Medical Education<br>Thomas, Nicholas; Windrush Medical Practice; NIHR Clinical Research Network Thames Valley and South Midlands<br>Evans, Philip; University of Exeter, Faculty of Health and Life Sciences; National Institute for Health Research Clinical Research Network<br>van Hecke, Oliver; University of Oxford Nuffield Department of Primary Care Health Sciences<br>Koshkouei, Mona; University of Oxford, Nuffield Department of Primary Care Health Sciences<br>Coates, Maria; University of Oxford Nuffield Department of Primary Care Health Sciences<br>Barrett, Sarah; University of Oxford Nuffield Department of Primary Care Health Sciences<br>Bateman, Clare; University of Oxford Nuffield Department of Primary Care Health Sciences<br>Davies, Jennifer; University of Oxford Nuffield Department of Primary Care Health Sciences<br>Raymundo-Wood, Ivy; University of Oxford Nuffield Department of Primary Care Health Sciences<br>Ustianowski, Andrew; North Manchester General Hospital, Regional Infectious Diseases Unit<br>Nguyen-Van-Tam, Jonathan; University of Nottingham School of Medicine, Lifespan and Population Health Unit<br>Carson-Stevens, A; Cardiff University, Centre for Trials Research<br>Hobbs, Richard; University of Oxford Nuffield Department of Primary Care Health Sciences<br>Little, Paul; University of Southampton, Primary Care Research Centre<br>Butler, Christopher C.; University of Oxford, Nuffield Department of Primary Health Care Sciences
**Primary Subject Heading**:	Infectious diseases
Secondary Subject Heading:	General practice / Family practice
Keywords:	COVID-19, Clinical trials < THERAPEUTICS, VIROLOGY, PRIMARY CARE,

	THERAPEUTICS

For peer review only - http://bmjopen.bmj.com/site/about/guidelines.xhtml

Platform Adaptive trial of NOvel antiviRals for eArly treatMent of COVID-19 In the Community (PANORAMIC): protocol for a randomised, controlled, open-label, adaptive platform trial of community novel antiviral treatment of COVID-19 in people at increased risk of more severe disease.

Oghenekome A Gbinigie[1ǂ]
Emma Ogburn[1ǂ]
Julie Allen[1ǂ]
Jienchi Dorward[1,2]
Melissa Dobson[3]
Tracie-Ann Madden[4]
Ly-Mee Yu[1]
David M Lowe[5]
Najib M Rahman[3,6,7]
Stavros Petrou[1]
Duncan B Richards[8]
Kerenza Hood[4]
Mahendra G Patel[1]
Benjamin R Saville[9,10]
Joe Marion[9]
Jane Holmes[1]
May Ee Png[1]
Gail Hayward[1]
Mark Lown[11]

For peer review only

For peer review only - http://bmjopen.bmj.com/site/about/guidelines.xhtml

Victoria Harris[1]

Bhautesh Jani[12]

Nigel D Hart[13]

Saye Khoo[14]

Heather Rutter[1]

Jem Chalk[1]

Joseph F Standing[15,16]

Judith Breuer[15]

Layla Lavallee[1]

Elizabeth Hadley[1]

Lucy Cureton[1]

Magdalena Benysek[1]

Monique Andersson[17]

Nick Francis[11]

Nicholas P B Thomas[18,19,20]

Philip Evans[21,22]

Oliver van Hecke[1]

Mona Koshkouei[1]

Maria Coates[1]

Sarah Barrett[1]

Clare Bateman[1]

Jennifer C Davies[1]

Ivy Raymundo-Wood[1]

Andrew Ustianowski[23]

Jonathan S Nguyen-Van-Tam[24]

For peer review only

Andrew Carson-Stevens[4]

F.D. Richard Hobbs[1†]

Paul Little[11†]

Christopher C Butler[1†]

Corresponding author - Christopher C Butler christopher.butler@phc.ox.ac.uk

Postal address: Nuffield Department of Primary Care Health Sciences, University of Oxford, Woodstock Road, Oxford, OX2 6GG

[‡] Joint first authors

[†]Joint senior authors

Author affiliations

1. Nuffield Department of Primary Care Health Sciences, University of Oxford, Oxford, UK
2. Centre for the AIDS Programme of Research in South Africa (CAPRISA), University of KwaZulu–Natal, Durban, South Africa.
3. Oxford Respiratory Trials Unit, Nuffield Department of Medicine, University of Oxford, UK
4. Centre for Trials Research, Cardiff University, UK
5. Institute of Immunity and Transplantation, University College London, London, UK
6. Oxford NIHR Biomedical Research Centre, UK
7. Chinese Academy of Medicine Oxford Institute, University of Oxford, UK
8. Nuffield Department of Orthopaedics, Rheumatology and Musculoskeletal Sciences, University of Oxford, Oxford, UK
9. Berry Consultants, Austin, Texas, USA
10. Department of Biostatistics, Vanderbilt University School of Medicine, Tennessee, USA

For peer review only

11. Primary Care Research Centre, University of Southampton, Southampton, UK

12. General Practice and Primary Care, School of Health and Wellbeing, MVLS, University of Glasgow, UK

13. School of Medicine, Dentistry and Biomedical Sciences, Queen's University Belfast, Northern Ireland, UK

14. Department of Pharmacology, University of Liverpool, Liverpool, UK

15. Infection Inflammation and Immunology, UCL Great Ormond Street Institute of Child Health, London, UK

16. Department of Pharmacy, Great Ormond Street Hospital for Children, London, UK

17. Department of Microbiology, Oxford University Hospitals NHS Foundation Trust, Oxford, UK

18. Windrush Medical Practice, Witney, UK

19. National Institute for Health and Care Research, Thames Valley and South Midlands Clinical Research Network, UK

20. Royal College of General Practitioners, London, UK

21. Faculty of Health and Life Sciences, University of Exeter, UK

22. National Institute for Health and Care Research Clinical Research Network, UK

23. Regional Infectious Diseases Unit, North Manchester General Hospital, Manchester, UK

24. Lifespan and Population Health Unit, University of Nottingham School of Medicine, Nottingham, UK

For peer review only

Word count: 5393/4000

For peer review only - http://bmjopen.bmj.com/site/about/guidelines.xhtml

ABSTRACT

Introduction: There is an urgent need to determine the safety, effectiveness and cost-effectiveness of novel antiviral treatments for COVID-19 in vaccinated patients in the community at increased risk of morbidity and mortality from COVID-19.

Methods and analysis: PANORAMIC is a UK-wide, open-label, prospective, adaptive, multi-arm platform, randomised clinical trial that evaluates antiviral treatments for COVID-19 in the community. A master protocol governs the addition of new antiviral treatments as they become available, and the introduction and cessation of existing interventions via interim analyses. The first two interventions to be evaluated are molnupiravir (Lagevrio) and nirmatrelvir/ritonavir (Paxlovid). Eligibility criteria: community-dwelling within five days of onset of symptomatic COVID-19 (confirmed by PCR or lateral flow test), and either (1) aged 50 years and over, or (2) aged 18-49 years with qualifying co-morbidities. Registration occurs via the trial website and by telephone. Recruitment occurs remotely through the central trial team, or in person through clinical sites. Participants are randomised to receive either usual care or a trial drug plus usual care. Outcomes are collected via a participant-completed daily electronic symptom diary for 28 days post randomisation. Participants and/or their Trial Partner are contacted by the research team after days 7, 14 and 28 if the diary is not completed, or if the participant is unable to access the diary. The primary efficacy endpoint is all-cause, non-elective hospitalisation and/or death within 28 days of randomisation. Multiple pre-specified interim analyses allow interventions to be stopped for futility or superiority based on pre-specified decision criteria. A prospective economic evaluation is embedded within the trial.

For peer review only - http://bmjopen.bmj.com/site/about/guidelines.xhtml

Ethics and dissemination: Ethical approval granted by South Central–Berkshire REC number: 21/SC/0393; IRAS project ID: 1004274. Results will be presented to policymakers and at conferences, and published in peer-reviewed journals.

Trial registration number: ISRCTN 30448031; EudraCT number: 2021-005748-31

296/300

For peer review only

For peer review only - http://bmjopen.bmj.com/site/about/guidelines.xhtml

STRENGTHS AND LIMITATIONS

- PANORAMIC is a platform trial, enabling interventions to be added as the trial progresses, with interim analyses allowing interventions to be dropped as soon as pre-specified criteria for superiority or futility are met, or for safety concerns.
- In addition to recruitment by investigators at research sites, the research can be delivered "direct-to-patient" through recruitment by a centralised team, with remote consent, follow-up, and delivery of study medication to participants' homes, thereby not limiting participation to where people live or receive their health care.
- A national inclusion and diversity strategy has been employed to actively promote the trial across the four UK nations to diverse communities and people from all backgrounds collaborating with the NIHR Clinical Research Network and equivalent networks in UK devolved administrations.
- The open-label design means that it is not possible to quantify the contribution of any placebo-effect to treatment effects, but is more closely reflective of real-world practice.

INTRODUCTION

The development and roll-out of national Coronavirus disease 2019 (COVID-19) vaccination schemes has been transformative in reducing disease severity and to a lesser extent SARS-CoV-2 transmission. [1-3] Despite this, the emergence of new variants and waning immunity have led to intermittent surges in COVID-19 cases and hospitalisations. [4] The implementation of effective COVID-19 treatments therefore remains a critical management strategy and may be of great importance if future vaccine-escaping variants emerge. A number of drugs have been trialled as re-purposed COVID-19 community treatments with evidence that some

For peer review only - http://bmjopen.bmj.com/site/about/guidelines.xhtml

should not be used for this indication [5,6] while others are likely to be beneficial. [7,8] Directly-acting antiviral drugs are an important therapeutic approach, but evidence is limited.

Two new antiviral options are molnupiravir (Lagevrio) and nirmatrelvir/ritonavir (Paxlovid), with others being developed. Molnupiravir is a prodrug; the ribonucleoside analogue β-d-N4-hydroxycytidine (NHC) is metabolised to NHC-triphosphate in cells, which when integrated introduces catastrophic hypermutation. [9] Paxlovid is a combination of nirmatrelvir and ritonavir; nirmatrelvir inhibits the activity of the SARS-CoV-2 3-CL protease that is necessary for viral replication [10], and ritonavir significantly slows the clearance of nirmatrelvir.[11]

Initial trials of molnupiravir and nirmatrelvir/ritonavir for COVID-19 have demonstrated safety and efficacy. [12,13] However, these trials were in unvaccinated patients prior to the omicron SARS-CoV-2 variant becoming prevalent, and it is not clear if there are particular subgroups of patients who should be prioritised for treatment. Furthermore, the impact on viral load, antiviral resistance and emergence of new variants requires further evaluation, and cost-effectiveness of these agents at scale is as yet unknown. The impact on long COVID is also yet to be assessed. Nevertheless, these encouraging efficacy trials, and the likelihood that further plausible treatments will become available and require evaluation, justifies a large-scale, ongoing, pragmatic evaluation of antiviral treatments for use in the community in a largely vaccinated population, while current variants are circulating, to rapidly generate robust evidence for guiding decisions about widespread deployment.

For peer review only - http://bmjopen.bmj.com/site/about/guidelines.xhtml

We therefore established an adaptive multi-arm platform trial with a master protocol to test whether novel antiviral agents are safe, effective and cost-effective treatments for people in the community with COVID-19 who are at increased risk of an adverse outcome.

Objective

To assess the effectiveness and cost effectiveness of novel antiviral treatments in reducing all-cause, non-elective hospitalisation and/or death within 28 days of randomisation among patients with test-positive COVID-19 in the community and who are at increased risk of requiring hospital treatment.

METHODS AND ANALYSIS

Trial Design

The Platform Adaptive trial of Novel antiviRals for eArly treatment of COVID-19 in the Community (PANORAMIC) is an open-label, prospective, adaptive platform, randomised clinical trial in community care.

A multi-arm 'platform trial' is a clinical trial that allows for multiple treatments for the same disease to be tested simultaneously under a single master protocol. Pre-specified adaptations allow interventions to be added to the trial, or stopped for futility or superiority whilst the trial is in progress through pre-specified interim analyses. [14 15] Participants are randomly assigned to either usual care, or usual care plus a trial intervention. Usual care represents the standard care that participants would receive via the National Health Service (NHS), and is largely supportive, apart from for those at the highest risk of an adverse outcome. [16]

For peer review only - http://bmjopen.bmj.com/site/about/guidelines.xhtml

The master protocol defines *a priori* decision rules to allow for dropping a treatment for futility or declaring a treatment superior to usual care. [17] If at an interim analysis, usual care plus an antiviral is deemed superior to usual care alone for the primary endpoint of all-cause, non-elective hospitalisation and/or death within 28 days of randomisation, the superior treatment may be incorporated into usual care as the new standard of care. Cost-effectiveness will also be assessed. A subset of participants is additionally enrolled into a virology sub-study, and are asked to provide nasopharyngeal swabs and fingerpick blood samples at intervals over the 14 days following recruitment.

The first and second antivirals to be evaluated in PANORAMIC are molnupiravir[18] and nirmatrelvir/ritonavir, respectively.

Patient and Public Involvement (PPI)

PPI contributors contribute to refining the study question, design, implementation, interpretation and dissemination of findings. At trial conception, the aims and design of the study were discussed with members of the public who had experience of COVID-19, either personally or through household members, and who were at higher risk of complications from COVID-19. PPI groups supporting the trial include an ethnically diverse main study PPI group who have advised on patient facing documents and study processes, and have helped to draft easy read versions of study documents. In addition, bespoke PPI groups established in Northern Ireland, Scotland and Wales have advised on data capture and recruitment processes specific to their local health systems, and will contribute to advise on dissemination. Two PPI contributors sit on the Trial Steering Committee to help guide trial progress. A co-investigator has a specific remit for community engagement, developing and implanting initiatives with the support of pharmacy networks to ensure uptake especially in areas of

For peer review only - http://bmjopen.bmj.com/site/about/guidelines.xhtml

higher social deprivation and among minority ethnic groups: feedback about all aspects of the trial is received from this community engagement program.

Study Setting

The trial is implemented by the University of Oxford Primary Care and Vaccines Collaborative Clinical Trials Unit (PCV-CTU)[19] with further support from the Oxford Respiratory Trials Unit and the Centre for Trials Research, Cardiff University, supported by the National Institute of Health and Care Research Clinical Research Network, the National Institute of Health and Care Research, and the Department of Health and Social Care (and equivalents in devolved administrations).

The PCV-CTU is able to act as a central recruiting site, and PANORAMIC Hubs act as clinical recruitment sites. PANORAMIC Hubs are clinical sites that include GP sites as single practices or a federation of practices that are able to operate under a single site agreement with a Principal Investigator to undertake study procedures as detailed in the master protocol. Hubs can include GP practices, community trusts, and other healthcare providers. Potential participants can be referred to Hubs by other healthcare facilities for screening. As well as recruiting patients through routine consultations, Hubs perform database searches for COVID-19 positive test results in registered patients who are clinically vulnerable (see Table 1), and invite them to take part in the trial. All mandated study procedures can be conducted remotely, in keeping with the prevailing self-isolation advisory governmental guidance for patients with COVID-19 in the community. [20]

For peer review only - http://bmjopen.bmj.com/site/about/guidelines.xhtml

Table 1: Criteria considered to make a potential participant at higher risk of worse outcomes from COVID-19

○ Chronic respiratory disease (including chronic obstructive pulmonary disease (COPD), cystic fibrosis and asthma requiring at least daily use of preventative and/or reliever medication)<br>○ Chronic heart or vascular disease<br>○ Chronic kidney disease<br>○ Chronic liver disease<br>○ Chronic neurological disease (including dementia, stroke, epilepsy)<br>○ Severe and profound learning disability<br>○ Down's syndrome<br>○ Diabetes mellitus (Type 1 or Type 2)<br>○ Immunosuppression: primary (e.g., inherited immune disorders resulting from genetic mutations, usually present at birth and diagnosed in childhood) or secondary due to disease or treatment (e.g., sickle cell, HIV, cancer, chemotherapy)<br>○ Solid organ, bone marrow and stem cell transplant recipients<br>○ Morbid obesity (BMI >35 kg/m²)<br>○ Severe mental illness<br>○ Care home resident<br>○ Judged by recruiting medically qualified professional, research nurse, nurse prescriber, prescribing pharmacist, dependent on the Intervention Specific Appendix for the specific antiviral involved, to be clinically vulnerable

Eligibility criteria

The inclusion criteria are: patient or their legal representative is able and willing to provide informed consent; patient presenting with symptoms attributable to COVID-19 starting within the past five days and ongoing; patient has a positive SARS-CoV-2 test (lateral flow test and/or PCR) up to two days before symptom onset and randomisation; and, patient is aged ≥50 years or aged 18-49 years with an underlying chronic health condition considered to make them clinically vulnerable (see Table 1). Exclusion criteria are: patient currently admitted to hospital (inpatient); patient previously randomised in the PANORAMIC trial; and, patient currently participating in a clinical trial of a therapeutic agent for acute COVID-19. Additional exclusion criteria specific to each intervention arm, if any, are listed in the Intervention Specific Appendices (ISAs) of trial arms within the master protocol. Patients

For peer review only - http://bmjopen.bmj.com/site/about/guidelines.xhtml

must be eligible for at least two arms (Usual Care and at least one novel antiviral intervention).

Study procedures

Recruitment

The entire recruitment process can be done remotely as well as in person. Potential participants can register via the trial website, through a free-phone telephone call to the central trial team, or via a PANORAMIC hub.

Informed consent, screening and enrolment

Eligibility is assessed at a PANORAMIC Hub, other NHS healthcare provider, or by the central clinical trial team, by a suitably trained and experienced medically qualified professional, research nurse, nurse prescriber or prescribing pharmacist, as determined by the regulator and specified in the ISA for the specific antiviral involved.

Prospective participants are provided with written, pictorial and/or verbal versions of the Patient Information Sheet (PIS), detailing the nature of the trial and the known side-effects/risks involved in taking part. Participants provide consent to participate through a two-way discussion (apart from with those who lack capacity to do this) either face-to-face or by a telephone/video call. Prospective participants with capacity and being recruited in-person provide written informed consent (see additional file 1). Consent forms for participants recruited in-person via PANORAMIC Hubs are filed in participants' medical notes, with a printed copy given to the participant. Participants recruited remotely provide consent using an electronic consent form that is held securely on the trial database.Participants can either download their consent form, or a hard copy of the consent form is posted to them. .

For peer review only - http://bmjopen.bmj.com/site/about/guidelines.xhtml

Prospective participants lacking capacity to consent are only eligible if they live in a care home. If the recruiting healthcare professional considers that a patient in a care home lacks capacity to provide consent for themselves, then a personal or professional legal representative (England and Wales only) is asked to provide consent in-person or remotely.

Participants who are unable or too unwell to complete baseline information or respond to surveys for themselves can identify a Trial Partner to assist them in: completing the initial screening questionnaire and baseline information; completing the informed consent forms; and, completing the electronic symptom diary (see 'follow-up' section). A letter is issued to Trial Partners, informing them of the study and notifying them that they have been nominated for this role by the prospective participant.

Randomisation and blinding

Participants are randomised using a secure, fully validated, and compliant web-based randomisation system embedded within Spinnaker (a data entry system), with binary stratification by age (<50 years vs ≥ 50 years) and vaccination status (yes vs no). Participants are randomised to one trial arm using fixed equal allocation ratios corresponding to the number of eligible arms in the trial. For example, if there are two active interventions (A & B), the allocation ratio will be 1:1:1 for Usual Care, active A, active B (respectively), such that 33% of participants are randomised to Usual Care. If there are three active interventions, the allocation ratio will be 1:1:1:1, such that 25% of participants are randomised to Usual Care. As this is a nationwide, individually randomised trial that aims to include large numbers of participants, individual participant characteristics and infecting strain types of SARS-CoV-2 are expected to be equally distributed between trial arms.

For peer review only - http://bmjopen.bmj.com/site/about/guidelines.xhtml

PANORAMIC is an open-label trial. The participant, legal representative (if applicable), and recruiting clinician know the participant's allocation. The trial team and recruiting clinicians are kept blind to emerging results of interim analyses. Only the unblinded statisticians and the independent members of the Data and Safety Monitoring Committee (DSMC) have access to unblinded interim results corresponding to a given intervention, until such time as a decision is made to close the intervention.

Baseline assessments

During screening and enrolment, participants and/or recruiting clinicians record baseline data including: date of birth; sex; ethnicity; vaccination status; qualifying co-morbidities; symptoms and severity; a measure of their health-related quality of life (EuroQoL EQ-5D-5L); [21] number of household contacts; and, whether they have received a monoclonal antibody treatment for COVID-19.

Interventions

PANORAMIC trial is testing novel antiviral agents that have the potential to be widely used to treat COVID-19 in the community. Each agent is fully specified in an ISA. The antiviral drugs are couriered to participants, typically within 24 hours of randomisation. Pharmacies can supply antivirals to participants via community pharmacy services or online pharmacy services. The antivirals can also be collected from a pharmacy by the participant (or someone on their behalf, with appropriate infection control measures).

For peer review only - http://bmjopen.bmj.com/site/about/guidelines.xhtml

PANORAMIC is a randomised controlled, open-label, pragmatic trial. [22,23] The control arm is Usual Care. Usual Care can include antiviral treatment available to individual patients in routine care in the NHS. [24] In the UK, patients at highest risk are able to access antiviral treatments directly from the NHS via COVID Medicine Delivery Units (CMDUs) and analogous organisations; otherwise, in the absence of complicated infection (e.g. bacterial super-infection), Usual Care in the NHS is generally supportive. [24] Participants assigned to an intervention arm additionally receive the usual care through the NHS that they would ordinarily have received, had they not participated in the trial. The trial team are not involved in making clinical or clinical management decisions for participants. Participants receiving a monoclonal antibody infusion or an antiviral agent as part of their usual care were eligible to receive a (different) antiviral through the trial. However, those at highest risk of an adverse outcome were informed that they were eligible for access to antiviral treatment through NHS services.

Follow-up

Following randomisation, participants in the intervention arm receive a participant pack containing: the allocated antiviral agent; an information booklet; a participant card detailing how the medication should be administered, precautions and safety guidance; a medication appendix providing further information about the allocated intervention; an emergency card with a phone number with a 24 hour phone line to access an on-call clinician for safety concerns; and, a pregnancy test to be used by participants of child-bearing potential for certain interventions.

All participants are emailed a link each day to an online symptom diary and are asked to complete it daily for 28 days. Participants are asked: to rate a variety of symptoms (such as

For peer review only - http://bmjopen.bmj.com/site/about/guidelines.xhtml

For peer review only

fever, cough, breathlessness and fatigue) on an ordinal scale (e.g. 'no problem,' 'mild problem,' 'moderate problem' or 'major problem'); whether they have been hospitalised or required contact with health and social services; how they are feeling on a scale of zero to 10 (zero being the worst one can imagine, and 10 being the best one can imagine); whether they feel fully recovered; whether they are taking over-the-counter medication; whether the number of people in the household has changed; confirm whether they have taken the antiviral agent (if applicable); and, at fortnightly intervals the EQ-5D-5L to assess their health-related quality of life. The central trial team calls participants/Trial Partners with no internet access and those who have not completed their diary for at least two consecutive days before days 7, 14 and 28.

All participants receive a phone call from the trial team on Day 2 of the trial to confirm receipt of trial materials, confirm consent and understanding of follow-up procedures, and to answer any queries. Participants receiving an antiviral agent receive additional safety calls from members of the trial team, to determine whether participants are experiencing adverse effects, and, if applicable, to ensure that participants who are physiologically capable of becoming pregnant and who are not using highly effective contraception confirm a negative pregnancy test result prior to starting the intervention. The exact schedule of safety calls is intervention-dependent, and outlined in each ISA.

To investigate the impact of trial interventions on the longer-term effects of COVID-19, we contact participants at three and six months after randomisation to ascertain wellbeing, persistence of symptoms perceived to be related to the index COVID-19 illness, and longer-term consequences. Participants' medical record data may additionally be accessed up to twelve months following enrolment to gather follow up data from enrolment to 6 months.

For peer review only - http://bmjopen.bmj.com/site/about/guidelines.xhtml

Sources of routinely collected data (e.g., NHS Digital) may also be used to follow-up participants for up to 10 years.

Study Outcomes

The primary endpoint is all-cause, non-elective hospitalisation and/or death within 28 days of randomisation, ascertained through patient/Trial Partner report, and/or patient medical records. Secondary endpoints include: time to self-reported recovery defined as the first instance that a participant report feeling fully recovered from the illness; duration of symptoms; symptom recurrence; daily rating of feeling well reported by participants; healthcare service use; participant reported new COVID-19 infections in their household; safety and cost-effectiveness outcomes; symptoms; and, well-being at three and six months (with determination of proportion reporting symptoms perceived to be related to long COVID) from randomisation.

Data Collection and Management

Data are entered into electronic case report forms (CRFs) by the participant, their Trial Partner, or a Hub team member, using Spinnaker. Spinnaker is an online secure, FDA part 11B compliant, data entry system, which is designed to collect sensitive data, such as participant and Trial Partner contact details. All identifiable participant data are encrypted using the Advanced Encryption Standard. Data are stored on a secure cloud hosted server physically located in London, UK. Participant and Trial Partner data will be kept and stored securely for as long as required by the trial and reviewed on annual basis.

Statistical Methods

Primary endpoint Analysis

For peer review only - http://bmjopen.bmj.com/site/about/guidelines.xhtml

Details of the statistical design and methods are described in a Master Statistical Analysis Plan (M-SAP) and Adaptive Design Report (ADR). The primary endpoint analysis is a Bayesian logistic regression model of the primary endpoint comparing a given intervention versus Usual Care, adjusting for age, co-morbidity status, and vaccination status.

The trial design incorporates multiple pre-specified interim analyses that allow each intervention to stop early for futility or superiority. If the Bayesian posterior probability of beneficial treatment effect (alternative hypothesis) is greater than or equal to a pre-specified threshold at an interim or final analysis, the null hypothesis (no beneficial intervention effect) is rejected, and the intervention is deemed superior to Usual Care with respect to Hospitalisation/Death. The decision criteria are defined in the ADR and control the Type I error at the traditional 0.05 two-sided level for each intervention, accounting for multiple interim analyses. As described in the ADR, the pre-specified interim analyses may be bypassed for a given intervention at the discretion of the blinded Trial Management Group (TMG) in the event of a fast accrual rate. The success thresholds at final and interim analysis are prespecified and dependent on the number of interim analyses, which is a function of the speed of enrolment. .The ADR also contains extensive simulations to explore the performance of the adaptive design, including power and Type I error. Subgroup analyses are performed according to age group, baseline comorbidity status, severity of symptoms at baseline, duration of symptoms at baseline, use of an inhaled corticosteroid steroid at randomisation or during 28 days of follow-up, swab positivity status (PCR positive versus Lateral Flow Device positive), vaccination status, and COVID-19 risk category (as per the UK government description). Details of subgroup analyses can be found in the statistical analysis plan. All statistical analyses of primary and some secondary outcome data analyses are performed by Berry Consultants and the University of Oxford. Berry Consultants is based in the USA; as such they will not receive identifiable trial data.

For peer review only - http://bmjopen.bmj.com/site/about/guidelines.xhtml

Sample size

The master protocol specifies a maximum sample of approximately 5300 participants per arm, which provides approximately 90% power for detecting a 33% relative reduction in the risk of hospitalisation/death in an experimental arm relative to Usual Care, based on the assumption of an underlying 3% hospitalisation/death rate in the Usual Care arm, and an intervention lowering the hospitalisation/death rate to 2%. However, an intervention-specific appendix may define an alternative maximum sample size for an intervention based on alternative assumptions for a given intervention or based on the relevant state of the pandemic. For example, if the severity of COVID-19 weakens and the aggregate (blinded) primary endpoint event rate is lower than expected, the maximum sample size may be increased to ensure sufficient statistical power.

Primary analysis population

For each intervention, the primary analysis population includes all concurrently randomised patients who were eligible to be randomised to an antiviral agent (concurrent and eligible), who fulfil the eligibility criteria, and who have had the opportunity to complete 28 days of follow-up. Eligible participants will be analysed according to the group they were randomised to regardless of deviation from protocol.

Safety Monitoring

Symptoms, potential medication side-effects and Serious Adverse Events (SAE) are collected from participant daily diaries, calls to participants/Trial Partners, face-to-face visits with Hub clinicians, medical records, notes reviews, and data extracts from hospital and primary care medical records from all UK devolved administrations.

For peer review only - http://bmjopen.bmj.com/site/about/guidelines.xhtml

A risk assessed and proportionate approach to safety monitoring is adopted for each antiviral included in the trial. In line with the Summary of Product Characteristics or Investigator Brochure, the risks and the safety profile for each antiviral agent are assessed, and the mitigation and monitoring procedures are detailed in the ISA. All safety procedures will be according to University of Oxford Primary Care Clinical Trials Unit pharmacovigilance Standard Operating Procedures.

For each antiviral agent, we only collect Adverse Events (AEs), other than those pre-specified symptoms collected via the participant diaries, if and when specified in the relevant ISA. For certain interventions, pregnancy occurring within 28 days of first intervention administration is recorded as an AE of Special Interest. All-cause hospitalisation and/or death is the primary outcome, and these data are captured in CRFs. Serious adverse events (SAEs) other than hospitalisation or death due to COVID-19 are reported for all antiviral agents over the follow up period. Hospitalisations for pre-existing conditions, including elective procedures planned prior to trial entry, which has not worsened, do not contribute to our primary outcome, and do not constitute SAEs.

A risk assessment and monitoring plan is prepared before opening recruitment to each antiviral agent and is reviewed as necessary over the course of the trial to reflect significant changes to the protocol or outcomes of monitoring activities. Monitoring is performed by the Primary Care Clinical Trials Unit (PC-CTU). The level of monitoring required is informed by the risk assessment.

For peer review only - http://bmjopen.bmj.com/site/about/guidelines.xhtml

VIROLOGY SUB-STUDY

A subset of patients from the intervention and comparator arms of the trial are invited to participate in a virology sampled cohort for additional virological testing. The primary aim of the virology sampled cohort is to determine whether the antiviral treatment under study reduces viral load to undetectable levels sooner, and to explore the effect of antiviral treatment on development of antiviral resistance. The sub-study primary outcome is SARS-CoV-2 viral load at Day 7. Secondary outcomes include SARS-CoV-2 viral load Days 0-7 and Day 14; SARS-CoV-2 viral genetic whole genome sequence at Day 1, Day 5 (+/- 1 day) and Day 14 (+/- 1 day) and SARS-CoV-2 antibodies at Day 1, Day 5 (+/- 1 day), and Day 14 (+/- 1 day); and to identify any common viral genetic mutations (occurring in greater than 1% of patients) in patients receiving novel antiviral(s).

The eligibility criteria are as for participants taking part in the main trial, but with an additional exclusion criterion: participants who are within 3 months of receiving a non-trial anti-SARS-CoV-2 antibody therapy are ineligible.

Up to approximately three hundred participants from each trial intervention arm and the Usual Care arm are recruited into the voluntary virology sampled cohort. The first 30 patients enrolling from each trial arm undergo intensive daily viral load monitoring, and are asked to provide daily nasopharyngeal self-taken swabs for seven days, and an additional nasopharyngeal swab on Day 14 (+/- 1 day). For participants in intervention arms, the first sample will be taken immediately prior to commencing anti-viral treatment (Day 1). The remaining 270 from each arm in the virology samples cohort have less intensive viral load

For peer review only - http://bmjopen.bmj.com/site/about/guidelines.xhtml

monitoring, and are asked to provide three nasopharyngeal swabs: one prior to starting treatment, one on Day 5 (+/- 1 day) and one on Day 14 (+/- 1 day).

All participants are asked to take three finger prick dried blood spot samples: one pre-treatment, one on Day 5 (+/- 1 day) and one on Day 14 (+/- 1 day). Participants consenting to take part in the virology sampled cohort are sent CE-IVD approved (that is, compliant with the European In-Vitro Diagnostic Devices Directive) sampling kits for nasopharyngeal sampling, dried blood spot sampling, pre-paid postage and packaging, to post samples to the virology processing site. Samples taken at home should be posted to the trial team within 3 days of sampling, and ideally within 24 hours.

HEALTH ECONOMIC EVALUATION

A prospective economic evaluation is embedded within the trial design to assess the cost-effectiveness of each antiviral from an NHS and Personal Social Services (PSS) perspective. The resource inputs associated with embedding each trial antiviral treatment into routine clinical practice are estimated. Broader resource use is drawn from linked routine health data – encompassing primary care encounters, hospital inpatient/day case admissions, outpatient visits, and accident and emergency attendances. Unit costs are valued using national reference tariffs and attached to resource inputs to generate a compound total NHS and PSS cost per trial participant over the trial time horizon. EQ-5D-5L data are converted using standard algorithms into utility scores for quality-adjusted life year (QALY) estimation. Cost-effectiveness is expressed as incremental cost per QALY gained. [25] Secondary expressions of cost-effectiveness include incremental cost per hospitalisation and/or death prevented over 28 days. Bivariate regression of costs and measures of health consequence, with multiple imputation of missing data, will be conducted to generate within-trial estimates of

For peer review only - http://bmjopen.bmj.com/site/about/guidelines.xhtml

incremental cost-effectiveness. Sensitivity analyses will assess the impact of areas of uncertainty surrounding components of the economic evaluation. If economic outcomes are non-convergent within the trial follow-up period, then extrapolation of cost-effectiveness through decision-analytic modelling will be considered, drawing upon the best available information from the literature to supplement the trial data. Specific plans for the economic evaluation are outlined in a pre-specified health economics analysis plan.

ETHICS, APPROVALS, MONITORING AND DISSEMINATION:

The trial has been approved by the University of Oxford Research Governance Ethics and Assurance Team as study sponsor, the South Central–Berkshire Research Ethics Committee (REC number: 21/SC/0393) of the Health Research Authority (HRA), and the UK Medicines and Healthcare products Regulatory Agency (MHRA). All participants provide informed consent, online or by telephone, before participation. All participants completing the 28 day follow up are provided with a £10 voucher in recognition of their contribution to the study. The University of Oxford as sponsor has a specialist insurance policy in place, which would operate in the event of any participant suffering harm as a result of their involvement in the research (Newline Underwriting Management Ltd, at Lloyd's of London). NHS indemnity operates in respect of the clinical treatment that is provided.

An independent Data Monitoring and Safety Committee (DMSC) reviews emerging data provided by the Statistical Analysis Committee (SAC) and communicates key decisions to the Trial Steering Committee (TSC), which in turn advises the Trial Management Group (TMG) and also provides trial oversight.

For peer review only

It is expected that trial results will be published in peer-reviewed journals and relevant findings presented at national and international conferences.

Trial Status

PANORAMIC was registered on the ISRCTN registry (ISRCTN 30448031) on 3rd November 2021. Enrolment started on 8th December 2021. By 17 September 2022, 26,285 participants have been recruited. Protocol v.5.0, 09 May 2022 (see additional file 2).

DISCUSSION

Summary

Despite high uptake of vaccination against COVID-19 in many countries, the disease remains prevalent, with many patients continuing to experience considerable morbidity and require treatment in hospital. We describe a platform randomised trial to evaluate antiviral therapeutic agents for use by people at higher risk from COVID-19 in the community with confirmed acute, symptomatic SARS-CoV-2 infection.

Comparison with other studies of novel antiviral agents for community treatment of COVID-19

A phase 3 placebo-controlled, randomised trial of molnupiravir recruited 1,433 COVID-19 outpatients in over 20 countries, with a primary efficacy endpoint of all-cause hospitalisation or death within 29 days of enrolment (MOVe-OUT trial). [26] The authors found that treatment with molnupiravir reduced the risk of hospitalisation or death compared with placebo (risk difference, −3.0 %; 95% CI: −5.9 % to −0.1%). [26] Adverse events occurred with similar frequency in molnupiravir and placebo groups (30.4 % and 33.0 %, respectively), as did adverse events deemed to be related to the trial regimen (8.0 % and 8.4%, respectively). No

For peer review only

deaths were attributed to the trial treatment (one death in the molnupiravir group and nine deaths in the placebo group).

As in the PANORAMIC trial, participants were at higher risk of an adverse illness course, received a five-day course of molnupiravir at a dose of 800 mg twice daily, and received the intervention within five days of symptom onset. However, the trial recruited unvaccinated patients; the vast majority of the UK adult population are multiply-vaccinated (primary course plus boosters). [27] Furthermore, Delta, Gamma and Mu variants accounted for the majority of SARS-CoV-2 variants in the MOVe-OUT trial [28], whereas the predominant variant in circulation in the UK has been Omicron since December 2021. [29] PANORAMIC additionally incorporates an assessment of the impact of antiviral drugs on viral load and markers of viral resistance.

In a phase 2-3 randomised, placebo-controlled trial of 2,246 outpatients with COVID-19 from the United States (41%), Europe (30%), South America (12.3%), Asia (14%) and Africa (0.6%), at higher risk of an adverse illness course, treatment with nirmatrelvir/ritonavir resulted in a 5.8% absolute risk reduction in the primary outcome of COVID-19 related hospitalisation and all-cause death within 28 days (0.72% and 6.53% respectively, risk difference -5.81%, 95% CI: -7.78 % to -3.84%, p<0.0001). [13] Viral load was significantly reduced by treatment with nirmatrelvir/ritonavir (adjusted mean difference of −0.868 log10 copies per millilitre, 95% CI: -1.074 to -0.6615, p<0.001). The incidence of adverse events was similar in both groups, and all thirteen deaths occurred in the placebo group. The trial population was again unvaccinated, and therefore distinct from the UK population taking part in the PANORAMIC trial.

For peer review only - http://bmjopen.bmj.com/site/about/guidelines.xhtml

Strengths and Limitations

The platform design, informed by the experience of the PRINCIPLE trial, [30] allows PANORAMIC to add new interventions to the trial as they become available; this increases the efficiency of the trial as multiple interventions can be assessed by a single trial platform without having to set up a new trial each time a new intervention for this condition requires evaluation. Pre-specified interim analyses allow randomisations to interventions to be stopped as soon as pre-specified criteria for superiority or futility are met, potentially reducing time to trial conclusions. This ensures the trial's relevance in the face of rapidly evolving pandemic circumstances.

Deploying antimicrobials of any kind at scale raises the question of their possible impact on antimicrobial resistance. A virology sub-study has been incorporated in PANORAMIC, which allows us to estimate virological endpoints, as well as facilitating careful evaluation of potential harms associated with antiviral treatment, such as the development of antiviral resistance and emergence of new variants.

Cost effectiveness of novel antivirals is as yet unknown, but is critically important to considerations of widespread deployment of expensive: PANORAMIC aims to fill this gap in the evidence base for these agents.

Traditionally, primary care research implementation has followed a similar model to hospital-based studies, in which the "participant comes to the research." In this approach, potential participants are invited to participate if they receive their health care or live in the proximity to the research site. The capacity of PANORAMIC for recruitment of eligible people from almost anywhere in the UK, not limited by where people live or receive their health care,

For peer review only - http://bmjopen.bmj.com/site/about/guidelines.xhtml

allows the "research to be taken to the patient." This is particularly important, given that participants are ill and probably highly infectious.

The trial has been designed to be minimally burdensome for participants; all trial procedures are possible remotely, from registration, to eligibility checks, to receiving trial medications and virology sub-study materials by courier. This has facilitated rapid recruitment to the trial, with over 26,000 participants recruited to date. PANORAMIC strives to be a truly representative trial, with participants from various backgrounds recruited nationally from all four UK nations. A proactive outreach strategy has been employed, led by the trial's national pharmacy, and inclusion and diversity lead, with the support of UK-wide pharmacy networks, to help to promote the trial to diverse communities and to those disproportionately affected by COVID-19. This includes people from ethnic minority backgrounds and those living in areas of higher deprivation, traditionally known to be under-represented in clinical trials. The proportion of PANORAMIC participants in the molnupiravir versus usual care comparison older than 50 years who are from ethnic minorities is approximately 5%, which is not dissimilar to that in the English and Welsh general population (just over 6%). [31] However, we recognise that recruitment to the trial requires prospective participants to navigate the registration process, which might mean that people from certain groups, such as non-English speaking populations, may be less likely to enrol in the trial.

In addition to the primary outcome that is measured at 28 days, PANORAMIC evaluates longer-term outcomes at three and six months, which will help ascertain the effect of antiviral treatment on long COVID. Long COVID, defined as symptoms beyond four weeks after index illness [32] may affect between 10% [33] and 43.4% [34] of patients with COVID-19, and is

For peer review only - http://bmjopen.bmj.com/site/about/guidelines.xhtml

characterised by a range of physical and psychological symptoms. [32] Thus far, we do not know whether novel antiviral treatments reduce symptoms associated with the acute illness over the longer term.

Some may consider the open-label design of the trial a weakness. The lack of blinding means that we cannot estimate the proportion of any positive effect from the treatment that results from a possible placebo effect. Performance bias is more likely to affect outcomes that are considered subjective, such as symptom or wellness ratings. However, the objective primary outcome in PANORAMIC (non-elective hospitalisation and/or death) is unlikely to be affected by a placebo effect, as hospital admission is a clinical decision, and the virology sub-study will also provide a helpful pointer as to whether the treatments are effective.[18] Furthermore, comparison with usual care is in keeping with pragmatic trial design and more closely reflective of real-world practice. [35] As placebos are not used in clinical care, the results of an open-label trial are more likely to reflect what would happen if the intervention were introduced into routine clinical practice, [35] additionally enabling a more realistic assessment of cost effectiveness. Findings from the pragmatic, open label PRINCIPLE trial have found no difference in outcome measures that rely on participants' self-reported recovery between participants allocated to usual care and usual care plus a study drug. [5 30 36]

For peer review only - http://bmjopen.bmj.com/site/about/guidelines.xhtml

Acknowledgements

The authors would like to especially thank all participants in the study and acknowledge the work and support of all participating general practices, NHS COVID-19 treatment services and other health and social care organisations supporting the trial. We would also like to thank our PPI contributors, the Trial Steering Committee, the Data Monitoring and Safety Committee, primary care and other colleagues in the National Institute of Health and Care Research Clinical Research Network, and the National Institute of Health and Care Research. CCB acknowledges part support as Senior Investigator of the National Institute of Health and Care Research, the NIHR Community Healthcare Medtech and In-Vitro Diagnostics Co-operative (MIC), and the NIHR Health Protection Research Unit on Health Care Associated Infections and Antimicrobial Resistance. FDRH acknowledges his part-funding from the NIHR Applied Research Collaboration (ARC OxTV) and the NIHR Community Healthcare Medtech and In-Vitro Diagnostics Co-operative (MIC). GH is funded by and NIHR Advanced Fellowship and by the NIHR Community Healthcare Medtech and In-Vitro Diagnostics Co-operative (MIC). JD is funded by the Wellcome Trust PhD Programme for Primary Care Clinicians (216421/Z/19/Z). SP receives support as a NIHR Senior Investigator (NF-SI-0616-10103) and from the UK NIHR Applied Research Collaboration Oxford and Thames Valley. OVH is supported by an NIHR Development and Skills Enhancement Award. KH receives support as a HCRW Senior Research Leader and the Centre for Trials Research receives infrastructure funding from Health & Care Research Wales and Cancer Research UK. OAG is funded by ECRAID-Prime (grant number 101046109).

Data Monitoring and Safety Committee Independent members:

Prof Deborah Ashby (Chair)

For peer review only - http://bmjopen.bmj.com/site/about/guidelines.xhtml

Prof Benjamin Fisher

Prof Simon Gates

Prof Gordon Taylor

Prof Martin Underwood

Trial Steering Committee Independent members

Philip Hannaford (Chair)

Ms Corina Cheeks

Prof Ranjit Lall

Prof Alastair Hay

Prof William Hollingworth

Prof Matthew Sydes: Independent observer

Prof Mike Moore: Independent observer

Authors' contributions

CCB and JSN-V-T conceived the study. CCB is the Chief Investigator. PL, FDRH are co-Chief Investigators. CCB, PL, and FDRH decided to publish the paper. BRS, L-MY, JH, MD, CCB, FDRH, PL, GH, OAG, JD, NMR, DBR, SP, DML, JFS, KH, PE, OVH and ML provided input to the trial design. EO, JA, PE, LL, EH, LC, MB, MC, SB, CB, JCD, AC-S and IR-W are responsible for study implementation and acquisition of data. CCB, OAG, GH, FDRH, JH, L-MY, JD, JM, BRS, EO, JA, MGP, SP PL, KH, NMR, JFS and SP drafted the manuscript. HR leads the clinical team. L-MY, BRS, JH, VH and JM contribute to statistical analysis. SK, DBR, NMR and MD provide input to safety evaluations, monitoring, and drug interactions. MGP is the National Pharmacy, and Inclusion and Diversity Lead for the trial. SP and MEP run the economic evaluation. JFS, DML and JB lead the virology sub-study. JC

For peer review only

leads on the information systems. MB leads data management. CCB, PL, OAG, NMR, SP, DBR, KH, MGP, BRS, EO, JD, DML, SK, NF, NPBT, PE, JFS, JB, JA, MD, T-AM, MEP, GH, ML, BJ, NDH, JC, EH, LC, MB, MA, OvH, AU, MK, L-MY and FDRH are members of the Trial Management Group supporting site recruitment, activity and delivery. OAG and CCB produced the first draft of the manuscript. All authors critically revised the manuscript. All authors are contributing to the conduct of the trial.

Funding statement:

This work is supported by the UKRI/NIHR, Grant number NIHR135366. Virology team members are partly supported by an MRC COVID modelling grant, Grant number MR/W015560/1. JD is funded by the Wellcome Trust PhD Programme for Primary Care Clinicians (216421/Z/19/Z). For the purpose of Open Access, the author has applied a CC BY public copyright license to any Author Accepted Manuscript version arising from this submission. NDH was funded by the Northern Ireland Clinical Research Network, Public Health Agency. OAG is funded by ECRAID-Prime (grant number 101046109).

Competing interests:

JSN-V-T was seconded to the Department of Health and Social Care, England (DHSC) from October 2017 to March 2022. The views expressed in this paper are those of its authors and not necessarily those of DHSC. JSN-V-T reports a lecture fee from Gilead Sciences Ltd (manufacturer of remdesivir) and a paid Influenza Advisory Board for F. Hoffmann-La Roche (manufacturer of tocilizumab), both after March 2022. KH is a member of the following NIHR committees: HTA General Committee, HTA Funding Strategy Group, Research Professors Funding Committee. KH is co-investigator on the grant provided by UKRI/NIHR, Grant number NIHR135366 (subcontract from University of Oxford to Cardiff University). KH received a grant from AstraZeneca to support a trial of Evusheld for the

For peer review only

prevention of COVID in high risk individuals (to Cardiff University). KH is an independent member of the IDMC for the OCTAVE-DUO trial of vaccines for COVID in high risk individuals (unpaid). DML has received grants/contracts from LifeArc, Medical Research Council, Bristol Myers Squibb and Blood Cancer UK. DML received personal fees/honoraria for a lecture from Biotest UK, for an educational video from Gilead and for a 'round table' discussion with Merck. SP is co-investigator on the grant provided by UKRI/NIHR, Grant number NIHR135366. DBR has received consulting fees from OMASS therapeutics. DBR has a leadership/fiduciary role in the Heal-COVID trial TMG. BRS reports grant money paid to his employer (Berry Consultants, LLC) from The University of Oxford, from the Sponsor's grant from the UKRI/NIHR, per the statistical design and analyses for the PANORAMIC trial. GH reports that the NIHR funded this study. ML reports funding directly from the PANORAMIC trial (NIHR). ML reports being a RAPIS-TEST (NIHR EME) DMC member. JM reports that this is part of his consulting work for Berry Consultants. SK reports research funding from GSK, ViiV Healthcare, Ridgeback Biotherapeutics, Vir and Merck unrelated to this work. SK reports speaker's fees from ViiV Healthcare and participation on ViiV Healthcare, Pfizer advisory boards. SK reports receiving donation of drugs for clinical studies from ViiV Healthcare, Toyama and GSK. JFS reports receiving research grants from MRC (MR/X004724/1), Wellcome, NIHR, DNDi, Gates and MRC (MR/W015560/1) paid to his institution. JFS reports receiving Pharmacometric consultancy fees from Adrenomed Ltd paid to his institution. JFS reports participation on a Data Safety Monitoring board/Advisory Board for GSK Sotrovimab paediatric programme. MA reports receiving grants from BTRU – GEMS, Janssen – Cartography , Pfizer – Myst, Prenetics, Dunhill Medical Trust, BMA Trust – Kathleen Harper Fund and Antibiotic Research UK – all paid to the institution. MA reports receiving consultancy fees from Prenetics and OxDx. MA reports a planned patent for Ramanomics. MA reports participation on a Data Safety Monitoring board/Advisory Board

For peer review only - http://bmjopen.bmj.com/site/about/guidelines.xhtml

for Prenetics. MA has an unpaid leadership/fiduciary role in the E3 Initiative. NPBT reports his current affiliations with RCGP and NIHR TVSM. NPBT reports a payment for a single episode of participation on the MSD advisory board in July 2021, prior to any knowledge or planning of this trial. OVH reports receiving an NIHR Development and Skills Personal Award. OVH reports receiving consulting fees for MINDGAP BV, with the fees paid to Oxford University lnnovation Limited. OVH reports unpaid participation on a Data Safety Monitoring board/Advisory Board for The CHIldren with COugh Cluster Randomised Controlled Trial (CHICO). OVH has an unpaid leadership/fiduciary role in the British Society of Antimicrobial Chemotherapy. CB reports full employment with the Nuffield Department of Primary Care Health Sciences. AU reports receiving consulting fees and payment/honoraria from Merck/MSD and Gilead Sciences. FDRH reports receiving expenses reimbursed for talks on COVID monoclonal antibodies. PL reports support from the NIHR for the grant to do the PANORAMIC study. NF reports receiving consulting fees from Abbott Diagnostics and GSK, a presentation fee from Abbott Diagnostics, and has stocks in Synairgen PLC.

For peer review only

Word Count: 5393 words

References

1. Bergwerk M, Gonen T, Lustig Y, et al. Covid-19 breakthrough infections in vaccinated health care workers. *New England Journal of Medicine* 2021;385(16):1474-84.
2. Paris C, Perrin S, Hamonic S, et al. Effectiveness of mRNA-BNT162b2, mRNA-1273, and ChAdOx1 nCoV-19 vaccines against COVID-19 in healthcare workers: an observational study using surveillance data. *Clinical Microbiology and Infection* 2021;27(11):1699. e5-99. e8.
3. Pilishvili T, Gierke R, Fleming-Dutra KE, et al. Effectiveness of mRNA Covid-19 vaccine among US health care personnel. *New England Journal of Medicine* 2021;385(25):e90.
4. The Telegraph. Covid hospital admissions are surging again – is this the start of a new wave? 2022 [Available from: https://www.telegraph.co.uk/global-health/science-and-disease/covid-hospital-admissions-surging-start-new-wave/ accessed 10th May 2022.
5. Butler CC, Yu LM, Dorward J, et al. Doxycycline for community treatment of suspected COVID-19 in people at high risk of adverse outcomes in the UK (PRINCIPLE): a randomised, controlled, open-label, adaptive platform trial. *Lancet Respir Med* 2021;9(9):1010-20. doi: 10.1016/S2213-2600(21)00310-6 [published Online First: 20210727]
6. Principle Trial Collaborative Group. Azithromycin for community treatment of suspected COVID-19 in people at increased risk of an adverse clinical course in the UK (PRINCIPLE): a randomised, controlled, open-label, adaptive platform trial. *Lancet* 2021;397(10279):1063-74. doi: 10.1016/S0140-6736(21)00461-X [published Online First: 2021/03/08]
7. Yu L-M, Bafadhel M, Dorward J, et al. Inhaled budesonide for COVID-19 in people at higher risk of adverse outcomes in the community: interim analyses from the PRINCIPLE trial. *Medrxiv* 2021
8. RECOVERY Collaborative Group. Dexamethasone in hospitalized patients with Covid-19. *New England Journal of Medicine* 2021;384(8):693-704.
9. Malone B, Campbell EA. Molnupiravir: Coding for catastrophe. *Nature Structural & Molecular Biology* 2021;28(9):706-08.
10. Pfizer. Pfizer's Novel COVID-19 Oral Antiviral Treatment Candidate Reduced Risk of Hospitalization or Death by 89% in Interim Analysis of Phase 2/3 EPIC-HR Study 2021 [Available from: https://www.pfizer.com/news/press-release/press-release-detail/pfizers-novel-covid-19-oral-antiviral-treatment-candidate accessed 10th May 2022.
11. Extance A. Covid-19: What is the evidence for the antiviral Paxlovid? *bmj* 2022;377
12. Singh AK, Singh A, Singh R, et al. Molnupiravir in COVID-19: a systematic review of literature. *Diabetes & Metabolic Syndrome: Clinical Research & Reviews* 2021;15(6):102329.
13. Hammond J, Leister-Tebbe H, Gardner A, et al. Oral nirmatrelvir for high-risk, nonhospitalized adults with COVID-19. *New England Journal of Medicine* 2022;386(15):1397-408.
14. Berry SM, Connor JT, Lewis RJ. The platform trial: an efficient strategy for evaluating multiple treatments. *Jama* 2015;313(16):1619-20.
15. Butler CC, Connor JT, Lewis RJ, et al. Answering patient-centred questions efficiently: response-adaptive platform trials in primary care. *British Journal of General Practice* 2018;68(671):294-95.
16. National Institute for Health and Care Excellence. COVID-19 rapid guideline: managing COVID-19 2021 [Available from: https://www.nice.org.uk/guidance/ng191 accessed 26th April 2023.
17. Woodcock J, LaVange LM. Master protocols to study multiple therapies, multiple diseases, or both. *New England Journal of Medicine* 2017;377(1):62-70.
18. Butler CC, Hobbs FR, Gbinigie OA, et al. Molnupiravir plus usual care versus usual care alone as early treatment for adults with COVID-19 at increased risk of adverse outcomes

For peer review only

For peer review only - http://bmjopen.bmj.com/site/about/guidelines.xhtml

(PANORAMIC): an open-label, platform-adaptive randomised controlled trial. *The Lancet* 2022
19. University of Oxford Primary Care Clinical Trials Unit. PANORAMIC trial 2021 [Available from: https://www.panoramictrial.org/ accessed 16th March 2022.
20. UK Health Security Agency. People with symptoms of a respiratory infection including COVID-19 2022 [Available from: https://www.gov.uk/guidance/people-with-symptoms-of-a-respiratory-infection-including-covid-19#what-to-do-if-you-have-a-positive-covid-19-test-result accessed 10th May 2022.
21. Herdman M, Gudex C, Lloyd A, et al. Development and preliminary testing of the new five-level version of EQ-5D (EQ-5D-5L). *Quality of life research* 2011;20(10):1727-36.
22. Moustgaard H, Clayton GL, Jones HE, et al. Impact of blinding on estimated treatment effects in randomised clinical trials: meta-epidemiological study. *bmj* 2020;368
23. Anand R, Norrie J, Bradley JM, et al. Fool's gold? Why blinded trials are not always best. *Bmj* 2020;368
24. National Institute for Health and Care Excellence. COVID-19 rapid guideline: managing COVID-19 [NG191] 2021 [updated 13th April 2022. Available from: https://www.nice.org.uk/guidance/ng191 accessed 30th April 2022.
25. Van Hout B, Janssen M, Feng Y-S, et al. Interim scoring for the EQ-5D-5L: mapping the EQ-5D-5L to EQ-5D-3L value sets. *Value in health* 2012;15(5):708-15.
26. Jayk Bernal A, Gomes da Silva MM, Musungaie DB, et al. Molnupiravir for oral treatment of Covid-19 in nonhospitalized patients. *New England Journal of Medicine* 2022;386(6):509-20.
27. GOV.UK. Vaccinations in the United Kingdom 2022 [Available from: https://coronavirus.data.gov.uk/details/vaccinations accessed 10th May 2022.
28. Merck. Merck and Ridgeback's Investigational Oral Antiviral Molnupiravir Reduced the Risk of Hospitalization or Death by Approximately 50 Percent Compared to Placebo for Patients with Mild or Moderate COVID-19 in Positive Interim Analysis of Phase 3 Study 2021 [Available from: https://www.merck.com/news/merck-and-ridgebacks-investigational-oral-antiviral-molnupiravir-reduced-the-risk-of-hospitalization-or-death-by-approximately-50-percent-compared-to-placebo-for-patients-with-mild-or-moderat/ accessed 4th May 2022.
29. Burki TK. Omicron variant and booster COVID-19 vaccines. *The Lancet Respiratory Medicine* 2022;10(2):e17.
30. Hayward G, Butler CC, Yu LM, et al. Platform Randomised trial of INterventions against COVID-19 In older peoPLE (PRINCIPLE): protocol for a randomised, controlled, open-label, adaptive platform, trial of community treatment of COVID-19 syndromic illness in people at higher risk. *BMJ Open* 2021;11(6):e046799. doi: 10.1136/bmjopen-2020-046799 [published Online First: 20210618]
31. Office for National Statistics. 2011 Census 2011 [Available from: https://www.nomisweb.co.uk/census/2011/data_finder accessed 26th October 2022.
32. National Institute for Health and Care Excellence. COVID-19 rapid guideline: managing the long-term effects of COVID-19 2020 [updated 11 November 2021. Available from: https://www.nice.org.uk/guidance/NG188 accessed 10th October 2022.
33. Greenhalgh T, Knight M, Buxton M, et al. Management of post-acute covid-19 in primary care. *bmj* 2020;370
34. Cirulli E, Barrett KMS, Riffle S, et al. Long-term COVID-19 symptoms in a large unselected population. *medrxiv* 2020
35. MacPherson H. Pragmatic clinical trials. *Complementary therapies in medicine* 2004;12(2-3):136-40.
36. Dorward J, Yu LM, Hayward G, et al. Colchicine for COVID-19 in the community (PRINCIPLE): a randomised, controlled, adaptive platform trial. *Br J Gen Pract* 2022;72(720):e446-e55. doi: 10.3399/BJGP.2022.0083 [published Online First: 20220630]

For peer review only - http://bmjopen.bmj.com/site/about/guidelines.xhtml

For peer review only

For peer review only - http://bmjopen.bmj.com/site/about/guidelines.xhtml

Platform Adaptive trial of NOvel antiviRals for eArly treatMent of covid-19 In the Community: PANORAMIC

REC Number: 21/SC/0393 **IRAS Number: 1004274**

Chief Investigator: Professor Christopher Butler **Participant ID:** ______________________

CONSENT FORM

Thank you for completing the screening questionnaire, you have passed screening for the trial.

Please read the Participant Information Sheet (PIS) if you haven't already done so, and if you are willing to participate please select 'Yes', TYPE your FIRST and LAST names below and then click Submit

If you agree, please select 'Yes' to confirm that you have read and understood the following:

		YES	NO
1	I confirm I have read and understood the information sheet version number ____-________dated __ __ / __ __ __/ __ __ __ __ for the above study. I have had the opportunity to ask questions and had these answered satisfactorily.		
2	I understand my participation is voluntary and that I am free to withdraw at any time, without giving any reason, and without my medical care or legal rights being affected.		
3	I understand that if I chose to withdraw data already collected will continue to be used and I or my GP may be contacted if there are further questions regarding side-effects from trial treatments.		
4	I understand that I will be randomised to receive either: standard care plus an antiviral treatment or standard care, and that I will not be able to choose which I will receive.		
5	I understand that relevant sections of my GP and hospital medical notes and data collected during the study may be looked at by members of the research team and individuals from University of Oxford, both during and for up to 10 years after the scheduled follow-up period. It may also be reviewed by relevant people from regulatory authorities and from NHS Organisation(s). I give permission for these individuals to have access to my records which identify me by name.		
6	I understand that my personal information may be shared with University of Dundee's Health Informatics Centre and that my date of birth and/or my NHS number (or equivalent UK NHS Identifier) will be shared with NHS Digital**,** electronic Data Research and Innovation Service (eDRIS), The Secure Anonymised Information Linkage (SAIL) Databank or Health and Social Care Northern Ireland (HSC Business Services Organisation/HSC Trusts) (HSC NI) to enable them to supply the study team with additional healthcare data about me, which is relevant to the trial. The data supplied by NHS Digital, eDRIS, SAIL or HSC NI is linked by the research team to the data collected during my participation in the trial. I am free to withdraw my consent for data linkage with NHS Digital, eDRIS, SAIL or HSC NI at any time and it will not affect my ongoing care.		
7	I understand that members of the research team may view my general practice and hospital medical records, including the summaries of my medical records (e.g. Summary Care Record (SCR), Emergency Care Summary (ECS), The GP Summary, Northern Ireland		

For peer review only

For peer review only - http://bmjopen.bmj.com/site/about/guidelines.xhtml

NUFFIELD DEPARTMENT OF PRIMARY CARE HEALTH SCIENCES | Primary Care Clinical Trials Unit | PANORAMIC Platform Adaptive trial of NOvel antiviRals for eArly treatMent of COVID-19 In the Community

	Electronic Care Record, and the Welsh Clinical Portal to check my medication, allergies, adverse reactions, and additional information to make sure that it is safe for me to take trial medication. I give permission for these individuals to access my medical records for this purpose.		
8	I consent to being contacted by the research team for the purposes of trial follow up (up to 6 months) and I understand that this will require me to provide my contact details to the research team.		
9	I consent to my GP and/or Care Home being informed of my participation within the study, and I understand that the trial team may contact my GP about my ongoing participation in the trial.		
10	I understand that the information collected about me may be shared in a form that cannot identify me with commercial companies to support the licensing of trial treatments, within the UK and abroad.		
11	I agree to take part in the trial.		
	For participants capable of being pregnant (regardless of current contraception methods) *(to show only for those who meet this criterion in the screening form)*		
12	I agree to taking a pregnancy test prior to taking the trial treatment.		
13	I understand that I must use reliable methods of contraception (as specified in the PIS appendices). I agree to provide information requested on any pregnancy, including pregnancy outcome, occurring within 28-days following first administration of the IMP, as requested by the MHRA. I understand that if I report a pregnancy the Sponsor will report this to The UK Teratology Information Service (UKTIS).		
	ADDITIONAL (optional, not required for study participation)	**YES**	**NO**
14	I agree to provide the research team with the contact details of my Trial Partner. I confirm my Trial partner is aware of their role and willing to answer questions.		
15	I agree to take part in the Virology Sampled Cohort.		
	For Participants Agreeing to take part in Virology Study		
16	I agree to donate blood and nasopharyngeal samples. I consider these samples a gift to the University of Oxford, and I understand I will not gain any direct personal or financial benefit from them. I understand that even if I withdraw from the above study, the samples collected from me may still be used in the study analysis.		

If you are the participant completing the consent form, please provide your signature below

Participant Signature: ______________________________

For peer review only - http://bmjopen.bmj.com/site/about/guidelines.xhtml

NUFFIELD DEPARTMENT OF PRIMARY CARE HEALTH SCIENCES
Primary Care Clinical Trials Unit

PANORAMIC
Platform Adaptive trial of NOvel antiviRals for eArly treatMent of COVID-19 In the Community

First Name: ______________________ ______________________

Last Name:

Date: __ __ / __ __ / __ __ __ __

If the participant has provided verbal consent, but they are unable to complete the consent form due to lack of online access, too unwell, too frail or consent is completed via the telephone (the participant must have capacity), please provide:

1. **Name of the participant:**

First Name: ______________________ **Last Name:** ______________________

Date: __ __ / __ __ / __ __ __ __

2. **Signature of person completing the form:**

First Name: ______________________ **Last Name:** ______________________

Role: Trial partner/trial team member/Health Care Professional

Date: __ __ / __ __ / __ __ __ __

If participant lacks capacity to give consent:

I have read the information (or had it read to me), had an opportunity to ask questions and signed the *Legal Representative Letter*. I understand that the patient will be asked to confirm their consent as soon as they have the capacity to do so and that if they wish, they will be able to withdraw from the trial without it affecting their medical care.

Participant:

Name: ______________________ Date: __ __ / __ __ / __ __ __ __

I believe that if they were able to, the patient would wish to take part in this trial.

______________________ ______________________

PRINTED name of Legal Representative **Signature of Legal Representative**

Today's date __ __ / __ __ / __ __ __ __

For peer review only - http://bmjopen.bmj.com/site/about/guidelines.xhtml

NUFFIELD DEPARTMENT OF PRIMARY CARE HEALTH SCIENCES
UNIVERSITY OF OXFORD

Primary Care Clinical Trials Unit

PANORAMIC
Platform Adaptive trial of NOvel antiviRals for eArly treatMent of COVID-19 In the Community

Relationship to participant (as confirmed in the signed *Legal Representative Letter*)

____

You will have the opportunity to print a copy of the consent form after submission. Please contact the study team if you would like a copy sent to you.

By submitting, I confirm that I am the person whose name is stated above.

If you have any questions about consent or the trial, please contact the study team:

Tel: 08081 560017   Email panoramic@phc.ox.ac.uk

For peer review only

For peer review only - http://bmjopen.bmj.com/site/about/guidelines.xhtml

NUFFIELD DEPARTMENT OF PRIMARY CARE HEALTH SCIENCES — Primary Care Clinical Trials Unit

PANORAMIC
Platform Adaptive trial of NOvel antiviRals for eArly treatMent of COVID-19 In the Community

Trial Title: **P**latform **A**daptive trial of **NO**vel antivi**R**als for e**A**rly treat**M**ent of covid-19 **I**n the **C**ommunity

Internal Reference Number / Short title: PANORAMIC
Ethics Ref: 21/SC/0393
IRAS Project ID: 1004274
EudraCT Number: 2021-005748-31
Date and Version No: 9 May 2022 Version 5.0

For peer review only

**Chief Investigator and trial leader:**	Professor Christopher Butler, Department of Primary Care Health Sciences University of Oxford
**Co-Principal Investigator and Co-trial lead:**	Professor Paul Little, School of Primary Care, Population Sciences and Medical Education, University of Southampton
**Co-Principal Investigator and Co-trial lead:**	Professor Richard Hobbs, Department of Primary Care Health Sciences University of Oxford
**Investigators:**	Dr Ly-Mee Yu, Primary Care Clinical Trials Unit, Department of Primary Care Health Sciences, University of Oxford
	Dr Emma Ogburn, Primary Care Clinical Trials Unit, Department of Primary Care Health Sciences, University of Oxford
	Dr Gail Hayward, Department of Primary Care Health Sciences, University of Oxford
	Dr Hannah Swayze, Primary Care Clinical Trials Unit, Department of Primary Care Health Sciences, University of Oxford
	Ms Julie Allen, Primary Care Clinical Trials Unit, Department of Primary Care Health Sciences, University of Oxford
	Dr Jienchi Dorward, Department of Primary Care Health Sciences, University of Oxford
	Dr Oliver van Hecke, Department of Primary Care Health Sciences, University of Oxford
	Dr Kome Gbinigie, Department of Primary Care Health Sciences, University of Oxford
	Professor Stavros Petrou, Department of Primary Care Health Sciences, University of Oxford

For peer review only - http://bmjopen.bmj.com/site/about/guidelines.xhtml
© Copyright: The University of Oxford and Oxford University Hospitals NHS Foundation Trust 2019

Professor Najib Rahman, Oxford Respiratory Trials Unit, Nuffield Department of Medicine of the University of Oxford

Ms Melissa Dobson, Oxford Respiratory Trials Unit, Nuffield Department of Medicine of the University of Oxford

Professor Duncan Richards, Oxford Clinical Trials Research Unit, Nuffield Department of Orthopaedics, Rheumatology and Musculoskeletal Sciences, University of Oxford

Dr Monique Anderson, Oxford University Hospitals NHS Foundation Trust, University of Oxford

Dr Ben Saville, Berry Consultants, Texas, USA, & Department of Biostatistics, Vanderbilt University School of Medicine, Tennessee, USA

Professor Saye Khoo, Department of Pharmacology, University of Liverpool

Professor Kerry Hood, Centre for Trials Research, Cardiff University

Dr Tracie-Ann Madden, Centre for Trials Research, Cardiff University

Professor Andrew Ustianowski, Infectious Disease and Tropical Medicine, Manchester University NHS Foundation Trust

Dr Bhautesh Jani, General Practice & Primary Care, University of Glasgow

Professor Joseph Standing, Infection, Immunity & Inflammation, University College London

Professor Judith Breuer, Infection, Immunity & Inflammation, University College London

Dr David Lowe, Infection, Immunity & Inflammation, University College London

Professor Tim McHugh, Infection, Immunity & Inflammation, University College London

Professor Mahendra G Patel, Primary Care Clinical Trials Unit, Department of Primary Care Health Sciences, University of Oxford
Dr Mark Lown, Department of Medicine, University of Southampton

Professor Nick Francis, School of Primary Care, Population Sciences and Medical Education, University of Southampton

Professor Nigel Hart, School of Medicine, Dentistry and Biomedical Sciences, Queens University Belfast

For peer review only - http://bmjopen.bmj.com/site/about/guidelines.xhtml
© Copyright: The University of Oxford and Oxford University Hospitals NHS Foundation Trust 2019

NUFFIELD DEPARTMENT OF PRIMARY CARE HEALTH SCIENCES | Primary Care Clinical Trials Unit | PANORAMIC Platform Adaptive trial of NOvel antiviRals for eArly treatMent of COVID-19 In the Community

Dr Nicholas PB Thomas, National Institute for Health Research (NIHR) Clinical Research Network, National Institute for Health Research, London, UK and Royal College of General Practitioners, London, UK

Sponsor: University of Oxford
Joint Research Office
1st floor, Boundary Brook House
Churchill Drive, Headington, OX3 7GB

Funder: Department of Health and Social Care (DHSC), National Institute of Health Research (NIHR)

Chief Investigator Signature (Professor Christopher Butler):

Lead Trial Statistician Signature (Dr Ly-Mee Yu):

No potential conflict of interest

Confidentiality Statement

This document contains confidential information that must not be disclosed to anyone other than the Sponsor, the Investigator Team, HRA, host organisation, and members of the Research Ethics Committee and Regulatory Authorities unless authorised to do so.

The term '**central clinical team'** refers to a team of medically qualified professionals and research nurses located at the PC-CTU and ORTU.

The term '**central trial team'** refers to the team responsible for the day-to-day conduct of the trial, which includes the central clinical team, as well as other non-clinical trial staff.

PC-CTU SOPs will be used for all aspects of PANORAMIC.

See *supplementary material B* for **Key Trial Contacts**.

For peer review only - http://bmjopen.bmj.com/site/about/guidelines.xhtml
© Copyright: The University of Oxford and Oxford University Hospitals NHS Foundation Trust 2019

NUFFIELD DEPARTMENT OF PRIMARY CARE HEALTH SCIENCES
Primary Care Clinical Trials Unit
PANORAMIC Platform Adaptive trial of NOvel antiviRals for eArly treatMent of COVID-19 In the Community

Platform Adaptive trial of NOvel antiviRals for eArly treatMent of covid-19 In the Community (PANORAMIC): Overview

Background: Despite high uptake of vaccination against COVID-19, the disease remains prevalent in the UK and in many countries around the world, with many patients continuing to experience considerable morbidity and require treatment in hospital. There is therefore an urgent need to identify treatments for COVID-19 for use in the community early on in the illness that speeds recovery and prevents the need for hospital admission.

Aims and objectives:

Main trial: This protocol describes a platform randomised trial of antiviral therapeutic agents for use by clinically vulnerable people in the community with confirmed acute symptomatic SARS-CoV-2 infection.
Virology sampled cohort: The primary aim of the virology sampled cohort is to determine whether antiviral treatment in the community reduces viral load to undetectable levels more quickly than untreated patients and to explore antiviral treatment on potential development of antiviral resistance.

Platform trial: A "platform trial" is a trial in which multiple treatments for the same disease can be tested simultaneously, and in which new interventions can be added or replace existing ones during the course of the trial in accordance with pre-specified criteria.

Interventions: Participants will be randomised to receive either Usual Care (see Usual Care Intervention Specific Appendix (ISA), or an antiviral agent in addition to Usual Care (see ISA for each antiviral agent under trial). Potential participants can be included if they are eligible to be randomised to at least one novel antiviral agent, as well as the Usual Care arm.

Eligibility: Participants who meet the following inclusion criteria may be eligible to take part in the main trial:

- Participant or their legal representative is able and willing to provide informed consent
- Symptoms attributable to COVID-19 starting within the past 5 days and ongoing
- A positive PCR or lateral flow SARS-CoV-2 test
- Aged ≥50 years OR aged 18-49 years with any known underlying chronic health condition considered to make them clinically vulnerable

Adaptive randomisation: Participants in the main trial will be randomised to one trial arm using equal allocation ratios corresponding to the number of eligible arms in the trial. Pre-specified decision criteria allow for dropping an antiviral agent for futility, declaring an antiviral superior, or adding a new antiviral to be tested. If at any point an antiviral agent is deemed superior to the Usual Care, the superior antiviral may become part of Usual Care arm as the new standard of care according to recommended treatment guidelines and changing effects of Usual Care will be taken into account in the analysis.

Outcomes:
Main trial: The primary outcome will be all-cause, non-elective hospitalisation and/or death within 28 days of randomisation. Secondary outcomes will include time to self-reported recovery defined as

For peer review only - http://bmjopen.bmj.com/site/about/guidelines.xhtml
© Copyright: The University of Oxford and Oxford University Hospitals NHS Foundation Trust 2019

the first instance that a participant report feeling fully recovered from the illness; duration of symptoms; symptom recurrence; daily rating of feeling well reported by participants; healthcare service use; participant reported household infection rate; safety outcomes and cost-effectiveness outcomes; symptoms and well-being at three and six months (with determination of proportion with Long COVID) from randomisation.

Virology sampled cohort: The primary outcome will be SARS-CoV-2 viral load at Day 7. Secondary outcomes will include SARS-CoV-2 viral load Days 0-7 and Day 14; SARS-CoV-2 viral genetic whole genome sequence at Day 1, Day 5 and Day 14 and SARS-CoV-2 antibodies at Day 1, Day 5, and Day 14; and to identify any common genetic mutations in patient receiving novel antiviral(s).

See *supplementary material C* for details of objectives and outcome measures.

Efficient trial design: Depending on the drug licensing status and available safety data, all enrolment (screening, informed consent, eligibility review and baseline data) can be done either by PANORAMIC Hubs or by the central trial team, with follow-up procedures (daily diary, data capture of hospitalisations and deaths) conducted remotely with participants using the trial website or a telephone call with the trial team. Randomisation will be online and automatic, following eligibility confirmation.

PANORAMIC Hubs: These will include GP Sites, Community Trusts, and other health service providers, including government agencies e.g., UK Health Security Agency, who will actively identify potential participants and invite them to take part. Potential participants may be referred to Hubs by other NHS facilities for possible inclusion in the trial. A medically qualified professional , research nurse, nurse prescriber or prescribing pharmacist (as specified in the ISA for the specific antiviral involved) from the Hub will complete all recruitment procedures, screening, baseline, informed consent, and eligibility review. Participants will be provided with a participant pack (containing the antiviral agent, if randomised to this arm), either issued by the Hub or sent directly to participants homes. Hubs will be able to store and issue trial antiviral agents. The Hubs will also allow additional safety monitoring visits where required and as defined in the ISA. A Principal Investigator (PI) at each Hub will provide trial oversight for participants recruited via the Hub.

Central recruitment: A central trial team will also be able to recruit and randomise participants and a participant pack containing an antiviral agent (if randomised to this arm) will be sent directly to participants homes.

Data to be recorded: Demographic features including ethnicity will be captured at baseline. In the online daily diary (completed each day for 28 days) and during telephone calls, participants or their Trial Partners will rate the severity of symptoms including how well they are feeling, record contacts with the health services (including hospital admission), record trial medication use, resource use, and new infections in the household. Follow-up beyond 28 days after randomisation will be by accessing electronic medical records and by participant questionnaire for information relevant to the longer-term consequences of COVID-19 at three and six months from randomisation. To investigate the impact of trial interventions on the longer-term effects of COVID-19, we will also remotely follow-up participants, for up to 10 years.

For peer review only

For peer review only - http://bmjopen.bmj.com/site/about/guidelines.xhtml
© Copyright: The University of Oxford and Oxford University Hospitals NHS Foundation Trust 2019

Numbers to be randomised: An estimated maximum of approximately 5300 participants per arm will be required to provide approximately 90% power for detecting a 33% relative reduction in the hospitalisation/death in an experimental arm relative to Usual Care, based on the assumption of an underlying 3% combined hospitalisation/death rate in the Usual Care arm, and an intervention lowering the hospitalisation/death rate to 2%.

To enquire about the trial, contact the PANORAMIC Trial Team:

PANORAMIC Trial
Nuffield Department of Primary Care Health Sciences
Radcliffe Primary Care
Radcliffe Observatory Quarter, Woodstock Road
Oxford
OX2 6GG

Email Address: panoramic@phc.ox.ac.uk

For peer review only

For peer review only - http://bmjopen.bmj.com/site/about/guidelines.xhtml
© Copyright: The University of Oxford and Oxford University Hospitals NHS Foundation Trust 2019

NUFFIELD DEPARTMENT OF PRIMARY CARE HEALTH SCIENCES Primary Care Clinical Trials Unit

PANORAMIC Platform Adaptive trial of NOvel antiviRals for eArly treatMent of COVID-19 In the Community

TABLE OF CONTENTS

1. BACKGROUND and RATIONALE 11
1.1 Aims and objectives 11
2. TRIAL DESIGN AND PROCEDURES 11
2.1 Participant identification 12
2.1.1 Trial participants 12
2.1.2 Inclusion criteria 12
2.1.3 Exclusion criteria 12
2.1.3.1 Additional exclusion criteria for virology sampled cohort only: 12
2.2 Trial procedures 13
2.2.1 Informing potential participants about the trial 13
2.2.2.2 Central recruitment 14
2.2.2.3 Virology sampled cohort recruitment 14
2.3 Screening 14
2.4 Informed consent 15
2.5 Eligibility assessment 16
2.6 Randomisation 17
2.7 Blinding and codebreaking 17
2.8 Follow-up procedures 17
2.9 Virology sampled cohort additional sample processing and storage 20
2.10 Economic evaluation 20
2.11 Early discontinuation/withdrawal of participants 21
2.12 Definition of end of trial 21
3. TRIAL INTERVENTIONS 22
3.1 Medication distribution 22
3.2 Medication adherence 22
4. SAFETY REPORTING 22
4.1 Procedures for reporting Adverse Events (AEs) and SAEs 23
4.1.1. AE reporting 23
4.1.2 AE Severity assessment (for assessing clinician) 23
4.1.3 SAEs 24
4.1.4 Other events exempt from immediate reporting as SAEs 24
4.1.5 Procedure for immediate reporting of SAEs 24
4.1.6 Assessment of causality 24
4.1.7 Expectedness 25
4.2 SUSAR reporting 25

For peer review only

For peer review only - http://bmjopen.bmj.com/site/about/guidelines.xhtml
© Copyright: The University of Oxford and Oxford University Hospitals NHS Foundation Trust 2019

4.3 Development Safety Update Reports ... 25
5. STATISTICS ... 26
5.1 Master Statistical Analysis Plan (M-SAP) ... 26
5.2 Open platform trial ... 26
5.2.1 Primary efficacy endpoints and analyses ... 26
5.2.2 Primary efficacy hypothesis & analysis ... 26
5.2.3 Adaptive design ... 26
5.2.4 Interim analyses ... 27
5.2.5 Allocation & adaptive randomisation ... 27
5.2.6 Sample size justification ... 27
5.2.7 Virtual trial simulations ... 29
5.2.8 Procedure for accounting for missing, unused, and spurious data ... 29
5.3 Primary analysis population ... 29
5.4 Procedures for reporting unplanned deviation(s) from the M-SAP ... 29
6. DATA MANAGEMENT ... 29
6.1 Source data ... 29
6.2 Access to data ... 30
6.3 Data recording and record keeping ... 30
7. QUALITY ASSURANCE PROCEDURES ... 30
7.1 Risk assessment and monitoring ... 31
7.2 Trial committees ... 31
8. PROTOCOL DEVIATIONS ... 32
9. SERIOUS BREACHES ... 32
10. ETHICAL AND REGULATORY CONSIDERATIONS ... 32
10.1 Declaration of Helsinki ... 32
10.2 Guidelines for Good Clinical Practice ... 32
10.3 Approvals ... 32
10.4 Other ethical considerations ... 33
10.5 Reporting ... 33
10.6 Transparency in research ... 33
10.7 Participant confidentiality ... 33
10.8 Expenses and benefits ... 33
11. FINANCE AND INSURANCE ... 33
11.1 Funding ... 33
11.2 Insurance ... 33
11.3 Contractual arrangements ... 34
12. PUBLICATION POLICY ... 34

For peer review only

For peer review only - http://bmjopen.bmj.com/site/about/guidelines.xhtml
© Copyright: The University of Oxford and Oxford University Hospitals NHS Foundation Trust 2019

13. DEVELOPMENT OF A NEW PRODUCT/ PROCESS OR THE GENERATION OF INTELLECTUAL PROPERTY 34
14. ARCHIVING 34
15. APPENDIX A: SCHEDULE OF PROCEDURES 36
16. APPENDIX B: Participant Flow Diagram 39
17. APPENDIX C: AMENDMENT HISTORY 40
18. APPENDIX D: SUPPLEMENTARY MATERIAL 44
A. Abbreviations 44
B. Key trial contacts 45
C. Objectives and outcome measures 49
D. Adverse Events 52
19. APPENDIX E: INTERVENTION SPECIFIC APPENDICES 54
1. USUAL CARE ARM 54
1. Background and rationale 54
2. Detail of intervention 54
2. USUAL CARE PLUS MOLNUPIRAVIR 55
1. Background 55
2. Detail of intervention 56
3. Trial visits 58
4. Outcome measures 58
5. Eligibility criteria (in addition to master protocol) 58
6. Professional role of those checking eligibility 59
7. Antiviral agent: Molnupiravir 59
8. Safety reporting 61
3 USUAL CARE PLUS PAXLOVID 66
1. Background 66
2. Detail of intervention 67
3. Trial visits 69
4. Outcome measures 69
5. Eligibility criteria (in addition to master protocol) 69
6. Professional role of those checking eligibility 70
7. Antiviral agent: Paxlovid 70
8. Safety reporting 73
APPENDIX F: STANDARD SCRIPT FOR SAFETY MONITORING OF DRUGS THAT REQUIRE ADJUSTMENT WHEN CO-ADMINISTERED WITH PAXLOVID 81
APPENDIX G: PAXLOVID DRUG-DRUG INTERACTIONS 82
List A: Alphabetical summary of drugs that may interact with Paxlovid 82

For peer review only - http://bmjopen.bmj.com/site/about/guidelines.xhtml
© Copyright: The University of Oxford and Oxford University Hospitals NHS Foundation Trust 2019

List B: Details of Paxlovid drug interactions and implications for eligibility for drugs that are not recommended or require adjustment with Paxlovid in the PANORAMIC trial 85

20. REFERENCES 94

For peer review only

For peer review only - http://bmjopen.bmj.com/site/about/guidelines.xhtml
© Copyright: The University of Oxford and Oxford University Hospitals NHS Foundation Trust 2019

NUFFIELD DEPARTMENT OF PRIMARY CARE HEALTH SCIENCES
Primary Care Clinical Trials Unit
PANORAMIC
Platform Adaptive trial of NOvel antiviRals for eArly treatMent of COVID-19 In the Community

1. BACKGROUND and RATIONALE

Despite high uptake of vaccination against COVID-19, the disease remains prevalent in the UK and in many countries around the world, with many patients continuing to require hospital admission. COVID-19 causes considerable suffering, including loss of ability to perform activities of daily living, loss of educational and work opportunities, and inability to perform caring duties, with far reaching personal and societal consequences. Many go on to experience persisting and/or relapsing symptoms. People with underlying health conditions, unvaccinated people, and those in whom the vaccine is not effective are at increased risk of more severe disease.(1) New 'vaccine escaping' variants may yet emerge, and the impact of early antiviral treatment on long COVID syndromes is as yet unknown. Early treatment with antiviral agents may prevent progression to the later phase of COVID-19. Therefore, there is an urgent need to identify treatments for COVID-19 for use in the community early on in the illness that prevent the need for hospital admission and improves time to recovery.(2, 3)

Antiviral agents may reduce viral shedding, and use of antiviral agents may lead to the emergence of resistance to novel antiviral agents, but the impact of novel antiviral agents on shedding and resistance is not yet known.(4)

1.1 Aims and objectives

Main trial: The primary aim is to determine the effectiveness of selected antiviral agents in preventing hospitalisation and/or death in higher-risk patients with a confirmed positive SARS-CoV-2 PCR or lateral flow test result (see Inclusion/Exclusion Criteria, below).

Virology sampled cohort: A subset of patients from the intervention and comparator arms of the trial will be invited to participate in a virology sampled cohort for virology which aims to determine if there are differences in viral load decay in patients who are/are not treated with antivirals and to identify any common genetic mutations (occurring in greater than 1% of patients) in patient receiving novel antiviral(s).

2. TRIAL DESIGN AND PROCEDURES

PANORAMIC is an open label, prospective, individually randomised, platform, adaptive, controlled clinical trial in community care. Trial arms will include:

Intervention arms: Novel antiviral agents (or combinations) targeting SARS-CoV-2, specified by the Antivirals Taskforce (AT) and with capacity for sequential introduction of each treatment regimen into the trial plus Usual Care.

Comparator arm: Usual Care, defined as the currently recommended treatment delivered by responsible clinicians. Usual Care will not be mandated by the trial, as recommended treatments may change and be tailored to individual characteristics, and self-care will vary. Use of over-the-counter medication as well as key medications such as inhaled steroids and monoclonal antibodies will be captured and changing outcomes and treatment modalities over time in the Usual Care arm will be accounted for in the analysis: see Usual Care ISA.

For peer review only

For peer review only - http://bmjopen.bmj.com/site/about/guidelines.xhtml
© Copyright: The University of Oxford and Oxford University Hospitals NHS Foundation Trust 2019

2.1 Participant identification

2.1.1 Trial participants

The trial includes participants who test positive for SARS-CoV-2 infection and with ongoing symptoms consistent with COVID-19, not hospitalised, and who are aged 50 years and over, or 18-49 years and considered clinically vulnerable (see Inclusion Criteria below).

2.1.2 Inclusion criteria

- Participant is able and willing to provide informed consent, or their legal representative is willing to provide informed consent
- Symptoms attributable to COVID-19 started within the past 5 days and ongoing
- A positive PCR or lateral flow SARS-CoV-2 test*
- Aged ≥50 years OR aged 18-49 years with one of the following known underlying chronic health conditions considered to make them clinically vulnerable:
    - chronic respiratory disease (including chronic obstructive pulmonary disease (COPD), cystic fibrosis and asthma requiring at least daily use of preventative and/or reliever medication)
    - chronic heart or vascular disease
    - chronic kidney disease
    - chronic liver disease
    - chronic neurological disease (including dementia, stroke, epilepsy)
    - severe and profound learning disability
    - Down's syndrome
    - Diabetes mellitus (Type or Type II)
    - immunosuppression: primary (e.g., inherited immune disorders resulting from genetic mutations, usually present at birth and diagnosed in childhood) or secondary due to disease or treatment (e.g., sickle cell, HIV, cancer, chemotherapy)
    - solid organ, bone marrow and stem cell transplant recipients
    - morbid obesity (BMI >35)
    - severe mental illness
    - care home resident
    - judged by recruiting medically qualified professional, research nurse, nurse prescriber, prescribing pharmacist, dependent on the ISA for the specific IMP involved, to be clinically vulnerable

* Any positive PCR or lateral flow test taken up to two days before symptom onset and randomisation qualifies.

2.1.3 Exclusion criteria

- Patient currently admitted to hospital (inpatient)
- Previous randomisation in the PANORAMIC trial
- Currently participating in a clinical trial of a therapeutic agent for acute COVID-19
- Additional exclusions specific to each intervention arm, if any, as listed in the ISA's of currently open trial arms

2.1.3.1 Additional exclusion criteria for virology sampled cohort only:

- Receipt of a non-trial anti-SARS-CoV-2 antibody therapy within the previous 3 months

For peer review only - http://bmjopen.bmj.com/site/about/guidelines.xhtml
© Copyright: The University of Oxford and Oxford University Hospitals NHS Foundation Trust 2019

2.2 Trial procedures

2.2.1 Informing potential participants about the trial

I. All Health, health related, and Social Care professionals (including NHS 111 and Test and Trace clinicians, care home staff, pharmacy staff, etc) will be able to provide information about participation and direct potential participants to the online trial information and the trial website

II. The ZOE COVID-19 Application, Health Wise Wales, Join Dementia Research (JDR) and other COVID-19 research studies e.g., REACT, VIRUS WATCH) will sign-post to the trial.

III. National media campaigns will use television, radio, and social media platforms to generate awareness of the trial and to signpost to the trial

IV. Targeted campaigns for vulnerable groups will be by media campaigns, via national charities, social media groups and relevant secondary care clinicians.

V. All NHS facilities including testing centres including NHS walk in/ drive through centres will be able to inform potentially eligible participants about the trial and refer them to the trial website and/or trial team

VI. Clinicians can reach out to potentially eligible participants identified by receiving SARS-CoV-2 test results from Test and Trace and laboratories, and by regular searches for patients with a positive SARS-CoV-2 test result in their clinical database. Contact can be made with potential participants verbally or by text, email, and telephone

VII. NHS Digital (and analogous services in devolved administrations) will provide a daily list of contact details from Pillar 2 testing data of people with a positive SARS-CoV-2 test. The trial team and the Hubs will be able to contact these people within 24-48hrs of test result to discuss participation. Patient details will be provided in accordance with section 251 under the General Notice under the Health Service Control of Patient Information Regulations 2002 (COPI). The COPI notice provides a temporary legal basis to allow access to participant data and protects participants whilst avoid confidentiality breaches for COVID-19 purposes. COPI is only applicable to Hubs in England and Wales. Following the expiration of the current COPI notice, PANORAMIC will gain access to and process participant identifiable information, in England and Wales only, without consent under Regulation 5 of the Health Service (Control of Patient Information) Regulations 2002 as amended by Section 117 of the Care Act 2014.

VIII. EMIS Anywhere, a data extraction service for primary care data, and analogous general practice clinical record facilities, will be able to reach out to potentially eligible participants and signpost them to the PANORAMIC website to explore their participation

For peer review only

2.2.2 Recruitment

Face-to-face as well as remote (trial website or telephone call) screening, eligibility and consent procedures will be used. All participants (apart from those who lack capacity to do this) will have a two-way discussion, either face-to-face or by a telephone/video call from a medically qualified professional , research nurse, nurse prescriber or prescribing pharmacist, dependent on the ISA for the specific IMP involved, prior to randomisation.

For participants who are too unwell or unable to respond to surveys for themselves, a Trial Partner they identify will be able to assist their participant in completing screening, baseline, consent and follow up online forms and/or calls and provide information to them on their behalf where necessary. A letter will be issued to Trial Partners, informing them of the trial, notifying them that they have been nominated for this role by the participant.

For peer review only - http://bmjopen.bmj.com/site/about/guidelines.xhtml
© Copyright: The University of Oxford and Oxford University Hospitals NHS Foundation Trust 2019

2.2.2.1 Recruitment at PANORAMIC Hubs

PANORAMIC Hubs will include GP sites (either single practices or a federation of practices that are able to operate under a single site agreement and PI to undertake trial procedures as detailed in the protocol), community trusts, and other health service providers, including government agencies e.g., UK Health Security Agency. Potential participants can be referred to Hubs by other health care facilities for possible inclusion. As well as recruiting patients through routine consultations, Hubs will search their databases and test results they receive for patients defined as clinically vulnerable (see inclusion criteria for definition) with a positive test for COVID-19, and telephone or text them to invite them to take part in the trial. Either face-to-face or by telephone, a medically qualified professional , research nurse, nurse presciber or prescribing pharmacist, as specified in the ISA for the specific IMP involved, at the Hub will explain the trial to the potentially eligible participant; collect screening, baseline, and contact information; take informed consent; and confirm eligibility (see details below for each trial procedure). If the participant is eligible, they will automatically be randomised to one of the trial arms and provided with a participant pack (see section 3.1 Medication Distribution).

A PI at each Hub will provide trial oversight, for participants recruited via the Hub and inform the central trial team of any Serious Adverse Events (SAE).

2.2.2.2 Central recruitment

Potential participants can present directly to the central trial team via the trial website or free-phone telephone number, in additional to via a PANORAMIC Hub. Screening, baseline, contact information and informed consent can be self-completed by the potential participant, or completed during a telephone call with a member of the central trial team. A medically qualified professional or appropriately trained research nurse will then confirm eligibility. If eligible, the participant will be randomised and provided with a participant pack (see section 3.1 Medication Distribution). All trial procedures are described below in detail.

2.2.2.3 Virology sampled cohort recruitment

The virology sampled cohort will consist of enhanced monitoring of a subset of participants who additionally volunteer for this aspect of the trial in each arm of the trial. Recruitment will be from PANORAMIC Hubs that are assigned virology sampled recruiting sites, or through the central trial team.

2.3 Screening

Screening can be completed face-to-face as well as remotely via the trial website, or a free-phone telephone service that enables participants to have a two-way discussion with the central trial team or Hub staff who are trained in trial procedures.

Participants of child-bearing potential are required to confirm a negative pregnancy test prior to starting any antiviral agent in the trial that may be teratogenic, and as specified in its ISA. Thus, they should indicate willingness to take such a pregnancy test at screening. For those recruited at face-to-face visits at PANORAMIC Hubs, undertaking a pregnancy test will be part of the initial screening visit. For participants recruited remotely, the pregnancy test will be supplied in the participant pack with the antiviral agent. The pregnancy test must be completed prior to starting an antiviral agent that requires confirmation of a negative pregnancy test before staring the agent. This will be clearly

For peer review only - http://bmjopen.bmj.com/site/about/guidelines.xhtml
© Copyright: The University of Oxford and Oxford University Hospitals NHS Foundation Trust 2019

explained prior to randomisation (see section 2.8 Follow-up Procedures for details regarding confirmation of a negative test result).
Those who are ineligible because they are asymptomatic will be alerted to possible trial participation should they develop symptoms.

2.4 Informed consent

There are separate procedures for recruiting eligible participants with capacity to give informed consent and residents of care homes who lack capacity to consent. All consent forms will be completed online and paperless.

Eligible participants capable of giving informed consent will be asked to provide informed consent after a two-way discussion between a medically qualified professional , research nurse, nurse prescriber or prescribing pharmacist, as specified in the ISA for the specific IMP involved, and the participant, either face-to-face or by telephone, prior to randomisation, where the risks and benefits of taking part and follow-up procedures will be explained.

In addition to taking consent face-to-face, consent may also be taken remotely, using online paperless consent forms and via telephone/video discussion, because of the pandemic circumstances and the need to maximise the pragmatic nature of the trial. Participants will be able to download their consent form after completion, and it can be printed by the central trial team and delivered to participants. Electronic consent forms will be held securely on the trial database. For those recruited in Hubs, a copy will be filed in patients' medical notes and a copy will be printed and given to patients.

Prior to consent, written and summary versions of the Patient Information Sheet (PIS), and Informed Consent Form (ICF) will be available to participants detailing no less than: the exact nature of the trial; and the known side-effects and risks involved in taking part. It will be clear that the participant is free to withdraw from the trial at any time. A pictorial and/or video and a summary PIS will be available which can be more easily read by those feeling very unwell, or those with low reading comprehension skills. Adequate time will be given to the participant to consider the information and to ask any questions about the trial before deciding whether to participate. After consent, participants will enter online baseline information, including their address, contact details and those of a Trial Partner. Identifying a Trial Partner is not a requirement of trial participation.

People who lack capacity to consent for themselves will only be recruited from care homes: adults who lack capacity to consent living elsewhere will not be recruited. If the recruiting health and social care professional deems that a patient in a care home lacks capacity to provide consent for themselves, then a personal or professional legal representative (England and Wales only) will be asked to provide consent. A personal legal representative is defined as a person not connected with the conduct of the trial who is suitable to act as the legal representative by virtue of their relationship with the adult. A professional legal representative may be a doctor responsible for the medical treatment of the adult if they are independent of the trial, or a person nominated by the healthcare provider. In all instances, a personal legal representative will be sought first, and a professional legal representative sought only if a personal legal representative cannot be identified. Legal representative and recruiting clinicians will not endeavour to obtain consent for or recruit people into the trial people who, in addition to their lack of capacity, have a quality of life which can reasonably be considered as not acceptable to the potential participant to avoid potentially life lengthening

For peer review only - http://bmjopen.bmj.com/site/about/guidelines.xhtml
© Copyright: The University of Oxford and Oxford University Hospitals NHS Foundation Trust 2019

intervention in those who would not wish to have such an intervention. Legal representative consent (relative/family member/independent treating physician) can be taken face to face or remotely.

The legal representative will be provided with information about the trial and made aware of the following: they are being asked to give consent on behalf of the incapacitated adult, they are free to decide whether they wish to make this decision or not, and they are being asked to consider what the adult would want, and to set aside their own personal views when making this decision.

2.5 Eligibility assessment

For participants who have provided consent, eligibility will be assessed by a medically qualified professional, research nurse, nurse prescriber or prescribing pharmacist, as specified in the ISA for the specific IMP involved, at a PANORAMIC Hub, other health service providers including government agencies e.g., UK Health Security Agency or by the central clinical team. For some antiviral agents, eligibility may only be assessed by a medically qualified professional, and the professional roles of each Health Care Professional (HCP) qualifying them to do this will be specified in the ISA for each agent.

PANORAMIC Hubs can contact the central clinical team for guidance regarding eligibility queries. Depending on the exclusion criteria outlined in ISAs, eligibility can be assessed by eliciting medical history and relevant information, including a drug history, directly from the participant, and the participant can be randomised if they are deemed eligible and there is no contraindication to the trial drugs currently in the trial. Where specified in the ISA, eligibility checking will be assessed additionally through direct access to the participant's Summary Care Record in England or a medical record summary in use for clinical care in any UK Devolved Administration, and by reference to relevant medical information obtained from the participant's primary care or secondary care records (where the person confirming eligibility deems this necessary)..

Potential participants will be informed that those at the highest risk of complications from COVID-19 are able to get antiviral treatment outside of the trial from the NHS.

If an additional IMP is introduced into the trial, which requires extensive clinical interpretation of the eligibility criteria, the eligibility assessment process will be reviewed and amended accordingly and outlined fully in the ISA with screening and eligibility CRFs and associated processes updated accordingly.

For peer review only - http://bmjopen.bmj.com/site/about/guidelines.xhtml
© Copyright: The University of Oxford and Oxford University Hospitals NHS Foundation Trust 2019

NUFFIELD DEPARTMENT OF PRIMARY CARE HEALTH SCIENCES
Primary Care Clinical Trials Unit
PANORAMIC
Platform Adaptive trial of NOvel antiviRals for eArly treatMent of COVID-19 In the Community

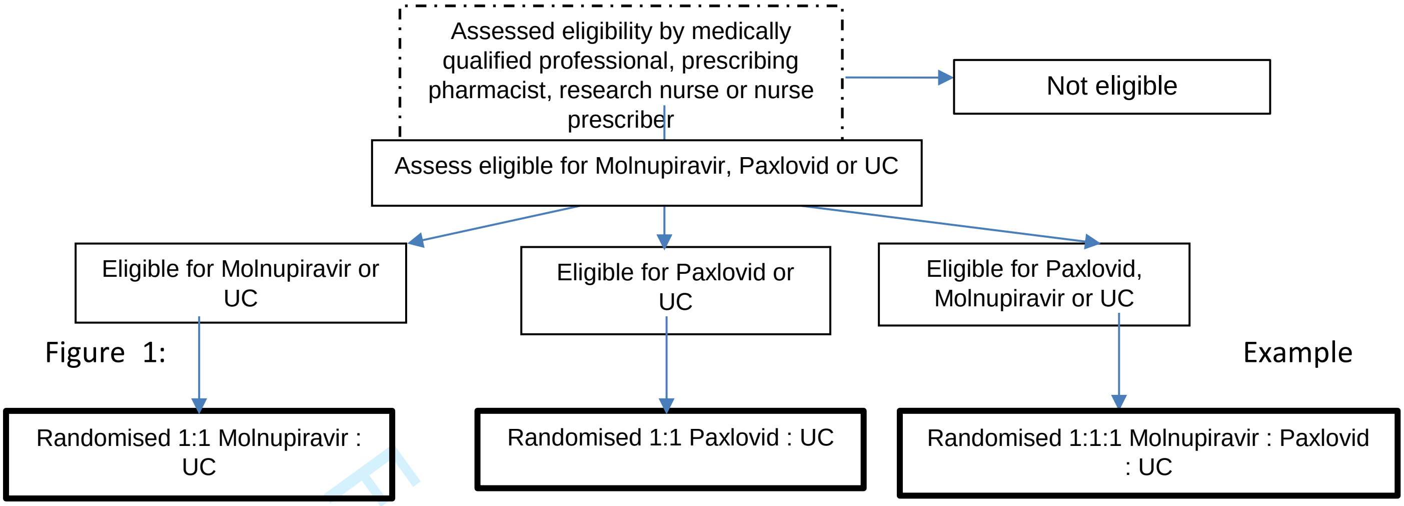

Figure 1: Example of eligibility flow for randomisation when adding Paxlovid as a new intervention.

2.6 Randomisation

Participants will be randomised using a secure, fully validated, and compliant web-based randomisation system. Once deemed eligible, a medically qualified professional , research nurse, nurse prescriber or prescribing pharmacist, as specified in the ISA for the specific IMP involved, from the central clinical team or Hub (as documented on the delegation log) will randomise the participant. Participants will be randomised to one trial arm using equal allocation ratios corresponding to the number of eligible arms for which the participant is eligible for in the trial. For instance, if there are two active interventions (A & B), the allocation ratio will be 1:1:1 for Usual Care, active A, active B (respectively), such that 33% of participants are randomised to Usual Care. If there are 3 active interventions, the allocation ratio will be 1:1:1:1, such that 25% of participants are randomised to Usual Care. Patients must be eligible for at least two arms (Usual Care and at least one novel antiviral intervention). Stratification will be by age and vaccination status.

The randomisation database will automatically alert the relevant IMP distributor and the participant, trial team and legal representative if applicable will be notified electronically of the treatment allocation. If the participant does not have an email address, they will be notified by telephone.

2.7 Blinding and codebreaking

PANORAMIC is an open-label trial. The participant, legal representative if applicable, and the recruiting clinician will know the participant's allocation. Therefore, no unblinding or code breaking is required. However, those managing the data will be blind to participant allocation.

The trial team and recruiting clinicians will be blinded to emerging results of interim analyses. During the course of the trial, only the unblinding statisticians and the independent members of the Data and Safety Monitoring Committee (DSMC) will have access to the unblinded interim results.

2.8 Follow-up procedures

Following randomisation, participants in the intervention arm will be sent a participant pack (see section 3.1 Medication Distribution). The participant pack will contain: the antiviral agent, an information booklet; participant card detailing how the medication should be administered, precautions and safety guidance; medication appendix providing further information about the treatment (available prior to randomisation as part of the PIS); wallet emergency card; pregnancy test

For peer review only

For peer review only - http://bmjopen.bmj.com/site/about/guidelines.xhtml
© Copyright: The University of Oxford and Oxford University Hospitals NHS Foundation Trust 2019

(only for participants of child-bearing potential). Those randomised to Usual Care, will receive an information booklet via email or post.

The participant pack for participants randomised to the intervention arm who have consented to take part in the virology sampled cohort will be supplemented with; an additional virology sampling kit containing approved instructions, and materials to post samples to the virology processing sites which will be posted separately to participants. Those randomised to Usual Care, will receive these additional materials in addition to an information booklet via email or post.

Patients might be asked to attend a face-to-face visit or to donate a microbiological or blood sample, depending on the requirements for the evaluation of each specific antiviral agent. This will depend on the antiviral agents' licensing status, available safety data and their approval status. Thus, for antiviral agents with an established safety profile, follow-up will be via self-completed questionnaires online or through telephone calls, and primary care and/or hospital record searches. For other antiviral agents, the trial will have capability for face-to-face assessment, sampling, and safety checks initially, after which a drug may progress to 'remote evaluation', which will only be implemented following approval of a substantial amendment.

A safety call will be made on Day 1 (day after randomisation) with participants of child-bearing potential who have been allocated to an antiviral agent with teratogenic potential (as specified in the relevant ISA) by a member of the central trial team or the recruiting PANORAMIC Hub, to confirm receipt of the participant pack (containing a urine pregnancy test). During this Day 1 call, a member of the trial team will confirm with participants of childbearing potential, that a pregnancy test has been done and that the result is negative before starting an antiviral agent with teratogenic potential. In the event of a positive test result, the participant will be asked not to take any of the antiviral agent, return it, and will be withdrawn from the trial. Results will be documented in the Day 1 Call CRF. The pregnancy test must be completed prior to taking the antiviral agent in question and this will be clearly explained prior to randomisation. Participants of child-bearing potential will also be asked to confirm a negative pregnancy test result in their day 1-3 of daily diaries.

All participants, irrespective of group allocation, will be contacted on Day 2 (2 days after randomisation) to confirm receipt of trial materials, confirm follow-up procedures and answer queries. This call will be made by a member of the central trial team or the PANORAMIC Hub. At this day 2 call, participants allocated to any antiviral agent arm of the trial, will be also asked if they have received their trial pack and if they are experiencing any potential side-effects from the IMP. This call will be made by clinicians, research nurses, nurse prescribers or prescribing pharmacists, dependent on the ISA for the specific IMP involved, from the central trial team (for those recruited centrally or from a Hub) or PANORAMIC Hub (for those recruited via their Hubs). For higher risk IMPs, additional safety calls may be made as detailed in the relevant ISA.

If the participant or their Trial Partner cannot be reached at this stage, the trial team will contact the patient's GP to request information on any healthcare contacts that the participant may have had since they were enrolled into the trial, to capture any potential safety events.

Participants on all arms of the trial will be asked to complete a daily diary each day for 28 days and be contacted at 3 and 6 months from randomisation, where they will rate the severity of symptoms, record contacts with the health services (including hospital admissions, hospital outpatient visits, accident and emergency attendances, use of specialist services and primary care encounters), impact

For peer review only - http://bmjopen.bmj.com/site/about/guidelines.xhtml
© Copyright: The University of Oxford and Oxford University Hospitals NHS Foundation Trust 2019

of symptoms on work/trial, record medication use and new infections in the household. We will collect the *EuroQoL EQ-5D-5L* (baseline, days 14 and 28, and 3 and 6 months). The central trial team will call participants/trial partners with no internet access or those who have not completed their diary for at least two consecutive days before days 7, 14 and 28. No more than six contact attempts will be made at each of these follow-up points. All participants will be telephoned within one day, and 24-hour access to the safety phone line and emergency procedures will be emphasised to those randomised to an antiviral agent. Participants will be contacted at three and six months to ascertain wellbeing and longer-term consequences of their illness, including proportion meeting criteria for 'long Covid'. Vaccination status, including number of vaccinations received will be recorded.

Adherence to trial medication will be assessed by self-report.

Participants' medical records will be accessed up to twelve months following enrolment to ascertain follow up data from enrolment to 6 months. Data will be collected as close to real time as possible; RCGP RSC, EMIS, NHS Digital, electronic Data Research and Innovation Service (eDRIS), The Secure Anonymised information Linkage (SAILS) Databank, Health and Social Care Northern Ireland (HSC Business Services Organisations/HSC Trusts) (HSC NI) and other sources of routinely collected data will be utilised if required. To investigate the impact of trial interventions on the longer-term effects of COVID-19, we will use these data collection methods to follow-up participants, for up to 10 years.

Virology samples cohort: 300 participants from each trial intervention arm and the Usual Care comparator arm will be recruited to enrol into the voluntary virology sampled cohort. Participants will fall into two categories; the first 30 patients volunteering to enrol from each trial arm will undergo intensive daily viral load monitoring, whereas the remaining 270 from each arm in the virology samples cohort will have less intensive viral load monitoring.

The first 30 participants in each arm will be asked to provide daily nasopharyngeal swabs for 7 days, and an additional nasopharyngeal swab on Day 14 (+/- 1 day). For patients in the intervention arms, the first sample will be taken immediately prior to the participant commencing anti-viral treatment (Day 1). For participants allocated to Usual Care Day 1 will be the day following randomisation.

The next 270 participants volunteering for this aspect of the trial in each arm will be asked to provide 3 nasopharyngeal swabs: once prior to starting treatment, once on Day 5 (+/- 1 day) and once on Day 14 (+/- 1 day).

All participants volunteering for this aspect of the trial will be asked to take 3 finger prick dried blood spot samples: once pre-treatment, once on Day 5 (+/- 1 day) and once on Day 14 (+/- 1 day).

Participants consenting to take part in the virology sampled cohort will be sent CE-IVD approved sampling kits for nasopharyngeal sampling, dried blood spot sampling, pre-paid postage, and packaging, to post samples to the virology processing site. The kits will include approved instructions and will be delivered to the participant by courier from a central stock or the PANORAMIC Hubs. Sampling may occur at home or at Hubs, with participants supported by the Hubs or the central trial team. Samples taken at home should be posted to the trial team within 3 days of sampling, and ideally within 24 hours.

For peer review only - http://bmjopen.bmj.com/site/about/guidelines.xhtml
© Copyright: The University of Oxford and Oxford University Hospitals NHS Foundation Trust 2019

Hubs and the central trial team will receive training in all virology sampling procedures from the Royal Free/University College London (UCL) team who will provide ongoing support to the Hubs and central trial team.

A telephone call and/or SMS text message/email reminder will be sent to participants who have enrolled into the intensive monitoring cohort (the first 30 in each trial arm) on Day 4 (+/- 1 day), Day 7 (+/-1 day) and Day 14 (+/- 1 day).

2.9 Virology sampled cohort additional sample processing and storage

Viral load in the upper respiratory tract rises to a peak at symptom onset, becoming undetectable in 1 or 2 weeks in most patients. The primary aim of this intensively sampled cohort is to assess the impact the antiviral agents have on viral load, with a focus on prediction of time to virus clearance. Important confounders of this are presence of antibodies and so these will be monitored.

The secondary aim is to evaluate the potential for antivirals to cause mutations. For those samples containing a sufficient viral load, whole genome sequencing of the pre- and post-treatment samples will be performed.

Viral load determination and viral genome sequencing will be performed using material extracted from nasopharyngeal swabs.

Since antibody status is likely most crucial to viral dynamics, it will be measured in dried blood spots collected via finger pricks as described above at Day 0, Day 5, and Day 14.

Samples will be labelled with the participant's trial ID number and the date of sample collection. Nasopharyngeal swabs will be sent to Great Ormond Street Hospital (GOSH) for Children who will process the samples for viral load and forward them to UCL for sequencing. Samples will be accessed by GOSH and UCL members of the trial team. Dried blood spots will be sent to Institute of Immunology and Immunotherapy Birmingham for processing to determine antibody status. After analyses samples will be returned to the research team and with participants consent may be stored for 12 months following the end of the trial. If consent is held for long-term storage, these samples may be used for future ethically approved research. However, where no consent is held samples will be destroyed on completion of the analyses in line with the Human Tissue Act 2004.

2.10 Economic evaluation

A prospective economic evaluation will be embedded within the trial design to assess the cost effectiveness of each antiviral from an NHS perspective. We will estimate the resource inputs associated with embedding each trial antiviral treatment into routine clinical practice and estimate societal costs. Broader resource use will be drawn from General Practice Data for Planning and Research (GPDPR) data and linked Hospital Episode Statistics – encompassing primary care encounters, hospital inpatient/day case admissions, outpatient visits, and accident and emergency attendances. Unit costs will be valued using national reference tariffs and attached to resource inputs to generate a compound total health care cost per trial participant over the trial time horizon. EQ-5D-5L data will be converted using standard algorithms into utility scores for quality-adjusted life year (QALY) estimation, and cost-effectiveness expressed as incremental cost per QALY gained (5). Secondary expressions of cost-effectiveness will include incremental cost per hospitalisation and/or death prevented over 28 days.

For peer review only - http://bmjopen.bmj.com/site/about/guidelines.xhtml
© Copyright: The University of Oxford and Oxford University Hospitals NHS Foundation Trust 2019

Bivariate regression of costs and measures of health consequence, with multiple imputation of missing data, will be conducted to generate within-trial estimates of incremental cost-effectiveness. Sensitivity analyses will assess the impact of areas of uncertainty surrounding components of the economic evaluation. Cost-effectiveness acceptability curves will show the probability of cost-effectiveness of each treatment evaluated at alternative cost-effectiveness thresholds. Cost-effectiveness threshold values will be informed by guidance from UK government departments on the value placed by decision-makers on an additional QALY (6) and on a statistical life (7).

A decision-analytic modelling-based economic evaluation will also be conducted. The baseline decision-analytic model will be developed during the early stages of the trial and aim to provide a framework for extrapolating the cost-effectiveness of each antiviral beyond the parameters of PANORAMIC trial. Accepted guidelines for good practice in decision-analytic modelling will be followed. The model will consider the progression of symptomatic COVID-19 status over time, and the model structure will capture disease progression using health states that represent the important natural history and clinical- and event-related activity for symptomatic COVID-19 symptomatic status, the appropriate model type (e.g., Markov or discrete-event simulation approach) and the appropriate analytical framework (e.g., cohort analysis versus individual-level simulation). Parameter inputs into the model will be informed by data extracted from PANORAMIC trial, supplemented by data identified from external sources following targeted literature searches. As with the within-trial economic evaluation, cost-effectiveness will be expressed in terms of incremental cost per QALY gained. Multi-parameter uncertainty in the model will be addressed using probabilistic sensitivity analysis. Cost-effectiveness acceptability curves will be used to show the probability of cost-effectiveness of each anti-viral strategy at alternative cost-effectiveness thresholds held by decision-makers. Long-term costs and health consequences will be discounted using nationally recommended discount rates. Specific plans for the economic evaluation will be outlined in a pre-specified health economics analysis plan.

2.11 Early discontinuation/withdrawal of participants

Each participant or their legal representative on the participant's behalf, has the right to withdraw from the trial at any time. For those that lack capacity, expression of dissent in any form will be taken as an indication they do not wish to be included and they will be withdrawn. In addition, the Investigator may discontinue a participant from the trial at any time if the Investigator considers it necessary for any reason including:

- Ineligibility (either arising during the trial or retrospectively)
- Withdrawal of consent

The reason for withdrawal will be recorded on the CRF. Data that has already been collected about the participant will be kept and used. Samples collected from participants and data arising from the processing of those samples for research purposes may be used in the trial analysis.

2.12 Definition of end of trial

The end of the trial will be the last data capture of last participant.

For peer review only - http://bmjopen.bmj.com/site/about/guidelines.xhtml
© Copyright: The University of Oxford and Oxford University Hospitals NHS Foundation Trust 2019

3. TRIAL INTERVENTIONS

Antiviral agent information can be found in the relevant ISAs.

3.1 Medication distribution

In general, the distribution of antivirals can be implemented by the PANORAMIC Hubs; an accredited licensed central facility; an online, community or hospital pharmacy, and the PC-CTU, if approved by MHRA. Distribution of trial packs to participants will be tracked via courier or call/text message. Clinicians may be able to prescribe trial antivirals that can be issued in the community, and pharmacies can issue antivirals to the patient by community pharmacy services or 'on-line pharmacy' services, or it can be collected from the pharmacy by the participant or someone on their behalf (with appropriate infection control measures).

The arrangements for the distribution of each antiviral agent are detailed in the ISA.

3.2 Medication adherence

Medication adherence will be captured in daily diaries and phone or video calls from the trial team.

Accountability logs will be kept by the distributor (as specified in the ISA) and central monitoring of the logs will allow oversight by the PC-CTU.

A member of the central clinical team or PANORAMIC Hub will telephone all participants to confirm receipt of the antiviral agent, and that the participant has read the instructions on the participant card. Receipt will be documented in the Day 1 or Day 2 telephone calls (see section 2.8 Follow-up procedures). If we are unable to contact participants or their trial partner, we will confirm and log receipt of antiviral agent by checking the patient's daily diary, where they are asked daily whether they have taken their trial treatment and the number of tablets/capsules taken. We can also check via the courier portal, whether the medication has been received by the participant, for additional confirmation.

If a participant decides that they no longer wish to take their medication, we will provide a pre-paid envelope so that they can return the medication to the trial team via courier and the trial team will ensure all drug accountability logs are updated accordingly.

4. SAFETY REPORTING

Symptoms, potential medication side-effects and Serious Adverse Events (SAE) will be collected from participant daily diaries, calls to participants/Trial Partners, face-to-face visits with Hub clinicians, medical records, notes reviews, NHS Digital, eDRIS, SAIL, HSC NI, data extracts and RCGP data downloads.

We will adopt a risk assessed and proportionate approach to safety monitoring. In line with the SmPC or Investigator Brochure, we will assess the risks and the safety profile for each antiviral agent, and detail the mitigation and monitoring procedures in the ISA. All safety procedures will be according to PC-CTU pharmacovigilance SOP.

For peer review only

For peer review only - http://bmjopen.bmj.com/site/about/guidelines.xhtml
© Copyright: The University of Oxford and Oxford University Hospitals NHS Foundation Trust 2019

4.1 Procedures for reporting Adverse Events (AEs) and SAEs

The participant will be asked to rate the severity of a number key COVID-19 symptoms which are also possible common medication side effects in their daily diary. The severity of individual events and symptoms will be assessed over time by participants on the following scale: no problem/mild problem/moderate problem/major problem.

	Participant reported symptom rating
**No problem**	Individual symptom not currently experienced
**Mild problem**	Symptom is short-lived or mild; medication may be required. No limitation to usual activity
**Moderate problem**	Symptom causes moderate limitation in usual activity. Medication may be required.
**Major problem**	Symptom causes considerable limitation in activity. Medication or medical attention required.

Symptoms of COVID-19 and medication AE symptoms may overlap and can be difficult to disentangle. Trends in the prevalence in the severity of symptoms between Usual Care and antiviral agent arms will be compared, for evidence of increased severity of measured symptoms in those randomised to receive trial antiviral agents.

4.1.1. AE reporting

For each antiviral agent, we will only collect AEs (other than those pre-specified symptoms collected via the participant diaries) if and when specified in the relevant ISA. If there is a requirement to collect AEs or specific AEs for an antiviral agent these will be monitored from the start of treatment for the 28-day trial duration, unless otherwise specified in the ISA, and assessed by a clinician (independent from the Sponsor) for causality and severity (definitions below).

Participants will be free to withdraw from taking the antiviral if they perceive they have an intolerable AE. Participants will also be provided with a Participant Card detailing potential side-effects and a Wallet Emergency Card with 24-hour contact telephone line, answered by a clinical team, enabling them to report AEs they experience whilst taking the drug. This card will also alert hospital clinicians about trial participation, should a participant be admitted to hospital. In the event of a medical emergency, trial participants will be instructed to show this card to the clinician they see. Based on clinical judgement, the clinician may contact the participant directly within 24 hrs of becoming aware of an AE reported in their daily diary or on the Freephone number, to advise the participant on the appropriate clinical care.

4.1.2 AE Severity assessment (for assessing clinician)

	Clinical assessment of severity
**GRADE 1 (Mild)**	Short-lived or mild symptoms; medication may be required. No limitation to usual activity
**GRADE 2 (Moderate)**	Moderate limitation in usual activity. Medication may be required.
**GRADE 3 (Severe)**	Considerable limitation in activity. Medication or medical attention required.

For peer review only - http://bmjopen.bmj.com/site/about/guidelines.xhtml
© Copyright: The University of Oxford and Oxford University Hospitals NHS Foundation Trust 2019

4.1.3 SAEs

All-cause hospitalisation and/or death is the primary outcome, and this data will be captured in CRFs. SAEs other than hospitalisation or death due to COVID-19 must be reported for all antiviral agents.

SAEs must be reported to PC-CTU by the person who has discovered the SAE or nominated delegate within 24 hours of becoming aware of the event. The sponsor or delegate will ensure it is reviewed by the CI or other delegated personnel for relatedness and expectedness as soon as possible taking into account the reporting time for a potential SUSAR according to the relevant competent authority. If the event has not resolved, at the 28-day time point the SAE will be reviewed again by the central clinical team, to see if resolution has occurred. If the event is considered 'resolved' no further follow up is required. If not, the event must be followed up until such a time point.

All SAEs that have not resolved by the end of the trial or those that are identified retrospectively, or that have not resolved upon discontinuation of the participant's participation in the trial, must be followed until any of the following occurs:

- The event resolves
- The event stabilises
- The event returns to "baseline", if a "baseline" value/status is available
- The event can be attributed to agents other than the trial intervention or to factors unrelated to trial conduct
- It becomes unlikely that any additional information can be obtained (participant or health care practitioner refusal to provide additional information, lost to follow-up after demonstration of due diligence with follow-up efforts)

See Appendix D. Supplementary Material for definitions of AEs

4.1.4 Other events exempt from immediate reporting as SAEs

Hospitalisations will be defined as at least a one-night admission to hospital, or at least one night in a 'Hospital at Home' program after hospital assessment. Hospitalisation for a pre-existing condition, including elective procedures planned prior to trial entry, which has not worsened, does not contribute to our primary outcome, and does not constitute an SAE.

4.1.5 Procedure for immediate reporting of SAEs

- Trial team/responsible clinician/GP Practice/CI will complete an SAE report form, directly into the database, for all reportable SAEs
- GP practice/trial team/RCGP will provide additional, missing or follow up information in a timely fashion
- If necessary, the participant/trial partner may be contacted to provide additional, missing or follow up information as required

An investigator, who is independent to the Sponsor but part of the trial team, will review the SAE once reported, collect as much information and report to the Sponsor delegate within the timeframe according to the PC-CTU SOPs.

4.1.6 Assessment of causality

For peer review only - http://bmjopen.bmj.com/site/about/guidelines.xhtml
© Copyright: The University of Oxford and Oxford University Hospitals NHS Foundation Trust 2019

The relationship of each SAE to the antiviral agent must be determined by a medically qualified individual according to the following definitions:

- **Unrelated** – where an event is not considered to be related to the antiviral agent
- **Possibly** – although a relationship to the antiviral agent cannot be completely ruled out, the nature of the event, the underlying disease, concomitant medication, or temporal relationship make other explanations possible
- **Probably** – the temporal relationship and absence of a more likely explanation suggest the event could be related to the antiviral agent
- **Definitely** – the known effects of the antiviral agent, its therapeutic class or based on challenge testing suggest that the antiviral agent is the most likely cause

Reported SAEs will be assessed for relatedness by an individual who is independent of the Sponsor (i.e., either the Hub PI or an independent reviewer). An independent reviewer is an investigator independent of the Sponsor, but part of the trial team.

AEs/SAEs judged possibly, probably, or definitely related will be considered as related to the antiviral agent.

4.1.7 Expectedness

Expectedness of SAEs will be assessed and determined by delegated members of the central trial team or by an independent reviewer. Expectedness will be assessed in accordance with the relevant Reference Safety Information (RSI) section of the Summary of Product Characteristics (SmPC) Investigator's Brochure (IB). The RSI will be the current Sponsor and MHRA approved version at the time of the event occurrence.

4.2 SUSAR reporting

All SUSARs will be reported by the sponsor delegate to the relevant Competent Authority and to the REC and other parties as applicable. For fatal and life-threatening SUSARs, this will be done no later than seven calendar days after the Sponsor or delegate is first aware of the reaction. Any additional relevant information will be reported within eight calendar days of the initial report. All other SUSARs will be reported within 15 calendar days.

PIs will be informed of all SUSARs for the relevant antiviral agent for all studies with the same Sponsor, whether or not the event occurred in the current trial.

4.3 Development Safety Update Reports

The DSUR will be developed and submitted annually on the anniversary date that the trial receives Clinical Trial Authorisation +60 days. Due to the nature of this trial and the importance of sharing the science of COVID-19 and the drug, internationally, we expect to produce reports to the UK Government and regulatory agency more frequently upon request.

For peer review only - http://bmjopen.bmj.com/site/about/guidelines.xhtml
© Copyright: The University of Oxford and Oxford University Hospitals NHS Foundation Trust 2019

5. STATISTICS

5.1 Master Statistical Analysis Plan (M-SAP)

Details of the statistical design and methods for both the main trial and the virology substudy will be described in a Master Statistical Analysis Plan (M-SAP).

PANORAMIC will begin as a two arm, 1:1 randomised trial but will have the capability to add additional interventions over time. The evaluation of any new interventions will be governed by this master protocol and M-SAP (including adaptive algorithm and decision criteria), with any planned deviations from the master protocol and M-SAP to be specified in arm-specific appendices. The inclusion of any new interventions will require additional arm-specific appendices to the master protocol and M-SAP and will be implemented as a substantial amendment to regulatory bodies.

5.2 Open platform trial

5.2.1 Primary efficacy endpoints and analyses

The primary efficacy endpoint is all-cause, non-elective hospitalisation and/or death within 28 days of randomisation ascertained through patient/trial partner report, and/or patient medical records.

5.2.2 Primary efficacy hypothesis & analysis

Let p_j denote the probability of hospitalisation/death for persons in treatment group j, where j = 0 denotes the Usual Care arm. A Bayesian posterior distribution will be derived for the estimated difference in probability of hospitalisation/death between treatment groups. Let ϑ_j denote the log odds ratio of hospitalisation/death comparing intervention j to Usual Care. The primary analysis for intervention j will test the following hypothesis:

$$H_0 : \theta_j \geq 0$$
$$H_1 : \theta_j < 0$$

If the Bayesian posterior probability of beneficial treatment effect (alternative hypothesis) is greater than or equal to a pre-specified threshold (e.g., 0.98), the null hypothesis will be rejected, and the intervention will be deemed superior to Usual Care with respect to Hospitalisation/Death in the primary analysis population. The exact threshold will be pre-specified and calibrated via simulation in the Adaptive Design Report to demonstrate control of Type I error at the traditional 0.05 two-sided level for each intervention, accounting for multiple interim analyses.

The analysis of primary and some secondary outcome data analysis will be performed by Berry Consultancy with support from statisticians at the University of Oxford. The company is based in the USA; however, no identifiable data will be given to them during this process.

5.2.3 Adaptive design

The pre-specified design will allow adaptations to the trial based on the observed primary endpoint data. These adaptations include the declaration of success or futility of an intervention at an interim analysis and the removal of treatment arms based on pre-specified decision criteria. The adaptive algorithm will be documented in the Adaptive Design Report, including pre-specified criteria for decisions regarding futility or effectiveness of interventions and/or replacing interventions in the trial.

For peer review only - http://bmjopen.bmj.com/site/about/guidelines.xhtml
© Copyright: The University of Oxford and Oxford University Hospitals NHS Foundation Trust 2019

The Adaptive Design Report (ADR) will contain extensive simulations to explore the performance of the adaptive design, including power and Type I error. Due to the urgent nature of the pandemic situation, this comprehensive ADR will be developed and finalised prior to the first scheduled interim analysis by a blinded statistician.

5.2.4 Interim analyses

Precise timing of the first interim analysis and frequency of subsequent interim analyses will be pre-specified in the Adaptive Design Report and DSMC Charter, based on both simulations and logistical considerations.

5.2.5 Allocation & adaptive randomisation

Participants will be randomised to one trial arm using fixed equal allocation ratios corresponding to the number of eligible arms in the trial. For instance, if there are two active interventions (A & B), the allocation ratio will be 1:1:1 for Usual Care, active A, active B (respectively), such that 33% of participants are randomised to Usual Care. If there are 3 active interventions, the allocation ratio will be 1:1:1:1, such that 25% of participants are randomised to Usual Care. As this is a nationwide, individually randomised trial that aims to include large numbers of participants, individual participant characteristics and infecting strain types of the infecting agent should be equally distributed between trial arms.

5.2.6 Sample size justification

Main Trial

The primary analysis will incorporate Bayesian logistic regression to estimate the odds ratio for hospitalisation/death for a treatment arm versus control, adjusting for age, vaccination status, and comorbidity status. An experimental treatment will be considered superior to the control if the Bayesian posterior probability of benefit is greater than a pre-specified threshold (e.g., 0.98) as detailed in the Adaptive Design Report. The trial design will incorporate multiple interim analyses that allow each intervention to stop early for futility, stop early for superiority, or continue to randomise participants. Additional interventions may be added as appendices to the master protocol throughout the duration of the trial. Extensive simulations will be conducted to evaluate and understand the operating characteristics and performance of the adaptive algorithm, such as control of Type I error and stopping guidance for efficacy and futility. Type I error will be controlled at the traditional 0.05 two-sided level for each intervention. A statistical analysis plan will be prepared and finalised before the first scheduled interim analysis.

The primary analysis will include those allocated to a particular antiviral agent and to the control condition (Usual Care) only during the period that that antiviral agent was in the trial (concurrently randomised population). A sensitivity analysis of the effect of subsequently introduced agents will include relevant control participants recruited prior to the introduction of that agent. To account for changes in the standard treatment in the Usual Care arm in this sensitivity analysis, and in changing patterns of recovery due to possible new variants, immunisations, behavioural interventions and other factors, this analytic model will include parameters to adjust for this temporal drift in the trial population, by estimating the primary endpoint in the usual care group across time via Bayesian hierarchical modelling.

For peer review only - http://bmjopen.bmj.com/site/about/guidelines.xhtml
© Copyright: The University of Oxford and Oxford University Hospitals NHS Foundation Trust 2019

NUFFIELD DEPARTMENT OF PRIMARY CARE HEALTH SCIENCES Primary Care Clinical Trials Unit PANORAMIC Platform Adaptive trial of NOvel antiviRals for eArly treatMent of COVID-19 In the Community

Should an intervention demonstrate superiority versus Usual Care, the superior intervention may become included in Usual Care and so become part of the control arm for subsequent interventions. Additionally, the Bayesian secondary analysis model will provide "bridging" across overlapping treatment groups through the temporal parameters, which will enable comparisons of subsequent interventions to the original Usual Care, even if there are no concurrent randomisations to the original Usual Care.

If there are important changes in Usual Care due to the introduction of new and superior interventions, the Trial Management Group will assess whether any design feature (such as futility and superiority criteria) need to be re-considered.

We estimated that the hospitalisation/date rate will be reduced to 3% in the Usual Care arm.

Based on the unblinded data from the PRINCIPLE Trial that the overall estimated hospitalisation/death was 8.8% in the Usual Care arm for the period that Budesonide was open for recruitment. However, the percentage of fully vaccinated participants was lower than the current percentage. Subsequent blinded data from PRINCIPLE has observed the overall COVID-19 related hospitalisation/death was 3.8% between 27 May 2021 and 25 July 2021 (8, 9). So, we believe our estimated based rate is not overly overestimated for the primary outcome defined as all-cause hospitalisation/death. Although vaccine has been efficacious on preventing hospitalisation, there is still a sub-population of unvaccinated cohort that is at higher risk of hospital admission/death. The adaptive nature of the platform trial means that the recruitment will continue until a pre-specified probability of superiority or futility thresholds is met.

An estimated maximum of approximately 5300 participants per arm will be required to provide approximately 90% power for detecting a 33% relative reduction in the hospitalisation/death in an experimental arm relative to Usual Care, based on the assumption of an underlying 3% combined hospitalisation/death rate in the Usual Care arm, and an intervention lowering the hospitalisation/death rate to 2%. We expect fewer participants will be needed to detect the same relative reduction if the event rate is larger than 3% in the Usual Care arm (Table 1), or if there is a greater reduction in the relative risk of hospitalisation/death for a given intervention. However, should the event rate be lower than expected, then the target sample size will be increased to reflect this.

Table 1: Power and sample size estimates for PANORAMIC per treatment arm

**90% power**			**80% power**		
**Usual Care**	**Treatment**	**Sample size**	**Usual Care**	**Treatment**	**Sample size**
1.0%	0.67%	16578	1.0%	0.67%	12534
1.5%	1.0%	10771	1.5%	1.0%	8145
2.0%	1.3%	7241	2.0%	1.3%	5480
3.0%	2.0%	5319	3.0%	2.0%	4023
4.0%	2.7%	4177	4.0%	2.7%	3159
5.0%	3.4%	3425	5.0%	3.4%	2590

For peer review only - http://bmjopen.bmj.com/site/about/guidelines.xhtml

© Copyright: The University of Oxford and Oxford University Hospitals NHS Foundation Trust 2019

Simulations are used to further quantify the statistical power for each experimental arm in the context of an adaptive design, as well as general performance characteristics, as detailed in the Adaptive Design Report.

Virology Sampled Cohort
Simulations from a viral dynamic model from early 2020 [10] suggests that 30 patients per arm will detect a 2.5-fold increase in viral clearance (undetectable viral load at day 7) in patients who start therapy within 5 days of symptom onset (90% power; alpha 0.05). Clinical improvement may be associated with smaller decreases in viral load, and viral dynamic modelling leveraging time series viral load data can detect much smaller drug effect sizes [11]. 300 patients will provide a 95% probability of seeing at least one example of a mutation occurring in 1% or more of patients.

5.2.7 Virtual trial simulations

Virtual trial simulations are used to demonstrate good performance and adequate control of Type I error for the adaptive design. Simulations will be provided in the Adaptive Design Report.

5.2.8 Procedure for accounting for missing, unused, and spurious data

Full details of handling missing data will be specified in the M-SAP.

5.3 Primary analysis population

For each intervention, the primary analysis population will include all concurrently randomised patients that were eligible to be randomised to the intervention (concurrent and eligible) and Usual Care. The primary analysis will use trial participants who fulfil the eligibility criteria and have had the opportunity to complete 28 days of follow-up. Eligible participants will be analysed according to the group they were randomised to regardless of deviation from the protocol. All other analysis populations will be defined in the M-SAP.

Complier Average Causal Effect (CACE) modelling will be undertaken to account for adherence.

5.4 Procedures for reporting unplanned deviation(s) from the M-SAP

Analyses will be carried out in accordance with the M-SAP and corresponding appendices. Any additional analysis that is not specified in the M-SAP/appendices or any unplanned deviation(s) from the M-SAP/appendices will be specified in the Statistical Analysis Report. Reasons for these changes will be documented and authorised by the CI.

6. DATA MANAGEMENT

The data management aspects of the trial are summarised here with details fully described in the Data Management Plan.

6.1 Source data

Source documents are where data are first recorded. These include, but are not limited to, hospital/medical records (from which medical history and previous and concurrent medication may be summarised into the CRF), NHS Digital, eDRIS, SAIL and HSC NI data, clinical and office charts, laboratory and pharmacy records, diaries, microfiches, radiographs, and correspondence.

For peer review only

For peer review only - http://bmjopen.bmj.com/site/about/guidelines.xhtml
© Copyright: The University of Oxford and Oxford University Hospitals NHS Foundation Trust 2019

NUFFIELD DEPARTMENT OF PRIMARY CARE HEALTH SCIENCES Primary Care Clinical Trials Unit PANORAMIC Platform Adaptive trial of NOvel antiviRals for eArly treatMent of COVID-19 In the Community

If a participant fails to complete data online and after six attempts at contacting the participant/Trial Partner, any sources of routinely collected data may be utilised to obtain missing data. Data collected will include participant identifiable information and will be accessed at the University of Oxford according to PC-CTU Information Governance policies and UK GDPR. Data will only be held for the duration it is required; this will be reviewed annually.

CRF entries will be considered source data if the CRF is the site of the original recording (e.g., there is no other written or electronic record of data). All documents will be stored safely in confidential conditions. On all trial-specific documents, other than the signed consent, the participant will be referred to by the trial participant number/code, not by name.

6.2 Access to data

Direct access will be granted to authorised representatives from the Sponsor, host institution, centres in other UK Devolved Administrations and the regulatory authorities to permit trial-related monitoring, audits, and inspections.

6.3 Data recording and record keeping

The Investigators will maintain appropriate medical and research records for this trial, in compliance with the requirements of the Medicines for Human Use (Clinical Trial) Regulations 2004, ICH E6 GCP and regulatory and institutional requirements for the protection of confidentiality of volunteers. The CI, PI, Co-Investigators, clinical team, including Clinical Research Nurses, and other authorised members of the trial team will have access to records. The Investigators will permit authorised representatives of the sponsor, and regulatory agencies to examine (and when required by applicable law, to copy) clinical records for the purposes of quality assurance reviews, audits and evaluation of the trial safety and progress.

The data will be entered into CRFs in an electronic format by the participant, trial Partner, Hub team member or trial team using an FDA part 11B compliant database. Data is stored on a secure cloud hosted server physically located in London, UK. Data will be entered in a web browser and then transferred to the database by encrypted (Https) transfer. This includes safety data, laboratory data and outcome data. Safety data will be collected through electronic diaries. Risks are mitigated using the ISO97001 framework.

An online secure data entry system designed to collect sensitive data, such as participant and Trial Partner contact details, will be used. All identifiable participant data is encrypted using the Advanced Encryption Standard. The participant portal will also manage online eligibility, eConsent and ePRO. Participant and Trial Partner data will be kept and stored securely for as long as it's required by the trial and reviewed on annual basis.

7. QUALITY ASSURANCE PROCEDURES

The trial will be conducted in accordance with the current approved protocol, GCP, relevant regulations and PC-CTU Standard Operating Procedures. All PIs, coordinating centre staff and site staff will receive training in trial procedures according to GCP where required. Regular monitoring will be performed according to GCP using a risk-based approach. Data will be evaluated for compliance with the protocol and accuracy in relation to source documents where possible.

For peer review only - http://bmjopen.bmj.com/site/about/guidelines.xhtml
© Copyright: The University of Oxford and Oxford University Hospitals NHS Foundation Trust 2019

The PC-CTU Trial Management Group will be responsible for the monitoring of all aspects of the trial's conduct and progress and will ensure that the protocol is adhered to, and that appropriate action is taken to safeguard participants and the quality of the trial itself. The TMG will be comprised of individuals responsible for the trial's day to day management and will meet regularly throughout the course of the trial.

7.1 Risk assessment and monitoring

A risk assessment and monitoring plan will be prepared before the trial opens for each antiviral agent and will be reviewed as necessary over the course of the trial to reflect significant changes to the protocol or outcomes of monitoring activities. Monitoring will be performed by the PC-CTU Quality Assurance Manager or delegate. The level of monitoring required will be informed by the risk assessment.

7.2 Trial committees

The composition, roles and responsibilities of committee are detailed in their respective charters except for the core project team and AT however their basic functions are as follows:

- Data and Safety Monitoring Committee (DSMC) will review the data received from the SAC at each interim analysis as described in the Statistical Analysis section, in order to ensure that the process is working correctly and to review and monitor the accruing data to ensure the rights, safety and wellbeing of the trial participants. Composition, and roles and responsibilities of the DSMC are detailed in the DSMC charter. The DSMC reviews data from interim analyses and makes recommendations to the TSC about antiviral agent s that have reached pre-specified thresholds for futility, success, or for which safety concerns have emerged
- Trial Steering Committee (TSC) will ensure the rights, safety, and wellbeing of the trial participants. They will make recommendations about how the trial is operating, any ethical or safety issues and any data being produced from other relevant studies that might impact the trial. Composition, and roles and responsibilities of the TSC are detailed in the TSC charter. The TSC advises the TMG about the conduct of the trial and stopping randomisation to trial arms (based on recommendations received from the DSMC and/or relevant information external to the trial), and the addition of new trial arms
- The Statistical Analysis Committee (SAC) will perform interim analysis and report these to the DSMC. The TMG will remain blind to these interim analyses until a recommendation is received form the TSC about stopping randomisation or safety concerns.
- Enhanced Safety Group (ESG) will review accumulating safety data in accordance with the ISA for each antiviral. The ESG will also provide advice and guidance to the relevant trial committees regarding the safety monitoring requirements for antiviral agents depending on their known safety profile
- Trial Management Group (TMG) – will be responsible for the day-to-day running of the trial, including monitoring all aspects of the trial and ensuring that the protocol is being adhered to. It will include Co-Investigators and will meet weekly in the first instance
- A project team from within the TMG will meet weekly or as required for operational decision making (meet daily at the start of the trial)
- The AT will advise on the antiviral agents to be included in the PANORAMIC trial

For peer review only

For peer review only - http://bmjopen.bmj.com/site/about/guidelines.xhtml
© Copyright: The University of Oxford and Oxford University Hospitals NHS Foundation Trust 2019

8. PROTOCOL DEVIATIONS

A trial related deviation is a departure from the ethically approved trial protocol or other trial document or process (e.g., consent process or administration of trial intervention) or from Good Clinical Practice (GCP) or any applicable regulatory requirements. Any deviations from the protocol will be documented in a protocol deviation form and filed in the trial master file.

A PC-CTU SOP is in place describing the procedure for identifying non-compliances, escalation to the central team and assessment of whether a non-compliance /deviation may be a potential Serious Breach.

9. SERIOUS BREACHES

A “serious breach” is a breach of the protocol or of the conditions or principles of Good Clinical Practice which is likely to affect to a significant degree:

(a) the safety or physical or mental integrity of the trial subjects; or
(b) the scientific value of the research.

In the event that a serious breach is suspected the Sponsor must be contacted within one working day. In collaboration with the CI, the serious breach will be reviewed by the Sponsor and, if appropriate, the Sponsor will report it to the approving REC committee and the relevant NHS host organisation within seven calendar days.

10. ETHICAL AND REGULATORY CONSIDERATIONS

10.1 Declaration of Helsinki

The Investigators will ensure that this trial is conducted in accordance with the principles of the Declaration of Helsinki.

10.2 Guidelines for Good Clinical Practice

The Investigators will ensure that this trial is conducted in accordance with relevant regulations and with Good Clinical Practice.

10.3 Approvals

Following Sponsor approval, the protocol, informed consent form, participant information sheets and any proposed informing material will be submitted to an appropriate Research Ethics Committee (REC), regulatory authorities, and host institution(s) for written approval. The PI and coordinating centres for each country will ensure and confirm correct regulatory approvals are gained prior to recruitment.

The Investigator will submit and, where necessary, obtain approval from the above parties for all substantial amendments to the original approved documents.

For peer review only - http://bmjopen.bmj.com/site/about/guidelines.xhtml
© Copyright: The University of Oxford and Oxford University Hospitals NHS Foundation Trust 2019

10.4 Other ethical considerations

If a particular arm is deemed futile and dropped, no further participants will be randomised to this arm and anyone who is currently on this arm will be informed it has been dropped.
Once a particular intervention has been declared superior and effective, that may become the comparator arm (i.e., standard care).

Participants who lack capacity to consent for themselves will only be recruited after consultation with their legal representative. Any sign of dissent in any form from the participant who lacks capacity to consent for themselves will be taken as an indication they do not wish to be involved and they will be withdrawn. Only residents of care homes who lack capacity to consent will be recruited, adults who lack capacity to consent will not be recruited from the wider community.

10.5 Reporting

The CI shall submit once a year throughout the clinical trial, or on request, an Annual Progress Report to the REC, HRA (where required), host organisation, funder (where required) and Sponsor. In addition, an End of Trial notification and final report will be submitted to the MHRA, the REC, host organisation and Sponsor.

10.6 Transparency in research

Prior to the recruitment of the first participant, the trial will have been registered on the ISRCTN Database. Results will be uploaded to this register within 12 months of the end of trial date as given on the end of trial declaration by the CI or their delegate. Where the trial has been registered on multiple public platforms, the trial information will be kept up to date during the trial, and the CI or their delegate will upload results to all those public registries within 12 months of the end of the trial declaration.

10.7 Participant confidentiality

The trial will comply with the UK General Data Protection Regulation (GDPR) and Data Protection Act 2018, which require data to be anonymised as soon as it is practical to do so. The processing of the personal data of participants will be minimised by making use of a unique participant trial number only on all trial documents and any electronic database(s). All documents will be stored securely and only accessible by trial staff and authorised personnel. The trial staff will safeguard the privacy of participants' personal data.

10.8 Expenses and benefits

All participants will be reimbursed with a £10 voucher as a token of recognition of giving their time and contribution to the trial. There will be no prescription charges for trial antiviral agents incurred by trial participants.

11. FINANCE AND INSURANCE

11.1 Funding

The trial is funded by the Department of Health and Social Care and the NIHR.
The Department of Health and Social Care will provide the antiviral agents to be evaluated in the trial without cost to the trial budget for trial use.

11.2 Insurance

For peer review only - http://bmjopen.bmj.com/site/about/guidelines.xhtml
© Copyright: The University of Oxford and Oxford University Hospitals NHS Foundation Trust 2019

The University has a specialist insurance policy in place, which would operate in the event of any participant suffering harm as a result of their involvement in the research (Newline Underwriting Management Ltd, at Lloyd's of London). NHS indemnity operates in respect of the clinical treatment that is provided.

11.3 Contractual arrangements

Appropriate contractual arrangements will be put in place with all third parties.

12. PUBLICATION POLICY

The Investigators (those listed on the protocol and others to be decided at publication) will be involved in reviewing drafts of the manuscripts, abstracts, press releases and any other publications arising from the trial. Authors will acknowledge the trial funders. Authorship will be determined in accordance with the ICMJE guidelines and other contributors will be acknowledged.

13. DEVELOPMENT OF A NEW PRODUCT/ PROCESS OR THE GENERATION OF INTELLECTUAL PROPERTY

Ownership of IP generated by employees of the University vests in the University. The University will ensure appropriate arrangements are in place as regards any new IP arising from the trial.

14. ARCHIVING

Archiving will be done according to PC-CTU SOP and trial specific working instructions. Research documents with personal information, such as consent forms, will be held securely at the University of Oxford's archiving facility according to the PC-CTU Archiving SOP.

For peer review only

For peer review only - http://bmjopen.bmj.com/site/about/guidelines.xhtml
© Copyright: The University of Oxford and Oxford University Hospitals NHS Foundation Trust 2019

For peer review only

For peer review only - http://bmjopen.bmj.com/site/about/guidelines.xhtml

© Copyright: The University of Oxford and Oxford University Hospitals NHS Foundation Trust 2019

15. APPENDIX A: SCHEDULE OF PROCEDURES

Main Trial

Procedures	Day 0	Day 0	Day 0	Day 1	Day 2	Day 0	Day 5	Daily Diary 1-28 and 3 and 6 months	Day 0 -12 months	Up to 10 years
	Screening completed by participant online/ phone	Baseline completed by participant online/ phone	Re-affirm consent and Eligibility completed by Clinician online/ phone	Telephone call: confirm receipt of participant pack	Telephone call to all participants	Antivirals requiring face-to-face recruitment (As defined in its ISA)		Symptom Diaries completed by participant online/ phone	Retrospective data collection by trial team	Data extraction from routine clinical records
						Screening/Baseline by Clinician face to face	Safety Review by Clinician face to face			
Informed consent	X	X	X			X	X	X		
Questionnaire	X	X						X		
Pregnancy test confirmation				X	X			X*		
Demographics	X	X				X			X	
Medical history	X	X	X			X			X	

For peer review only

For peer review only - http://bmjopen.bmj.com/site/about/guidelines.xhtml
© Copyright: The University of Oxford and Oxford University Hospitals NHS Foundation Trust 2019

NUFFIELD DEPARTMENT OF PRIMARY CARE HEALTH SCIENCES
Primary Care Clinical Trials Unit

PANORAMIC
Platform Adaptive trial of NOvel antiviRals for eArly treatMent of COVID-19 In the Community

Physical examination						X	X			
Concomitant medications		X	X			X		X**	X	
Vital signs measurements (if specified in ISA)						X				
Eligibility assessment	X		X			X				
Randomisation			X			X				
Dispensing of trial drugs			X			X				
Administer drug in clinic						X				
Post drug observation (for high-risk antivirals)						X				
Compliance								X		
Primary endpoint and secondary outcomes								X	X	X
AE assessments					X	X	X	X		
Safety bloods						X	X			
Evidence of sequalae and health care utilisation										X

* Days 1-3 only ** Daily symptom diaries will collect information on concomitant medications as specified in the antiviral ISA

For peer review only

For peer review only - http://bmjopen.bmj.com/site/about/guidelines.xhtml
© Copyright: The University of Oxford and Oxford University Hospitals NHS Foundation Trust 2019

Virology Sampled Cohort (as well as procedures described for the main trial and only for the first 300 patients who consent for this cohort in **each** arm of the trial)

	Baseline (Day 1, before first dose)	Day 2	Day 3	Day 4	Day 5	Day 6	Day 7	Day 14
**First 30 intensive sampled cohort participants****								
Virology sampling (nasopharyngeal swabs) at Hub or home	X	X	X	X	X	X	X	X*
Finger prick antibody test	X				X			X*
**Next viral 270 less intensive sampled cohort participants*****								
Virology sampling (nasopharyngeal swab sample (self-swab)	X				X*			X*
Finger prick antibody test	X				X*			X*

* +/- 1 day from randomisation ** To be evaluable for the intensive sampled cohort participants must return: i) a minimum of three nasopharyngeal swabs on Day 1, Day 4 and Day 7 and two finger prick blood tests on Day 1 and either Day 5 or Day 15. ***To be evaluable for the less intensive sampled cohort participants must return a minimum of two nasopharyngeal swabs on Day 1 and either the Day 5 or Day 14 and two finger prick blood tests on Day 1 and either Day 5 or Day 15.

For peer review only - http://bmjopen.bmj.com/site/about/guidelines.xhtml
© Copyright: The University of Oxford and Oxford University Hospitals NHS Foundation Trust 2019

16. APPENDIX B: Participant Flow Diagram

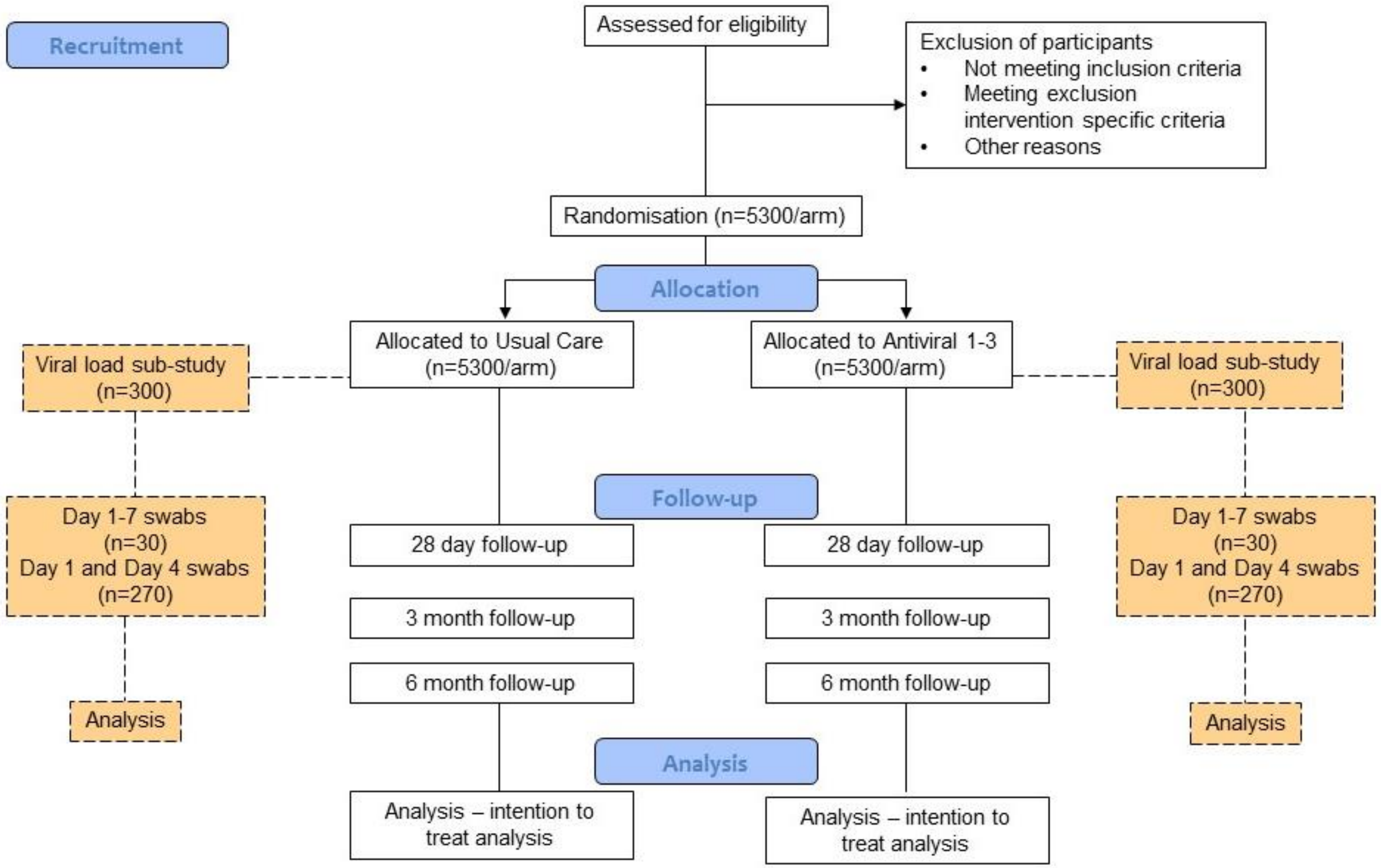

For peer review only - http://bmjopen.bmj.com/site/about/guidelines.xhtml
© Copyright: The University of Oxford and Oxford University Hospitals NHS Foundation Trust 2019

NUFFIELD DEPARTMENT OF PRIMARY CARE HEALTH SCIENCES Primary Care Clinical Trials Unit

PANORAMIC Platform Adaptive trial of NOvel antiviRals for eArly treatMent of COVID-19 In the Community

17. APPENDIX C: AMENDMENT HISTORY

Amendment No.	Protocol Version No.	Date issued	Author(s) of changes	Details of Changes made
Initial REC/MHRA submission	1.1	11/11/2021	Mina Davoudianfar	Replaced the word 'tablets' with 'capsules' in Molnupiravir ISA.
Initial REC/MHRA submission	1.2	18/11/2021	Mina Davoudianfar	Changes made in response to comments from REC review:<br>Removal of wording which allows recruitment of patients who lack capacity to consent, in a care home only.<br>Clarification of Day 1 and Day 2 phone calls.
Non-Substantial Amendment 1	1.3	24/11/2022	Mina Davoudianfar	Reinstated wording to include participants lacking capacity, to only be recruited from care homes, following request of Sponsor.
Substantial Amendment 1	1.4	17/01/2022	Tracie Madden	Changed health care providers to health service providers including government agencies e.g., UK Health Security Agency.
Substantial Amendment 2	2.0	02/03/2022	Tracie Madden	Added the Virology Sampled Cohort including sample processing and labelling requirements.
				Added Paxlovid as a new intervention.
				Updated information on contraception, following discussions with MHRA.
				Added that informed consent can be taken by a prescribing pharmacist, if specified in the relevant ISA.
				Provided clarification around change to the professional roles that each

For peer review only - http://bmjopen.bmj.com/site/about/guidelines.xhtml
© Copyright: The University of Oxford and Oxford University Hospitals NHS Foundation Trust 2019

				HCP (medically qualified clinicians, research nurses and prescribing pharmacists) can have with respect to assessing participant eligibility for randomisation to antiviral agents.
				Amended follow-up and medication adherence sections to reflect the fact that the Hubs are now recruiting.
				Added that informed consent will be sought from participants partner to collect pregnancy follow-up data.
				Added details for the members of trial oversight committees and referenced the committee charters where appropriate.
				Updated the sample size justification in case of a lower than anticipated event rate.
				Revised the definition of the primary analysis population and secondary outcome measures for clarity.
				Updated the participant flow diagram to reflect inclusion of the Virology Sampled Cohort.
				Added in Lateral Flow Test as an alternative to PCR for trial entry and removed the requirement for a confirmatory PCR test for participants to be included in the main analysis.
				Added two new co-investigators.
				Added in a statement to reflect that the main PIS has been edited to highlight to

For peer review only

For peer review only - http://bmjopen.bmj.com/site/about/guidelines.xhtml
© Copyright: The University of Oxford and Oxford University Hospitals NHS Foundation Trust 2019

NUFFIELD DEPARTMENT OF PRIMARY CARE HEALTH SCIENCES | Primary Care Clinical Trials Unit | PANORAMIC Platform Adaptive trial of NOvel antiviRals for eArly treatMent of COVID-19 In the Community

				potential participants, eligible for direct access to antivirals, that they can receive antiviral treatments out with the trial. DHSC approved table of potentially eligible cohorts added.
				Performed minor text corrections throughout.
				Added in details and function of ESG.
Substantial Amendment 3	3.0	25/03/22	Tracie Madden	Updated RSI and Paxlovid ISA, at the request of the MHRA, to reflect new information in the Paxlovid SmPC updated on 02/03/22.
				Inserted a statement, at the request of the MHRA, to state that a protocol substantial amendment will be required to be submitted for regulatory approval when List B in the Paxlovid ISA is modified.
				Replaced reference to access to a participants Summary Care Record including medication list as being sufficient to assess eligibility for entry into the Paxlovid trial arm with access primary care record.
				Updated references 21 and 22.
				Updated date of Molnupiravir RSI.
				Updated the risk mitigation strategies for drug interactions and side effect monitoring in the Paxlovid ISA at the request of the MHRA.
				Inserted Appendix F: standard script for safety monitoring of drugs that require adjustment when

For peer review only

For peer review only - http://bmjopen.bmj.com/site/about/guidelines.xhtml
© Copyright: The University of Oxford and Oxford University Hospitals NHS Foundation Trust 2019

				co-administered with Paxlovid at the request of the MHRA.
				Updated safety monitoring procedure for overdose in the Molnupiravir and Paxlovid ISAs at the request of the ESG.
				Updated AE reporting sections in the Molnupiravir and Paxlovid ISAs at the request of the ESG.
				Performed minor text corrections throughout.
				Appendix A: Schedule of Procedures updated to state that the daily symptom diary will collect information on concomitant medications as specified in the antiviral ISA.
				Removed website links to all RSI.
				Definitions of evaluable participants for the intensive and less intensive Virology sampled cohorts added to schedule of procedures for Virology sampled cohorts at request of TMG.
Substantial Amendment 4	4.0	20/04/2022	**Elizabeth Hadley**	Replacing a COPI notice used to recruit participants which expires in June 2022 with a CAG Approval.
Substantial Amendment 5	5.0	09/05/2022	**Julie Allen**	Updating of eligibility assessment for Paxlovid. To include information relating to standard prescribing practices across the UK, who can perform eligibility assessments and which medical records can be used. Update PPI Members. Update the use of national data collection agencies in all devolved nations.

For peer review only

For peer review only - http://bmjopen.bmj.com/site/about/guidelines.xhtml
© Copyright: The University of Oxford and Oxford University Hospitals NHS Foundation Trust 2019

Lists details of all protocol amendments whenever a new version of the protocol is produced.

Protocol amendments must be submitted to the funder and Sponsor for approval prior to submission to the REC committee, HRA (where required) and/or MHRA.

18. APPENDIX D: SUPPLEMENTARY MATERIAL

A. Abbreviations

AE	Adverse event
AR	Adverse reaction
AT	Antiviral Taskforce
CI	Chief Investigator
CRF	Case Report Form
CT	Clinical Trials
CTA	Clinical Trials Authorisation
DHSC	Department of Health and Social Care
DSMC	Data Monitoring Committee / Data and Safety Monitoring Committee
DSUR	Development Safety Update Report
eDRIS	Electronic Data Research and Innovation Service
ESG	Enhanced Safety Group
GCP	Good Clinical Practice
GPDPR	General Practice Data for planning and research
HSC NI	Health and Social Care Northern Ireland (HSC Business Services Organisation/HSC Trusts)
GP	General Practitioner
HRA	Health Research Authority
HCP	Healthcare Professional
IB	Investigators Brochure
ICF	Informed Consent Form
ICH	International Conference on Harmonisation
IMP	Investigational Medicinal Product
ISA	Intervention Specific Appendix
MHRA	Medicines and Healthcare products Regulatory Agency
NHS	National Health Service
NIHR	National Institute of Health Research
RES	Research Ethics Service
PI	Principal Investigator
PIS	Participant/ Patient Information Sheet
R&D	NHS Trust Research and Development Department
RCGP RSC	Royal College of General Practitioners Research Surveillance Centre

For peer review only

For peer review only - http://bmjopen.bmj.com/site/about/guidelines.xhtml
© Copyright: The University of Oxford and Oxford University Hospitals NHS Foundation Trust 2019

REC	Research Ethics Committee
RGEA	Research Governance, Ethics and Assurance
RSI	Reference Safety Information
SAE	Serious Adverse Event
SAIL	The Secure Anonymised Information Linkage Databank
SAR	Serious Adverse Reaction
SDV	Source Data Verification
SmPC	Summary of Medicinal Product Characteristics
SOP	Standard Operating Procedure
TSC	Trial Steering Committee
SUSAR	Suspected Unexpected Serious Adverse Reactions
TMF	Trial Master File
UCL	University College London
UKTIS	UK Teratology Service

B. Key trial contacts

**Chief Investigator**	Professor Chris Butler<br>Nuffield Department of Primary Care Health Sciences<br>Gibson Building<br>Radcliffe Observatory Quarter<br>Woodstock Road<br>Oxford<br>OX2 6GG<br>christopher.butler@phc.ox.ac.uk
**Sponsor**	Research Governance, Ethics and Assurance (RGEA)<br>Joint Research Office<br>1st floor, Boundary Brook House<br>Churchill Drive,<br>Headington<br>Oxford OX3 7GB<br>ctrg@admin.ox.ac.uk<br>Tel: +44 (0)1865616480
**Funder(s)**	UKRI/NIHR
**Clinical Trials Unit**	Primary Care Clinical Trials Unit,<br>Nuffield Department of Primary Care Health Sciences<br>Radcliffe Observatory Quarter<br>Woodstock Road<br>Oxford<br>OX2 6GG<br>panoramic@phc.ox.ac.uk<br>Tel: TBC
**Statistician**	Dr Ben Saville,<br>Berry Consultants,<br>Austin, Texas, USA,

For peer review only

For peer review only - http://bmjopen.bmj.com/site/about/guidelines.xhtml
© Copyright: The University of Oxford and Oxford University Hospitals NHS Foundation Trust 2019

	And<br>Department of Biostatistics,<br>Vanderbilt University School of Medicine,<br>Nashville, Tennessee, USA<br><br>Dr Ly-Mee Yu<br>Primary Care Clinical Trials Unit,<br>Nuffield Department of Primary Care Health Sciences<br>Radcliffe Observatory Quarter<br>Woodstock Road<br>Oxford<br>OX2 6GG
**Committees**	**DSMC Chair:**<br>Prof Deborah Ashby<br>Faculty of Medicine, School of Public Health<br>Imperial College London<br>deborah.ashby@imperial.ac.uk<br><br>**DSMC Members:**<br>Prof Simon Gates<br>Cancer Research Clinical Trials Unit (CRCTU)<br>Institute of Cancer and Genomic Sciences<br>University of Birmingham<br>S.Gates@bham.ac.uk<br><br>Prof Gordon Taylor<br>University of Exeter<br>g.j.taylor@exeter.ac.uk<br><br>Dr Benjamin Fisher<br>Institute of Inflammation and Ageing<br>University of Birmingham Research Labs<br>Queen Elizabeth Hospital<br>B.Fisher@bham.ac.uk<br><br>Prof Martin Underwood<br>Warwick Clinical Trials Unit<br>Warwick Medical School<br>The University of Warwick<br>M.Underwood@warwick.ac.uk
	**TSC Chair:**<br>Prof Philip Hannaford<br>University of Aberdeen<br>p.hannaford@abdn.ac.uk

For peer review only - http://bmjopen.bmj.com/site/about/guidelines.xhtml
© Copyright: The University of Oxford and Oxford University Hospitals NHS Foundation Trust 2019

NUFFIELD DEPARTMENT OF PRIMARY CARE HEALTH SCIENCES | Primary Care Clinical Trials Unit | PANORAMIC Platform Adaptive trial of NOvel antiviRals for eArly treatMent of COVID-19 In the Community

	**TSC Members:**<br>Prof Ranjit Lall<br>Warwick Clinical Trials Unit<br>Warwick Medical School<br>The University of Warwick<br>R.Lall@warwick.ac.uk<br><br>Prof Alastair Hay<br>Bristol Population Health Science Institute<br>Health Protection Research Unit (HPRU)<br>University of Bristol<br>alastair.hay@bristol.co.uk<br><br>Prof William Hollingworth<br>Bristol Population Health Science Institute<br>University of Bristol<br>William.Hollingworth@bristol.ac.uk<br><br>*PPI representatives*<br>Ms Corina Cheeks<br><br>**TSC Observers:**<br>Prof Matt Sydes<br>Institute of Clinical Trials and Methodology<br>90 High Holborn<br>2nd Floor<br>London<br><br>Prof Mike Moore<br>Primary Care Research Centre<br>Primary Care, Population Sciences and Medical Education (PPM)<br>Faculty of Medicine<br>University of Southampton
	**ESG Chair:**<br>Prof Najib Rahman<br>Oxford Respiratory Trials Unit<br>University of Oxford<br>najib.rahman@ndm.ox.ac.uk<br><br>**ESG Members:**<br>Professor Duncan Richards<br>Oxford Clinical Trial Research Unit<br>University of Oxford<br>duncan.richards@ndorms.ox.ac.uk<br><br>Professor Paramjit Gill<br>Department of Health Sciences<br>Warwick Medical School

For peer review only - http://bmjopen.bmj.com/site/about/guidelines.xhtml
© Copyright: The University of Oxford and Oxford University Hospitals NHS Foundation Trust 2019

	P.Gill1@warwick.ac.uk<br><br>Professor Gail Hayward<br>Department of Primary Care Health Sciences<br>University of Oxford<br>gail.hayward@phc.ox.ac.uk<br><br>Professor Andrew Ustianowski<br>Infectious Disease and Tropical Medicine, Manchester University NHS Foundation Trust<br>North Manchester General Hospital<br>North Manchester Care Organisation (Part of the Northern Care Alliance NHS Group)<br>Delaunays Road<br>Manchester M8 5RB. UK.<br>Andrew.Ustianowski@mft.nhs.uk

For peer review only

For peer review only - http://bmjopen.bmj.com/site/about/guidelines.xhtml
© Copyright: The University of Oxford and Oxford University Hospitals NHS Foundation Trust 2019

C. Objectives and outcome measures

	Objectives	Outcome Measures	Timepoint (s)
**Main Trial**			
**Primary**	To determine whether antiviral treatment in the community safely reduces non-elective hospitalisations/ deaths in higher risk, symptomatic patients with confirmed COVID-19	All cause, non-elective hospitalisation and/or death, within 28 days of randomisation	Within 28 days of randomisation<br>Patient report, Trial Partner report, HES/ONS/medical record data linkage
**Secondary**	To explore whether antiviral treatment affects:<br>1) Time to recovery (defined as the first instance that a participant report of feeling recovered from the illness).<br><br>2) Participant reported illness severity, reported by daily rating of how well participant feels, enabling identification of sustained recovery.<br><br>3) Duration of severe symptoms and symptom recurrence including time to alleviation of symptoms, time to initial reduction of severity of symptoms, time to sustained recovery, time to sustained alleviation of symptoms, number of days of severe symptoms and	1-3 Participant reports symptoms daily for 28 days and at 3 and 6 months.	1-3 Daily online symptom scores. Telephone call or text on days 7, 14 and 28 if data is not obtained through the online diary. Also, at 3 and 6 months.

For peer review only

For peer review only - http://bmjopen.bmj.com/site/about/guidelines.xhtml
© Copyright: The University of Oxford and Oxford University Hospitals NHS Foundation Trust 2019

NUFFIELD DEPARTMENT OF PRIMARY CARE HEALTH SCIENCES Primary Care Clinical Trials Unit

PANORAMIC Platform Adaptive trial of NOvel antiviRals for eArly treatMent of COVID-19 In the Community

	worsening of symptoms.<br><br>4) Contacts with the health services.	4) Contacts with health services reported by patients and/or captured by reports of patients' medical records.	4) GP notes review if available through Oxford RCGP RSC network; otherwise, other sources of routinely collected data after 28 days. Medical notes review for up to 10 years.
	5) New infections in household.	5) Reports of new infections in the household from daily diary.	5) Daily online symptom scores or telephone call or text on days 7, 14 and 28.
	6) To investigate the safety of antiviral agents.	6) Evaluation of overall safety of drugs by the monitoring of AEs as defined in the ISAs).	6) For the duration of the antiviral course and a defined period after the antiviral finishes (see ISAs).
	7) Longer term effects including proportion with long covid, long covid symptoms, health care use and wellness.	7) Well-being, symptoms, and heath care utilisation.	7) Patient contact at three and six months, electronic medical record search for up to one year.
	8) Cost effectiveness.	8) Resource use and cost data and EQ-5D-5L.	8) Baseline and Day 28.
	**Objectives**	**Outcome Measures**	**Timepoint (s)**
**Virology Sampled Cohort**			

For peer review only - http://bmjopen.bmj.com/site/about/guidelines.xhtml

© Copyright: The University of Oxford and Oxford University Hospitals NHS Foundation Trust 2019

**Primary**	To determine whether antiviral treatment in the community reduces viral load to undetectable levels more quickly than untreated patients.	SARS-CoV-2 viral load.	Day 7.
**Secondary**	1) To determine whether antiviral treatment in the community leads to faster viral elimination rates than untreated patients.<br><br>2) To determine whether genetic mutations in the virus are more frequent in patients taking antiviral treatment compared with untreated patients.<br><br>3) To assess the impact of antibodies on viral load decline in patients taking antiviral treatment compared to with untreated patients.<br><br>4) To assess the antibody response on viral load decline in patients taking antiviral treatment compared with untreated patients.<br><br>5) (Exploratory endpoint) To compare viral load rate of decline in patients receiving	1) SARS-CoV-2 viral load.<br><br>2) SARS-CoV-2 viral genetic whole genome sequence.<br><br>3) SARS-CoV-2 viral load.<br><br>4) SARS-CoV-2 antibodies.<br><br>5) SARS-CoV-2 viral load.	1) Days 1-7, Day 14.<br><br>2) Day 1, Day 5, Day 14.<br><br>3) Day 1, Day 5, Day 14.<br><br>4) Day 1, Day 5, Day 14.<br><br>5) Days 1-7, Day 14.

For peer review only

For peer review only - http://bmjopen.bmj.com/site/about/guidelines.xhtml
© Copyright: The University of Oxford and Oxford University Hospitals NHS Foundation Trust 2019

NUFFIELD DEPARTMENT OF PRIMARY CARE HEALTH SCIENCES Primary Care Clinical Trials Unit

PANORAMIC Platform Adaptive trial of NOvel antiviRals for eArly treatMent of COVID-19 In the Community

	different antiviral therapies.		

D. Adverse Events

Definitions:	
**Adverse Event (AE)**	Any untoward medical occurrence in a participant to whom a medicinal product has been administered, including occurrences which are not necessarily caused by or related to that product.
**Adverse Reaction (AR)**	An untoward and unintended response in a participant to an investigational medicinal product which is related to any dose administered to that participant.<br><br>The phrase "response to an investigational medicinal product" means that a causal relationship between a trial medication and an AE is at least a reasonable possibility, i.e., the relationship cannot be ruled out.<br><br>All cases judged by either the reporting medically qualified professional or the Sponsor as having a reasonable suspected causal relationship to the trial medication qualify as adverse reactions.
**Serious Adverse Event (SAE)**	A SAE is any untoward medical occurrence that:<br>• results in death<br>• is life-threatening<br>• requires inpatient hospitalisation or prolongation of existing hospitalisation<br>• results in persistent or significant disability/incapacity<br>• consists of a congenital anomaly or birth defect*.<br><br>Other 'important medical events' may also be considered a SAE when, based upon appropriate medical judgement, the event may jeopardise the participant and may require medical or surgical intervention to prevent one of the outcomes listed above.<br><br>NOTE: The term "life-threatening" in the definition of "serious" refers to an event in which the participant was at risk of death at the time of the event; it does not refer to an event which hypothetically might have caused death if it were more severe.<br><br>*NOTE: Pregnancy is not, in itself an SAE. In the event that a participant or their partner becomes pregnant whilst taking part in a clinical trial or

For peer review only

For peer review only - http://bmjopen.bmj.com/site/about/guidelines.xhtml
© Copyright: The University of Oxford and Oxford University Hospitals NHS Foundation Trust 2019

NUFFIELD DEPARTMENT OF PRIMARY CARE HEALTH SCIENCES Primary Care Clinical Trials Unit
PANORAMIC Platform Adaptive trial of NOvel antiviRals for eArly treatMent of COVID-19 In the Community

	during a stage where the foetus could have been exposed to the medicinal product (in the case of the active substance or one of its metabolites having a long half-life) the pregnancy should be followed up by the investigator until delivery for congenital abnormality or birth defect, at which point it would fall within the definition of "serious".
**Serious Adverse Reaction (SAR)**	An AE that is both serious and, in the opinion of the reporting Investigator, believed with reasonable probability to be due to one of the trial antiviral agents, based on the information provided.
**Suspected Unexpected Serious Adverse Reaction (SUSAR)**	A SAR, the nature and severity of which is not consistent with the Reference Safety Information (RSI) for the medicinal product in question set out:<br>• in the case of a product with a marketing authorisation, in the approved summary of product characteristics (SmPC) for that product<br>• in the case of any other investigational medicinal product, in the approved investigator's brochure (IB) relating to the trial in question.

NB: To avoid confusion or misunderstanding the difference between the terms "serious" and "severe", the following note of clarification is provided: "Severe" is often used to describe intensity of a specific event, which may be of relatively minor medical significance. "Seriousness"

For peer review only

For peer review only - http://bmjopen.bmj.com/site/about/guidelines.xhtml
© Copyright: The University of Oxford and Oxford University Hospitals NHS Foundation Trust 2019

19. APPENDIX E: INTERVENTION SPECIFIC APPENDICES

1. USUAL CARE ARM

1. Background and rationale

This Usual Care arm will follow current NHS care provision and provides a control against which the effect of new interventions that are added to usual care can be assessed. If a new trial intervention plus Usual Care is found to be superior to Usual Care alone, then the Usual Care will evolve to include interventions that are recommended as part of standard care in the NHS. Usual Care in the trial will not be specified or mandated, and it will vary over time according to emerging evidence and evolving national recommendations and will be tailored by responsible clinicians to patient characteristics, clinical picture, and individual need. In addition, individual patients will vary in the self-care they choose to use, including use of over-the-counter medication. Use of key treatments such as monoclonal antibodies will be captured and considered in analyses.

2. Detail of intervention

Participants randomised to the usual care arm will receive usual clinical care as per NHS care delivery practice.

a. Investigational Medicinal Product (IMP) description

Not applicable

b. Storage of IMP

Not applicable

3. Safety reporting

Mechanisms for safety reporting are outlined in the trial protocol

For peer review only

For peer review only - http://bmjopen.bmj.com/site/about/guidelines.xhtml
© Copyright: The University of Oxford and Oxford University Hospitals NHS Foundation Trust 2019

2. USUAL CARE PLUS MOLNUPIRAVIR

1. Background

a. Potential mechanism of efficacy

Molnupiravir is an oral antiviral that was initially developed for treatment of influenza, but has now been developed for treatment and prevention of COVID-19.(12-14) It is a prodrug of the ribonucleoside analogue NHC that is incorporated into viral RNA by RNA-dependent RNA polymerase and inhibits viral replication by inducing *viral error catastrophe* (i.e. causing the build-up of viral mutations with each replication cycle that impair viral fitness).(14, 15)

b. Evidence for potential benefits of Molnupiravir in COVID-19 illness

Pre-clinical data

Molnupiravir has been shown *in vitro* to have a high barrier to resistance and to inhibit pathogenic coronaviruses (e.g., MERS-CoV, SARS-CoV-1, and SARS-CoV-2) (8). Data from mouse, (9) ferret (10) and Syrian hamster models (11) shows that Molnupiravir inhibits SARS-CoV-2 replication in vivo.

Phase 1 studies

A phase 1 trial among 130 healthy adults found that Molnupiravir was well tolerated with no signals of clinical concern. (12)

Phase 2/3 studies

As of 17-JUL-2020, 122 participants have received placebo or MK-4482 in single doses of 50 to 1600 mg or in multiple doses of 50 to 600 mg Q12H for 5.5 days. Molnupiravir was generally well tolerated in hospitalised and non-hospitalised participants. The proportion of participants with AEs, drug related AEs (per investigator), SAEs, and AEs leading to trial intervention discontinuation during the protocol-specified AE safety follow-up period were comparable across the intervention groups, with no apparent dose effect observed. One participant was discontinued from trial treatment because of a rash of moderate intensity, appearing following 3 days of dosing (6 doses) with 800 mg Q12H MK-4482 or placebo (blinded trial). No clinically meaningful trends were observed for changes in clinical laboratory values as a function of dose or treatment. In trial MK-4482-001 among hospitalised patients, there was a numerical imbalance in AEs resulting in death in participants treated with Molnupiravir (14/218, 6.4%) compared with placebo (2/75, 2.7%). However, Molnupiravir was well tolerated in both hospitalised (MK-4482-001) and non-hospitalised (MK-4482-002) participants with COVID-19, and there were no clinically meaningful differences in the incidence of AEs, SAEs, drug-related AEs, discontinuations due to AEs, and deaths observed when comparing Molnupiravir to placebo, and no evidence of a dose response relationship with Molnupiravir (see below). There was no apparent dose effect based on the incidence of death in each of the Molnupiravir groups. None of the deaths were considered related to trial intervention by the investigator, and most were associated with complications of COVID-19 or to secondary bacterial infections.

Virology data from the completed Phase 2 trial (MK-4482-006) in 204 non-hospitalised participants with COVID-19 have shown that treatment with Molnupiravir results in an antiviral effect, including reduction in viral load and in infectious virus as well as a higher percentage of

For peer review only

For peer review only - http://bmjopen.bmj.com/site/about/guidelines.xhtml
© Copyright: The University of Oxford and Oxford University Hospitals NHS Foundation Trust 2019

random mutations in viral RNA post treatment consistent with the mechanism of action (i.e., viral error catastrophe. (13)

Regarding disease progression, in the ongoing Phase 2/3 randomised, placebo-controlled, double-blind MK-4482-002 trial in non-hospitalised patients with COVID-19 (n=302), there was a consistent trend toward potential benefit from treatment with Molnupiravir early in the course of disease as well as in individuals with risk factors for severe illness from COVID-19. Interim analyses showed the following:

- Fewer participants in the combined Molnupiravir treatment groups (7/225, 3.1%) were hospitalised or died through Day 29 compared with participants in the placebo group (4/74, 5.4%) **
- While none of the comparisons reached statistical significance, the difference in the rate of death or hospitalisation favours Molnupiravir in all comparisons
- Most participants achieved sustained symptom improvement/resolution by Day 29, regardless of treatment received. However, confidence intervals were wide and did not provide clear evidence of treatment effect for time to progression or sustained improvement/resolution of COVID-19 signs and symptoms

*** A post-hoc analysis of the primary endpoint in the subgroup of participants who were randomised within 5 days of initial COVID-19 symptom onset and who had at least 1 risk factor for severe illness was also performed: 4/107 (3.7%) participants were hospitalised in the combined Molnupiravir groups compared with 4/34 (11.8%) participants in the placebo group representing an observed reduction in the relative risk for hospitalisation of 68%.*

A systematic review of early studies suggest benefit in terms of reduced hospital admissions.(16)

2. Detail of intervention

Participants randomised to the Usual Care plus Molnupiravir arm will receive Usual Care as per NHS guidelines, plus Molnupiravir for five days.

a. Precautions

No adverse drug reactions have been defined for Molnupiravir based on current data safety data from a Phase 1 trial (MK-4482-004) in 130 healthy participants who received single doses up to 1600 mg (including the food effect panel) and multiple doses up to 800 mg Q12H for 5.5 days indicate that Molnupiravir was generally well tolerated.(12) One participant discontinued from trial treatment because of a rash, appearing following 3 days of dosing with 800 mg Q12H Molnupiravir. This AE was rated as mild in intensity and considered by the investigator to be related to trial drug.

Safety data from Phase 2 studies show that all evaluated Molnupiravir doses were generally well tolerated in both hospitalised (MK-4482-001) and non-hospitalised (MK-4482-002) participants with COVID-19. No clinically meaningful differences in the incidence of AEs, SAEs, drug-related AEs, discontinuations due to AEs, and deaths were observed when comparing Molnupiravir to placebo, and no evidence of a dose response relationship with Molnupiravir.

For peer review only - http://bmjopen.bmj.com/site/about/guidelines.xhtml
© Copyright: The University of Oxford and Oxford University Hospitals NHS Foundation Trust 2019

There were no clinically meaningful trends for changes in liver enzymes or amylase and lipase as a function of either dose or treatment. Additionally, there were no meaningful abnormalities in haematological parameters as a function of either dose or treatment, and no evidence of changes relative to baseline in any haematological parameters over time in those treated with Molnupiravir compared with placebo through Day 29. Preliminary unblinded safety data from MK-4482-006 in non-hospitalised participants and blinded safety data from hospitalised participants in MK-4482-007 support the above safety conclusions. In MK-4482-001, there was a numerical imbalance in AEs resulting in death in hospitalised participants treated with Molnupiravir (14/218, 6.4%) compared with placebo (2/75, 2.7%). There was no apparent dose effect based on the incidence of death in each of the Molnupiravir groups. None of the deaths were considered related to trial intervention by the investigator, and most were associated with complications of COVID-19 or to secondary bacterial infections.

A dose-escalating, open-label, randomised-controlled (standard-of-care) Bayesian adaptive Phase I trial of adult outpatients with PCR-confirmed SARS-CoV-2 infection within 5 days of symptom onset randomised participants in 2:1 in groups of 6 participants to 300, 600 and 800mg doses of Molnupiravir orally, twice daily for 5 days or control. A dose was judged unsafe if the probability of 30% or greater dose-limiting toxicity (the primary outcome) over controls was 25% or greater. Secondary outcomes included safety, clinical progression, pharmacokinetics, and virological responses. Of 103 participants screened, 18 participants were enrolled between 17 July and 30 October 2020. Molnupiravir was well tolerated at 300, 600 and 800mg doses with no serious or severe AEs. Overall, 4 of 4 (100%), 4 of 4 (100%) and 1 of 4 (25%) of the participants receiving 300, 600 and 800mg Molnupiravir, respectively, and 5 of 6 (83%) controls, had at least one AE, all of which were mild (grade 2). The probability of 30% excess toxicity over controls at 800mg was estimated at 0.9%. They concluded that Molnupiravir was safe and well tolerated at a dose of 800mg twice daily for 5 days.(17)

b. *Pregnancy and lactation*

In the reproductive and developmental toxicity studies, there were no Molnupiravir-related effects on female or male fertility or early embryonic development up to the highest dose tested, 500 mg/kg/day (2.1/6.1-fold (female/male) the clinical NHC exposure at 800 mg Q12H). In pregnant rats dosed with Molnupiravir during the organogenesis period, developmental toxicity including embryo lethality (post implantation losses) and teratogenicity was observed at 1000 mg/kg/day (7.5-fold the clinical NHC exposure at 800 mg Q12H), and reduced fetal growth was noted at ≥500 mg/kg/day (2.9-fold the clinical NHC exposure at 800 mg Q12H).
There was no developmental toxicity at doses up to 250 mg/kg/day (0.8-fold the clinical NHC exposure at 800 mg Q12H). In pregnant rabbits, developmental toxicity was limited to reduced mean fetal body weights at 750 mg/kg/day (18-fold the clinical NHC exposure at 800 mg Q12H). There was no developmental toxicity in rabbits at up to 400 mg/kg/day (6.5-fold the clinical NHC exposure at 800 mg Q12H).

There are no human studies of its use among pregnant or lactating women.

Pregnancy (known or suspected) and breast-feeding are exclusions for the Molnupiravir arm of the trial based on the currently available data:

- Limited information on animal reproductive toxicity studies is provided in the SmPC
- There is evidence for the potential teratogenicity of Molnupiravir

For peer review only - http://bmjopen.bmj.com/site/about/guidelines.xhtml
© Copyright: The University of Oxford and Oxford University Hospitals NHS Foundation Trust 2019

- The effects of Molnupiravir on pregnant people are unknown

To mitigate the risk of pregnancy in the trial, all participants of child-bearing potential will be required to take a urine pregnancy test prior to commencing trial treatment. We will confirm a negative test result during the Day 1 or Day 2 telephone call with a member of the trial team (see section 2.8 of the master protocol for further information). Before starting the trial treatment, the clinician/research nurse will explain to the participant that pregnancy is an exclusion criterion and explain the contraception requirements during the trial. If the participant confirms that there is a possibility that they may be pregnant during this call, they will be excluded from taking part.

As per 'PC-CTU SOP TM119 Pharmacovigilance', any pregnancy that occurs during Molnupiravir (antiviral agent) administration requires monitoring and follow-up until the outcome of the pregnancy and any postnatal sequelae are known is known. The CI, PI or delegated individual will report any pregnancy occurring whilst in the trial to the PC-CTU. The Sponsor will report any pregnancy occurring whilst in the trial to the UK Teratology Information Service (UKTIS).

Participants themselves will be asked in their daily diaries or during the day 7, 14 and 28 phone calls, whether they have become pregnant since enrolling into the trial. These responses will be monitored daily and if a participant does become pregnant during the trial, the clinical team will inform them to immediately stop the medication. Consent to collect follow-up information regarding the outcome of the pregnancy and any postnatal sequelae in the infant will be sought from potential participants prior to trial entry. The CI or delegated individual will liaise with the relevant Obstetrician or equivalent HCP throughout the pregnancy until delivery to monitor for congenital abnormality or birth defect, at which the pregnancy would fall under the definition of serious and require reporting as an SAE.

The DSMC will be informed of any pregnancies in this treatment group, in weekly safety reports. Pregnancies and outcomes will be included in annual safety reports.

3. Trial visits

As per Master Protocol

4. Outcome measures

As per Master Protocol

5. Eligibility criteria (in addition to master protocol)

Inclusion criteria:

- Willingness to take a pregnancy test prior to starting Molnupiravir treatment (Participants of childbearing potential)

Exclusion criteria:

- Known or suspected pregnancy*
- Breastfeeding
    - Participants of childbearing potential (participants who are anatomically and physiologically capable of becoming pregnant), not willing to use effective

For peer review only - http://bmjopen.bmj.com/site/about/guidelines.xhtml
© Copyright: The University of Oxford and Oxford University Hospitals NHS Foundation Trust 2019

NUFFIELD DEPARTMENT OF PRIMARY CARE HEALTH SCIENCES Primary Care Clinical Trials Unit

PANORAMIC Platform Adaptive trial of NOvel antiviRals for eArly treatMent of COVID-19 In the Community

contraceptive** for 28-day duration of the trial, and who do not confirm a negative pregnancy test prior to starting the drug.

- Known allergy to Molnupiravir
- Currently taking Molnupiravir

* As recorded by the participant on the screening form and confirmed by interaction between clinician and participant, and the pregnancy test supplied by the trial.

** Effective methods include sterilisation, long-acting reversible contraceptive (LARC) methods (intrauterine devices and implants), combined hormonal methods (oral, transdermal, or intravaginal), or the progestogen only pill or injection. Participants will also be eligible if they have been abstinent for the 28 days before enrolling in the trial and will continue to be abstinent for the 28-day duration of follow-up where this is in line with the preferred and usual lifestyle of the subject.

Note: a barrier method on its own is not sufficient.

6. Professional role of those checking eligibility

To confirm that the participant meets the criteria defined above, information will be elicited through a direct discussion between a medically qualified professional , research nurse, nurse prescriber or a prescribing pharmacist, dependent on the ISA for the specific IMP involved and the participant. The participant can be randomised to Molnupiravir if any of these three categories of HCPs considers the potential participant is eligible.

7. Antiviral agent: Molnupiravir

a. Name

Lagevrio contains the active substance Molnupiravir. The drug will be referred to by the active substance only.

b. Dose

Molnupiravir 200 milligram (mg) capsules. The capsules are for oral administration. Four 200mg capsules (800mg) Molnupiravir to be taken every 12 hours (twice a day), for five days. This regimen was identified and found to be safe in a dose finding trial,(17) and has been used in a clinical trial in which early reports indicate was safe and efficacious.(18)

c. Common side effects

Common side effects, according to the SmPC, include dizziness, headache, diarrhoea, and nausea. These symptoms will be collected in daily diaries and calls on 7, 14 and 28 and will be monitored weekly by DSMC committee.

For peer review only - http://bmjopen.bmj.com/site/about/guidelines.xhtml
© Copyright: The University of Oxford and Oxford University Hospitals NHS Foundation Trust 2019

NUFFIELD DEPARTMENT OF PRIMARY CARE HEALTH SCIENCES Primary Care Clinical Trials Unit

PANORAMIC Platform Adaptive trial of NOvel antiviRals for eArly treatMent of COVID-19 In the Community

d. Concomitant medications

Molnupiravir has been found to lack inhibitory or inductive activity towards xenobiotic metabolic enzymes and transporters tested in vitro, suggesting that the potential for DDIs between Molnupiravir/NHC and co-medications is low.

e. Licensing Status

At the time of writing, the MHRA has approved the IMP for a Conditional Marketing Authorisation.

f. Manufacturer

Merck Sharp & Dohme (UK) Limited, Marketing Authorisation Number: PLGB 53095/0089.

g. Labelling and QP release

Vertical Pharma Resources Ltd (trading as IPS Pharma), 41 Central Avenue, West Molesey, KT8 2QZ, UK Authorisation number: WDA (H) 32879, will label and QP release the medication for trial purposes in accordance with Annex 13.

h. Storage

All trial medication is to be kept in a dry area, stored at 1° to 30°C (59° to 86°F). We will ask participants to store the medication at room temperature.

The medication will be stored at Vertical Pharma Resources Ltd in locked cupboards in restricted access rooms. It may also be stored securely with restricted access in the Nuffield Department of Primary Care Health Sciences; in GP Practices; in Pharmacies.

i. Distribution

Molnupiravir will be labelled and QP released by an accredited licensed central facility: Vertical Pharma Resources Ltd. Vertical Pharma Resources Ltd will prepare and dispatch the participant pack containing IMP, directly to the participant at home, in accordance with their SOPs. The labelled and QP released Molnupiravir can also be held by the PC-CTU and trial Hubs, from where it may also be issued to participants.

j. Drug accountability

No additional mechanisms for drug accountability are required beyond those outlined in the master protocol.

k. Drug destruction/returns

Participants will be asked to return unused Molnupiravir to the PC-CTU via pre-paid courier, which will be documented in accountability logs. After a final reconciliation of drug accountability records and authorisation by the sponsor or delegate, unused trial medication at the PC-CTU and Vertical Pharma Resources Ltd will be disposed of in line with local SOPs. Unused trial medication may be destroyed by an authorised third party.

l. Overdose

There is no human experience of overdosage with Molnupiravir. Treatment of overdose with Molnupiravir should consist of general supportive measures including the monitoring of the clinical status of the patient. Haemodialysis is not expected to result in effective elimination of NHC. (SmPC, section 4.9). In line with the SmPC we will monitor potential overdoses by asking in the daily diary and Day 7,14 and 28 call CRF whether the participant has taken more than the

For peer review only - http://bmjopen.bmj.com/site/about/guidelines.xhtml
© Copyright: The University of Oxford and Oxford University Hospitals NHS Foundation Trust 2019

specified dose. A safety alert will be triggered if the participant records that they have exceeded the dose
A doctor from the central clinical team will contact the participant immediately and then follow-up accordingly (at clinical discretion) to monitor any potential AEs caused by the overdose. This may include no further action or repeated contact depending on the nature and severity of symptoms.

8. Safety reporting

a. Adverse effects

Pregnancy will be recorded as an AE of Special Interest.
Reporting period: Occurring within 28-day following first administration of the IMP as requested by the MHRA. Such events discovered after 28-day time point, will also be reported.

b. Reference Safety Information (RSI)

See section 4.8 of the SmPC, Merck Sharp and Dohme (UK) Limited, 05 Nov 2021.

c. Risk/benefit assessment

The UK Antivirals Taskforce (AT) established by the Department of Health and Social Care recommends including Molnupiravir into the PANORAMIC platform with an 800mg twice a day, for five days**,** based on a review of efficacy and safety data.

i. Risks

In the available six clinical studies in participants with COVID-19 (n=922 with COVID-19 receiving placebo or Molnupiravir as multiple doses up to 800 mg for 5 days), Molnupiravir was well-tolerated, with no clinically meaningful trends were observed for changes in clinical laboratory values as a function of dose or treatment.

In one phase 1 randomised, double-blind, placebo-controlled SAD/MAD trial (single ascending dose/multiple ascending dose) in 130 healthy adult male and female participants, receiving placebo or Molnupiravir in single doses of 50 to 1600 mg or in multiple doses of 50 to 800 mg twice daily for 5.5 days, overall, found no clinically meaningful trends for changes in clinical laboratory values, vital signs, or ECGs as a function of dose or treatment.(12) No clinically meaningful haematological laboratory test result abnormalities were observed. Transient elevations in serum lipase of ≥3-times the ULN were observed ≥3 days after the last dose of trial drug in a low and similar proportion of Molnupiravir and placebo recipients and were not associated with clinical symptoms of pancreatitis.

In a Phase 2 trial randomised, placebo-controlled, double-blind trial in hospitalised patients with COVID-19, there was an imbalance in mortality rates in patients treated with Molnupiravir (14/218, 6.4%) compared with placebo (2/75, 2.7%). None of the deaths were considered related to trial intervention by the investigators, and most were associated with complications of COVID-19 or to secondary bacterial infections.

Taking this evidence into account, participation requires participants to agree to use adequate contraception for the duration of the treatment and 28 days of follow-up.

For peer review only - http://bmjopen.bmj.com/site/about/guidelines.xhtml
© Copyright: The University of Oxford and Oxford University Hospitals NHS Foundation Trust 2019

ii. Benefits

Molnupiravir may reduce SARS-CoV-2 viral loads, COVID-19 symptoms, risk of onward transmission, and severity of disease.

Virology data from clinical studies (Part 1 of MK-4482-001 in hospitalised patients and MK-4482-002 in non-hospitalised patients) show that treatment with Molnupiravir reduces the SARS-CoV-2 VL compared with placebo (based on change from baseline, slope of decline, and greater proportion of participants with a VL below the LOQ at early time points) in non-hospitalised participants enrolled in MK-4482-002 and participants who had COVID-19 symptom onset ≤5 days prior to randomisation in both MK-4482-001 and MK-4482-002. In addition, consistent with the proposed mechanism of action of Molnupiravir of viral error catastrophe, the highest percentage of mutations in viral RNA post-treatment at Day 5 were observed in the 800 mg Q12H intervention group in MK-4482-001 and MK-4482-002.

In hospitalised participants (MK-4482-001), the observed rate of sustained recovery through 29 days was low for all studied doses of Molnupiravir as compared with placebo. While no clear dose effect was observed across Molnupiravir doses studied, there were a higher number of deaths through Day 29 in participants who received Molnupiravir compared with placebo. None of the deaths were assessed as related to trial intervention.

In non-hospitalised participants (MK-4482-002) evaluation of the primary clinical efficacy endpoint showed that 11 of 299 participants were hospitalised through Day 29 (; ~3% of participants in the Molnupiravir intervention groups were hospitalised or died through Day 29 (compared with ~5% in the placebo group). All hospitalised participants had at least 1 risk factor for severe illness from COVID. Protocol-specified subgroup analyses for the primary endpoint indicated potential clinical benefit from treatment with Molnupiravir early in the course of disease (i.e., symptom onset ≤5 days prior to the day of randomisation) as well as in individuals with risk factors for progression to severe illness from COVID-19, including age >60 years.

For peer review only

For peer review only - http://bmjopen.bmj.com/site/about/guidelines.xhtml
© Copyright: The University of Oxford and Oxford University Hospitals NHS Foundation Trust 2019

NUFFIELD DEPARTMENT OF PRIMARY CARE HEALTH SCIENCES Primary Care Clinical Trials Unit

PANORAMIC Platform Adaptive trial of NOvel antiviRals for eArly treatMent of COVID-19 In the Community

d. Risk Assessment: Oral Molnupiravir Four 200mg capsules (800mg) Molnupiravir, twice a day, for five days.

Hazard	Likelihood (L, M, H)	Impact (L, M, H)	Mitigation	Monitoring
1. Pregnancy:<br>i. Potential teratogenicity.<br>ii. There are no human studies of use among pregnant or lactating people.	H	H	Requirement for negative pregnancy test in participants of child-bearing potential, prior to starting medication.<br><br>We will exclude known pregnancy, breastfeeding, and require participants to use adequate contraception for the duration of the treatment and 28 days of follow-up.<br><br>During the pre-randomisation call, the clinician/research nurse will confirm this exclusion criteria with the participant.	Confirmation of negative pregnancy test documented in the Day 1 and/or Day 2 Call CRFs and Daily Diary.<br><br>We will monitor daily responses to the question 'have you become pregnant since starting the trial?' and follow-up as required to immediately stop treatment, if applicable.<br><br>Pregnancy occurring during the 28-day trial follow-up period will be reported as an AE of Special Interest.<br><br>As per 'PC-CTU SOP TM119 Pharmacovigilance', any pregnancy that occurs during IMP administration requires monitoring and follow-up until the outcome of the pregnancy is known. The CI or delegated individual will liaise with the relevant Obstetrician throughout the pregnancy.<br><br>The DSMC will be informed of any pregnancies in this treatment group, in weekly safety reports.

For peer review only

For peer review only - http://bmjopen.bmj.com/site/about/guidelines.xhtml
© Copyright: The University of Oxford and Oxford University Hospitals NHS Foundation Trust 2019

NUFFIELD DEPARTMENT OF PRIMARY CARE HEALTH SCIENCES Primary Care Clinical Trials Unit PANORAMIC Platform Adaptive trial of NOvel antiviRals for eArly treatMent of COVID-19 In the Community

2. Unknown/other potential side-effects.	M	M	All participants will receive a call on day 1 to make sure that they understand the possible risks associated with Molnupiravir and how to report potential side-effects and seek medical care if required.<br><br>Participants will be provided with a 24-hour contact telephone line to report any AEs that they experience and are concerned about, directly to a clinician.<br><br>We will collect symptoms and side effects from symptom diaries and participant telephone calls.	The DSMC will review weekly reports of unblinded symptom data to identify potential side-effects of Molnupiravir.<br><br>Any safety signals will be communicated to the TSC and TMG as defined in the DSMC Charter.<br><br>The ESG will review accumulating safety data in the Molnupiravir arm including AEs, SAEs and laboratory results as defined in the ISA and ESG Charter.<br><br>TMG will review the total number of SAEs as per TMG Charter.

For peer review only - http://bmjopen.bmj.com/site/about/guidelines.xhtml
© Copyright: The University of Oxford and Oxford University Hospitals NHS Foundation Trust 2019

3. Compliance			Participants will be asked in their daily diaries about trial medication use.	The trial team will monitor daily diary responses where the participant indicates that they have taken too much IMP and escalate to the clinical team to follow-up with the participant.

For peer review only

For peer review only - http://bmjopen.bmj.com/site/about/guidelines.xhtml
© Copyright: The University of Oxford and Oxford University Hospitals NHS Foundation Trust 2019

NUFFIELD DEPARTMENT OF PRIMARY CARE HEALTH SCIENCES Primary Care Clinical Trials Unit

PANORAMIC Platform Adaptive trial of NOvel antiviRals for eArly treatMent of COVID-19 In the Community

3 USUAL CARE PLUS PAXLOVID

1. Background

a. Potential mechanism of efficacy

Paxlovid consists of nirmatrelvir [PF-07321332] tablets and ritonavir tablets. Nirmatrelvir is an oral antiviral that has been developed specifically for treatment of COVID-19. (19) It is a protease inhibitor and inhibits the SARS-CoV-2 3CL protease, thereby preventing viral replication. (19) Ritonavir inhibits CYP3A-mediated metabolism of nirmatrelvir, and therefore increases plasma concentrations of nirmatrelvir to therapeutic levels.

b. Evidence for potential benefits of Paxlovid in COVID-19 illness

In vitro antiviral activity

In vitro studies have demonstrated that PF-07321332 is a potent inhibitor of SARS-CoV-2 3CL protease in a biochemical enzymatic assay (Ki = 3.11 nM) and in epithelial Vero E6 cells (EC50 = 74.5 nM). (19) PF-07321332 also exhibited antiviral activity against SARS-CoV-2 infection of differentiated normal human bronchial epithelial (dNHBE) cells (EC_{90} value of 181 nM) and human adenocarcinoma–derived alveolar basal epithelial cells expressing ACE2 (A549-ACE2 cells, EC_{90} value 215 nM). (19)

In vivo antiviral activity

PF-07321332 showed antiviral activity in mouse models with mouse-adapted SARS-CoV-2 infection in BALB/c and 129 mouse strains. Oral administration of PF-07321332 at 300 mg/kg or 1,000 mg/kg twice daily initiated 4 hours post-inoculation or 1,000 mg/kg twice daily initiated 12 hours post inoculation with SARS-CoV-2 MA10 resulted in reduction of lung viral titres and ameliorated indicators of disease (weight loss and lung pathology) compared to placebo-treated animals. (19)

Phase 1 studies

In a phase 1, randomised placebo-controlled trial of 70 healthy adult participants, (20) PF-07321332 was administered alone or with ritonavir in ascending doses. PF-07321332 was well tolerated and safe, and plasma concentrations were boosted when co-administered with ritonavir. (19) With a PF-07321332 dose of 250 mg, and 100mg of RTV at -12, 0 and 12 hours, plasma PF-07321332 concentrations after 12 hours were considerably above the SARS-CoV-2 antiviral EC_{90} value (total EC_{90} = 292 ng/ml, unbound EC_{90} = 90.5 ng/ml, 181 nM).

Phase 2/3 studies

The efficacy of Paxlovid to treat COVID-19 has been assessed in the Phase 2/3 Evaluation of Protease Inhibition for COVID-19 in High-Risk patients (EPIC-HR) trial. 2,246 non-hospitalized, high-risk adult patients with COVID-19 and symptom onset ≤5 days were randomised 1:1 to receive Paxlovid 300mg/100mg or placebo every 12 hours for 5 days. Eligible participants had at least one risk factor for severe COVID-19 and must not have been vaccinated or previously had COVID-19. Among those who received treatment within 3 days, 5/697 (0.7%) in the Paxlovid group met the primary endpoint of 28-day all-cause hospitalisation or death, compared to 44/682 (6.5%)

For peer review only - http://bmjopen.bmj.com/site/about/guidelines.xhtml
© Copyright: The University of Oxford and Oxford University Hospitals NHS Foundation Trust 2019

in the placebo group (relative risk reduction 89%). (21) (22) There were no deaths in the Paxlovid group and 9 deaths in the placebo group. In a secondary analysis among those treated within 5 days of symptom onset, 8/1039 (0.8%) in the Paxlovid group were hospitalised or died, versus 66/1046 (6.3%) in the placebo group (relative risk reduction 88%). Among 1574 participants who had SARS-CoV-2 viral load measured at Days 0 and 5, Day 5 viral loads were approximately 10-fold lower in the Paxlovid group versus placebo, after adjusting for baseline viral load, geographic region, and serology status. (21) Regarding safety, 23% of participants in the Paxlovid group experienced AEs, versus 24% in the placebo group. SAEs occurred in 1.6% of Paxlovid group versus 6.6% of placebo group participants. Dysgeusia (6% and <1%, respectively), diarrhoea (3% and 2%), and vomiting (1% and <1%) were the AEs (all grades regardless of causality) that occurred more frequently in the Paxlovid group (≥ 1%) than the placebo group respectively. (21)

The Evaluation of Protease Inhibition for COVID-19 in Standard-Risk Patients (EPIC-SR) Phase 2/3 trial, is assessing efficacy of Paxlovid among unvaccinated adults who were at standard risk (i.e., low risk of hospitalization or death) as well as vaccinated adults who had one or more risk factors for progressing to severe illness.(23) In an interim analysis, there was no evidence of superiority in the primary outcome of self-reported sustained recovery for 4 consecutive days.(21) The secondary outcome of hospitalisations and deaths was 70% lower in the Paxlovid group (3/428, 0.7%) versus placebo (10/426, 2.4%, p 0.051), and viral loads were approximately 10 times lower in the Paxlovid group. There were 22% versus 21% AEs, 1.4% versus 1.9% SAEs, and 2.1% versus 1.2% discontinuations of trial drug due to AEs in the Paxlovid versus placebo arms respectively. (21)

2. Detail of intervention

Participants randomised to the usual care plus Paxlovid arm will receive usual clinical care as per NHS guidelines, plus Paxlovid for five days. Nirmatrelvir must be given with ritonavir to achieve therapeutic concentrations. The usual recommended dosage is 300 mg PF 07321332 (two 150 mg tablets) with 100 mg ritonavir (one 100 mg tablet) all taken together orally twice daily for 5 days.

a. Precautions

Potential SARs due to drug-drug interactions

Paxlovid contains ritonavir. Ritonavir is an inhibitor, inducer, and substrate of various drug-metabolizing enzymes and/or drug transporters. Most notably, as a strong inhibitor of CYP3A, it may increase concentrations of certain concomitant medications, thereby increasing the potential for significant drug toxicities. CYP3A inhibition by ritonavir typically resolves 3 to 5 days after the drug is discontinued. When ritonavir is used for a treatment duration of 5 days, its induction properties are less likely to be clinically relevant than when the drug is used chronically for HIV. See section Appendix F for full lists of contraindicated concomitant medications and concomitant medications that may be taken with caution.

Medications that induce or inhibit CYP3A may also reduce or increase Paxlovid levels. Induction of 3A4 may result in sub-therapeutic Paxlovid levels, increasing the risk of development of viral

For peer review only - http://bmjopen.bmj.com/site/about/guidelines.xhtml
© Copyright: The University of Oxford and Oxford University Hospitals NHS Foundation Trust 2019

resistance. Increased inhibition of 3A4 may increase the risk of significant adverse reactions from increased levels of Paxlovid.

Hepatotoxicity

Increased hepatic transaminases, hepatitis and jaundice have occurred in patients receiving ritonavir. Patients with known severe liver disease will not be eligible to be randomised to Paxlovid.

Excipients
PF-07321332 tablets contain lactose. Patients with rare hereditary problems of galactose intolerance, total lactase deficiency or glucose-galactose malabsorption should not take Paxlovid.

b. Fertility, pregnancy, and lactation

Fertility
There are no human data on the effects of Paxlovid on fertility. In rats there was no evidence of effects of PF-07321332 on fertility or early embryonic development at doses up to 1000mg/kg/day, representing 12x/4.3x based on the predicted human Cmax/AUC24 at a twice-daily dose of 300 mg/100 mg PF-07321332/ritonavir.

Pregnancy

There is no human data on the effects of Paxlovid on pregnancy.

In studies of the effects of PF-07321332 on embryo-foetal development in rats and rabbits at doses of up to 1000mg/day, there was no evidence of PF-07321332 related effects in the rat model at any of the doses studied. In the rabbit model, foetal morphology and viability were not affected at any dose, however lower foetal body weights were noted with the highest dose of PF-07321332 1000mg/kg/day, along with slight decreases in maternal body weight and food consumption.

In rat and rabbit studies, ritonavir was associated with early resorptions, decreased foetal weight, ossification delays, decreased litter sizes and developmental variations, but only at dose levels that caused maternal toxicity. In humans, over 6100 live births have been reported to be exposed to ritonavir during pregnancy, of which 2800 were during the first trimester, with no increase in birth defects compared to rates seen in the population base birth defect surveillance system.

As the effect of Paxlovid on pregnancy in humans is unknown, pregnant women will be excluded and pregnancy will be reported as an AE of special interest.

As per 'PC-CTU SOP TM119 Pharmacovigilance', any pregnancy that occurs during Paxlovid (antiviral agent) administration requires monitoring and follow-up until the outcome of the pregnancy and any postnatal sequelae are known. The CI, PI or delegated individual will report any pregnancy occurring whilst in the trial to the PC-CTU. The Sponsor will report any pregnancy occurring whilst in the trial to UKTIS.

Breast-feeding

For peer review only - http://bmjopen.bmj.com/site/about/guidelines.xhtml
© Copyright: The University of Oxford and Oxford University Hospitals NHS Foundation Trust 2019

There are no human data on the effects of Paxlovid in breast-feeding, and it is not known whether PF-07321332 is excreted in human breast milk. Ritonavir is excreted in breast milk but the effect on breast milk production and on the new-born, infant is not known.

3. Trial visits

As per Master Protocol with the addition of extra safety calls on day 4 and day 10 to participants randomised to the Paxlovid arm only. The purpose of the day 4 safety call is to detect any early side-effects of Paxlovid and to enable the investigator to suggest changes to participants medication including stopping where required. The day 10 safety call is to allow the side-effect profile of Paxlovid to be compared against the SmPC for Paxlovid rather than the Usual Care arm.

4. Outcome measures

As per Master Protocol

5. Eligibility criteria (in addition to master protocol)

Inclusion criteria:

- Willing to take a pregnancy test after randomisation and prior to starting Paxlovid treatment (Participants of childbearing potential)
- Patients with known mild kidney disease (CKD) stage 2, must have an eGFR measurement taken in the past 6 months

Exclusion criteria:

- Known or suspected pregnancy*
- Breastfeeding*
- Participants of childbearing potential (participants who are anatomically and physiologically capable of becoming pregnant) who do not confirm a negative pregnancy test prior to starting the drug, and who are not willing to use one of the contraceptive methods for the durations defined below:
    - sterilisation, long-acting reversible contraceptive (LARC) methods (intrauterine devices, intrauterine systems, and implants), or the progestogen only pill or injection, for the 28-day duration of follow-up in the trial
    - combined hormonal contraception (oral, transdermal, or intravaginal) alongside **an additional barrier method** (e.g., male condom) for the duration of Paxlovid treatment, and until after one complete menstrual cycle after stopping Paxlovid
    - abstinence: being abstinent for the 28 days before enrolling in the trial and will continue to be abstinent for the 28-day duration of follow-up where this is in line with the preferred and usual lifestyle of the subject
    - Note: a barrier method on its own is **not** sufficient
- History of clinically significant hypersensitivity to the active substances in Paxlovid (PF-07321332/ritonavir) or to any of its excipients
- Patients with known rare hereditary problems of galactose intolerance, total lactase deficiency or glucose-galactose malabsorption

For peer review only - http://bmjopen.bmj.com/site/about/guidelines.xhtml
© Copyright: The University of Oxford and Oxford University Hospitals NHS Foundation Trust 2019

- Patients with known current severe liver impairment (characterised by severe ascites, encephalopathy, jaundice, or prolonged INR. People with liver disease **without** any of these features are eligible)
- Patients with known moderate or severe renal disease (defined as CKD stage 3, 4 or 5 or current acute kidney injury or most recent eGFR in the past 6 months <60 ml/min)
- Currently taking Paxlovid
- Clinical requirement to continue taking a drug which is contraindicated or not recommended for administration with Paxlovid in in the context of PANORAMIC (Appendix G) or is taking a drug which in the opinion of the investigator would put the subject at unacceptable risk

* As recorded by the participant on the screening form and confirmed by interaction between clinician and participant, and the pregnancy test supplied by the trial.

6. Professional role of those checking eligibility

To confirm that the participant meets the criteria defined above, information will be elicited through a direct discussion between the participant and a medically qualified professional , a prescribing pharmacist or a nurse prescriber (as required by standard prescribing practices at Covid Medicines Delivery Units across all four Administrations within the UK). Those assessing eligibility must take a relevant drug history and have access to a version of a summary care record in use in any Devolved Administration, and may, if necessary according to their clinical judgement, access and review further information contained within secondary care records or full primary care records.
If after reviewing relevant medical records and discussion with the patient, the recruiting health care professional considers the potential participant is eligible, they may then be randomised to Paxlovid.

7. Antiviral agent: Paxlovid

a. Name

Paxlovid is the brand name for two active substances nirmatrelvir (PF07321332) plus ritonavir. The drug will be referred to by brand name only.

b. Dose

Nirmatrelvir [PF-07321332] 150 mg tablets and ritonavir 100mg tablets. The tablets are for oral administration. Two 150 mg tablets (300mg) nirmatrelvir and one 100mg tablet (100 mg) ritonavir all taken together orally twice daily for 5 days.

If the patient misses a dose of Paxlovid within 8 hours of the time it is usually taken, the patient should take it as soon as possible and resume the normal dosing schedule. If the patient misses a dose by more than 8 hours, the patient should not take the missed dose and instead take the next

For peer review only - http://bmjopen.bmj.com/site/about/guidelines.xhtml
© Copyright: The University of Oxford and Oxford University Hospitals NHS Foundation Trust 2019

dose at the regularly scheduled time. The patient should not double the dose to make up for a missed dose.

If a patient requires hospitalization due to severe or critical COVID-19 after starting treatment with Paxlovid, the patient should complete the full 5-day treatment course at the discretion of his/her healthcare provider.

Paxlovid can be taken with or without food. The tablets should be swallowed whole and not chewed, broken, or crushed.

Renal failure

No dose adjustment is necessary for patients with mild renal impairment (eGFR ≥60 ml/min, CKD stage 1-2). Patients with moderate renal impairment (eGFR ≥30 to <60 mL/min, CKD stage 3) will not be eligible for randomisation to Paxlovid, as the dose of Paxlovid should be reduced to PF-07321332/ritonavir 150 mg/100 mg (1 tablet of each) twice daily for 5 days, and this is not feasible in this large scale, pragmatic trial. Patients with severe renal impairment (eGFR <30 ml/min, CKD stage 4-5) are not recommended to have Paxlovid and are also not eligible for randomisation to the Paxlovid arm.

Hepatic impairment

No dose adjustment is required for patients with mild to moderate hepatic impairment. Patients with severe hepatic impairment are not recommended for Paxlovid and are not eligible for randomisation to the Paxlovid arm.

Concomitant therapy with ritonavir- or cobicistat-containing regimen

No dose adjustment is needed; the dose of Paxlovid is 300 mg/100 mg twice daily for 5 days.
Patients diagnosed with human immunodeficiency virus (HIV) or hepatitis C virus (HCV) infection who are receiving ritonavir- or cobicistat-containing regimen should continue their treatment as indicated.

c. Common side effects

Common side effects include dysgeusia (disturbances of taste), diarrhoea and vomiting. (22)

d. Concomitant medications

Medications that may interact with Paxlovid, and the implications for eligibility for PANORAMIC, are listed in Appendix G. This list is based on the summary of product characteristics and will be updated as new information becomes available. Clinical judgement is required to evaluate potential drug interactions. Detailed advice is also available from the Liverpool COVID-19 Drug Interactions Checker website: https://www.covid19- druginteractions.org/. Patients who are taking Paxlovid as part of the trial will be advised that they must check with a clinician before initiating any new medications while taking Paxlovid to ensure that the potential for drug-drug interaction has been considered. Such participants with also be provided with a drug interaction

For peer review only - http://bmjopen.bmj.com/site/about/guidelines.xhtml
© Copyright: The University of Oxford and Oxford University Hospitals NHS Foundation Trust 2019

warning card with advice for their clinician, and their clinician will be able to seek advice from the trials clinical team.

e. Licensing status

At the time of writing, the MHRA has issued a Conditional Marketing Authorisation for Paxlovid in Great Britain and a temporary Regulation 174 authorisation for Northern Ireland.

f. Manufacturer

Pfizer Limited, Ramsgate Rd, Sandwich, Kent, CT13 9NJ, UK.
Marketing Authorisation Number: PLGB 00057/1710

g. Labelling and QP release

Vertical Pharma Resources Ltd (trading as IPS Pharma), 41 Central Avenue, West Molesey, KT8 2QZ, UK Authorisation number: WDA (H) 32879, will label and QP release the medication for trial purposes in accordance with Annex 13.

h. Storage

All trial medication is to be kept in a dry area, stored at 1° to 30°C (59° to 86°F). We will ask participants to store the medication at room temperature and not to refrigerate or freeze.

The medication will be stored at Vertical Pharma Resources Ltd in locked cupboards in restricted access rooms. It may also be stored securely with restricted access in the Nuffield Department of Primary Care Health Sciences; in GP Practices; in Pharmacies.

i. Distribution

Paxlovid will be labelled and QP released by an accredited licensed central facility: Vertical Pharma Resources Ltd. Vertical Pharma Resources Ltd will prepare and dispatch the participant pack containing IMP, directly to the participant at home, in accordance with their SOPs. The labelled and QP released Paxlovid can also be held by the PC-CTU and trial Hubs, from where it may also be issued to participants.

j. Drug accountability

No additional mechanisms for drug accountability are required beyond those outlined in the master protocol.

k. Drug destruction/returns

Participants will be asked to return unused Paxlovid to the PC-CTU via pre-paid courier, which will be documented in accountability logs. After a final reconciliation of drug accountability records and authorisation by the sponsor or delegate, unused trial medication at the PC-CTU and Vertical Pharma Resources Ltd will be disposed of in line with local SOPs. Unused trial medication may be destroyed by an authorised third party.

For peer review only - http://bmjopen.bmj.com/site/about/guidelines.xhtml
© Copyright: The University of Oxford and Oxford University Hospitals NHS Foundation Trust 2019

l. Overdose

There is no human experience of overdosage with nirmatrelvir and limited human experience of acute overdose with ritonavir. One patient in clinical trials took ritonavir 1500 mg/day for two days and reported paraesthesia, which resolved after the dose was decreased, and a case of renal failure with eosinophilia after ritonavir overdose has been reported. (24)

The signs of ritonavir toxicity observed in animals (mice and rats) included decreased activity, ataxia, dyspnoea, and tremors.

Treatment of overdose with Paxlovid should consist of general supportive measures including the monitoring of the clinical status of the patient. In line with the SmPC we will monitor potential overdoses by asking in the daily diary and in the D2 and D4 call CRF whether the participant has taken more than the specified dose. A safety alert will be triggered if the participant records that they have exceeded the dose. A doctor from the central clinical team will contact the participant immediately and then follow-up accordingly (at clinical discretion) to monitor any potential AEs caused by the overdose. This may include no further action or repeated contact depending on the nature and severity of symptoms.

8. Safety reporting

a. Adverse effects

All non-COVID-19 events (at the discretion of the reporting nurse/clinician) reported during the safety and follow up calls and recorded in the daily symptom diaries will be recorded as AEs in the first instance.
Pregnancy will be recorded as an AE of Special Interest.
Reporting period: Occurring within 28-day following first administration of the IMP as requested by the MHRA. Such events discovered after 28-day time point, will also be reported.

b. Reference Safety Information
See section 4.8 of the SmPC, Pfizer (UK) Limited, 02-Mar-2022.

c. Risk/benefit assessment

The UK AT established by the Department of Health and Social Care recommends including Paxlovid into the PANORAMIC platform with a dose of 300/100mg twice a day, for five days, based on a review of efficacy and safety data.

i. Risks

For peer review only - http://bmjopen.bmj.com/site/about/guidelines.xhtml
© Copyright: The University of Oxford and Oxford University Hospitals NHS Foundation Trust 2019

Adverse events

In the EPIC-HR trial, among 2,224 symptomatic unvaccinated adults age ≥18 years of age and at high risk of developing severe COVID-19 illness, n=1,109 received at least one dose of Paxlovid and n=1,115 received placebo. 23% versus 24% experienced AEs, and 1.6% versus 6.6% experienced SAEs (including COVID-19 related AEs), in the Paxlovid group versus placebo group respectively.(3) AEs (all grades regardless of causality) in the Paxlovid group (≥1%) that occurred at a greater frequency (≥5 subject difference) than in the placebo group were dysgeusia (6% and <1%, respectively), diarrhoea (3% and 2%), hypertension (1% and <1%), and myalgia (1% and <1%). 2% of participants in the Paxlovid group and 4% in the placebo group discontinued treatment due to an AE. (22)

In an interim analysis of the EPIC-SR trial among standard risk patients (i.e., unvaccinated with no risk factors for severe disease or vaccinated with a risk factor for severe disease), AEs (22% versus 21%), SAEs(1.4% vs 1.9%) and discontinuation of trial drug due to AEs (2.1% vs. 1.2%) were comparable between Paxlovid (22%) and placebo (21%). (21)

Hepatic transaminase elevations, clinical hepatitis and jaundice have occurred in patients receiving ritonavir. Therefore, caution should be exercised when administering Paxlovid to patients with pre-existing liver diseases, liver enzyme abnormalities or hepatitis.

Risk of drug interactions

CYP3A related drug interactions listed in Appendix G could lead to clinically significant adverse reactions, including severe, life threatening or fatal events, due to increased levels of concomitant medications, or increased levels of Paxlovid. Medications that induce CYP3A may also reduce Paxlovid levels, leading to sub-therapeutic Paxlovid levels and the risk of development of viral resistance. This may occur if Paxlovid is initiated in patients receiving CYP3A metabolised medications, or if CYP3A metabolised medications are initiated among patients receiving Paxlovid.

Risk of pregnancy in participants receiving combined oral contraceptives

Ritonavir may reduce ethinyl estradiol concentrations and reduce the efficacy of combined oral contraceptive methods. This is unlikely to impair contraceptive efficacy, particularly considering the short duration of nirmatrelvir/ritonavir treatment, though it may increase the risk of irregular bleeding. (25) We will advise participants of childbearing potential who are using combined hormonal contraception (oral, transdermal, or intravaginal) to use an additional barrier method of contraception during treatment with Paxlovid, and until one menstrual cycle is completed after the last dose of Paxlovid.

Risks in pregnancy and during breastfeeding

There is no human data on the effect of Paxlovid on pregnancy or in breastfeeding. The summary of product characteristics states that breast-feeding should be discontinued during treatment with Paxlovid and for 7 days after the last dose of Paxlovid. Therefore, to be eligible for

For peer review only

For peer review only - http://bmjopen.bmj.com/site/about/guidelines.xhtml
© Copyright: The University of Oxford and Oxford University Hospitals NHS Foundation Trust 2019

randomisation to Paxlovid, participants are required to use a highly effective method of contraception for the duration of the treatment and 28 days of follow-up. Pregnant and breastfeeding participants will not be eligible.

Antiretroviral resistance

In individuals with HIV-1 viraemia (either undiagnosed or diagnosed but not controlled), the low dose ritonavir in Paxlovid may lead to a risk of HIV-1 developing resistance to HIV protease inhibitors. However, due to the short duration of ritonavir exposure, and the high genetic barrier to HIV-1 drug resistance with HIV protease inhibitors, this risk is thought to be low.

ii. Benefits

Paxlovid may reduce SARS-CoV-2 viral loads and severity of disease.

In the Phase 2/3 EPIC-HR trial among 2246 non-hospitalized high-risk adults with laboratory confirmed SARS-CoV-2 infection and with symptom onset ≤5 days, hospital admissions and deaths were 88% lower in the Paxlovid group compared to placebo. Day 5 nasopharyngeal viral load levels were approximately 9-fold lower in the Paxlovid group versus placebo. (21) (23)

In an interim analysis of the Phase 2/3 EPIC-SR trial among non-hospitalized **standard-risk** adults with laboratory confirmed SARS-CoV-2 infection and with symptom onset ≤5 days
EPIC-SR, there was no difference in self-reported alleviation of all symptoms, but hospitalisations were 70% lower in the Paxlovid group versus placebo. Viral loads were also 10-fold lower in the Paxlovid group. (21)

d. Risk Assessment: Oral Paxlovid: two 150 mg tablets (300mg) nirmatrelvir and one 100mg tablet (100 mg) ritonavir all taken together orally twice daily for 5 days.

**Hazard**	**Likelihood (L, M, H)**	**Impact (L, M, H)**	**Mitigation**	**Monitoring**
1. Risk of drug interactions	H	H	We will exclude patients currently taking contra-indicated concomitant medication. Patients will be asked to confirm they are not taking contraindicated medication as part of the screening process.<br><br>Participants who report taking concomitant medication will be assessed for eligibility by a medically trained professional with	The DSMC will review weekly reports of unblinded symptom data to identify potential AEs caused by drug interactions with Paxlovid. Any safety signals will be communicated to the TSC and TMG as defined in the DSMC Charter. The ESG will review accumulating safety data in the

For peer review only

For peer review only - http://bmjopen.bmj.com/site/about/guidelines.xhtml
© Copyright: The University of Oxford and Oxford University Hospitals NHS Foundation Trust 2019

			access to a summary care medical record in use in any Devolved Administration in the UK and additional medical records if considered necessary. Participants who are on drugs that do not lead to exclusion (per Appendix G) but have specific recommendations for monitoring will be flagged on the Spinnaker data collection system.<br><br>Participants in the Paxlovid arm have enhanced safety follow up calls on days 2, 4 and 10. Participants who are flagged in the system will be asked about clinically significant drug interactions using the standard scripts (per Appendix F) on days 2 and 4. These include specific actions in the event of elicitation of AEs.<br><br>The importance of the participant informing their recruiting clinician or the safety line clinician, and completing the new medication CRF to alert the central safety team will be emphasised during the day 0, day 2 and day 4 calls.<br><br>Participants for whom we have no diary data will be asked additional questions	Paxlovid arm including AEs, SAEs and laboratory results as defined in the ISA and ESG Charter.

For peer review only - http://bmjopen.bmj.com/site/about/guidelines.xhtml
© Copyright: The University of Oxford and Oxford University Hospitals NHS Foundation Trust 2019

			regarding whether any new medications have been started during their day 7 diary follow-up call.<br><br>In addition, all participants will be asked in their daily diary (up to day 8) if they have started any new medications.<br><br>Completion of the new medication CRF will trigger a safety alert to the central safety team to follow-up participants.<br><br>Participants randomised to Paxlovid will be provided with an emergency wallet card stating that they are participating in the PANORAMIC trial and have been assigned Paxlovid. Their clinician will also be able to discuss any medication related queries with the trial clinical team.	
2. Pregnancy: There are no human studies of use among pregnant or lactating people.	H	H	Requirement for negative pregnancy test in participants of child-bearing potential, prior to starting medication. We will exclude known pregnancy, breastfeeding, and require participants to use effective contraception for the duration of the treatment and 28 days of follow-up. During the pre-randomisation call, the	Confirmation of negative pregnancy test documented in the Day 1 and/or Day 2 Call CRFs and Daily Diary We will monitor daily responses to the question 'have you become pregnant since starting the trial?' and follow-up as required to immediately stop

For peer review only - http://bmjopen.bmj.com/site/about/guidelines.xhtml
© Copyright: The University of Oxford and Oxford University Hospitals NHS Foundation Trust 2019

NUFFIELD DEPARTMENT OF PRIMARY CARE HEALTH SCIENCES Primary Care Clinical Trials Unit

PANORAMIC Platform Adaptive trial of NOvel antiviRals for eArly treatMent of COVID-19 In the Community

			clinician/research nurse will confirm this exclusion criteria with the participant.<br><br>Participants using combined hormonal contraceptive methods will not be eligible unless willing to use additional barrier methods during treatment with Paxlovid, and until after one complete menstrual cycle after stopping Paxlovid.	treatment, if applicable. Pregnancy occurring during the 28-day trial follow-up period will be reported as an AE of Special Interest. As per 'PC-CTU SOP TM119 Pharmacovigilance', any pregnancy that occurs during IMP administration requires monitoring and follow-up until the outcome of the pregnancy is known. The CI or delegated individual will liaise with the relevant Obstetrician throughout the pregnancy. The DSMC will be informed of any pregnancies in this treatment group.
3. Risk of antiretroviral resistance	L	M	The risk of HIV drug resistance with the short duration and dose of ritonavir is very low.	The risk of HIV drug resistance with the short duration and dose of ritonavir is very low.
4. Unknown/other potential side-effects	M	M	During the eligibility assessment, a medically qualified professional will fully explain the possible	The DSMC will review weekly reports of unblinded symptom data to

For peer review only - http://bmjopen.bmj.com/site/about/guidelines.xhtml
© Copyright: The University of Oxford and Oxford University Hospitals NHS Foundation Trust 2019

NUFFIELD DEPARTMENT OF PRIMARY CARE HEALTH SCIENCES
Primary Care Clinical Trials Unit
PANORAMIC
Platform Adaptive trial of NOvel antiviRals for eArly treatMent of COVID-19 In the Community

			risks associated with Paxlovid treatment to potential participants and advise them on how to report potential side-effects and seek medical care if required.<br><br>All participants will receive a call on day 2, 24h after starting treatment to discuss any side-effects experienced and how to seek medical care if required.<br><br>All participants will receive a call on day 4 to discuss any side-effects experienced and how to seek medical care. This will allow the investigator to detect any early side effects of Paxlovid and to suggest any required changes to the participants medication including stopping medications where required.<br><br>All participants will receive a call on day 10 to discuss any side-effects, this call will allow the investigator to compare the side-effect profile of Paxlovid against the SmPC rather than the Usual Care arm.<br><br>Participants will be provided with a 24-hour contact telephone line to report any AEs that they experience and	identify potential side-effects of Paxlovid. Any safety signals will be communicated to the TSC and TMG as per DSMC Charter.<br><br>The ESG will review accumulating safety data in the Paxlovid arm including AEs, SAEs and laboratory results as defined in the ISA and ESG Charter.<br><br>TMG will review the total number of SAEs as per TMG Charter.

For peer review only - http://bmjopen.bmj.com/site/about/guidelines.xhtml
© Copyright: The University of Oxford and Oxford University Hospitals NHS Foundation Trust 2019

NUFFIELD DEPARTMENT OF PRIMARY CARE HEALTH SCIENCES Primary Care Clinical Trials Unit

PANORAMIC Platform Adaptive trial of NOvel antiviRals for eArly treatMent of COVID-19 In the Community

			are concerned about, directly to a clinician. We will collect symptoms and side effects from symptom diaries and participant telephone calls.	
5. Compliance			Participants will be asked in their daily diaries about trial medication use	The trial team will monitor daily diary responses where the participant indicates that they have taken too much IMP and escalate to the clinical team to follow-up with the participant.

For peer review only

For peer review only - http://bmjopen.bmj.com/site/about/guidelines.xhtml
© Copyright: The University of Oxford and Oxford University Hospitals NHS Foundation Trust 2019

NUFFIELD DEPARTMENT OF PRIMARY CARE HEALTH SCIENCES Primary Care Clinical Trials Unit

PANORAMIC Platform Adaptive trial of NOvel antiviRals for eArly treatMent of COVID-19 In the Community

APPENDIX F: STANDARD SCRIPT FOR SAFETY MONITORING OF DRUGS THAT REQUIRE ADJUSTMENT WHEN CO-ADMINISTERED WITH PAXLOVID

Question 1	Question 2	Action
Are you taking:<br>Buprenorphine<br>*[Brand names: Bunov; Bupeaze; BuTec; BuTrans; BuVidal; Carlosafine; Hapoctasin; Panitas; Prefibin; Rebrikel; Reletrans; Relevtec]*<br><br>Norbuprenorphine	If yes:<br>Have you experienced increased drowsiness	If yes:<br>**Mild/moderate**- counsel about driving / operating machinery<br>Severe- withdraw Paxlovid
Are you taking:<br>Methadone<br>*[Brand name Physeptone]*	If yes:<br>Have you experienced increased withdrawal symptoms [N.B. subjects will know these as given for opioid dependency]	If yes:<br>**Mild**- follow up<br>**Moderate / severe**- withdraw Paxlovid
Are you taking:<br>Morphine<br>*[Brand names: Morphgesic; MST Continus; MXL capsules; Sevredol; Zomorph]*	If yes:<br>Have you experienced breakthrough pain/ increased pain	If yes:<br>**Mild**- recommend simple analgesia<br>**Moderate**- contact GP to request short term dose adjustment<br>**Severe**- withdraw Paxlovid
Are you taking:<br>Afatinib *[Giotrif]*,<br>Ceritinib *[Zykadia]*,<br>Dasatinib *[Sprycel]*,<br>Nilotinib *[Tasigna]*,<br>Vincristine,<br>Vinblastine,<br>Fostamatinib *[Tavlesse]*	If yes:<br>Have you experienced new symptoms of:<br>Bleeding or bruising<br>Nausea, vomiting or diarrhoea<br>Muscle pain or weakness<br>Pins and needles or shooting pains	If yes:<br>Withdraw Paxlovid
Are you taking:<br>Warfarin *[Brand name Marevan]*	If yes:<br>Ask to contact GP (unless unable to do so, in which case the safety monitor can do on their behalf) to organise an INR check on or around day 5 subject to self isolation advice	If the INR is out of range:<br>Withdraw Paxlovid

For peer review only - http://bmjopen.bmj.com/site/about/guidelines.xhtml
© Copyright: The University of Oxford and Oxford University Hospitals NHS Foundation Trust 2019

NUFFIELD DEPARTMENT OF PRIMARY CARE HEALTH SCIENCES Primary Care Clinical Trials Unit

PANORAMIC Platform Adaptive trial of NOvel antiviRals for eArly treatMent of COVID-19 In the Community

APPENDIX G: PAXLOVID DRUG-DRUG INTERACTIONS

How to use this Appendix

List A is a summary list in alphabetical order summarizing medication which is an absolute exclusion criterion for PANORAMIC Paxlovid arm, drugs that should not be included in PANORAMIC because temporary interruption or the monitoring requirements are considered impractical, and those drugs that may be included.

List B contains more detail with the rationale.

These lists are based on the SmPC of 02 March 2022 and UK Interim Clinical Commissioning Policy: Therapies for symptomatic non-hospitalised patients with COVID-19 and a protocol substantial amendment will be submitted to update List B when there are updates to the SmPC.

Clinical judgement is required to evaluate potential drug interactions. Detailed advice is also available from the Liverpool COVID-19 Drug Interactions Checker website. https://www.covid19-druginteractions.org/

List A: Alphabetical summary of drugs that may interact with Paxlovid

Note: You MUST check BOTH columns for each drug

Drugs NOT to be included in PANORAMIC Paxlovid arm	Drugs which may be included in PANORAMIC Paxlovid arm
**Drugs that are contraindicated with Paxlovid and /or because interruption or monitoring requirements considered impractical in the setting of the clinical trial**	**Drugs which may be used with Paxlovid with caution**<br><br>**Those marked with an asterisk have a specific recommendation- see list B for details**<br><br>**The investigator should consider whether inclusion is appropriate**
acalabrutinib<br>abemaciclib<br>aliskiren<br>alfuzosin<br>amiodarone<br>Amlodipine (≥10 mg daily)<br>apalutamide<br>apixaban<br>astemizole<br>atovaquone<br>avanafil<br>bedaquiline<br>bepridil<br>bosentan<br>carbamazepine	afatinib<br>alprazolam*<br>amitriptyline<br>amlodipine (2.5 or 5 mg) *<br>amprenavir<br>atazanavir<br>atorvastatin<br>budesonide<br>buprenorphine*<br>bupropion<br>buspirone*<br>ceritinib<br>clarithromycin*<br>clopidogrel*<br>dabigatran*

For peer review only - http://bmjopen.bmj.com/site/about/guidelines.xhtml
© Copyright: The University of Oxford and Oxford University Hospitals NHS Foundation Trust 2019

cisapride	darunavir
clonazepam	dasatinib
clozapine	dexamethasone
colchicine	diltiazem
cyclosporine	divalproex
dasabuvir	efavirenz
delaminid	ethinyl estradiol*
desipramine	fexofenadine
dexamfetamine	fluoxetine
diazepam	fluticasone propionate
digoxin	fluvastatin
dihydroergotamine	fosamprenavir
disopyramide	fostamatinib
dronedarone	haloperidol*
elbasvir	itraconazole (topical)
eletriptan	ketoconazole (topical)
encainide	lamotrigine
encorafenib	levothyroxine
(enzalutamide- discontinued)	loratadine
eplerenone	maraviroc
ergonovine	methadone*
ergotamine	morphine*
erythromycin	nifedipine
estazolam	nilotinib
everolimus	norbuprenorphine
fentanyl	nortriptyline
flecainide	paroxetine
flurazepam	pravastatin
fusidic acid	prednisolone
glecaprevir	raltegravir
grazoprevir	risperidone*
ibrutinib	rosuvastatin
imipramine	sertraline
isavuconazole	sulfamethoxazole/trimethoprim
itraconazole (systemic)	theophylline
ivabradine	thioridazine*
ketoconazole (systemic)	triamcinolone
lercanidipine	trimethoprim
letermovir	valproic acid
lomitapide	vinblastine
lovastatin	vincristine
lurasidone	voriconazole (topical)
methylergonovine	warfarin*
methylphenidate	zidovudine
midazolam (oral or parenteral)	zolpidem*
neratinib	
pethidine	
phenobarbital	

For peer review only

For peer review only - http://bmjopen.bmj.com/site/about/guidelines.xhtml
© Copyright: The University of Oxford and Oxford University Hospitals NHS Foundation Trust 2019

NUFFIELD DEPARTMENT OF PRIMARY CARE HEALTH SCIENCES Primary Care Clinical Trials Unit PANORAMIC Platform Adaptive trial of NOvel antiviRals for eArly treatMent of COVID-19 In the Community

phenytoin<br>pibrentasvir<br>pimozide<br>piroxicam (systemic)<br>propafenone<br>propoxyphene<br>quetiapine<br>quinidine<br>ranolazine<br>rifabutin<br>rifampicin<br>riociguat<br>rivaroxaban<br>salmeterol<br>sildenafil (Revatio®, for pulmonary arterial hypertension or for erectile dysfunction)<br>simvastatin<br>sirolimus<br>sodium fusidate<br>St. John's Wort (Hypericum perforatum)<br>tadalafil<br>tacrolimus<br>terfenadine<br>ticagrelor<br>triazolam<br>vardenafil<br>venetoclax<br>vorapaxar<br>voriconazole (systemic)	

Details of Paxlovid drug interactions and implications for eligibility for the Paxlovid arm of the PANORAMIC trial

Paxlovid is subject to a large number of drug interactions. At this stage the full range of interactions and their clinical significance is incompletely understood as clinical experience is limited. The PANORAMIC trial participants are home-based and are advised to isolate. This imposes some constraints on drugs which can be safely co-administered with Paxlovid in this context.

The following list is based on the summary of product characteristics (SmPC) list of drugs that are contraindicated for use with Paxlovid or should be used with caution with Paxlovid. For each a recommendation is provided based on a risk assessment. List B will be updated as new information becomes available and a protocol substantial amendment will be submitted for regulatory approval when the list is modified. The list is not exhaustive and if the investigator considers the potential participant is taking a drug which could put the subject at unacceptable risk, they should be excluded. There are some drugs which can be interrupted or adjusted during the trial- a specific recommendation is made for these.

For peer review only - http://bmjopen.bmj.com/site/about/guidelines.xhtml
© Copyright: The University of Oxford and Oxford University Hospitals NHS Foundation Trust 2019

NUFFIELD DEPARTMENT OF PRIMARY CARE HEALTH SCIENCES
Primary Care Clinical Trials Unit
PANORAMIC
Platform Adaptive trial of NOvel antiviRals for eArly treatMent of COVID-19 In the Community

List B: Details of Paxlovid drug interactions and implications for eligibility for drugs that are not recommended or require adjustment with Paxlovid in the PANORAMIC trial

Medicinal product class	Drugs in class with indicative effect of Paxlovid on Concentration of Medicinal product	Clinical comments	Implications for eligibility in PANORAMIC Paxlovid arm
α1-adrenoreceptor antagonist	↑alfuzosin	Increased alfuzosin plasma concentrations may lead to severe hypotension. **Contraindicated.**	**NOT ELIGIBLE**
Aldosterone antagonist	↑Eplerenone	Not recommended with strong 3A4 inhibitor as risk of hyperkalaemia. **Contraindicated.**	**NOT ELIGIBLE**
Amphetamine derivatives	↑methylphenidate, ↑dexamfetamine	Potential for increased concentrations of amphetamine and its derivatives. Careful monitoring of adverse effects is recommended. **Contraindicated.**	**NOT ELIGIBLE**
Analgesics	↑buprenorphine, ↑norbuprenorphine	The increases of plasma levels of buprenorphine and its active metabolite did not lead to clinically significant pharmacodynamic changes in a population of opioid tolerant patients.	**ELIGIBLE** – advise to monitor for opioid toxicity.
	↑pethidine, ↑piroxicam (systemic), ↑propoxyphene	Increased plasma concentrations may result in serious respiratory depression or haematologic abnormalities. **Contraindicated.**	**NOT ELIGIBLE**
	↑fentanyl	Ritonavir expected to increase the plasma concentrations of fentanyl. **Contraindicated.**	**NOT ELIGIBLE**
	↓methadone	Increased methadone dose may be necessary. Dose adjustment should be considered based on the patient's clinical response to methadone therapy.	**ELIGIBLE** – advise to monitor for potential under dosing of methadone.

For peer review only - http://bmjopen.bmj.com/site/about/guidelines.xhtml
© Copyright: The University of Oxford and Oxford University Hospitals NHS Foundation Trust 2019

NUFFIELD DEPARTMENT OF PRIMARY CARE HEALTH SCIENCES — Primary Care Clinical Trials Unit — PANORAMIC Platform Adaptive trial of NOvel antiviRals for eArly treatMent of COVID-19 In the Community

	↓morphine	Morphine levels may be decreased due to induction of glucuronidation by co-administered ritonavir dosed as a pharmacokinetic enhancer.	**ELIGIBLE** – advise to monitor for morphine underdosing and adjust dose if required.
Antianginal	↑ranolazine	Potentially increased ranolazine plasma concentrations may result in serious and/or life-threatening reactions.<br><br>**Contraindicated.**	**NOT ELIGIBLE**
Antiarrhythmics	↑amiodarone<br>↑bepridil<br>↑disopyramide,<br>↑dronedarone,<br>↑encainide,<br>↑flecainide,<br>↑ivabradine<br>↑propafenone,<br>↑quinidine	Potentially increased plasma concentrations may result in arrhythmias or other serious adverse effects.<br><br>**Contraindicated.**	**NOT ELIGIBLE**
	↑digoxin	Potentially increased concentrations. Inhibition of pgp may decrease renal digoxin clearance. Magnitude of effect not known.<br><br>**Contraindicated.**	**NOT ELIGIBLE**
Antiasthmatic	↓theophylline	Ritonavir could potentially decrease theophylline concentrations, but effects unlikely with short course of Paxlovid.	**ELIGIBLE** – no theophylline dose adjustment required.
Anticancer agents	↑afatinib,<br>↑ceritinib, ↑dasatinib,<br>↑nilotinib,<br>↑vincristine,<br>↑vinblastine,<br>↑fostamatinib	Serum concentrations may be increased when co-administered with ritonavir resulting in the potential for increased incidence of AEs.	**ELIGIBLE** with caution. Advise to monitor symptoms of increased anticancer agent concentrations.
	↑acalabrutinib<br>↑apalutamide,<br>↑abemaciclib,<br>↑encorafenib,<br>↑ibrutinib	Co-administration not recommended due to potentially increased concentration of anticancer agents and SAEs. Apalutamide may also decrease exposure of Paxlovid and cause potential loss of virologic response.	**NOT ELIGIBLE**

For peer review only

For peer review only - http://bmjopen.bmj.com/site/about/guidelines.xhtml
© Copyright: The University of Oxford and Oxford University Hospitals NHS Foundation Trust 2019

		**Contraindicated.**	
	↑neratinib, ↑venetoclax	Increased plasma concentrations which may increase the potential for serious and/or life-threatening reactions.<br><br>**Contraindicated.**	**NOT ELIGIBLE**
Anticoagulants	warfarin, ↑↓S-warfarin, ↓↔R-warfarin	Potentially decreased R-warfarin concentrations which may lead to reduced anticoagulation.	**ELIGIBLE** – recommend check INR on or around day 5 (as self-isolation allows)
	↑apixaban,	Potentially increased concentrations which may lead to an increased bleeding risk.<br><br>**Contraindicated.**	**NOT ELIGIBLE** as contraindicated.
	↑dabigatran	Potentially increased concentrations which may lead to an increased bleeding risk. No effect when co-administered with ritonavir (small effect when given at different time).	**ELIGIBLE –** recommend taking Paxlovid simultaneously with dabigatran
	↑rivaroxaban	Potentially increased concentrations which may lead to an increased bleeding risk.<br><br>**Contraindicated.**	**NOT ELIGIBLE**
	↑vorapaxar	Serum concentrations may be increased. Coadministration not recommended.<br><br>**Contraindicated.**	**NOT ELIGIBLE** as contraindicated.
Anticonvulsants	carbamazepine, phenobarbital<br>These drugs are expected to reduce the concentrations of Paxlovid	Decreased plasma concentrations of Paxlovid may lead to loss of virologic response and possible resistance.<br><br>**Contraindicated.**	**NOT ELIGIBLE** as contraindicated.

For peer review only - http://bmjopen.bmj.com/site/about/guidelines.xhtml
© Copyright: The University of Oxford and Oxford University Hospitals NHS Foundation Trust 2019

NUFFIELD DEPARTMENT OF PRIMARY CARE HEALTH SCIENCES Primary Care Clinical Trials Unit

PANORAMIC Platform Adaptive trial of NOvel antiviRals for eArly treatMent of COVID-19 In the Community

	↓phenytoin	Ritonavir is expected to decrease the plasma concentrations of phenytoin. Phenytoin may decrease serum levels of ritonavir.<br><br>**Contraindicated.**	**NOT ELIGIBLE**
	↓divalproex, valproic acid ↓lamotrigine	Ritonavir may decrease the plasma concentrations of anticonvulsants over time but given the short course of Paxlovid treatment, no a priori dosage adjustment is recommended.	**ELIGIBLE**
Antidepressants	↑amitriptyline,<br><br>↑fluoxetine,<br>↑nortriptyline,<br>↑paroxetine,<br>↑sertraline	Ritonavir used at higher doses than present in Paxlovid may increase concentrations of these antidepressants. With Paxlovid no a priori dosage adjustment is recommended.	**ELIGIBLE**
	↑desipramine,	Dosage reduction is recommended when co-administered.	**NOT ELIGIBLE**
	↑imipramine	Nirmatrelvir/ritonavir could potentially increase imipramine concentrations and increase the risk of QT prolongation.<br><br>**Contraindicated.**	**NOT ELIGIBLE**
Anti-gout	↑colchicine	Increased colchicine plasma concentrations may result in serious and/or life-threatening reactions.<br><br>**Contraindicated.**	**NOT ELIGIBLE**
Antihistamines	↑astemizole,<br>↑terfenadine	Increased plasma concentrations of astemizole and terfenadine may result in serious arrhythmias from these agents. Note both withdrawn from market globally.<br><br>**Contraindicated.**	**NOT ELIGIBLE**
	↑fexofenadine<br>↑loratadine	Ritonavir may increase fexofenadine and loratadine concentrations.	**ELIGIBLE**

For peer review only

For peer review only - http://bmjopen.bmj.com/site/about/guidelines.xhtml

© Copyright: The University of Oxford and Oxford University Hospitals NHS Foundation Trust 2019

Antifungals	↑ketoconazole, ↓voriconazole, ↑itraconazole, ↑isavuconazole	Potentially increased concentrations of ketoconazole, isavuconazole and itraconazole, and decreased plasma concentrations of voriconazole.<br><br>**Systemic use contraindicated.**	**NOT ELIGIBLE** when administered systemically. Topically used agents are not an exclusion criterion.
Antimycobacterial	rifampicin	Potentially decreased concentrations of Paxlovid may lead to loss of virologic response and possible resistance.<br><br>**Contraindicated.**	**NOT ELIGIBLE**
	↑bedaquiline, ↑delaminid ↑rifabutin	Potentially increased plasma concentrations of bedaquiline, delaminid and rifabutin.<br><br>**Contraindicated.**	**NOT ELIGIBLE**
Anti-infective	↑clarithromycin ↓14-OH clarithromycin metabolite	Potentially increased plasma concentrations of clarithromycin. Clarithromycin doses greater than 1 gr per day should not be co-administered with Paxlovid. For patients with renal impairment, a clarithromycin dose reduction should be considered: for patients with creatinine clearance of 30 to 60 ml/min the dose should be reduced by 50%, for patients with creatinine clearance less than 30 ml/min the dose should be reduced by 75%.	**Potentially ELIGIBLE** if no known renal impairment. If known renal impairment, **NOT ELIGIBLE.**
	↑erythromycin	Ritonavir is expected to increase plasma concentrations of erythromycin which may increase risk of QT prolongation.<br><br>**Contraindicated.**	**NOT ELIGIBLE**
	sulfamethoxazole/trimethoprim	Dose alteration of sulfamethoxazole/trimethoprim should not be necessary.	**ELIGIBLE**
	↑fusidic acid / sodium fusidate	Increased plasma concentrations of fusidic acid and ritonavir.<br><br>**Contraindicated.**	**NOT ELIGIBLE**

For peer review only - http://bmjopen.bmj.com/site/about/guidelines.xhtml
© Copyright: The University of Oxford and Oxford University Hospitals NHS Foundation Trust 2019

Anti-malarial	↓atovaquone	Ritonavir is expected to decrease the plasma concentrations of atovaquone.<br><br>**Contraindicated.**	**NOT ELIGIBLE**
Anti-platelet	↓Clopidogrel	Paxlovid may reduce conversion to active drug. Avoid in in patients at high risk of thrombosis and those within 6 weeks of stenting.	**POTENTIALLY ELIGIBLE. If within 6 weeks of coronary stent or at high risk of thrombosis NOT ELIGIBLE**
	↑ticagrelor	Expected to substantially increase exposure to ticagrelor.<br><br>**Contraindicated.**	**NOT ELIGIBLE**
Anti-HIV protease inhibitors	↑amprenavir,<br>↑atazanavir,<br>↑darunavir,<br>↑fosamprenavir	Potentially increased concentrations of protease inhibitors, but consensus is that no dose adjustment needed.	**ELIGIBLE**
Anti-HIV	↑efavirenz,<br>↑maraviroc	Potentially increased plasma concentrations of efavirenz and maraviroc.	**ELIGIBLE** – advise monitor for potential side effects.
	↓raltegravir,<br>↓zidovudine	Potentially minor decreased plasma concentrations of raltegravir and zidovudine.	**ELIGIBLE** – no dose adjustments required.
Antiviral	Letermovir. This drug is expected to reduce concentrations of Paxlovid.	Letermovir is an enzyme inducer so may render Paxlovid ineffective.<br><br>**Contraindicated.**	**NOT ELIGIBLE**
Antipsychotics	↑clozapine,<br>↑pimozide,<br>↑lurasidone<br>↑quetiapine	Increased concentrations may result in serious and/or life-threatening reactions.<br><br>**Contraindicated.**	**NOT ELIGIBLE**
	↑Haloperidol,<br>↑Risperidone,<br>↑Thioridazine	Ritonavir is likely to increase concentrations of haloperidol, risperidone and thioridazine.	**ELIGIBLE** – with caution and advise to monitor for increased adverse effects.
Long-acting beta-adrenoceptor agonist	↑salmeterol	Ritonavir is expected to increase the plasma concentrations of salmeterol, and may increase risk of QT prolongation, palpitations, and sinus tachycardia. Therefore,	**NOT ELIGIBLE**

For peer review only

For peer review only - http://bmjopen.bmj.com/site/about/guidelines.xhtml
© Copyright: The University of Oxford and Oxford University Hospitals NHS Foundation Trust 2019

		concomitant use is not recommended.<br><br>**Contraindicated.**	
Calcium channel antagonist	↑amlodipine,	Ritonavir is expected to increase the plasma concentrations of calcium channel antagonists.	**Potentially ELIGIBLE** – if taking 2.5 or 5 mg. If taking 10 mg or able **NOT ELIGIBLE**
	↑diltiazem,<br>↑nifedipine	Ritonavir may increase the plasma concentrations of calcium channel antagonists.	**ELIGIBLE** – advise to monitor for side effects.
	↑lercanidipine	Expected to substantially increase exposure to lercanidipine.<br><br>**Contraindicated.**	**NOT ELIGIBLE**
Endothelin receptor antagonists	↑bosentan<br>↑riociguat	Potentially increased concentrations.<br><br>**Contraindicated.**	**NOT ELIGIBLE**
Ergot Derivatives	↑dihydroergotamine,<br>↑ergonovine,<br>↑ergotamine,<br>↑methylergonovine	Increased concentrations of ergot derivatives potentially leading to acute ergot toxicity, including vasospasm and ischaemia.<br><br>**Contraindicated.**	**NOT ELIGIBLE**
GI motility agent	↑cisapride	Increased plasma concentrations of cisapride, thereby increasing the risk of serious arrhythmias from this agent.<br><br>**Contraindicated.**	**NOT ELIGIBLE**
Hepatitis C direct acting antivirals	↑elbasvir/grazoprevir,<br>↑glecaprevir/pibrentasvir<br>↑dasabuvir	Serum concentrations may be increased by ritonavir, leading to an increased risk of ALT elevations associated with increased glecaprevir and grazoprevir exposure.<br><br>**Contraindicated.**	**NOT ELIGIBLE**
Herbal products	St. John's Wort (*Hypericum perforatum*)<br>This drug is expected to reduce concentrations of Paxlovid	Potentially decreased concentrations of Paxlovid may lead to loss of virologic response and possible resistance.<br><br>**Contraindicated.**	**NOT ELIGIBLE**

For peer review only - http://bmjopen.bmj.com/site/about/guidelines.xhtml
© Copyright: The University of Oxford and Oxford University Hospitals NHS Foundation Trust 2019

HMG-CoA reductase inhibitors	↑lovastatin, ↑simvastatin	Increased concentrations resulting in increased risk of myopathy, including rhabdomyolysis. **Contraindicated.**	**NOT ELIGIBLE**
	↑atorvastatin, ↑fluvastatin, ↑pravastatin, ↑rosuvastatin,	Potentially increased concentrations.	**ELIGIBLE** – advise to monitor for side effects.
Microsomal triglyceride transfer protein (MTTP) inhibitor	↑lomitapide	Increased plasma concentrations of lomitapide.<br><br>**Contraindicated.**	**NOT ELIGIBLE**
Migraine treatments	↑Eletriptan	Not recommended with strong 3A4 inhibitors.<br><br>**Contraindicated.**	**NOT ELIGIBLE**
Hormonal contraceptive	↓ethinyl estradiol	Ritonavir may reduce ethinyl estradiol concentrations and change the uterine bleeding profile and reduce the effectiveness of estradiol-containing contraceptives.	**Potentially ELIGIBLE** if willing to use an additional barrier method during treatment with Paxlovid, and until one full menstrual cycle after stopping Paxlovid.
Immunosuppressants	↑cyclosporine, ↑tacrolimus, ↑everolimus ↑sirolimus	Ritonavir is expected to increase the plasma concentrations of cyclosporine, tacrolimus, sirolimus or everolimus.<br><br>**Contraindicated.**	**NOT ELIGIBLE**
Phosphodiesterase (PDE5) Inhibitors	↑ avanafil ↑ vardenafil	Increased plasma concentrations of avanafil and vardenafil.<br><br>**Contraindicated.**	**NOT ELIGIBLE**
	↑sildenafil (Revatio®) used for pulmonary arterial hypertension (PAH)	Increased sildenafil concentrations can potentially result in visual abnormalities, hypotension, prolonged erection, and syncope.<br><br>**Contraindicated.**	**NOT ELIGIBLE**
	↑sildenafil for erectile dysfunction	**Contraindicated.**	**NOT ELIGIBLE**
	↑tadalafil	**Contraindicated.**	**NOT ELIGIBLE**
Renin inhibitor	↑Aliskiren	Not recommended with a 3A4 and pgp inhibitor.	**NOT ELIGIBLE**

For peer review only - http://bmjopen.bmj.com/site/about/guidelines.xhtml
© Copyright: The University of Oxford and Oxford University Hospitals NHS Foundation Trust 2019

		**Contraindicated.**	
Sedative/hypnotics	↑clonazepam, ↑diazepam, ↑estazolam, ↑flurazepam, ↑oral and parenteral midazolam, ↑triazolam	Increased concentrations of can increase risk of extreme sedation and respiratory depression.<br><br>**Contraindicated.**	**NOT ELIGIBLE**
	↑alprazolam ↑buspirone	Potentially increased concentrations of alprazolam and buspirone.	**ELIGIBLE** – but advise to monitor for side effects and drowsiness.
Sleeping agent	↑zolpidem	Zolpidem & ritonavir may be co-administered with careful monitoring for excessive sedative effects.	**ELIGIBLE** – but advise to monitor for side effects and drowsiness.
Smoke cessation	↓bupropion	Concurrent administration of bupropion with repeated doses of ritonavir is expected to decrease bupropion levels. Effects may not be seen with the short course of Paxlovid.	**ELIGIBLE**
Steroids	Inhaled, injectable or intranasal fluticasone propionate, budesonide, triamcinolone	Systemic corticosteroid effects have been reported in patients receiving long-term ritonavir and inhaled or intranasal fluticasone propionate<br>Given the short course of Paxlovid this risk is considered to be low.	**ELIGIBLE**
	↑dexamethasone, ↑prednisolone	Ritonavir is expected to increase concentrations of dexamethasone and prednisolone. However, given the short duration of Paxlovid treatment, this risk is considered to be low.	**ELIGIBLE**
Thyroid hormone replacement therapy	levothyroxine (no interaction expected)	Post-marketing cases have been reported indicating a potential interaction between ritonavir containing products and levothyroxine. Given the short duration of Paxlovid treatment, this risk is considered to be low.	**ELIGIBLE**

For peer review only - http://bmjopen.bmj.com/site/about/guidelines.xhtml
© Copyright: The University of Oxford and Oxford University Hospitals NHS Foundation Trust 2019

NUFFIELD DEPARTMENT OF PRIMARY CARE HEALTH SCIENCES Primary Care Clinical Trials Unit

PANORAMIC Platform Adaptive trial of NOvel antiviRals for eArly treatMent of COVID-19 In the Community

20. REFERENCES

1. Hippisley-Cox J, Coupland CA, Mehta N, Keogh RH, Diaz-Ordaz K, Khunti K, et al. Risk prediction of covid-19 related death and hospital admission in adults after covid-19 vaccination: national prospective cohort study. BMJ. 2021;374:n2244.

2. Stokel-Walker C. The search for antivirals for covid-19. BMJ. 2021;374:n2165.

3. Kim PS, Read SW, Fauci AS. Therapy for Early COVID-19: A Critical Need. JAMA. 2020;324(21):2149-50.

4. Cevik M, Tate M, Lloyd O, Maraolo AE, Schafers J, Ho A. SARS-CoV-2, SARS-CoV, and MERS-CoV viral load dynamics, duration of viral shedding, and infectiousness: a systematic review and meta-analysis. Lancet Microbe. 2021;2(1):e13-e22.

5. van Hout B, Janssen MF, Feng YS, Kohlmann T, Busschbach J, Golicki D, et al. Interim scoring for the EQ-5D-5L: mapping the EQ-5D-5L to EQ-5D-3L value sets. Value Health. 2012;15(5):708-15.

6. National Institute for Health and Care Excellence (NICE). NICE Guide to the methods of technology appraisal London, UK: NICE; 2013 [Available from: http://www.nice.org.uk/article/pmg9/resources/non-guidance-guide-to-the-methods-oftechnology-appraisal-2013-pdf.

7. HM Treasury. The Green Book. Appraisal and Evaluation in Central Government. 2020 [Available from: https://www.gov.uk/government/publications/the-green-book-appraisal-and-evaluationin-central-governent.

8. Yu LM, Bafadhel M, Dorward J, Hayward G, Saville BR, Gbinigie O, et al. Inhaled budesonide for COVID-19 in people at high risk of complications in the community in the UK (PRINCIPLE): a randomised, controlled, open-label, adaptive platform trial. Lancet. 2021;398(10303):843-55.

9. Dorward J, Yu LM, Hayward G. Colchicine for COVID-19 in adults in the community (PRINCIPLE): a randomised, controlled, adaptive platform trial. medRxiv 2021.

10. Gastine S, Pang J, Boshier FAT, Carter SJ, Lonsdale DO, Cortina-Borja M, et al. Systematic Review and Patient-Level Meta-Analysis of SARS-CoV-2 Viral Dynamics to Model Response to Antiviral Therapies. Clin Pharmacol Ther. 2021;110(2):321-33.

11. Laouenan C, Guedj J, Mentre F. Clinical trial simulation to evaluate power to compare the antiviral effectiveness of two hepatitis C protease inhibitors using nonlinear mixed effect models: a viral kinetic approach. BMC Med Res Methodol. 2013;13:60.

12. Amara A, Penchala SD, Else L, Hale C, FitzGerald R, Walker L, et al. The development and validation of a novel LC-MS/MS method for the simultaneous quantification of Molnupiravir and its metabolite ss-d-N4-hydroxycytidine in human plasma and saliva. J Pharm Biomed Anal. 2021;206:114356.

13. Fischer W, Eron JJ, Holman W, Cohen MS, Fang L, Szewczyk LJ, et al. Molnupiravir, an Oral Antiviral Treatment for COVID-19. medRxiv. 2021.

14. Imran M, Kumar Arora M, Asdaq SMB, Khan SA, Alaqel SI, Alshammari MK, et al. Discovery, Development, and Patent Trends on Molnupiravir: A Prospective Oral Treatment for COVID-19. Molecules. 2021;26(19).

15. Abdelnabi R, Foo CS, Kaptein SJF, Zhang X, Do TND, Langendries L, et al. The combined treatment of Molnupiravir and Favipiravir results in a potentiation of antiviral efficacy in a SARS-CoV-2 hamster infection model. EBioMedicine. 2021;72:103595.

16. Singh AK, Singh A, Singh R, Misra A. Molnupiravir in COVID-19: A systematic review of literaturef. Diabetes Metab Syndr. 2021;15(6):102329.

For peer review only

For peer review only - http://bmjopen.bmj.com/site/about/guidelines.xhtml
© Copyright: The University of Oxford and Oxford University Hospitals NHS Foundation Trust 2019

17. Khoo SH, Fitzgerald R, Fletcher T, Ewings S, Jaki T, Lyon R, et al. Optimal dose and safety of Molnupiravir in patients with early SARS-CoV-2: a Phase I, open-label, dose-escalating, randomized controlled study. J Antimicrob Chemother. 2021.

18. Mahase E. Covid-19: Molnupiravir reduces risk of hospital admission or death by 50% in patients at risk, MSD reports. BMJ. 2021;375:n2422.

19. Owen DR, Allerton CMN, Anderson AS, et al. An oral SARS-CoV-2 M(pro) inhibitor clinical candidate for the treatment of COVID-19. *Science* 2021; **374**(6575): 1586-93.

20. ClinicalTrials.gov. Study of PF-07321332 in health participants. 2021. https://clinicaltrials.gov/ct2/show/NCT04756531.

21. Hammond J, Leister-Tebbe H, Gardner A, et al. Oral Nirmatrelvir for High-Risk, Nonhospitalized Adults with Covid-19. N Engl J Med 2022.

22.Pfizer Limited. Paxlovid 150 mg/100 mg film-coated tablets. Summary of Product Characteristics. Updated 02-Mar-2022.

23. Pfizer. Evaluation of Protease Inhibition for COVID-19 in Standard-Risk Patients (EPIC-SR) (NCT05011513). 2021. https://clinicaltrials.gov/ct2/show/NCT05011513 (accessed 06 Jan 2022.

24. The Electronic Medicines Compendium (EMC). Norvir 100mg film-coated tablets. 2022. https://www.medicines.org.uk/emc/product/510/smpc.

25. University of Liverpool. COVID-19 Drug interaction checker. 2022. https://www.covid19-druginteractions.org/ (accessed 06 Jan 2022.

For peer review only

For peer review only - http://bmjopen.bmj.com/site/about/guidelines.xhtml
© Copyright: The University of Oxford and Oxford University Hospitals NHS Foundation Trust 2019

STANDARD PROTOCOL ITEMS: RECOMMENDATIONS FOR INTERVENTIONAL TRIALS

SPIRIT 2013 Checklist: Recommended items to address in a clinical trial protocol and related documents*

Section/item	Item No	Description	Page Number on which item is reported
**Administrative information**			
Title	1	Descriptive title identifying the study design, population, interventions, and, if applicable, trial acronym	1
Trial registration	2a	Trial identifier and registry name. If not yet registered, name of intended registry	6
	2b	All items from the World Health Organization Trial Registration Data Set	6
Protocol version	3	Date and version identifier	24
Funding	4	Sources and types of financial, material, and other support	30
Roles and responsibilities	5a	Names, affiliations, and roles of protocol contributors	1-3 & 31-32
	5b	Name and contact information for the trial sponsor	24
	5c	Role of study sponsor and funders, if any, in study design; collection, management, analysis, and interpretation of data; writing of the report; and the decision to submit the report for publication, including whether they will have ultimate authority over any of these activities	30
	5d	Composition, roles, and responsibilities of the coordinating centre, steering committee, endpoint adjudication committee, data management team, and other individuals or groups overseeing the trial, if applicable (see Item 21a for data monitoring committee)	30-31
**Introduction**			

For peer review only

Background and rationale	6a	Description of research question and justification for undertaking the trial, including summary of relevant studies (published and unpublished) examining benefits and harms for each intervention	8-9
	6b	Explanation for choice of comparators	8-9
Objectives	7	Specific objectives or hypotheses	9
Trial design	8	Description of trial design including type of trial (e.g. parallel group, crossover, factorial, single group), allocation ratio, and framework (e.g. superiority, equivalence, non-inferiority, exploratory)	9-10
**Methods: Participants, interventions, and outcomes**			
Study setting	9	Description of study settings (e.g. community clinic, academic hospital) and list of countries where data will be collected. Reference to where list of study sites can be obtained	11-12
Eligibility criteria	10	Inclusion and exclusion criteria for participants. If applicable, eligibility criteria for study centres and individuals who will perform the interventions (e.g. surgeons, psychotherapists)	12-13
Interventions	11a	Interventions for each group with sufficient detail to allow replication, including how and when they will be administered	15-16
	11b	Criteria for discontinuing or modifying allocated interventions for a given trial participant (e.g. drug dose change in response to harms, participant request, or improving/worsening disease)	N/A
	11c	Strategies to improve adherence to intervention protocols, and any procedures for monitoring adherence (e.g. drug tablet return, laboratory tests)	11-12
	11d	Relevant concomitant care and interventions that are permitted or prohibited during the trial	15-16
Outcomes	12	Primary, secondary, and other outcomes, including the specific measurement variable (e.g. systolic blood pressure), analysis metric (e.g. change from baseline, final value, time to event), method of aggregation (e.g. median, proportion), and time point for each outcome. Explanation of the clinical relevance of chosen efficacy and harm outcomes is strongly recommended	18 and 21-22

For peer review only - http://bmjopen.bmj.com/site/about/guidelines.xhtml

Participant timeline	13	Time schedule of enrolment, interventions (including any run-ins and washouts), assessments, and visits for participants. A schematic diagram is highly recommended (see Figure)	16-18
Sample size	14	Estimated number of participants needed to achieve study objectives and how it was determined, including clinical and statistical assumptions supporting any sample size calculations	19-20
Recruitment	15	Strategies for achieving adequate participant enrolment to reach target sample size	13-14 & 27-28
**Methods: Assignment of interventions (for controlled trials)**			
Allocation:			
Sequence generation	16a	Method of generating the allocation sequence (eg, computer-generated random numbers), and list of any factors for stratification. To reduce predictability of a random sequence, details of any planned restriction (e.g. blocking) should be provided in a separate document that is unavailable to those who enrol participants or assign interventions	14-15
Allocation concealme nt mechanis m	16b	Mechanism of implementing the allocation sequence (e.g. central telephone; sequentially numbered, opaque, sealed envelopes), describing any steps to conceal the sequence until interventions are assigned	14-15
Implement ation	16c	Who will generate the allocation sequence, who will enrol participants, and who will assign participants to interventions	14-15
Blinding (masking)	17a	Who will be blinded after assignment to interventions (e.g. trial participants, care providers, outcome assessors, data analysts), and how	15
	17b	If blinded, circumstances under which unblinding is permissible, and procedure for revealing a participant's allocated intervention during the trial	N/A
**Methods: Data collection, management, and analysis**			

For peer review only - http://bmjopen.bmj.com/site/about/guidelines.xhtml

Data collection methods	18a	Plans for assessment and collection of outcome, baseline, and other trial data, including any related processes to promote data quality (e.g. duplicate measurements, training of assessors) and a description of study instruments (e.g. questionnaires, laboratory tests) along with their reliability and validity, if known. Reference to where data collection forms can be found, if not in the protocol	15-17 and 22-23
	18b	Plans to promote participant retention and complete follow-up, including list of any outcome data to be collected for participants who discontinue or deviate from intervention protocols	13-14 & 27-28
Data management	19	Plans for data entry, coding, security, and storage, including any related processes to promote data quality (e.g. double data entry; range checks for data values). Reference to where details of data management procedures can be found, if not in the protocol	14
Statistical methods	20a	Statistical methods for analysing primary and secondary outcomes. Reference to where other details of the statistical analysis plan can be found, if not in the protocol	18-19
	20b	Methods for any additional analyses (e.g. subgroup and adjusted analyses)	21-23
	20c	Definition of analysis population relating to protocol non-adherence (e.g. as randomised analysis), and any statistical methods to handle missing data (e.g. multiple imputation)	20
**Methods: Monitoring**			
Data monitoring	21a	Composition of data monitoring committee (DMC); summary of its role and reporting structure; statement of whether it is independent from the sponsor and competing interests; and reference to where further details about its charter can be found, if not in the protocol. Alternatively, an explanation of why a DMC is not needed	24 & 30-31
	21b	Description of any interim analyses and stopping guidelines, including who will have access to these interim results and make the final decision to terminate the trial	18-19

Harms	22	Plans for collecting, assessing, reporting, and managing solicited and spontaneously reported adverse events and other unintended effects of trial interventions or trial conduct	20-21
Auditing	23	Frequency and procedures for auditing trial conduct, if any, and whether the process will be independent from investigators and the sponsor	20-21
**Ethics and dissemination**			
Research ethics approval	24	Plans for seeking research ethics committee/institutional review board (REC/IRB) approval	24
Protocol amendments	25	Plans for communicating important protocol modifications (e.g. changes to eligibility criteria, outcomes, analyses) to relevant parties (e.g. investigators, REC/IRBs, trial participants, trial registries, journals, regulators)	24
Consent or assent	26a	Who will obtain informed consent or assent from potential trial participants or authorised surrogates, and how (see Item 32)	18-19
	26b	Additional consent provisions for collection and use of participant data and biological specimens in ancillary studies, if applicable	18-19 and 22
Confidentialit y	27	How personal information about potential and enrolled participants will be collected, shared, and maintained in order to protect confidentiality before, during, and after the trial	18
Declaration of interests	28	Financial and other competing interests for principal investigators for the overall trial and each study site	30-31
Access to data	29	Statement of who will have access to the final trial dataset, and disclosure of contractual agreements that limit such access for investigators	18
Ancillary and post-trial care	30	Provisions, if any, for ancillary and post-trial care, and for compensation to those who suffer harm from trial participation	24
Disseminatio n policy	31a	Plans for investigators and sponsor to communicate trial results to participants, healthcare professionals, the public, and other relevant groups (e.g. via publication, reporting in results databases, or other data sharing arrangements), including any publication restrictions	24

For peer review only

	31b	Authorship eligibility guidelines and any intended use of professional writers	N/A
	31c	Plans, if any, for granting public access to the full protocol, participant-level dataset, and statistical code	N/A
**Appendices**			
Informed consent materials	32	Model consent form and other related documentation given to participants and authorised surrogates	Additional file
Biological specimens	33	Plans for collection, laboratory evaluation, and storage of biological specimens for genetic or molecular analysis in the current trial and for future use in ancillary studies, if applicable	21-23

*It is strongly recommended that this checklist be read in conjunction with the SPIRIT 2013 Explanation & Elaboration for important clarification on the items. Amendments to the protocol should be tracked and dated. The SPIRIT checklist is copyrighted by the SPIRIT Group under the Creative Commons "Attribution-NonCommercial-NoDerivs 3.0 Unported" license.

For peer review only
